# Integration of segmented regression analysis with weighted gene correlation network analysis identifies genes whose expression is remodeled throughout physiological aging in mouse tissues

**DOI:** 10.18632/aging.203379

**Published:** 2021-07-29

**Authors:** Margarida Ferreira, Stephany Francisco, Ana R. Soares, Ana Nobre, Miguel Pinheiro, Andreia Reis, Sonya Neto, Ana João Rodrigues, Nuno Sousa, Gabriela Moura, Manuel A. S. Santos

**Affiliations:** 1 Institute of Biomedicine – iBiMED, Department of Medical Sciences, University of Aveiro, Aveiro 3810-193, Portugal; 2 Life and Health Sciences Research Institute (ICVS), School of Medicine, University of Minho, Braga 4710-057, Portugal; 3 ICVS/3B’s–PT Government Associate Laboratory, Braga/Guimarães, Portugal

**Keywords:** aging, transcriptome, mus musculus, trendy, WGCNA

## Abstract

Gene expression alterations occurring with aging have been described for a multitude of species, organs, and cell types. However, most of the underlying studies rely on static comparisons of mean gene expression levels between age groups and do not account for the dynamics of gene expression throughout the lifespan. These studies also tend to disregard the pairwise relationships between gene expression profiles, which may underlie commonly altered pathways and regulatory mechanisms with age. To overcome these limitations, we have combined segmented regression analysis with weighted gene correlation network analysis (WGCNA) to identify high-confidence signatures of aging in the brain, heart, liver, skeletal muscle, and pancreas of C57BL/6 mice in a publicly available RNA-Seq dataset (GSE132040). Functional enrichment analysis of the overlap of genes identified in both approaches showed that immune- and inflammation-related responses are prominently altered in the brain and the liver, while in the heart and the muscle, aging affects amino and fatty acid metabolism, and tissue regeneration, respectively, which reflects an age-related global loss of tissue function. We also explored sexual dimorphism in the aging mouse transcriptome and found the liver and the muscle to have the most pronounced gender differences in gene expression throughout the lifespan, particularly in proteostasis-related pathways. While the data showed little overlap among the age-dysregulated genes between tissues, aging triggered common biological processes in distinct tissues, which we highlight as important features of murine tissue physiological aging.

## INTRODUCTION

Gene expression alterations occurring throughout the lifespan have been described for a multitude of species, organs, and cell types [1–10]. The most commonly reported age-related dysregulations involve the immune system [9, 11–13] where inflammatory response genes are upregulated even in the absence of pathogen infection [5, 6, 9, 11, 14–19]. Energy metabolism, redox homeostasis, and mitochondrial function alterations are also frequently observed in age-related studies [6, 9, 11, 15–18, 20], particularly the downregulation of genes encoding mitochondrial ribosomal proteins and components of the electron transport chain [5, 11, 14–16, 18], protein synthesis machinery [5, 11, 17], developmental and cell differentiation genes [9, 11, 19], and extracellular matrix components [6, 14–16]. Up-regulated genes are associated with the stress response and DNA repair [5, 6, 9, 11, 14, 16–18], RNA processing [11, 12, 17] and cell cycle arrest [5, 16, 19]. Despite this, the existence of specific genetic signatures of aging continue to be a matter of debate as gene regulation is mostly tissue- [5–7, 15, 20–24] and cell-specific [25, 26], but also because there is focus on comparisons between young and old individuals without much consideration of the dynamics of gene expression throughout the lifespan. Nonetheless, recent studies in humans and animal models shed light on these dynamics. As an example, a marked shift in mRNA and microRNA expression has been reported to occur at around age 20 in the human prefrontal cortex [27, 28]. Less striking alterations were reported in the same brain region between 30-60 years [28, 29], entailing genes related to the synapse, fatty acid metabolism, purine nucleotide binding, ubiquitin proteolysis, channel activity, translation, DNA damage response, transcriptional activation, and neuronal function [30]. Late middle-age and early old-age shifts have also been described in human peripheral blood leukocytes for genes involved in cancer, hematological and immunological diseases, cell-mediated immune response and signaling pathways [31], and in the human brain and muscle for both coding and non-coding RNAs pertaining to longevity pathways [32].

Similar findings have been reported in a study across 11 rat organs where the most frequent changes in gene expression occurred at around 6 and 21 months [33], proposed to be equivalent to middle-age in humans [34]. Another study across 17 mouse tissues, whose dataset we re-analyzed in this work, identified shift points of gene expression trajectories at around 6 months for extracellular matrix genes, 10 months for mitochondrial genes, 12 months for genes encoding heat shock proteins, and at around 15 months for immune response genes [6]. Other studies also suggest tissue-specific turning points in gene expression profiles [6, 20, 35]. For example, immune response gene expression was found to change in the mouse kidney between 13 and 20 months, in line with the previously described organismal trend, whereas in the spleen and lung this shift occurs later in life, at around 26 months [35].

Interestingly, various works show prominent sex-differences in gene expression and regulation in mammals, affecting processes such as hormone secretion, immune response, extracellular matrix organization, oxidoreductase activity, lipid metabolism, nucleotide metabolism, cytoplasmic and mitochondrial translation, RNA helicase activity, ribosomal RNA processing, synaptic plasticity, and neurotransmitter transport [36–38, recently reviewd in 39]. However, evidence regarding gender-biased gene expression across the lifespan is much scarcer. A recent study in mice found sex-specific differences in the aging of cells of renin lineage in the kidney, with aged females exhibiting up-regulation of genes involved in angiogenesis, apoptosis, epithelial to mesenchymal transition, and TGFβ signaling, whereas in aged males these genes were down-regulated [40]. Another study showed age-related induction of genes involved in the apoptosis of microglia in the retinas of old female mice, while in old males the expression of these genes was not affected by aging [41]. Sex differences across the lifespan have also been described for the mouse hippocampus, with aged females exhibiting activation of genes involved in inflammatory processes, when compared with aged males [42].

These studies have mainly focused on differential gene expression to characterize the aging transcriptome, using methods that rely on static comparisons of mean expression values between consecutive age groups, or relative to a reference time point, thus ignoring the influence of gene expression levels in earlier time points in those of later ages [comprehensively reviewed in 43] and making it difficult to perform gene prioritization. These methods also disregard the pairwise relationships between gene expression profiles, which may underlie commonly altered pathways and regulatory mechanisms with age. To clarify these issues, we have combined segmented regression analysis with weighted gene correlation network analysis (WGCNA) to re-analyze a publicly available mouse RNA sequencing (RNA-Seq) dataset (GSE132040) [6, 44].

The GSE132040 dataset was made publicly available by the Tabula Muris Consortium [45] and consists of transcriptomic data from 17 male and female mouse tissues sampled across 10 different time points, making it a very useful resource for the study of mammalian aging. In the article resulting from the analysis of this dataset [6], Schaum and colleagues performed differential gene expression, both relative to a reference (1, 3 or 6 months) or between consecutive time points and, acknowledging the limitations of pairwise comparisons, also grouped genes into different clusters based on Euclidean distance and fitted local regression (LOESS) models for each gene in order to address the expression dynamics across the lifespan. We believe this approach does not fully account for the limitations we presented above, and since our goal is to identify gene expression signatures and shifts in expression throughout aging, we decided to re-analyze this data using the R packages Trendy [46] and WGCNA [47].

Trendy is an R package that fits a set of segmented (piece-wise) regression models to high-throughput, ordered expression data, allowing to identify dynamic gene expression patterns over a time course and to determine the direction and point in time when changes in expression occur [46]. It is a recently developed methodology that has been so far applied in stem cell differentiation [48] and in hepatic disease progression [49] studies. Interestingly, the latter research also integrated Trendy with WGCNA to identify high-confidence genes involved in human autoimmune hepatitis (AIH) [49]. Conversely, WGCNA is a widely implemented systems biology approach not only used for constructing co-expression networks based on the pairwise correlations between genes, but also for detecting significant associations between these networks’ modules and a given trait of interest, and to identify important players in these associations [47]. It has been broadly applied in the context of aging research to identify novel age-related gene expression signatures, as is the case of *CR2*, *VPREB3*, *MS4A1* and *CCR6*, involved in B cell activation and receptor signaling pathways in human peripheral blood cells [50], *PPP3CB*, *CAMSAP1*, *ACTR3B*, and *GNG3*, involved in synaptic vesicle cycle, cGMP-PKG, and dopaminergic synapse signaling pathways in human pre-frontal cortex [51], and *PGLS*, a gene potentially involved in synaptic loss in the aging brain of rhesus macaques [52], among other examples.

For all these reasons, we subjected the Tabula Muris Consortium’s GSE132040 dataset to a new analysis pipeline comprising the Trendy and WGCNA approaches in order to: 1) identify expression signatures of genes significantly correlated with age (and with sex) in different mouse tissues, 2) establish their trajectories from mature adulthood to old age, 3) identify the time point of the shift in the gene expression profile, and 4) evaluate the biological processes (BPs) associated with the gene dysregulations. Different onsets of gene dysregulation were identified, demonstrating the asynchronous impairment of gene expression with age described by Schaum et al*.* [6]. Gene Ontology (GO) biological processes’ over-representation analysis revealed that inflammation and immunity-related responses are prominently altered in the brain and the liver, while in the heart and the muscle aging affects fatty acid metabolism, and tissue regeneration, respectively, which may reflect the global loss of organ function, confirming previous reports. Additionally, functional enrichment analysis showed sex-differences in the expression throughout the lifespan of genes involved in energy metabolism and proteostasis-related pathways, with only males exhibiting age-related dysregulation of these processes. Despite little overlap in age-dysregulated genes between tissues, a comparison at the level of dysregulated processes revealed inter-tissue commonalities, such as alterations in immune response, tissue regeneration, energy metabolism, glucocorticoid signaling and response to amino acid stimulus. We propose that the genes involved in these processes are important players of murine physiological aging.

## RESULTS

### Most dynamic changes in gene expression across the lifespan are observed within mature adulthood and middle life

**Figure 1:**
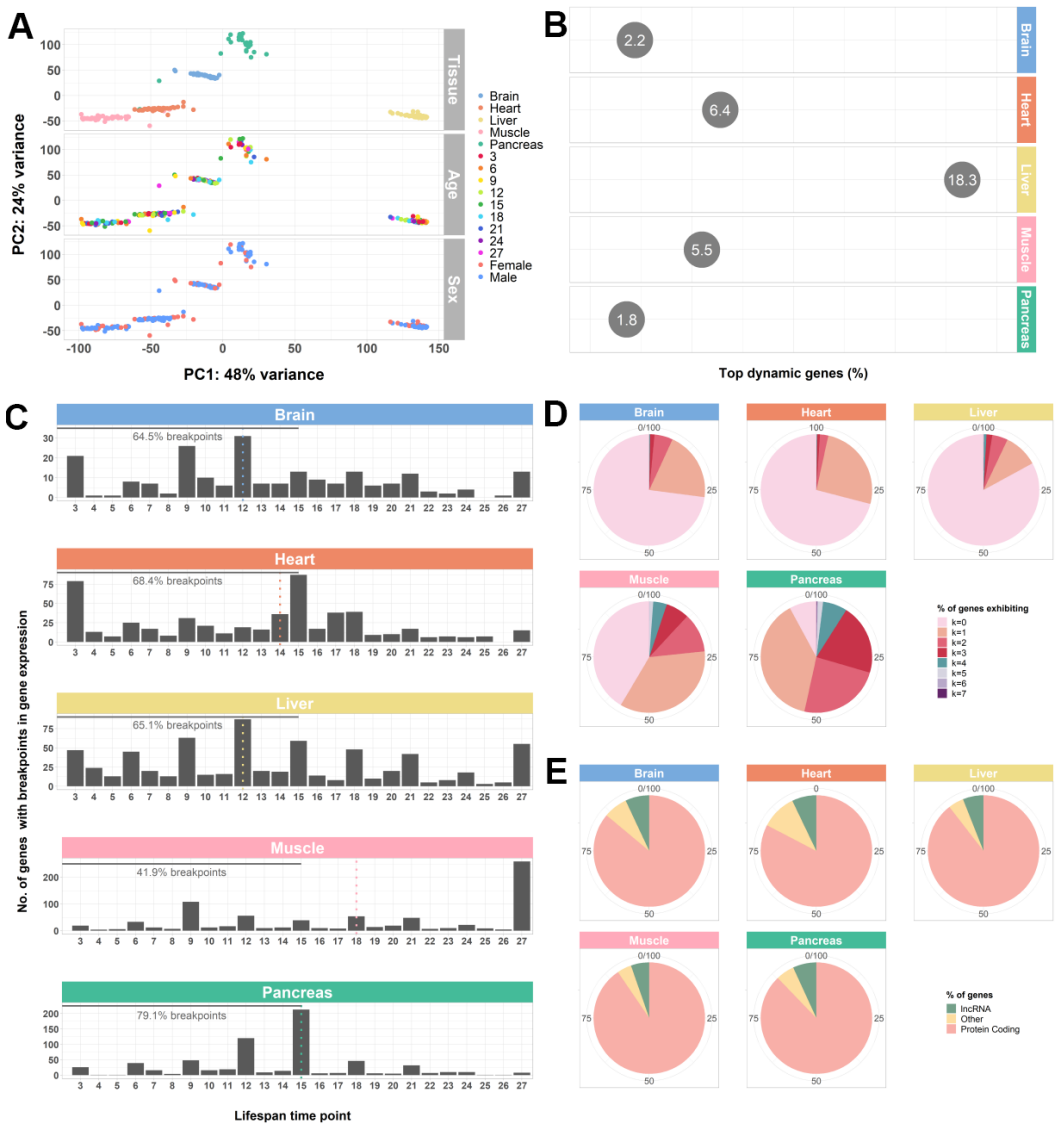
**Whole-transcriptome characterization of different mouse tissues throughout the lifespan by segmented regression analysis.** (**A**) PCA of all tissues performed on VST-normalized read counts of the 500 most variable genes and colored by all known effects highlights type of tissue as the main contributor to sample segregation. (**B**) Percentage of top dynamic (Trendy) genes across the lifespan for the mouse brain, heart, liver, muscle, and pancreas, highlighting the liver and the pancreas the ones with higher and lower dysregulation, respectively. The displayed values correspond to the percentages of the total number of Trendy genes found in each tissue relative to the total number of expressed genes per tissue (brain: 34164, heart: 28073, liver: 20157, muscle: 18978, pancreas: 18414). Top dynamic genes were selected based on tissue-specific adjusted R^2^ thresholds (brain: > 0.2; heart: > 0.2; liver: > 0.1; muscle: > 0.3; pancreas: > 0.3) and *p-values* < 0.1 in at least one segment; Supplemental File SF1). (**C**) Histograms of the distribution of breakpoints in gene expression of the top dynamic genes per tissue. Each bar depicts the sum of all breakpoints of all Trendy genes at that given time point. Monotonic behaviors (i.e., no breakpoints) are not included in the histograms. Dotted, vertical lines indicate the median breakpoint distribution for each tissue. Median breakpoint distribution in the muscle was significantly different from all the other tissues (Kruskal-Wallis Test followed by Dunn’s Test; Supplementary Table 1). (**D**) Percentage of top dynamic genes exhibiting 0 to 7 breakpoints (maximum number of breakpoints allowed in the Trendy regression model). In all tissues, except from the Pancreas, most Trendy genes exhibit monotonic expression patterns (continuously up or down). In the Pancreas, the majority of genes display one breakpoint. (**E**) Biotype distribution of the top dynamic genes per tissue. Biotype nomenclature based on Ensembl annotation. In all tissues, protein coding genes were significantly over-enriched relative to the reference genome’s annotation, whereas lncRNAs were significantly under-enriched (Fisher’s Exact Test; Supplementary Table 2).

In order to obtain a global characterization of the mouse aging transcriptome and, in particular, to understand which of the main known sources of genetic variation (tissue, age, and sex) is responsible for the highest percentage of sample segregation, we performed a principal component analysis (PCA) of brain, heart, liver, muscle and pancreas samples, ranging from 3- to 27-month -old male and female mice, based on variance stabilizing transformation (VST)-normalized read counts. Principal components were calculated based on the 500 most variable genes as they are expected to capture the greatest variability between samples (see Methods - Data pre-processing and normalization. We observed that, based on VST-normalized gene expression values, samples tend to cluster by tissue, which is in line with what Schaum et al*.* [6] found, as well as with previous observations [5, 7, 15, 20–24]. For this reason, subsequent analyses were performed on each tissue separately (Figure 1A). Additionally, we focused only on samples from the brain, heart, muscle, liver, and pancreas, in order to provide an in-depth discussion of the findings and also for future integration with in-house proteomics data. When considering gene expression variation in each tissue independently, we observed that sex, rather than age, is responsible for most of the between-sample variability and adjusted for its effect on gene expression by adding it as a co-variable in the regression model (Supplementary Figure 1; see Methods – Trendy segmented regression analysis.

As a first approach to establish age-regulated genes, we carried out segmented regression analysis on each tissue’s expressed genes (brain: 34164, heart: 28073, liver: 20157, muscle: 18978, pancreas: 18414; post-filtering, normalized expression; see Methods – Data pre-processing and normalization), using the R package Trendy (v. 1.8.2) [46]. In brief, in a segmented regression model, each gene’s expression is regarded as a linear piece-wise function over time, with each segment being separated by a breakpoint (i.e., a point when gene expression changes). The Trendy model fits to each gene multiple segmented regression models with varying number of breakpoints, and then selects the one who explains the best the dynamics of that given gene’s expression across the time-course (see Methods – Trendy segmented regression analysis). Then, we selected the top dynamic (Trendy) genes based on the goodness of fit of the chosen model to the corresponding gene expression pattern, and on the significance of the segments (see Methods – Trendy segmented regression analysis; Supplementary Table 10).

Next, to obtain a global view of the amplitude of gene expression dysregulation of each tissue with aging, we calculated the percentage of Trendy genes across the lifespan, considering each tissue’s total number of top dynamic genes relative to each tissue’s total expressed genes. We identified a total of 747, 1799, 3690, 1048, and 336 dynamic genes in the brain (2.2%), heart (6.4%), liver (18.3%), muscle (5.5%), and pancreas (1.8%), respectively (Figure 1B). These results highlight the pancreas and the liver with the lowest and the highest number of genes changing their expression with age, respectively. When considering the distribution of the breakpoints of each tissue’s top dynamic genes, we found that, for all tissues except for the muscle, the majority of the changes in gene expression throughout the lifespan occur between mature adulthood and middle age. Until 15 months (approximately the end of middle-age [34], we observed 64.5% of breakpoints in the brain, 68.4% in the heart, 65.1% in the liver, 41.9% in the muscle, and 79.1% in the pancreas (Figure 1C). Moreover, the median breakpoint times for the brain (12), heart (14), liver (12) and pancreas (15) were not significantly different between these tissues but differ significantly from the median breakpoint time of the muscle (18) (Figure 1C; Supplementary Table 1). Nevertheless, with the exception of the pancreas, the majority of Trendy genes exhibit monotonic expression behaviors (no breakpoints, k=0; Figure 1D), either continuously increasing or decreasing expression throughout time. Biotype assessment of the top dynamic genes in each tissue based on the Ensembl classification (see Methods – Data pre-processing and normalization) showed that dysregulation mainly occurs at the protein coding level (Figure 1E; Supplementary Table 2). The observed under-enrichment of long non-coding RNAs (lncRNAs) in each tissue when compared with the reference genome, might be an artifact resulting from the filtering process as these RNA genes tend to exhibit lower expression levels (Supplementary Table 2). Nonetheless, changes in the expression of long non-coding RNAs may be of relevance, especially in the brain, liver, muscle, and pancreas (Figure 1E).

### Different subsets of co-expressed genes exhibit specific age- and sex-related trajectories

As a second approach to defining age-regulated genes, we performed weighted gene correlation network analysis (WGCNA) to explore age- and sex-associated co-expression patterns of gene expression. Similar to the regression analysis, WGCNA was performed on each tissue independently, with each tissue’s co-expression network comprising a variable number of modules of positively correlated genes (Supplementary Table 3).

One of the great advantages of WGCNA is that this methodology takes into consideration the inter-dependency of gene expression. Rather than analyzing each gene independently, WGCNA groups together correlated genes into modules, taking advantage of a power transformation on the pairwise gene correlations to accentuate strong correlations and play down weak ones. The resulting co-expression modules can then be associated with traits of interest (such as age or sex) and the strength of the association can be used to select modules of interest. WGCNA is also very useful for gene prioritization as it allows to rank genes within each cluster and identify intramodular hub genes, likely to be important for the phenotype [47].

**Figure 2:**
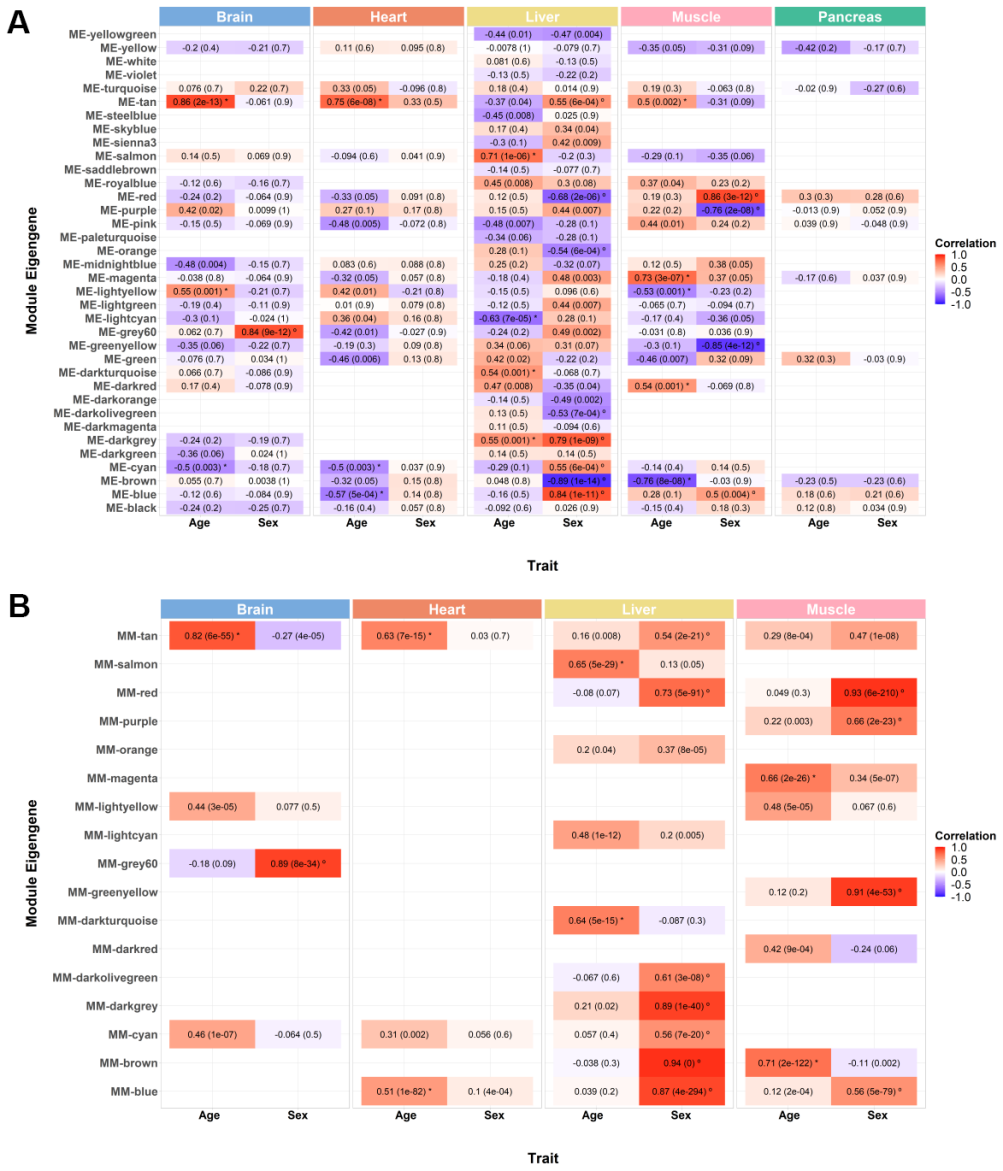
**Weighted gene co-expression network module selection.** (**A**) Correlation between each module’s eigengene (ME) with age and sex. Each tissue exhibits a variable number of modules of co-expressed genes (brain: 24; heart: 19; liver: 36; muscle: 21; pancreas: 10), and unassigned genes are clustered together in the grey module (not shown). ME is the first principal component of the expression matrix of a module, thus being the most representative gene expression profile of that group of correlated genes. Cells are annotated with bicor values and corresponding FDR adjusted *p-values* (inside brackets). Red and blue cells depict positive and negative correlations, respectively. The intensity of color represents the degree of correlation. All modules whose ME’s correlation with the trait of interest is significantly equal or higher than 0.5 were considered (moderate correlation and above; FDR < 0.05; significant correlations with age marked with * and significant correlations with sex marked with º). (**B**) Correlation between module membership (MM) and gene significance (GS) of the previously selected significant modules. MM is obtained by correlating the expression of individual genes to the ME, and GS corresponds to the absolute value of the correlation between individual genes and the trait of interest. Only modules with moderate or higher (≥ 0.5) and significant (*p-value* < 0.05) correlations with age and sex were considered for subsequent analysis (marked with * and º, respectively).

To select the most interesting modules for the aging process, as well as sex-dimorphic genes, we followed a two-step approach. First, for each module, the corresponding gene expression profiles were summarized into a representative - module eigengene (ME; see Methods - Identification of significantly age- and sex- associated modules, hub genes, and Trendy-module-hub overlapping genes) - and this illustrative profile was correlated with age and sex. All modules with a significant (false discovery rate (FDR) < 0.05) and at least moderate (≥ 0.5) correlations between their ME and age were selected. The brain and the heart both exhibited 3 modules significantly associated with age (2 positive and 1 negative in the brain; 1 positive and 2 negative in the heart); the liver displayed 4 age-associated modules (3 positive and 1 negative); the muscle exhibited 5 modules correlated with age (3 positive and 2 negative); and the pancreas did not have any modules significantly correlated with age (Figure 2A). Regarding association with sex, the brain exhibited only 1 positively sex-correlated module; the muscle showed 4 sex-associated modules (2 positive and 2 negative) while the heart and the pancreas exhibited none (Figure 2A). In line with what we previously observed, the liver appears to be the analyzed tissue whose gene expression is the most influenced by sex, as it displays 8 modules significantly sex-associated (4 positive and 4 negative) (Figure 2A; Supplementary Figure 1). Because the pancreas did not exhibit any age- or sex- associated modules, it was not considered for further analyses (Figure 2A). Noteworthily, none of the age-associated modules overlapped with sex-associated ones, with the exception of the Darkgrey module in the liver (Figure 2A). Because sex was treated as a binary variable, with 0 encoding females and 1 encoding males, all positive associations in sex-associated modules correspond to over-representation in males, while all negative-associations correspond to over-representation in females.

Next, the gene significance (GS) and module membership (MM) measures were analyzed. GS refers to the absolute value of individual correlations of genes to the trait of interest, whereas MM relates to the individual correlations of genes to the ME. High correlations between these two measures are indicative of genes that are highly significant for the aging process and for sex differences in gene expression, being as well highly important to the module. Modules exhibiting significant (*p-value* < 0.05) and at least moderate MM-GS correlations (≥ 0.5) were selected. From the 3 previously selected age-associated modules in the brain, only 1 exhibited significant MM-GS correlations Tan module), and the single selected sex-associated module also displayed a significant MM-GS correlation (Grey60 module) (Figure 2B). In the heart, 2 out of 3 age-associated modules displayed significant MM-GS correlations (*Tan* and *Blue* modules; Figure 2B). As for the liver, from the 4 previously selected age-related modules, 2 exhibited significant MM-GS correlations Salmon and Darkturquoise modules), while from the 8 selected sex-associated modules, 7 also passed the MM-GS correlation criteria Tan, Red, Darkolivegreen, Darkgrey, Cyan, Brown and Blue modules) (Figure 2B). Interestingly, the genes in the Darkgrey module did not seem to be highly associated with aging as the MM-GS correlation is rather small (0.21; Figure 2B). For this reason, we will consider the Darkgrey module as only a sex-associated module for further analyses. Finally, in the muscle, 2 out 5 age-related modules were selected based on MM-GS correlations (Magenta and Brown modules), and all 4 sex-associated modules also exhibited significant MM-GS correlations (Red, Purple, Greenyellow and Blue modules) (Figure 2B). These selected clusters of genes can be considered the most relevant to the aging process and for sex-dimorphic gene expression.

**Figure 3:**
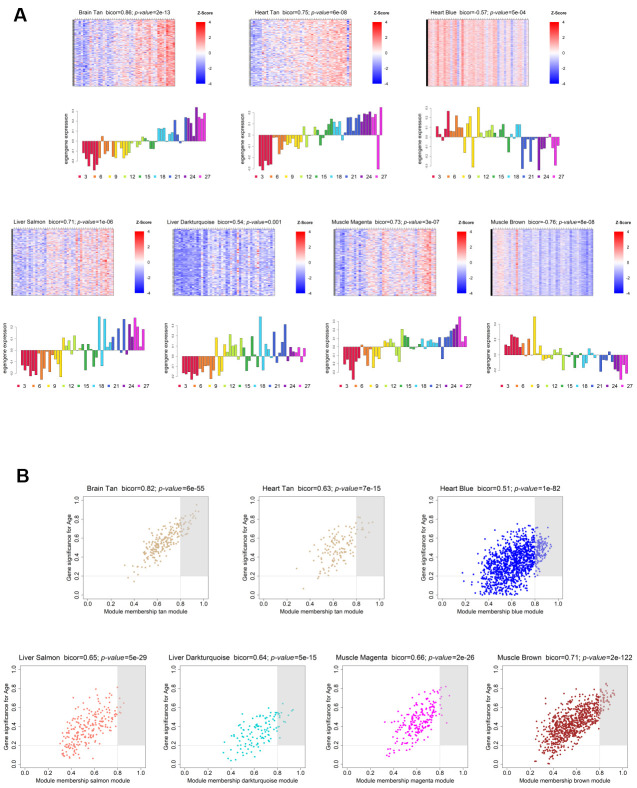
**Weighted gene co-expression network significantly age-associated modules.** (**A**) Gene expression profile of each significantly age-associated module. The heatmaps (top) display the standardized expression (*z-score*) of individual genes (rows) per sample (columns), whereas the bar plots (below) represent the ME expression profile. Each bar of the bar plot corresponds to the same samples of the heatmap. Negative (positive) values of ME expression relate to the under-expression (over-expression) of genes in each module’s heatmap (blue and red colors, respectively). (**B**) Intramodular hub gene identification. For each module, genes with individual GS > 0.2 and MM > 0.8 were considered to be the most functionally important (inside grey rectangles).

In order to evaluate each module’s age- and sex-related gene expression, we plotted a heatmap of VST-normalized expression values of the genes belonging to the module, accompanied by a bar plot representing the ME expression profile (Figure 3A; Supplementary Figure 2). This visualization allowed us to better understand the behavior of the age- and sex-related genes across time, as well as to identify the lifespan periods where shifts in expression occur. The brain showed an increasing trend in gene expression with age (*Tan* module; bicor=0.86; *p-value*=3e-13), with the shift from down- to up-regulation occurring around the transition from middle- to old-age (15 to 18 months) (Figure 3A, upper left panel). Regarding the genes present in its sex-associated module – Grey60 (bicor=0.84; *p-value*=9e-12), they are over-expressed in males, and no obvious trend of increased/decreased expression throughout time was observed (Supplementary Figure 2, upper left panel). As for the heart, the 2 significant age-related modules display different expression trends. The Blue module (bicor=-0.57; *p-value*=5e-04) exhibits a decreasing trend in gene expression with age, and the transition from up- to down-regulation happened at old-age (18 to 21 months) (Figure 3A, upper left panel). Conversely, the Tan module presents an increasing trend in expression throughout the lifespan, transitioning from down- to up-regulation within middle-age (12 to 15 months) (Figure 3A, upper central panel). Concerning the liver, both age-associated modules displayed an increasing trend in gene expression over time, the difference being the onset of expression change. In the Salmon module (bicor=0.71; *p-value*=1e-06), gene expression starts to increase within middle-age (9 to 12 months), whereas in the *Darkturquoise* (bicor=0.54; *p-value*=0.001) this shift is less defined, probably occurring around the transition from mature adulthood to middle-age (6 to 9 months) (Figure 3A, lower left panels). In the case of modules significantly correlated with sex in the liver, the Brown (bicor=-0.89; *p-value*=1e-14), the Darkolivegreen (bicor=-0.53; *p-value*=7e-04) and the Red (bicor=-0.68; *p-value*=2e-06) modules are female-associated, and none displayed any obvious trends in gene expression changes over time (Supplementary Figure 2, upper right and central panels). As for the male-associated modules in the liver, all exhibit slightly decreasing trends in the expression of their genes, with all shift points in expression occurring within old age. Both the Blue (bicor=0.84; *p-value*=1e-11) module and the Darkgrey (bicor=0.79; *p-value*=1e-09) modules exhibited a shift between 24 and 27 months (within old age) (Supplementary Figure 2, upper right and central panels). Moreover, the Cyan module (bicor=0.55; *p-value*=6e-04) displayed its shift in gene expression around the transition from 18 to 21 months (Supplementary Figure 2, upper right and central panels), whereas the Tan module’s (bicor=0.55; *p-value*=6e-04) expression change occurred between 21 and 24 months (Supplementary Figure 2, upper right and central panels). Concerning the muscle, we observed opposite trends in the two age-associated modules. In the Magenta module (bicor=0.73; *p-value*=3e-07) we observed increased gene expression across the lifespan, whereas in the Brown module (bicor=-0.76; *p-value*=8e-08), the expression tends to decrease over time (Figure 3A, lower right panels). Interestingly, in both modules the shift in expression occurs within middle-age, around the transition from 9 to 12 months (Figure 3A, lower right panels). Finally, in regards to the sex-associated modules in the muscle, only the Blue module (bicor=0.5; *p-value*=0.004), which is male-related, displayed a discernible trend of increased expression across the lifespan, with the transition from down- to up-regulation taking place within middle-age (9 to 12 months) (Supplementary Figure 2, lower left panel). The other 3 sex-related modules in this tissue did not exhibit any obvious trends in gene expression over time, with the Red module (bicor=0.86; *p-value*=3e-12) being male-associated, and the Greenyellow (bicor=0.85; *p-value*=4e-12) and Purple (bicor=0.76; *p-value*=2e-08) modules being female-associated (Supplementary Figure 2, lower right panels).

Furthermore, in each module, we identified the genes with the highest MM and GS – Hub genes - as they are important elements of the module, as well as the most significantly associated with the trait of interest (see Methods - Identification of significantly age- and sex-associated modules, hub genes, and Trendy-module-hub overlapping genes; Figure 3B and Supplementary Figure 3; and Supplementary Table 4).

### Altered genes and biological networks provide tissue-specific markers of aging

**Figure 4:**
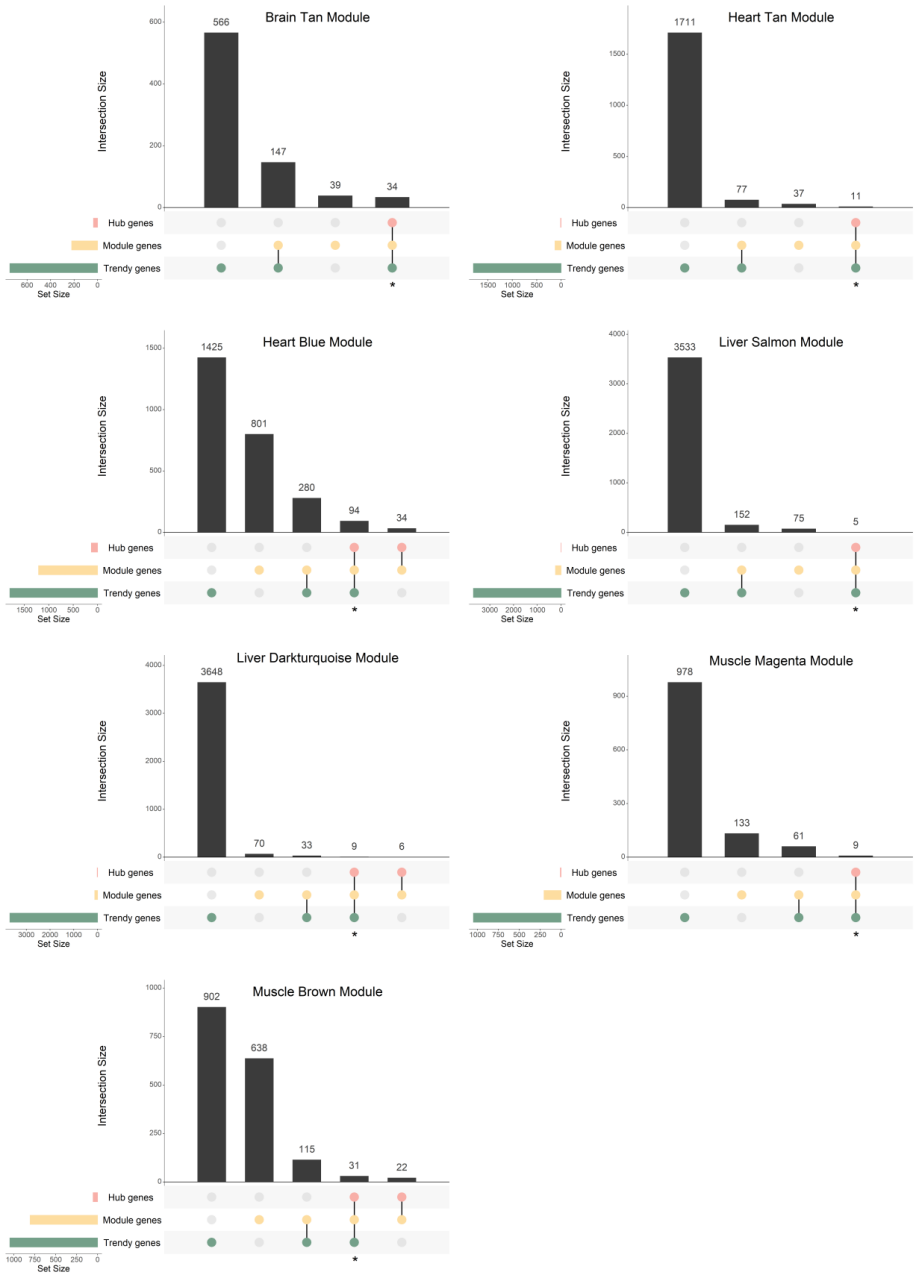
**Gene overlap between Trendy genes, module genes, and hub genes in the age-associated modules with significantly enriched GO terms.** Bars represent intersection size and colored circles depict the gene sets involved. Genes in common in the Trendy and hub gene sets were considered for further analysis (identified with *).

In order to integrate the results from the two described approaches for finding age-dysregulated genes, we intersected the resulting gene lists and evaluated their functional implications. For each tissue, we compared the previously identified Trendy top dynamic genes with the gene sets of interest resulting from WGCNA (Module and Hub genes) and selected for functional analysis the intersection of the Trendy and the Hub genes (Figure 4; Supplementary Figure 4; Supplementary Table 5). As expected, all Hub genes overlapped with the Module genes. Because our interest is to explore gene expression dysregulation across the lifespan, we performed this analysis on all age-associated modules and on only the sex-associated modules exhibiting discernible trends of increased/decreased gene expression over time (i.e., the Blue, Cyan, Darkgrey, and Tan modules in the liver, and the Blue module in the muscle).

In the brain Tan module, 34 genes were selected for further analysis (Figure 4; Supplementary Table 5). As for the heart, the size of the overlap between Trendy and Hub genes was 11 and 94, for the Tan and Blue modules, respectively (Figure 4; Supplementary Table 5). In the liver, regarding age-associated, 5 and 9 intersecting genes were selected from the Salmon and Darkturquoise modules, respectively (Figure 4; Supplementary Table 5), whereas 92, 12, 20 and 13 genes were selected from the Blue, Cyan, Darkgrey, and *Tan* sex-associated modules, respectively (Supplementary Figure 4; Supplementary Table 5). Finally, in the muscle we proceeded the analysis with 9, 31, and 21 genes from the *Magenta*, Brown and Blue modules, respectively (Figure 4 and Supplementary Figure 4; Supplementary Table 5).

To better understand the functions underlying these signature gene lists, we performed an enrichment analysis on GO BPs and selected the ones with an FDR adjusted p-value of less than 0.05 (see Methods - Functional characterization of Trendy-module-hub genes’ overlap; Supplementary Table 6). To deal with the large number of significant BPs exhibited by some modules and to provide a clear picture of how they and their associated genes relate to each other, we constructed a network of these results, with nodes representing GO terms and edges corresponding to the overlap of genes between any two nodes (see Methods - Network visualization of functionally enriched terms). After having constructed the network, we addressed GO term redundancy by clustering together nodes based on gene overlap similarity and then assigning automatically created labels from the most frequent words in the cluster, as well as words adjacent to the most represented ones (see Methods - Network visualization of functionally enriched terms; Supplementary Table 6) [as seen in 53, and in 54]. To further simplify the visualization, we created a summary network based on the generated clusters, where all the nodes belonging to the same cluster collapsed into a meta-node and all the edges connecting the different clusters collapsed into *meta-edges* as well [55] (Supplementary Table 6). Selected results are present in Table 1.

**Table 1:** Tissue-specific age-dysregulated functions.

**Tissue**	**Module**	**Summary network meta-node**	**Summary network FDR**	**No. of nodes**	**Meta-node choice criteria**	**Genes involved**	**WGCNA expression trend**	**WGCNA expression shift**
Brain	Tan	mediated immunoglobulin regulation response	8.06E-03	142	Largest	*B2m, C1qa, C1qb, C1qc, C3, C4b, Csf1, Ctsh, Ctss, Ctsz, Cx3cr1, Gfap, H2-D1, H2-K1, H2-T23, Hexb, Il33, Irf7, Itgb2, Lag3, Lgals3, Neat1, Psmb8, Serpina3n, Slc11a1, Tap2, Tapbp*	Increasing with age	Middle to old-age - 15-18 months
Brain	Tan	neuron death activation microglial	6.59E-03	7	Most significant	*C1qa, Csf1, Ctsz, Cx3cr1, Il33, Slc11a1*	Increasing with age	Middle to old-age - 15-18 months
Heart	Blue	branched-chain amino process metabolic	1.57E-02	17	Largest	*Acaa2, Adk, Auh, Ckm, Ckmt2, Cyc1, Eno3, Gnpat, Hmgcl, Isca1, Mccc2, Nfs1, Pdhb, Suclg1*	Decreasing with age	Old-age - 18-21 months
Heart	Blue	acid catabolic lipid fatty	4.19E-04	12	Most significant	*Acaa1a, Acaa2, Adipor1, Auh, Eci2, Etfb, Gnpat, Hadh, Hmgcl, Mccc2, Pgm2, Phyh, Ptges2, Smpd1*	Decreasing with age	Old-age - 18-21 months
Liver	Darkturquoise	response immune regulation cell	7.07E-03	57	Both	*Cd19, Cd79a, Cd79b, Ighm, Igkc, Igkv3-5, Iglc2, Jchain, Mzb1*	Increasing with age	Adulthood to middle-age - 6-9 months
Liver	Salmon	regulation assembly cell positive	3.32E-02	184	Both	*Ccl5, H2-Aa, H2-Eb1, Ntrk2, Slamf7*	Increasing with age	Middle-age - 9-12 months
Liver	Blue	lipid biosynthetic process	3.43E-02	1	Both	*Cdipt, Elovl2, Elovl3, Hsd17b12, Mecr, Pip5k1a, Pisd, Rest, Scp2, Serinc1, Smpd1*	Decreasing with age (males)	Old-age - 24-27 months
Liver	Cyan	endoplasmic reticulum golgi vesicle-mediated	2.80E-03	4	Most significant	*Arcn1, Copg1, Sec22b, Sec24d*	Decreasing with age (males)	Old-age - 18-21 months
Liver	Cyan	protein transmembrane response development	1.41E-02	21	Largest	*Arcn1, Copg1, Hspa5, Sdf2l1, Sec22b, Sec24d, Sec61a1, Serp1*	Decreasing with age (males)	Old-age - 18-21 months
Muscle	Brown	regulation morphogenesis negative development	1.12E-02	82	Both	*Angptl1, Anxa2, Cd34, Col3a1, Col5a1, Col5a2, Col6a1, Col6a2, Col6a3, Dok2, Fn1, Igfbp6, Itgbl1, Lrp1, Ndn, Nid1, Pi16, Serpinf1, Serping1, Sparc, Ssc5d, Tgfbi, Timp2*	Decreasing with age	Middle-age - 9-12 months
Muscle	Blue	proteasomal ubiquitin-independent protein catabolic	1.78E-02	1	Most significant	*Psmb1, Psmb3*	Increasing with age (males)	Middle-age - 9-12 months
Muscle	Blue	ribonucleoside triphosphate metabolic process	2.89E-02	13	Largest	*Atp5l, Atp5g3, Cox5a, Ndufa8*	Increasing with age (males)	Middle-age - 9-12 months

The choice of the representative meta-nodes was based on the number of nodes (GO terms) included (Largest) and/or on the *p-values* (Most significant). In the sex-associated modules, the sex in which the genes are enriched is indicated inside parentheses. Relates to Supplementary Table 6.

### Neuronal death and immune response processes are upregulated in the aging brain

GO enrichment analysis of the overlap of the brain Trendy genes with the Tan module hub genes identified 233 significantly over-represented BPs, allocated into 9 meta-nodes (Supplementary Table 6). The largest meta-node is involved in antigen-mediated immunity and comprises 142 GO terms, thus representing more than half of all significant processes observed for this set (Brain Tan module: Table 1; Supplementary Table 6). The genes present in this group exhibit increased expression with increasing age, shifting from down- to up-regulation around the transition from middle to old age (15-18 months; Brain Tan module: Figure 3A; Table 1).

Furthermore, 7 processes related to neuronal death were found to be the most significantly enriched in the aging brain (Brain Tan module: Table 1; Supplementary Table 6). This meta-node also includes genes whose expression tend to increase throughout aging with the shift from down- to up-regulation occurring from middle- to old-age (15-18 months; Brain Tan module: Figure 3A; Table 1).

### Decline of cardiac ribonucleotide and fatty acid metabolism with age

In the heart, we identified 11 clusters of similar GO terms, comprising 79 significantly enriched GO BPs based on the intersection between the heart top dynamic genes and the heart Blue module hub genes (Heart Blue module: Table 1; Supplementary Table 6). The most significant meta->node includes processes related to fatty acid metabolism, whereas the largest cluster comprises BPs related to ribonucleotide metabolism (Heart Blue module: Table 1; Supplementary Table 6). The genes found to be involved in these processes exhibit decreased expression along the lifespan, with the transition from up- to down-regulation occurring late in life (18-21 months; Heart Blue module: Figure 3A; Table 1).

### Muscle regeneration is impaired with aging and mitochondrial energy metabolism and proteasomal activity are altered in the muscle of aged male mice

Over-representation analysis of GO terms in the gene set obtained from the overlap of the muscle Trendy genes and the muscle Brown module hub genes resulted in a highly interconnected network of 82 BPs organized into a single meta-node (Supplementary Table 6). This cluster includes genes related to muscle tissue regeneration (Muscle Brown module: Table 1; Supplementary Table 6), which are down-regulated across the lifespan. A shift from up- to down-regulation occurs in middle age (9-12 months; Muscle Brown module: Figure 3A; Table 1).

Additionally, the muscle exhibits one gene module whose expression is male-associated where we could observe an increasing trend in expression over time (Muscle Blue module: Supplementary Figure 2; Table 1). After performing GO over-representation analysis on the overlap of muscle dynamic genes with the muscle Blue module hub genes we identified 28 processes allocated into 4 meta-nodes (Muscle Blue module: Supplementary Table 6). The largest cluster is related to the metabolism of adenosine triphosphate (ATP), while the most significant one is associated with proteasomal activity (Muscle Blue module: Table 1; Supplementary Table 6). The genes involved in these processes exhibit increased expression in aged males, with the transition from under- to over-expression taking place around middle-age (9-12 months; Muscle Blue module: Supplementary Figure 2; Table 1).

### Liver aging is characterized by a global dysregulation of immune function and sex-specific differences in lipid metabolism and stress response activity

In the liver, a total of 247 BPs were identified as being significantly enriched among the gene lists resulting from the overlap of the liver top dynamic genes and the age-associated modules hub genes (Liver Darkturquoise and Salmon modules: Table 1; Supplementary Table 6). In the Trendy- Darkturquoise module gene set, 63 enriched GO terms were allocated into 2 meta-nodes, with the largest and most significant (n = 57) relating to immune cell activation, differentiation and proliferation (Liver Darkturquoise module: Table 1; Supplementary Table 6). The genes involved in these processes exhibit increased expression throughout the lifespan, with the shift from down- to up-regulation occurring between adulthood to middle age (6-9 months; Liver Darkturquoise module: Figure 3A; Table 1). Regarding the Trendy-Salmon module hub gene overlap, 1 cluster of processes also related to immune response was identified, comprising 184 GO terms (Liver Salmon module: Table 1; Supplementary Table 6). The identified genes within this cluster present an increased trend in expression with increasing age, shifting from down- to up-regulation within middle age (9-12 months; Liver Salmon module: Figure 3A; Table 1).

Similar to what we have observed in the muscle, the liver also exhibits sex-dimorphism in gene expression with aging. In the liver Blue module, GO over-representation analysis on the overlap of top dynamic genes with hub genes allowed for the identification of 1 BP related to biosynthesis of lipids, whose comprising genes exhibit decreased expression over time, shifting from up- to down-regulation really late in life, around the transition from 24 to 27-months (Liver Blue module: Supplementary Figure 2; Table 1; Supplementary Table 6). Moreover, in the Cyan module, 25 identified processes were allocated into 2 meta-nodes, the most significant one related to the endoplasmic reticulum (ER) transport system, and the largest one associated with ER stress response (Liver Cyan module: Table 1; Supplementary Table 6). The genes involved in these processes exhibit decreased expression in aged males, with the transition from over- to under-expression taking place within old-age (18-21 months; Liver Cyan module: Supplementary Figure 2; Table 1).

### Similar biological processes altered across multiple tissues

**Figure 5:**
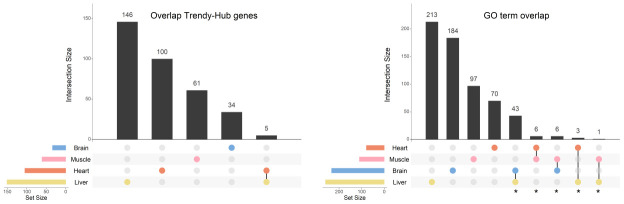
**Overlap of genes and biological processes between the brain, heart, liver, and muscle.** Upset plots depicting the gene overlap between Trendy genes, module genes, and hub genes per tissue (left), as well as the overlap of the enriched GO terms in the same tissues (right). Bars represent intersection size and colored circles depict the gene/GO term sets involved. Each tissue's gene list results from the intersection of Trendy, module, and hub genes. For this plot we considered all significantly age-associated modules and the significantly-sex associated modules with an observable increase/decrease in gene expression over time (i.e., liver tan, blue, cyan and Darkgrey modules, and muscle blue module). In tissues with more than one significant module (i.e. the heart, the muscle and the liver), the gene list results from the combination of each module's intersection, and the GO term list results from the combination of each module's GO terms. GO terms in common at least in two tissues were considered for further analysis (identified with *).

Evidence from other studies have showed that differences in gene expression across the lifespan are strongly associated with tissue type. For that reason, all the analyses were performed in each tissue independently. Our data confirmed this trend since few players that are associated with aging are shared between tissues (5 genes in common between only the heart and the liver) (Figure 5 - left panel; Supplementary Table 7). Nonetheless, when we compared the GO terms enriched between tissues, we found a much higher overlap than that observed at the gene level (Figure 5 - right panel; Supplementary Table 8). The highest number of shared BPs (43) was observed between the brain and the liver, followed by 6 processes shared between the heart and the muscle. The brain and the muscle also exhibited 6 GO terms in common, while the heart and the liver shared 3 BPs. Finally, the muscle and the liver, presented only 1 BP in common (Figure 5 – right panel; Supplementary Table 8). As described above, we organized the GO terms in summary networks (Supplementary Table 8) and summarized the results in Table 2.

**Table 2:** Inter-tissue age-dysregulated functions.

**Brain-Liver**								
**Related function**	**Summary network description**	**No. of nodes**	**Genes involved - Brain**	**Genes involved - Liver**	**WGCNA expression trend - Brain**	**WGCNA expression trend - Liver**	**WGCNA expression shift - Brain**	**WGCNA expression shift - Liver**
Immune response	Response immune mediated immunoglobulin	13	*B2m, C1qa, C1qb, C1qc, C3, C4b, Ctsh, Gbp3, H2-D1, H2-K1, H2-T23, Il33, Irf7, Itgb2, Lag3, Lgals3, Lyz2, Slc11a1, Tap2*	*Cd19, Cd79a, Cd79b, H2-Aa, H2-Eb1, Ighm, Igkc, Iglc2, Jchain, Slamf7*	Increase	Increase	Middle to old-age - 15-18 months	Adulthood to Middle-age - 6-9 months and 9-12 months
	regulation production cell proliferation	26	*B2m, C1qa, C1qc, C3, Csf1, Ctsz, Cx3cr1, H2-D1, H2-K1, H2-T23, Il33, Itgb2, Lag3, Lgals3, Slc11a1, Tap2*	*Ccl5, Cd19, Cd79a, H2-Aa, Ighm, Igkv3-5, Mzb1, Ntrk2, Slamf7*	Increase	Increase	Middle to old-age - 15-18 months	Middle-age - 9-12 months
	processing presentation peptide antigen	4	*B2m, Ctss, H2-D1, H2-K1, H2-T23, Psmb8, Slc11a1, Tap2, Tapbp*	*H2-Eb1, H2-Aa*	Increase	Increase	Middle to old-age - 15-18 months	Middle-age - 9-12 months
**Brain-Muscle**								
**Related function**	**Summary network description**	**No. of nodes**	**Genes involved - Brain**	**Genes involved - Muscle**	**WGCNA expression trend - Brain**	**WGCNA expression trend - Muscle**	**WGCNA expression shift - Brain**	**WGCNA expression shift - Muscle**
Tissue regeneration	angiogenesis	1	*C3, Lgals3, Itgb2, Ctsh, Cx3cr1*	*Fn1, Serpinf1, Sparc, Cd34, Anxa2*	Increase	Decrease	Middle to old-age - 15-18 months	Middle-age - 9-12 months
	glial cell migration	1	*Csf1, Hexb*	*Fn1, Ndn, Lrp1*	Increase	Decrease	Middle to old-age - 15-18 months	Middle-age - 9-12 months
	extracellular structure organization response	3	*C3, Ctss, Gfap, Lgals3, Neat1, Slc11a1*	*Anxa2, Cd34, Col3a1, Col5a1, Col5a2, Fn1, Lrp1, Nid1, Serping1, Tgfbi*	Increase	Decrease	Middle to old-age - 15-18 months	Middle-age - 9-12 months
	regulation peptidase activity	1	*Serpina3n, Ctsh, Psmb8, Ctsd*	*Serpinf1, Pi16, Serping1, Timp2*	Increase	Decrease	Middle to old-age - 15-18 months	Middle-age - 9-12 months
**Heart-Muscle**								
**Related function**	**Summary network description**	**No. of nodes**	**Genes involved - Heart**	**Genes involved - Muscle**	**WGCNA expression trend – Heart**	**WGCNA expression trend - Muscle**	**WGCNA expression shift - Heart**	**WGCNA expression shift - Muscle**
Energy metabolism	respiratory electron transport atp	6	*Coq9, Cyc1, Eno3, Ndufa10, Ndufs2, Sdhd, Slc25a12, Uqcrc1*	*Atp5l, Atp5g3, Cox5a, Ndufa8*	Decrease	Increase (males)	Old-age - 18-21 months	Middle-age - 9-12 months
**Liver-Heart**								
**Related function**	**Summary network description**	**No. of nodes**	**Genes involved - Liver**	**Genes involved - Heart**	**WGCNA expression trend – Liver**	**WGCNA expression trend - Heart**	**WGCNA expression shift - Liver**	**WGCNA expression shift - Heart**
Glucocorticoid signaling	receptor signaling pathway glucocorticoid	3	*Ntrk2*	*Ppp5c, Phb*	Increase	Decrease	Middle-age - 9-12 months	Old-age - 18-21 months
**Liver-Muscle**								
**Related function**	**Summary network description**	**No. of nodes**	**Genes involved - Liver**	**Genes involved - Muscle**	**WGCNA expression trend – Liver**	**WGCNA expression trend - Muscle**	**WGCNA expression shift - Liver**	**WGCNA expression shift - Muscle**
Response to amino acid stimulus	cellular response to amino acid stimulus	1	*Ntrk2*	*Col3a1, Col5a2, Col6a1*	Increase	Decrease	Middle-age - 9-12 months	Middle-age - 9-12 months

In the sex-associated modules, the sex in which the genes are enriched is indicated inside parentheses. Relates to Supplementary Table 8.

The liver and the brain share 43 GO biological processes clustered into 3 meta-nodes (Brain-Liver; Table 2; Supplementary Table 8). In line with what we observed at the tissue-level, immune processes are represented in the intersection of GO terms between these two tissues. In both tissues, the genes involved in the processes comprising these meta-nodes exhibit an increasing trend of expression across the lifespan (Brain Tan and Liver Salmon and Darkturquoise modules: Figure 3A; Brain-Liver: Table 2; Supplementary Table 8). However, the dysregulation of the genes involved in these processes occurs later in the brain, in the transition from middle to old age (15-18 months), than in the liver (6-9 months and 9-12 months; Brain Tan and Liver Salmon and Darkturquoise modules: Figure 3A; Brain-Liver: Table 2; Supplementary Table 8).

Interestingly, in the brain and the muscle the commonly affected BPs are related to tissue regeneration, with the 6 identified GO terms allocated to 4 clusters (Brain-Muscle: Table 2; Supplementary Table 8). In the brain, the genes comprised by these meta-nodes display increased expression across the lifespan, whereas the corresponding muscle genes displayed a down-regulation trend (Brain Tan and Muscle Brown modules: Figure 3A; Brain-Muscle: Table 2). Moreover, the shifts in regulation occurred late in life in the brain, around the transition from middle to old age, whereas in the muscle they are observed earlier, within middle age (15-18 and 9-12 months, respectively; Brain Tan and Muscle Brown modules: Figure 3A; Brain-Muscle: Table 2).

In the case of the heart and muscle, the 6 age-related dysregulated processes are related to energy metabolism, particularly to the synthesis of ATP (Heart-Muscle: Table 2; Supplementary Table 8). Notably, the overlap of these processes is observed between the hearts of both male and female mice, and the muscle of only male mice. In the heart, the genes related to these processes exhibit a decreasing trend in expression, transitioning from over- to under-expression within old age (18-21 months; Heart Blue module: Figure 3A; Heart-Muscle: Table 2*;* Supplementary Table 8). As for the genes in the muscle they tend to increase their expression in males over time, shifting from down- to up-regulation within middle age (9-12 months; Muscle Blue module: Supplementary Figure 2; Heart-Muscle: Table 2; Supplementary Table 8).

The liver and the heart share 3 biological processes, all related to glucocorticoid signaling (Liver-Heart: Table 2; Supplementary Table 8). Interestingly, the genes enriched in these GO terms display opposite trends in expression throughout life. In the liver, we have observed increased expression with age, with a shift point within middle age (9-12 months; – Liver Salmon module: Figure 3A; Liver-Heart: Table 2; Supplementary Table 8), whereas in the heart, we found gene expression to decrease across the lifespan, transitioning from up- to down-regulation later in life (18-21 months; Heart Blue module: Figure 3A; Liver-Heart: Table 2; Supplementary Table 8).

As for the liver and the muscle, we found only 1 process in common between these tissues involving the response to amino acid stimulus (Liver-Muscle: Table 2; Supplementary Table 8). Despite the genes comprised in this single meta-node changing their expression within middle age (9-12 months) in both tissues, they present opposite expression trends, increasing in the liver and decreasing in the muscle (Liver Salmon and Muscle Brown modules: Figure 3A; Liver-Muscle: Table 2; Supplementary Table 8).

## DISCUSSION

In this work, we provide an in-depth characterization of the age-associated alterations in gene expression throughout the murine lifespan, through the re-analysis of publicly available mouse aging transcriptomic data (GSE132040). We have combined, for each tissue, a segmented regression model fitted to each gene to select the top dynamic genes over time with a network-based approach to establish tissue-specific clusters of co-expressed genes correlated with increasing age and to identify sex-dimorphic gene expression patterns. By integrating the results of these two methodologies, we were able to both prioritize aging genes and establish gene expression signatures of aging with greater confidence. Among the selected tissues, the liver showed the highest percentage of dynamic genes across the lifespan, which is in line with recent works from other laboratories [20] and may be related to its high metabolic activity and protein synthesis rate [56, 57]. Nevertheless, these observations do not consider the magnitude of the dysregulation so conclusions must be drawn carefully as only one aspect of age-related gene expression dysregulation was addressed. We have also observed that most alterations in gene expression occur between 12 and 15 months in all tissues but the muscle, where the majority of changes took place in old age. Interestingly, Schaum and colleagues also observed a late-life gene expression dysregulation in this tissue, with the largest number of differentially expressed genes (DEGs) occurring in the 24 and 27 month age groups [6]. Additionally, the pancreas appeared to be largely unaffected by aging since it displayed the lowest amplitude of dysregulation and no significantly age-associated gene co-expression modules, also corroborating the findings of Schaum et al*.* [6]. The reason for this is not clear and data analysis does not highlight technical issues with the pancreas RNA-Seq dataset, however, these results should be interpreted with caution since RNA isolation may be affected by the high level of ribonucleases present in this tissue [58, 59].

The observed gene expression alterations across the lifespan generally reflect loss of tissue function and homeostasis (Supplementary Discussion), with different tissues exhibiting diverse onsets of gene expression dysregulation, as has been described before [6, 20]. We have also observed the existence of significant sex-dimorphic expression patterns in the brain, muscle, and liver, with the former exhibiting the lowest number of sex-biased modules, and the latter displaying the highest. This observation is concordant with previous research in mice that showed the highest dimorphic gene expression to occur in the liver, followed by the muscle, and then by the brain [36]. These findings are also in line with those from the original study of the dataset re-analyzed in this work, which described prominent sex effects in gene expression in the liver and other tissues, not mentioning, however, neither the brain nor the muscle [6]. Sex-differences in gene expression have already been reported for the heart [60] and the pancreas [61] in healthy humans and mice, respectively, however, we did not find modules significantly correlated with sex in the heart and in the pancreas. One limitation of our study, which is inherent to the dataset, is the unbalanced number of subjects from both sexes that may mask more subtle, but existing, sex-differences in gene expression in these tissues.

Genes involved in proteostasis-related pathways were significantly enriched in males over females and exhibited expression differences over time in the liver and the muscle of only male mice. For instance, in the liver, we observed sex-dimorphism in the expression of genes related to ER stress response, with decreased expression in males within old age (18-21 months). Notably, a recent study focusing on sex differences in hepatic ER stress described an increase in markers of the unfolded protein response (UPR) in the liver of male rats in the transition from the prepubertal phase to adulthood [62]. In line with these findings, we observed a peak in the expression of ER-stress related genes in the adult age prior to the described decrease throughout the lifespan. For that reason, further studies addressing sex-differences in these markers in a larger number of time points would be very important for deepening the knowledge on age-related ER-stress. As for the muscle, genes encoding subunits of the proteasome were found to be enriched in males and exhibited increased expression with aging, shifting from under- to over-expression around middle-age (9-12 months). A recent study has reported females as having significantly higher proteasomal activity in several tissues [63], however, this study did not include muscle tissue.

One major implication from our results is that sex-specific alteration in genes involved in proteostasis-related processes can be detrimental to male survival and longevity since these pathways maintain protein quality control and prevent aberrant protein aggregation and proteotoxic stress [recently reviewed by us in 64]. It is well known that proteostasis decline is a hallmark of aging [65], leading to proteome imbalances and contributing to protein aggregation, including of amyloid-like aggregates [66–69], particularly in the muscle [70, 71]. Nonetheless, despite past studies having reported age-related declines in proteostasis in mammals, sex-dimorphism in these processes across the lifespan has been less explored, making this a very interesting subject to address in future research.

Additionally, during liver aging there was a decreased male-enriched expression of genes involved in the biosynthesis of lipids (shift point within old age; 24-27 months) while the muscle showed increased male-biased expression (shift point within middle age; 9-12 months) of genes related to energy metabolism, particularly to the synthesis of ATP (Supplementary Discussion).

Although very few commonalities in age-related dysregulation were observed at the gene-level, we realized that the studied tissues could be grouped according to shared biological processes affected by aging. For the most part, the observed shifts in gene expression occurred within middle-age (9-12 months), as seen in the muscle and liver, however, in the brain and the heart these shifts occurred later in life (15-18 months and 18-21 months, respectively). The brain and the liver had common age-related activation of immune responses, with increased expression of immune-related genes occurring later in the brain (15-18 months) than in the liver (6-9 months and 9-12 months). This activation pattern is consistent with the findings of Schaum et al. [6], while also being implicated in age-related neurodegeneration, as well as in several hepatic age-related diseases, such as non-alcoholic fatty liver disease (NAFLD) and hepatocellular carcinoma (HCC) (Supplementary Discussion). Interestingly, a recent study comparing age-related transcriptomic alterations between different tissue-resident macrophages has shown that the most prominent dysregulation occurred in microglia, followed by Kupffer Cells (KC), the brain and the liver’s resident macrophages, respectively [72; preprint].

Another major implication from our observations is that, similarly to the shift points, the direction of dysregulation varies between tissues, despite shared biological pathways. For instance, the brain exhibits an up-regulation of genes involved in regeneration processes at old age, while the muscle shows an earlier (middle-age) down-regulation of these processes. Interestingly, serpin family genes are altered in both tissues, with the downregulation of *Serpinf1* (serine (or cysteine) peptidase inhibitor, clade F, member 1) and *Serping1* (serine (or cysteine) peptidase inhibitor, clade F, member 1) in the muscle contrasting with the up-regulation of *Serpina3n* (serine (or cysteine) peptidase inhibitor, clade A, member 3N) in the brain. Serpins are a family of serine (or cysteine) protease inhibitors involved in several biological functions including homeostasis control [73]. In the muscle, both *Serpinf1* and *Serping1* are involved in muscle growth and function through regulation of Akt and FoxO signaling pathways [74–76] whereas *Serpina3n*, which has been linked with increased immune response activity, is upregulated in aging astrocytes throughout the brain [77], as well as in a Prion disease mouse model [78], and has been implicated in Alzheimer’s disease (AD) [addressed in 79] and in Multiple Sclerosis (MS) disease progression [80].

Alterations in signaling and cellular response processes were also shared between tissues, with opposing regulation over time in each tissue-pair (Supplementary Discussion). For example, the liver and the heart both showed alterations in genes involved in glucocorticoid signaling, with an early (9-12 months) increase in expression in the liver contrasting with a late life (18-21 months) decrease in expression in the heart. Moreover, the liver and the muscle shared dysregulation of cellular responses to amino acid stimuli with the increase in gene expression in the liver opposing to the decrease in the muscle, albeit the shift point occurring around the same time (9-12 months). Interestingly, the aging heart exhibits a late life (18-21 months) decreased expression of respiratory metabolism genes, while the muscle showed an earlier increased expression of genes involved in the same processes (9-12 months), but only for male mice.

In conclusion, this work opens new research avenues as it highlights many unexplored genes and mechanisms in the context of healthy aging and temporally contextualizes gene expression alterations. We identified tissue-specific key players of aging and addressed the functional implications of their age-related alterations and sex differences in gene expression throughout the lifespan. We also identified groups of tissues based on shared age-affected processes, potentially uncovering tissue axes of common age-related functional dysregulation. We found that proteostasis impairment is a common feature of aging in the muscle and liver of male mice and may dictate sex-differences in lifespan. Because these alterations occur at a transcriptional level and protein abundances can be post-transcriptionally regulated, we are currently working on integrating these results with translatomic and proteomic data to further explore these findings. Additionally, expanding this approach to the other tissues present in the dataset would be pivotal to comprehensively understand this phenomenon and successfully promote healthy aging strategies.

## MATERIALS AND METHODS

### Dataset characterization

The mouse bulk RNA-Seq data used in this study was made publicly available by the Tabula Muris Consortium [6, 45], and is deposited in NCBI’s Gene Expression Omnibus (GEO) under the GEO Series accession number GSE132040 [44]. The original dataset consists of transcriptomic data from 17 male and female mouse tissues across 10 time points (1, 3, 6, 9, 12, 15, 18, 21, 24, and 27 months). For this study, we excluded the 1-month-old samples to avoid the influence of developmental genes [34], and selected the brain, heart, muscle, liver, and pancreas for further analysis (Supplementary Table 9). The RNA extraction, cDNA library preparation, RNA sequencing, read quality control, pre-processing and alignment, transcriptome reconstruction, and expression quantification steps are reported in the original study, in the GEO webpage (GSE132040 entry), and in the protocols.io repository (2uvgew6 entry) [6, 44, 81]. Briefly, libraries were sequenced on the NovaSeq 6000 Sequencing Systems (Illumina) and originated 100-bp paired-end reads which were then de-multiplexed with bcl2fastq (v.2.20.). Read alignment to the GRCm39.p6 (*Mus musculus*) reference genome with Gencode (v.M19) annotations was performed using STAR (v. 2.5.2b). Transcript reconstruction and gene expression quantification was performed with HTSeq (v. 0.6.1p1).

### Data pre-processing and normalization

Similar to what was described in the original study [6], we discarded samples with library size smaller than 4.000.000 reads across all genes (Supplementary Table 7). We further carried out outlier sample identification and removal, which was not performed in the original analysis. Outlier samples were identified based on the sample network approach [82, 83] and excluded if their standardized connectivities (z.K) were more than 2 standard deviations away from the mean z.K (Supplementary Table 9; Supplementary Figure 5).

Gene symbols were associated with Ensembl (release 99) biotype annotations [84] (*Mus musculus* reference genome GRCm39) using the R package biomaRt (v. 2.44.0) [85, 86]. Low count genes were pre-filtered and only genes with total read count higher than 10 in at least *n* samples were kept, with *n* corresponding to each tissue’s minimum group size, whereas in the original study no information regarding gene filtering is provided. Read count data was normalized across samples and transformed with DESeq2’s (v. 1.28.1) [87] estimateSizeFactors and vst functions [88], respectively, as described by Schaum and colleagues [6]. PCA based on the 500 genes with highest row-wise variance (i.e., across all samples) was performed to identify the highest contributing sources of variance, but only for the 5 selected tissues. In line with the observations of Schaum et al*.* [6], since all samples segregated mainly by tissue (Figure 1A), the subsequent analyses were carried out separately for the brain, heart, liver, muscle, and pancreas (see Methods - Dataset characterization).

### Trendy segmented regression analysis

Segmented regression analysis was carried out in normalized expression data (see Methods – Data pre-processing and normalization) using Trendy (v. 1.8.2) [46]. For each gene, a set of segmented regression models were fit, with gene expression as a function of time. We also included ‘Sex’ in the model. Each model comprised a varying number of breakpoints, representing a dynamic change in the gene expression profile, and ranging from 0 (linear regression) to 8 (maxK parameter). We also determined a minimum number of samples present in a segment (expression profile interrupted by a breakpoint), based on the minimum group size in each tissue (brain: 3, heart: 4, liver: 3, muscle: 2, pancreas: 2). Then, the method selected the fitted model having the optimal number of breakpoints by identifying the one with the smallest Bayesian information criterion (BIC) value. We defined top dynamic genes based on the adjusted R^2^ values (indicative of the goodness of fit of a model) and on the segment slope *p-values*. The cutoff for the adjusted R^2^ was defined for each tissue independently (brain: 0.2, heart: 0.2, liver: 0.1, muscle: 0.3, pancreas: 0.3) and chosen as the value above which less than 1% of the genes were kept after a permutation procedure [as performed in 48]. Thus, a gene was considered to be top dynamic (Trendy) if the adjusted R^2^ was higher than the tissue-specific established cutoff and if at least one segment was significant (segment slope *p-value* < 0.1). For the comparison of breakpoint distribution between the tissues, we performed a Kruskal-Wallis test, followed by a Dunn’s test of multiple comparisons using the kruskal.test and dunnTest functions, from the R packages stats (v. 4.1.0) and FSA (v. 0.8.32), respectively. For testing biotype enrichment, we performed the Fisher’s Exact Test using the R function chisq.test, from the stats (v. 4.1.0) package.

### Gene co-expression network construction and module construction

The WGCNA R package (v. 1.69) [47] was used to construct co-expression networks for the VST-transformed data (see Methods – Differential gene expression analysis). Due to the large size of the datasets, an automatic block-wise network construction and module detection approach was chosen [89].

First, genes with zero variance across all samples were flagged and excluded. Then, for each filtered dataset a correlation matrix was calculated based on biweight midcorrelation (bicor) values and raised to specific soft thresholding powers (β; brain: 7; heart: 7; liver: 5; muscle: 6; pancreas: 7; Supplementary Figure 6). In the cases where the scale-free topology fit index failed to reach values above 0.8, the soft-threshold power was chosen based on the number of samples and mean connectivity values [90]. The resultant signed adjacency matrices were used to compute measures of topological overlap between each pair of genes, present in Topological Overlap Matrices (TOM). Next, genes in each dataset were hierarchical clustered (average linkage method) based on topological overlap dissimilarity (1- TOM). Modules of co-expressed genes were constructed accounting for a minimum size of 50 genes, a dendrogram branch merge cut height of 0.15, and default module detection sensitivity (deepsplit = 2) for all datasets except for the liver (deepsplit = 4).

### Identification of age- and sex-associated modules, hub genes, and Trendy-module-hub overlapping genes

An initial selection of modules was based on the association of each module eigengene (ME) with age and sex. Age was treated as a continuous variable, while sex was transformed into a binary variable, with 0 encoding females and 1 encoding males. ME is the first principal component of the expression matrix of a module and is usually considered to be the most representative gene expression profile of that group of correlated genes. The association between a given module and the trait of interest was calculated using bicor values. All modules whose ME displays a significant (FDR adjusted *p-values* < 0.05), moderate or higher (≥ 0.5) correlation with age were selected for subsequent analyses. Next, for each of the selected modules, module membership (MM) and gene significance (GS) measures were calculated. MM results from correlating the expression of individual genes to the ME, whereas GS corresponds to the absolute value of the correlation between individual genes and the trait of interest. Similar to the previous step, only modules with moderate or higher (≥ 0.5) and significant (*p-values* < 0.05) correlations were considered to be relevant. Lastly, for each selected module, genes with individual GS > 0.2 and MM > 0.8 were considered to be the most functionally important, i.e. hub genes (as seen in [91–93]). The R package UpSetR (v. 1.4.0) [94] was used to calculate and visualize the overlap between Trendy, module, and hub genes.

### Functional characterization of Trendy-module-hub genes’ overlap

To functionally characterize the gene lists corresponding to the intersection of Trendy, module, and hub genes per tissue, we performed over-representation analysis of GO BPs using the R package clusterProfiler (v. 3.16.0) [95]. Because this package requires NCBI’s Entrez Gene IDs as input, we converted gene symbols into EntrezIDs with the org.Mm.eg.db R package (v. 3.11.4) [96]. We considered each tissue’s expressed genes (after filtering; see Methods – Data pre-processing and normalization) as the universe for over-representation analysis. GO terms with an FDR adjusted *p-value* less than 0.05 were selected for subsequent analyses.

### Network visualization of functionally enriched terms

Network visualization of the enriched GO terms used the enrichmentMap plugin (v. 3.3.0) [97] of Cytoscape (v. 3.8.0) [98], with nodes representing GO terms, and edges depicting similarity scores based on the number of genes in common between nodes. To construct our networks, we set an edge similarity cutoff of 0.7. GO term redundancy was addressed with the AutoAnnotate (v. 1.3.3) [55], clusterMaker2 (v. 1.3.1) [99], and WordCloud (v. 3.1.3) [100] plug-ins from Cytoscape. Similar GO terms were clustered together using the Markov Clustering Algorithm (MCL), also with an edge similarity cutoff of 0.7, and cluster labels were created with the default label algorithm Adjacent Words, with 4 maximum words per label and an adjacent word bonus of 8.

### Code availability

All analyses in the R statistical environment were performed using R (v. 4.0.3) and RStudio (v. 1.1.463). All R code is available at https://github.com/mmccferreira/Aging_2021.

## Supplementary Material

Supplementary Materials

Supplementary Figures

Supplementary Tables 1 and 2

Supplementary Table 3

Supplementary Table 4

Supplementary Table 5

Supplementary Table 6

Supplementary Table 7

Supplementary Table 8

Supplementary Table 9

Supplementary Table 10
