# Supplementary material for: Integration of segmented regression analysis with weighted gene correlation network analysis identifies genes whose expression is remodeled throughout physiological aging in mouse tissues
Source: Aging (Albany NY). 2021 Jul 29;13(14):18150–90. doi: 10.18632/aging.203379 (PMC8351669; doi:10.18632/aging.203379)
Supplement: Supplementary Materials [file aging-13-203379-s001.pdf]

SUPPLEMENTARY MATERIALS

Supplementary Discussion

Tissue-specific aging expression signatures reflect an age-related loss of tissue function and homeostasis

In the brain, we reported an increase in the expression of genes encoding for components of the Major Histocompatibility Complex (MHC) I (H2-T23 (H-2 class I histocompatibility antigen D-37 alpha chain), *H2-D1* (histocompatibility 2, D region locus 1), *H2-K1* (histocompatibility 2, K1, K region) and *B2m* (beta 2 microglobulin)). These observations are concordant with the available literature, indicating that genes involved in immune responses, especially in MHC antigen processing and presentation, are increasingly expressed during aging in the brains of C57BL/6 mice, and probably constitute early markers of age-related neurodegeneration. Apart from *H2-T23*, all of the MHCI genes identified have been recently shown to be upregulated with age in several regions of the central nervous system (CNS) in the mouse [1]. Interestingly, higher levels of MHCI components have been linked with limited synapse density in the mouse hippocampus [2], as well as age-related synaptic loss in murine neuromuscular junctions [3], while other evidence points to an important role of MHCI in maintaining synaptic plasticity in healthy aging brain [4]. Importantly, our results are consistent with observations made in long-lived primate species and human fibroblasts that showed increased expression of MHC antigen presentation pathway genes with age, particularly *B2m* [5]. Furthermore, increased expression of *B2m* has also been shown to result in impaired hippocampal neurogenesis in aged mice, thus contributing to cognitive decline [6].

Regarding the heart, we observed the downregulation of genes involved in energy metabolism, including Pdhb (pyruvate dehydrogenase (lipoamide) beta) and Acaa2 (acetyl-Coenzyme A acyltransferase 2 (mitochondrial 3-oxoacyl-Coenzyme A thiolase)). Pdhb is involved in the irreversible oxidative decarboxylation of pyruvate, as it encodes for a catalytic enzyme (E1β subunit) of the pyruvate dehydrogenase complex (PDC) [7, 8]. Although its role in the aging heart remains unclear, PDC is crucial in mitochondrial energy production with its end products acetyl-CoA and NADH being central molecules in the Krebs cycle and mitochondrial respiration, respectively [7, 9]. Interstingly, higher efficiency of PDC activity has been reported in older F344 rats [10] and, more recently, heart failure patients reportedly showed increased PDC activity in the left ventricular myocardium, characterized by greater expression levels of PDC catalytic enzymes, including E1β [11]. It has also been shown that PDC activation is able to improve cardiac function in murine hearts [12], with the beneficial effects of PDC activity on heart function probably being due to increasing energy production under large energetic demand conditions. Furthermore, Acaa2 is involved in fatty acid beta-oxidation, which also generates acetyl-CoA and NADH [13–15]. Despite not being studied in the context of aging, this gene has been shown to play a role in maintaining proper cardiac function, with possible implications for age-associated heart dysfunction. In heart failure-induced rats, a treatment successfully improved myocardial energy metabolism through the upregulation of the expression of genes involved in fatty acid metabolism, including Acaa2 [16]. Additionally, an aging-induced decline in fatty acid oxidation has also been reported in the hearts of aging mice [17]. We also observed the decrease in expression of other genes involved in fatty acid oxidation in this tissue, particularly of Acaa1a (acetyl-Coenzyme A acyl-transferase 1A), Adipor1 (adiponectin receptor 1), Auh (AU RNA binding protein/enoyl-coenzyme A hydratase), Eci2 (enoyl-Coenzyme A delta isomerase 2), Etfb (electron transferring flavoprotein, beta polypeptide), and Hadh (hydroxyacyl-Coenzyme A dehydrogenase), which corroborates previous reports of age-related cardiac dysfunction mediated by cardiac lipotoxicity as a result of impaired oxidation of fatty acids [recently reviewed in 18]. Together, these observations indicate a general impairment of cardiac energy substrate metabolism and suggest that the energy requirements of the aging heart are severely compromised.

As for the muscle, we found a general decline in the expression of genes mainly involved in regulating muscle hypertrophy, regeneration, and homeostasis, as is the case of *Anxa2* (annexin A2), *Lrp1* (low density lipoprotein receptor-related protein 1) and *Fn1* (fibronectin 1). Regarding *Annexin A2*, it encodes for a protein belonging to the annexin family and is known to play an important role in plasma membrane repair of skeletal muscle cells [19–21]. Loss of *Anxa2* is associated with impaired myofiber repair and regeneration as well as progressive muscle weakening with age [22]. Interestingly, and contrary to our observations, *Anxa2* expression has been found to increase with age both in healthy humans [23] and in ad libitum fed rats [24]. *Lrp1* is a large endocytic receptor involved in muscle fibrosis, where Lrp1-Decorin pathway leads to activation of TGF-β, promoting the expression of pro-fibrotic molecules [25, 26]. Additionally, *Lrp1* depletion impairs fracture repair in the bones of old mice, while overexpression

improves it [27]. Notwithstanding these observations, the role of *Lrp1* should be further elucidated in the context of skeletal muscle aging. Finally, fibronectin is an extracellular matrix component that has been shown to be an important player in muscle fiber regeneration by interacting with satellite cells, the muscle's stem cells [28]. Our observations of decreased levels of *Fn1* with aging are in line with previous findings reporting not only that the aged stem cell niche displays substantially lower fibronectin mRNA and protein levels, as well as that the knock-out of Fn1 results in decreased numbers of muscle stem cells [29]. Overall, our results indicate that skeletal muscle structure and functioning declines with age, where key genetic players in muscle regeneration are found to be downregulated. However, the exact role and underlying mechanisms of these genes in the aging mammalian skeletal muscle remains a marker of interest to be explored in future studies.

Lastly, we observed an overall increase in gene expression associated with immune responses during hepatic aging, with significantly dysregulated genes including the inflammatory chemokine *Ccl5* (chemokine (C-C motif) ligand 5), *Cd79a* (CD79A antigen (immunoglobulin-associated alpha)) and *Cd79b* (CD79B antigen), and *H2-Aa* (histocompatibility 2, class II antigen A, alpha) and *H2-Eb1* (histocompatibility 2, class II antigen E beta). Ccl5 is a chemokine – chemotactic cytokine – involved in directing leukocyte migration [30]. In agreement with our observations, *Ccl5* mRNA levels were reported to be significantly increased in aged mice, and accompanied by other markers of chronical inflammation [31]. Moreover, up-regulation of *Ccl5* expression has been linked to several hepatic diseases, many of them having age as an important risk factor, such as non-alcoholic fatty liver disease (NAFLD) and hepatocellular carcinoma (HCC) [32–34]. *Cd79a* and *Cd79b* encode for components of the B cell antigen receptor, whose expression is important for B cell maturation [35]. In line with our observations of increased expression with aging of these genes, Schaum et al., the authors of the original study, report high numbers of B cells in the livers of old mice (18-30m) based on *Cd79a* expression [36]. Moreover, a different study of the same authors reported age-related overexpression of MHC II antigens H2-Aa and H2-Eb1 in the same tissue [37]. Up-regulation of these genes has also been found in a NAFLD mouse model [38]. Taken together, these findings are in agreement with the notion that exacerbation or dysregulation of inflammatory response is associated with liver pathology [addressed in 39], expanding it to the aging process.

Sex-dimorphic expression of genes involved in metabolic-related pathways

We also reported a decreased male-enriched expression of genes involved in the biosynthesis of lipids in the aging liver (shift point within old age; 24-27 months), including *Elovl2* and *Elovl3* (elongation of very long chain fatty acids *(FEN1/Elo2, SUR4/Elo3, yeast)-like 2* and *3*), encoding for fatty acid elongase enzymes. These observations are in agreement with a previously found male bias of genes enriched in functions related to fatty acid metabolism in the liver of mammals [40], even though the dynamics of their expression over time haven't been addressed. Interestingly, a recent critical review on sex-differences in NAFLD has pinpointed the male sex as a positive risk factor for the occurrence of this disease that, not only is age-related, but also is characterized by hepatic lipid accumulation [41]. Together, this evidence suggest a male-biased impair of lipid metabolism with aging, most likely mediated by intensification of lipid biosynthesis, however, more studies are needed to enlighten the potential mechanisms behind these observations.

Furthermore, we observed an increased male-biased expression with aging (shift point within middle age; 9-12 months) of genes related to energy metabolism, particularly to the synthesis of adenosine triphosphate (ATP), in the muscle. In line with our findings, a previous study regarding sex-differences in gene expression in human skeletal muscle reported higher expression of genes encoding mitochondrial proteins in men, albeit the influence of age was not explored [42]. More indirectly, an age-related increase in oxidative damage in human skeletal muscle was observed and was more prominent in males than in females [43], which may be explained by increased ATP production with aging in this group. Nevertheless, despite sex-differences in muscle energy metabolism being reported [reviewed in 44], and some evidence regarding age-related sexual dimorphism in these processes existing [addressed in 45], these differences have not been well characterized and more research is needed to improve our understanding on this matter.

Alterations in signaling and cellular response processes between the aging heart, liver and muscle

Alterations in signaling and cellular response processes are also shared between tissues. For example, the liver and the heart both show alterations in genes involved in glucocorticoid signaling, with an early (9-12 months) increase in expression in the liver contrasting with a late life (18-21 months) decrease in expression in the heart. Moreover, the liver and the muscle share dysregulation of cellular responses to amino acid stimuli with the

increase in gene expression in the liver opposing to the decrease in the muscle, albeit the shift point occurring around the same time (9-12 months).

Interestingly, only one gene is involved in both cases in the liver – *Ntrk2* (neurotrophic tyrosine kinase, receptor, type 2). *Ntrk2* encodes for tyrosine receptor kinase B (TrkB), one of the three tropomyosin kinase receptors, that are mainly expressed in the brain and known to play an essential role in the homeostasis of this tissue as it is involved in neuronal differentiation and survival and synaptic formation and plasticity, among others [reviewed in 46]. This gene has also been reported to be expressed in the murine liver, being probably involved in the innervation of this tissue [47]. Notably, recent evidence has implied the overexpression of one isoform of TrkB (TRKB-T1) in the pathogenesis of non-alcoholic steatohepatits (NASH), an advance form of NAFLD, through the promotion of inflammatory signaling in hepatocytes and stress-induced cell death [48]. Furthermore, increased expression of *Ntrk2* has also been suggested to contribute to the exacerbation of hepatocarcinogenesis in a mouse model of hepato-cellular carcinoma [49]. Despite the lack of direct evidence establishing a link between the aging liver and TrkB signaling, our observations of increased *Ntrk2* expression across the lifespan suggest a role of this gene in age-related liver dysfunction, albeit further studies are needed.

Additionally, in the heart, we observed the decreased expression of genes involved in both the positive (*Ppp5c* - protein phosphatase 5, catalytic subunit) [50] and negative (*Phb* - prohibitin) [51] regulation of glucocorticoid signaling, evincing an age-related dysregulation of this pathway in the heart, potentially impacting cardiac function, in line with previous reports [addressed in 52]. In the muscle, we also report an age-related decrease in the expression of genes encoding some collagen proteins (*Col3a1*, *Col5a2*, *Col6a1* – collagen type III, V and VI, alpha 1 and 2), which is in agreement of recent study reporting the down-regulation of fibrillar, fibril-associated and networking collagen genes in aged skeletal muscle of C57BL/6 mice [53].

Dysregulation of respiratory metabolism genes in the aging heart and in the muscle of aged males

Interestingly, the aging heart shares dysregulation of respiratory metabolism genes with the muscle tissue of male mice. Age-related mitochondrial dysfunction in the heart has been widely studied and reported [for reviews, see 54–57], being characterized, among other markers, by decreased mitochondrial respiration activity [57], which is in agreement with our observations of decreased expression of genes involved in this process.

As for the muscle, sex-differences in energy metabolism have already been addressed (see *Sex-dimorphic expression of genes involved in metabolic-related pathways*), with the conclusion that more studies are needed to better understand the mechanisms behind sexual dimorphism in mitochondrial respiration dysregulation, and potential implications for the aging process.

Supplementary References

1. Mangold CA, Masser DR, Stanford DR, Bixler GV, Pisupati A, Giles CB, Wren JD, Ford MM, Sonntag WE, Freeman WM. CNS-wide Sexually Dimorphic Induction of the Major Histocompatibility Complex 1 Pathway With Aging. J Gerontol A Biol Sci Med Sci. 2017; 72: 16–29. https://doi.org/10.1093/gerona/glv232 PMID:26786204

2. Dixon-Salazar TJ, Fourgeaud L, Tyler CM, Poole JR, Park JJ, Boulanger LM. MHC class I limits hippocampal synapse density by inhibiting neuronal insulin receptor signaling. J Neurosci. 2014; 34:11844–56. https://doi.org/10.1523/JNEUROSCI.4642-12.2014 PMID:25164678

3. Tetruashvily MM, McDonald MA, Frietze KK, Boulanger LM. MHCI promotes developmental synapse elimination and aging-related synapse loss at the vertebrate neuromuscular junction. Brain Behav Immun. 2016; 56:197–208. https://doi.org/10.1016/j.bbi.2016.01.008 PMID:26802986

4. Lazarczyk MJ, Kemmler JE, Eyford BA, Short JA, Varghese M, Sowa A, Dickstein DR, Yuk FJ, Puri R, Biron KE, Leist M, Jefferies WA, Dickstein DL. Major Histocompatibility Complex class I proteins are critical for maintaining neuronal structural complexity in the aging brain. Sci Rep. 2016; 6:26199. https://doi.org/10.1038/srep26199 PMID:27229916

5. Pickering AM, Lehr M, Miller RA. Lifespan of mice and primates correlates with immunoproteasome expression. J Clin Invest. 2015; 125:2059–68. https://doi.org/10.1172/JCI80514 PMID:25866968

6. Smith LK, He Y, Park JS, Bieri G, Snethlage CE, Lin K, Gontier G, Wabl R, Plambeck KE, Udeochu J, Wheatley EG, Bouchard J, Eggel A, et al. β2-microglobulin is a systemic pro-aging factor that impairs cognitive function and neurogenesis. Nat Med. 2015; 21:932–37. https://doi.org/10.1038/nm.3898 PMID:26147761

7. Byron O, Lindsay JG. The Pyruvate Dehydrogenase Complex and Related Assemblies in Health and Disease. Subcell Biochem. 2017; 83:523–50.

https://doi.org/10.1007/978-3-319-46503-6_19
PMID:28271489

8. Patel MS, Nemeria NS, Furey W, Jordan F. The pyruvate dehydrogenase complexes: structure-based function and regulation. J Biol Chem. 2014; 289: 16615–23.
https://doi.org/10.1074/jbc.R114.563148
PMID:24798336

9. Stacpoole PW. The pyruvate dehydrogenase complex as a therapeutic target for age-related diseases. Aging Cell. 2012; 11:371–77.
https://doi.org/10.1111/j.1474-9726.2012.00805.x
PMID:22321732

10. Moreau R, Heath SH, Doneanu CE, Harris RA, Hagen TM. Age-related compensatory activation of pyruvate dehydrogenase complex in rat heart. Biochem Biophys Res Commun. 2004; 325:48–58.
https://doi.org/10.1016/j.bbrc.2004.10.011
PMID:15522199

11. Sheeran FL, Angerosa J, Liaw NY, Cheung MM, Pepe S. Adaptations in Protein Expression and Regulated Activity of Pyruvate Dehydrogenase Multienzyme Complex in Human Systolic Heart Failure. Oxid Med Cell Longev. 2019; 2019:4532592.
https://doi.org/10.1155/2019/4532592
PMID:30881593

12. Handzlik MK, Constantin-Teodosiu D, Greenhaff PL, Cole MA. Increasing cardiac pyruvate dehydrogenase flux during chronic hypoxia improves acute hypoxic tolerance. J Physiol. 2018; 596:3357–69.
https://doi.org/10.1113/JP275357
PMID:29383727

13. Bartlett K, Eaton S. Mitochondrial beta-oxidation. Eur J Biochem. 2004; 271:462–69.
https://doi.org/10.1046/j.1432-1033.2003.03947.x
PMID:14728673

14. Ellis JM, Mentock SM, Depetrillo MA, Koves TR, Sen S, Watkins SM, Muoio DM, Cline GW, Taegtmeyer H, Shulman GI, Willis MS, Coleman RA. Mouse cardiac acyl coenzyme a synthetase 1 deficiency impairs Fatty Acid oxidation and induces cardiac hypertrophy. Mol Cell Biol. 2011; 31:1252–62.
https://doi.org/10.1128/MCB.01085-10
PMID:21245374

15. Stanley WC, Recchia FA, Lopaschuk GD. Myocardial substrate metabolism in the normal and failing heart. Physiol Rev. 2005; 85:1093–129.
https://doi.org/10.1152/physrev.00006.2004
PMID:15987803

16. Gao K, Zhang J, Gao P, Wang Q, Liu Y, Liu J, Zhang Y, Li Y, Chang H, Ren P, Liu J, Wang Y, Wang W. Qishen granules exerts cardioprotective effects on rats with heart failure via regulating fatty acid and glucose metabolism. Chin Med. 2020; 15:21.
https://doi.org/10.1186/s13020-020-0299-9
PMID:32158496

17. Hyyti OM, Ledee D, Ning XH, Ge M, Portman MA. Aging impairs myocardial fatty acid and ketone oxidation and modifies cardiac functional and metabolic responses to insulin in mice. Am J Physiol Heart Circ Physiol. 2010; 299:H868–75.
https://doi.org/10.1152/ajpheart.00931.2009
PMID:20601465

18. Drosatos K. Fatty old hearts: role of cardiac lipotoxicity in age-related cardiomyopathy. Pathobiol Aging Age Relat Dis. 2016; 6:32221.
https://doi.org/10.3402/pba.v6.32221
PMID:27558317

19. Lennon NJ, Kho A, Bacskai BJ, Perlmutter SL, Hyman BT, Brown RH Jr. Dysferlin interacts with annexins A1 and A2 and mediates sarcolemmal wound-healing. J Biol Chem. 2003; 278:50466–73.
https://doi.org/10.1074/jbc.M307247200
PMID:14506282

20. Roostalu U, Strähle U. *In vivo* imaging of molecular interactions at damaged sarcolemma. Dev Cell. 2012; 22:515–29.
https://doi.org/10.1016/j.devcel.2011.12.008
PMID:22421042

21. Barthélémy F, Defour A, Lévy N, Krahn M, Bartoli M. Muscle Cells Fix Breaches by Orchestrating a Membrane Repair Ballet. J Neuromuscul Dis. 2018; 5:21–28.
https://doi.org/10.3233/JND-170251 PMID:29480214

22. Defour A, Medikayala S, Van der Meulen JH, Hogarth MW, Holdreith N, Malatras A, Duddy W, Boehler J, Nagaraju K, Jaiswal JK. Annexin A2 links poor myofiber repair with inflammation and adipogenic replacement of the injured muscle. Hum Mol Genet. 2017; 26: 1979–91.
https://doi.org/10.1093/hmg/ddx065 PMID:28334824

23. Ubaida-Mohien C, Lyashkov A, Gonzalez-Freire M, Tharakan R, Shardell M, Moaddel R, Semba RD, Chia CW, Gorospe M, Sen R, Ferrucci L. Discovery proteomics in aging human skeletal muscle finds change in spliceosome, immunity, proteostasis and mitochondria. Elife. 2019; 8:e49874.
https://doi.org/10.7554/eLife.49874 PMID:31642809

24. Hord JM, Botchlett R, Lawler JM. Age-related alterations in the sarcolemmal environment are attenuated by lifelong caloric restriction and voluntary exercise. Exp Gerontol. 2016; 83:148–57.
https://doi.org/10.1016/j.exger.2016.08.006
PMID:27534381

25. Cabello-Verrugio C, Brandan E. A novel modulatory mechanism of transforming growth factor-beta signaling through decorin and LRP-1. J Biol Chem. 2007; 282:18842–50.
https://doi.org/10.1074/jbc.M700243200
PMID:17485468

26. Cabello-Verrugio C, Santander C, Cofré C, Acuña MJ, Melo F, Brandan E. The internal region leucine-rich repeat 6 of decorin interacts with low density lipoprotein receptor-related protein-1, modulates transforming growth factor (TGF)-β-dependent signaling, and inhibits TGF-β-dependent fibrotic response in skeletal muscles. J Biol Chem. 2012; 287:6773–87.
https://doi.org/10.1074/jbc.M111.312488
PMID:22203668

27. Vi L, Baht GS, Soderblom EJ, Whetstone H, Wei Q, Furman B, Puviindran V, Nadesan P, Foster M, Poon R, White JP, Yahara Y, Ng A, et al. Macrophage cells secrete factors including LRP1 that orchestrate the rejuvenation of bone repair in mice. Nat Commun. 2018; 9:5191.
https://doi.org/10.1038/s41467-018-07666-0
PMID:30518764

28. Yin H, Price F, Rudnicki MA. Satellite cells and the muscle stem cell niche. Physiol Rev. 2013; 93:23–67.
https://doi.org/10.1152/physrev.00043.2011
PMID:23303905

29. Lukjanenko L, Jung MJ, Hegde N, Perruisseau-Carrier C, Migliavacca E, Rozo M, Karaz S, Jacot G, Schmidt M, Li L, Metairon S, Raymond F, Lee U, et al. Loss of fibronectin from the aged stem cell niche affects the regenerative capacity of skeletal muscle in mice. Nat Med. 2016; 22:897–905.
https://doi.org/10.1038/nm.4126 PMID:27376579

30. Hughes CE, Nibbs RJB. A guide to chemokines and their receptors. FEBS J. 2018; 285:2944–71.
https://doi.org/10.1111/febs.14466 PMID:29637711

31. Singh P, Coskun ZZ, Goode C, Dean A, Thompson-Snipes L, Darlington G. Lymphoid neogenesis and immune infiltration in aged liver. Hepatology. 2008; 47:1680–90.
https://doi.org/10.1002/hep.22224 PMID:18395842

32. Chen L, Zhang Q, Yu C, Wang F, Kong X. Functional roles of CCL5/RANTES in liver disease. Vol. 4, Liver Research. KeAi Communications Co. 2020; 28–34.
https://doi.org/10.1016/j.livres.2020.01.002

33. Sheedfar F, Di Biase S, Koonen D, Vinciguerra M. Liver diseases and aging: friends or foes? Aging Cell. 2013; 12:950–54.
https://doi.org/10.1111/acel.12128
PMID:23815295

34. Li BH, He FP, Yang X, Chen YW, Fan JG. Steatosis induced CCL5 contributes to early-stage liver fibrosis in nonalcoholic fatty liver disease progress. Transl Res. 2017; 180:103–17.e4.
https://doi.org/10.1016/j.trsl.2016.08.006
PMID:27639593

35. Chu PG, Arber DA. CD79: a review. Appl Immunohistochem Mol Morphol. 2001; 9:97–106.
https://doi.org/10.1097/00129039-200106000-00001
PMID:11396639

36. Schaum N, Lehallier B, Hahn O, Pálovics R, Hosseinzadeh S, Lee SE, Sit R, Lee DP, Losada PM, Zardeneta ME, Fehlmann T, Webber JT, McGeever A, et al, and Tabula Muris Consortium. Ageing hallmarks exhibit organ-specific temporal signatures. Nature. 2020; 583:596–602.
https://doi.org/10.1038/s41586-020-2499-y
PMID:32669715

37. Tabula Muris Consortium. A single-cell transcriptomic atlas characterizes ageing tissues in the mouse. Nature. 2020; 583:590–95.
https://doi.org/10.1038/s41586-020-2496-1
PMID:32669714

38. Xiang L, Jiao Y, Qian Y, Li Y, Mao F, Lu Y. Comparison of hepatic gene expression profiles between three mouse models of Nonalcoholic Fatty Liver Disease. Genes Dis. 2021.
https://doi.org/10.1016/j.gendis.2021.02.008

39. Robinson MW, Harmon C, O'Farrelly C. Liver immunology and its role in inflammation and homeostasis. Cell Mol Immunol. 2016; 13:267–76.
https://doi.org/10.1038/cmi.2016.3 PMID:27063467

40. Naqvi S, Godfrey AK, Hughes JF, Goodheart ML, Mitchell RN, Page DC. Conservation, acquisition, and functional impact of sex-biased gene expression in mammals. Science. 2019; 365:eaaw7317.
https://doi.org/10.1126/science.aaw7317
PMID:31320509

41. Lonardo A, Nascimbeni F, Ballestri S, Fairweather D, Win S, Than TA, Abdelmalek MF, Suzuki A. Sex Differences in Nonalcoholic Fatty Liver Disease: State of the Art and Identification of Research Gaps. Hepatology. 2019; 70:1457–69.
https://doi.org/10.1002/hep.30626 PMID:30924946

42. Welle S, Tawil R, Thornton CA. Sex-related differences in gene expression in human skeletal muscle. PLoS One. 2008; 3:e1385.
https://doi.org/10.1371/journal.pone.0001385
PMID:18167544

43. Fanò G, Mecocci P, Vecchiet J, Belia S, Fulle S, Polidori MC, Felzani G, Senin U, Vecchiet L, Beal MF. Age and sex influence on oxidative damage and functional

status in human skeletal muscle. J Muscle Res Cell Motil. 2001; 22:345–51. https://doi.org/10.1023/a:1013122805060 PMID:11808774

44. Rosa-Caldwell ME, Greene NP. Muscle metabolism and atrophy: let's talk about sex. Biol Sex Differ. 2019; 10:43. https://doi.org/10.1186/s13293-019-0257-3 PMID:31462271

45. Gheller BJ, Riddle ES, Lem MR, Thalacker-Mercer AE. Understanding Age-Related Changes in Skeletal Muscle Metabolism: Differences Between Females and Males. Annu Rev Nutr. 2016; 36:129–56. https://doi.org/10.1146/annurev-nutr-071715-050901 PMID:27431365

46. Amatu A, Sartore-Bianchi A, Bencardino K, Pizzutilo EG, Tosi F, Siena S. Tropomyosin receptor kinase (TRK) biology and the role of NTRK gene fusions in cancer. Ann Oncol. 2019 (Suppl_8); 30:viii5–iii15. https://doi.org/10.1093/annonc/mdz383 PMID:31738427

47. García-Suárez O, González-Martínez T, Perez-Perez M, Germana A, Blanco-Gélaz MA, Monjil DF, Ciriaco E, Silos-Santiago I, Vega JA. Expression of the neurotrophin receptor TrkB in the mouse liver. Anat Embryol (Berl). 2006; 211:465–73. https://doi.org/10.1007/s00429-006-0098-9 PMID:16763809

48. Xiong J, Liu T, Mi L, Kuang H, Xiong X, Chen Z, Li S, Lin JD. hnRNPU/TrkB Defines a Chromatin Accessibility Checkpoint for Liver Injury and Nonalcoholic Steatohepatitis Pathogenesis. Hepatology. 2020; 71:1228–46. https://doi.org/10.1002/hep.30921 PMID:31469911

49. Potikha T, Pappo O, Mizrahi L, Olam D, Maller SM, Rabinovich GA, Galun E, Goldenberg DS. Lack of galectin-1 exacerbates chronic hepatitis, liver fibrosis, and carcinogenesis in murine hepatocellular carcinoma model. FASEB J. 2019; 33:7995–8007. https://doi.org/10.1096/fj.201900017R PMID:30897344

50. Gene Ontology Annotations. 2021. http://www.informatics.jax.org/go/term/GO:2000324

51. Gene Ontology Annotations. 2021. http://www.informatics.jax.org/go/term/GO:2000323

52. Cruz-Topete D, Oakley RH, Cidlowski JA. Glucocorticoid Signaling and the Aging Heart. Front Endocrinol (Lausanne). 2020; 11:347. https://doi.org/10.3389/fendo.2020.00347 PMID:32528419

53. Chen WJ, Lin IH, Lee CW, Chen YF. Aged Skeletal Muscle Retains the Ability to Remodel Extracellular Matrix for Degradation of Collagen Deposition after Muscle Injury. Int J Mol Sci. 2021; 22:2123. https://doi.org/10.3390/ijms22042123 PMID:33672763

54. Martín-Fernández B, Gredilla R. Mitochondria and oxidative stress in heart aging. Age (Dordr). 2016; 38:225–38. https://doi.org/10.1007/s11357-016-9933-y PMID:27449187

55. Tocchi A, Quarles EK, Basisty N, Gitari L, Rabinovitch PS. Mitochondrial dysfunction in cardiac aging. Biochim Biophys Acta. 2015; 1847:1424–33. https://doi.org/10.1016/j.bbabio.2015.07.009 PMID:26191650

56. Wang Y, Li Y, He C, Gou B, Song M. Mitochondrial regulation of cardiac aging. Biochim Biophys Acta Mol Basis Dis. 2019; 1865:1853–64. https://doi.org/10.1016/j.bbadis.2018.12.008 PMID:30593894

57. Lesnefsky EJ, Chen Q, Hoppel CL. Mitochondrial Metabolism in Aging Heart. Circ Res. 2016; 118: 1593–611. https://doi.org/10.1161/CIRCRESAHA.116.307505 PMID:27174952
