# Supplementary material for: Integration of segmented regression analysis with weighted gene correlation network analysis identifies genes whose expression is remodeled throughout physiological aging in mouse tissues
Source: Aging (Albany NY). 2021 Jul 29;13(14):18150–90. doi: 10.18632/aging.203379 (PMC8351669; doi:10.18632/aging.203379)
Supplement: Supplementary Figures [file aging-13-203379-s002.pdf]

SUPPLEMENTARY FIGURES

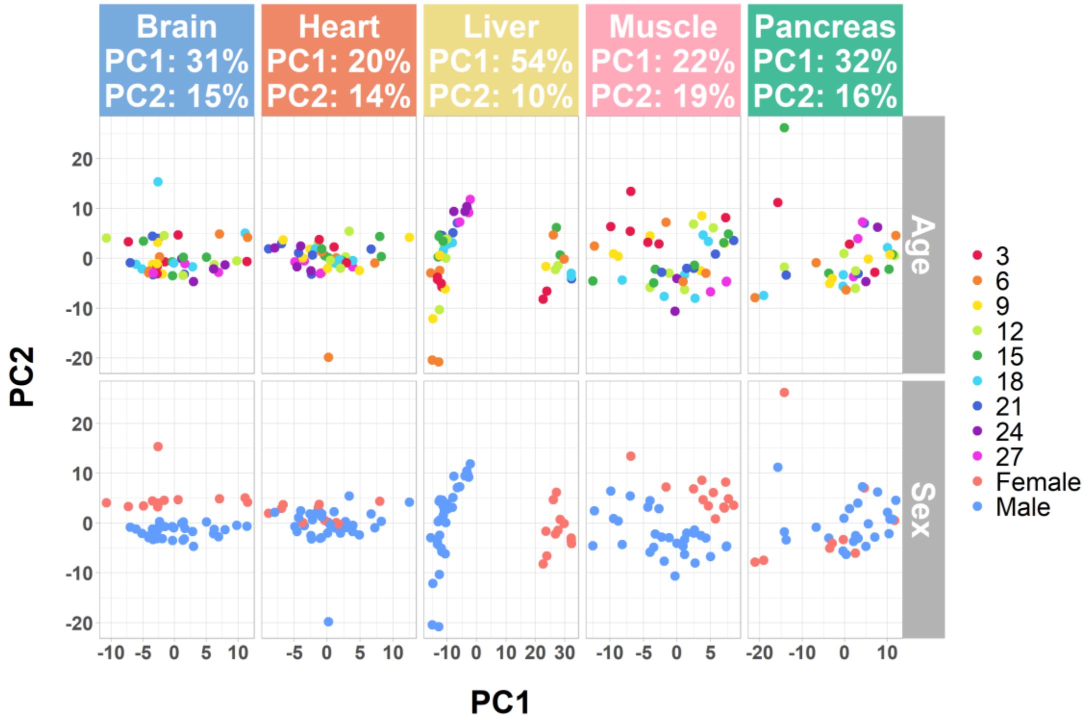

Supplementary Figure 1. Transcript variance per tissue of interest after outlier removal. PCA of selected tissues based on the top 500 most variable genes and colored by age and sex. Because sex is a considerable contributor to sample segregation it was included in the regression model as a co-variable.

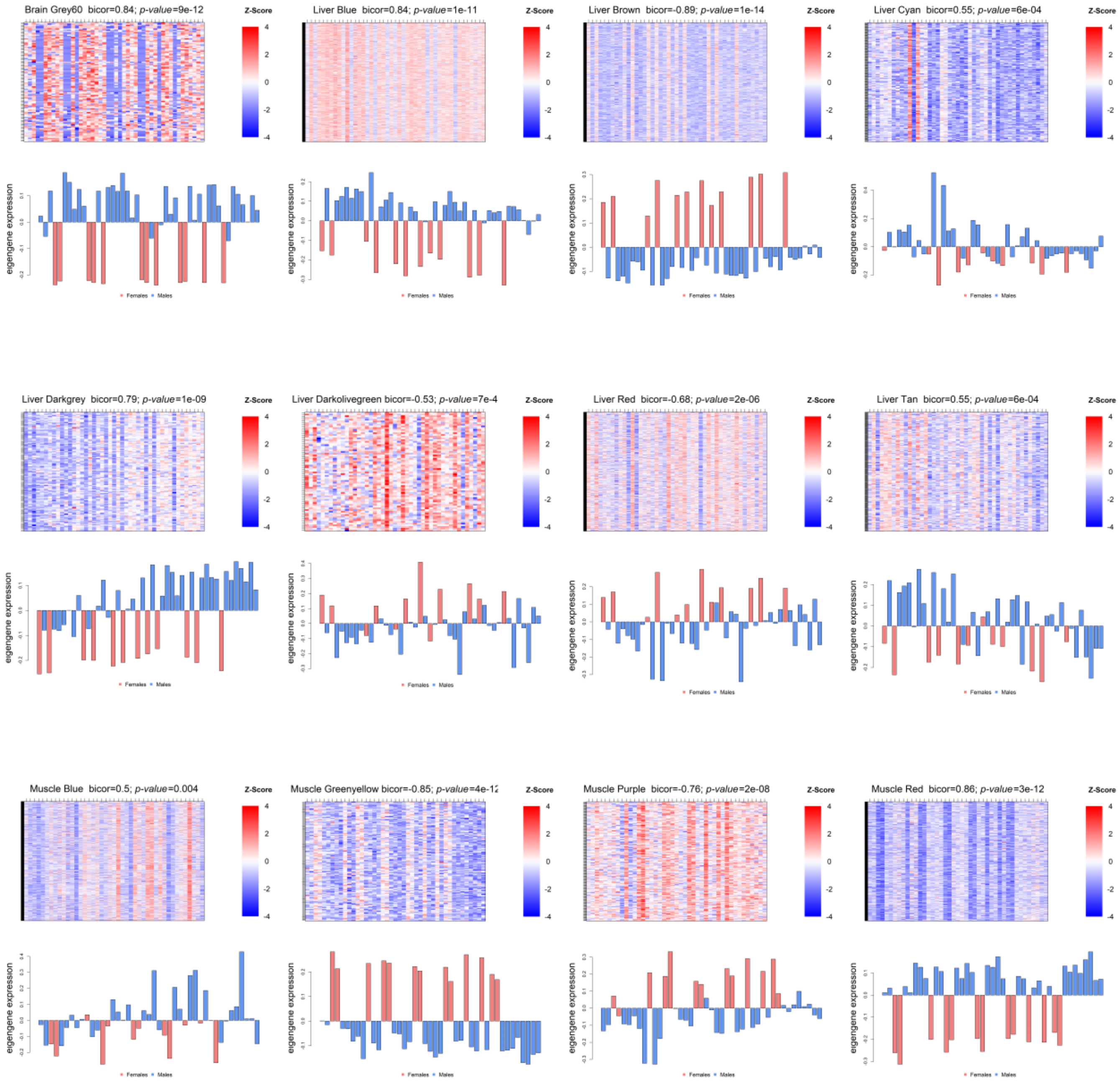

Supplementary Figure 2. Gene expression profiles of the significantly sex-associated modules. Gene expression profile of each significantly sex-associated module. The heatmaps (top) display the standardized expression (*z-score*) of individual genes (rows) per sample (columns), whereas the bar plots (below) represent the ME expression profile. Each bar of the bar plot corresponds to the same samples of the heatmap and are ordered by increasing age (3 to 27 months). Negative (positive) values of ME expression relate to the under-expression (over-expression) of genes in each module's heatmap (blue and red colors, respectively).

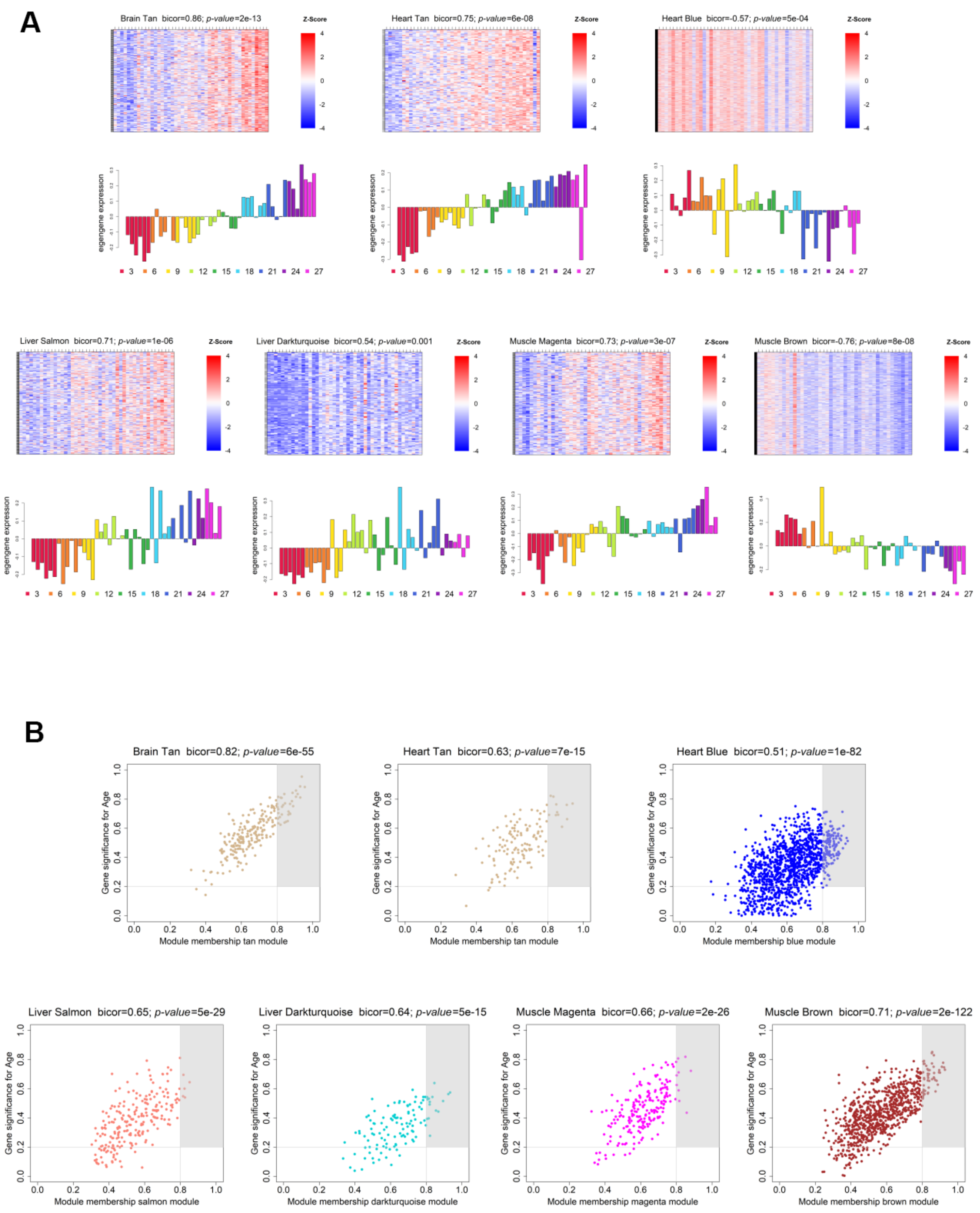

Supplementary Figure 3. Intramodular hub gene identification in the significantly sex-associated modules. For each module, genes with individual GS > 0.2 and MM > 0.8 were considered to be the most functionally important (inside grey rectangles).

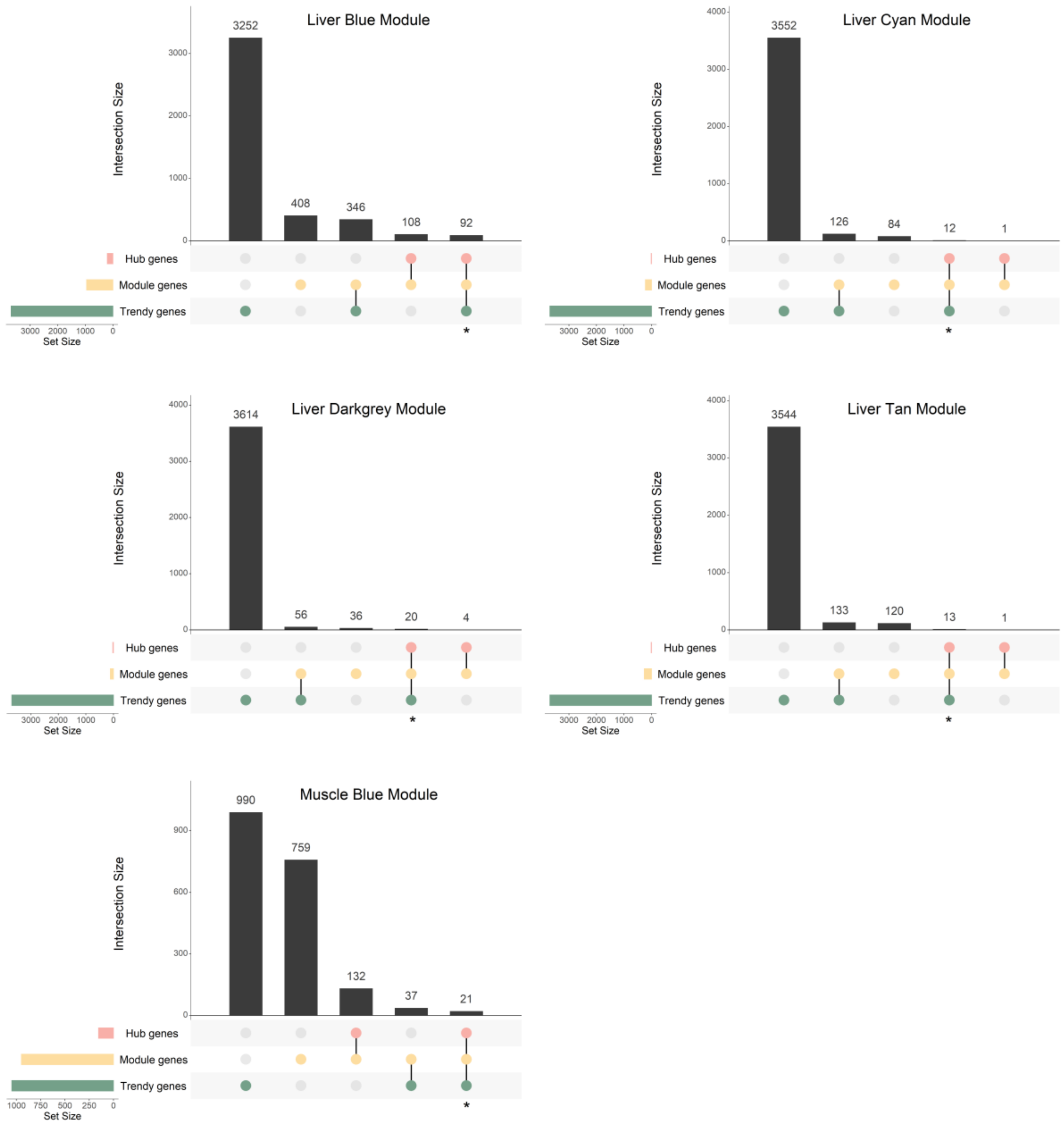

Supplementary Figure 4. Gene overlap between Trendy genes, module genes, and hub genes in the significant sex-associated modules with discernible trends in expression throughout the lfiespan. Bars represent intersection size and colored circles depict the gene sets involved. Genes in common in the Trendy and hub gene sets were considered for further analysis (identified with *).

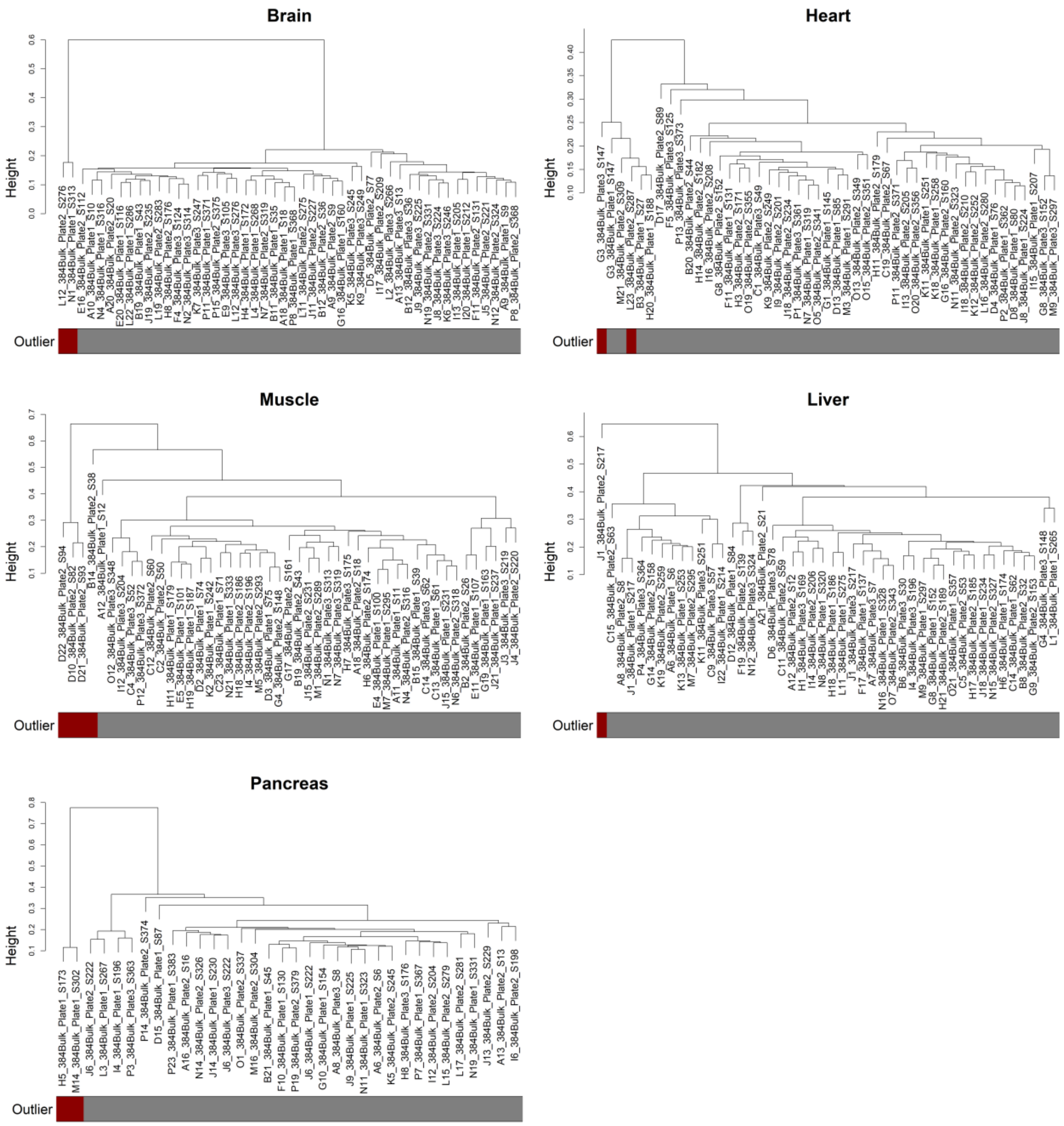

Supplementary Figure 5. Outlier detection by sample network approach [1]. Sample dendrograms were produced by hierarchical clustering using average linkage as the clustering method and 1-A (network adjacency) as the distance between samples. Samples were considered outliers (depicted in red) if their standardized connectivities (z.K, see [1]) were more than 2 standard deviations away from the mean z.K.

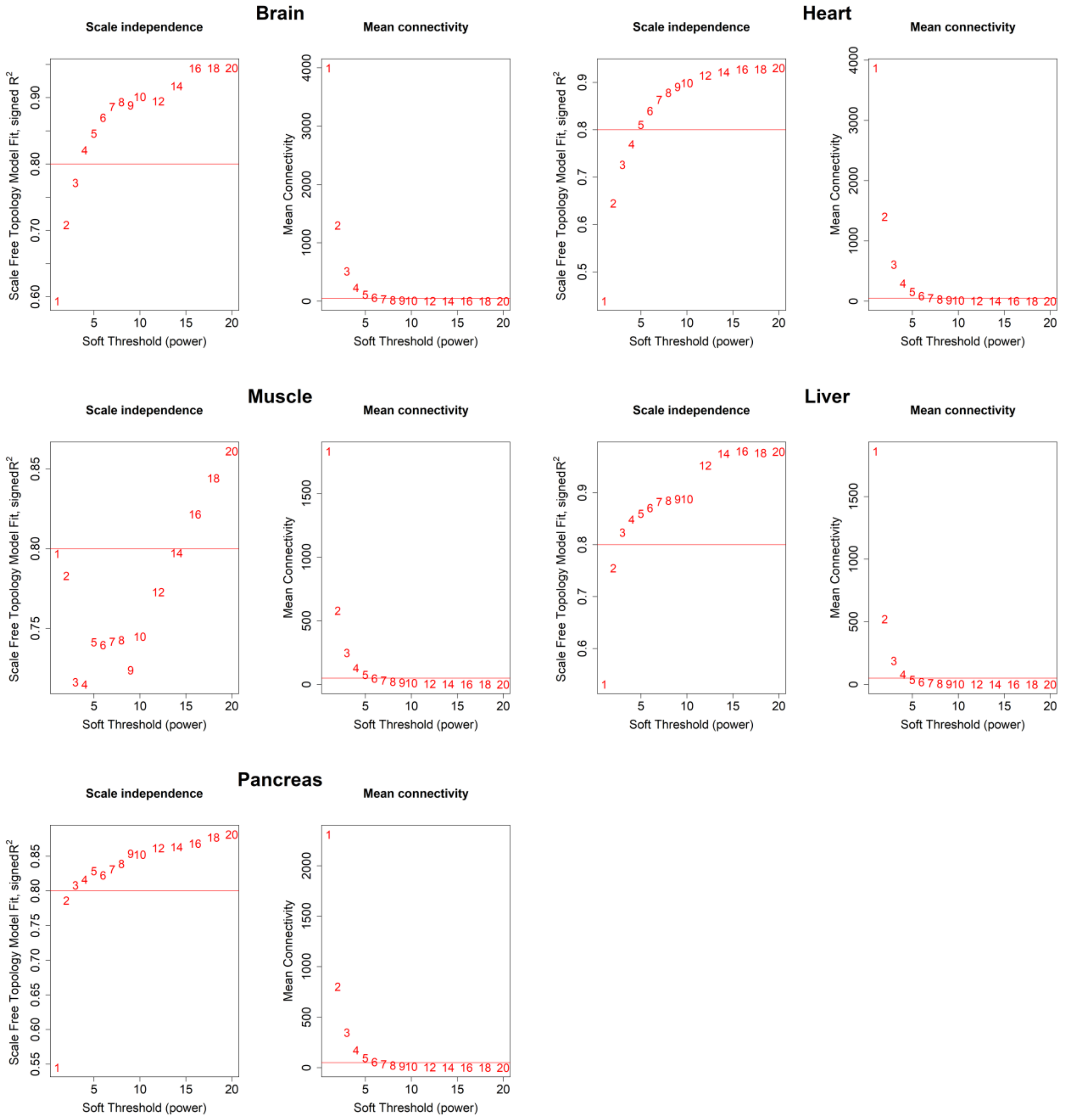

Supplementary Figure 6. Soft-thresholding power determination. Analysis of scale-free fit indexes (left panels) and mean connectivities (right panels) for different soft-thresholding powers (x-axes; red numbers). In cases where there is a lack of fit of scale-free topology, soft-thresholding powers were chosen based on sample number, as proposed by the authors [2].
