# Supplementary material for: Integration of segmented regression analysis with weighted gene correlation network analysis identifies genes whose expression is remodeled throughout physiological aging in mouse tissues
Source: Aging (Albany NY). 2021 Jul 29;13(14):18150–90. doi: 10.18632/aging.203379 (PMC8351669; doi:10.18632/aging.203379)
Supplement: Supplementary Tables 1 and 2 [file aging-13-203379-s003.pdf]

SUPPLEMENTARY TABLES

Supplementary Table 1. Inter-tissue differences in breakpoint distribution.

	Brain	Heart	Liver	Muscle	Pancreas
Brain	-	-	-	-	-
Heart	5.60e-01	-	-	-	-
Liver	9.99e-01	5.03e-01	-	-	-
Muscle	5.14e-13*	1.22e-27*	4.26e-26*	-	-
Pancreas	5.50e-01	9.58e-02	4.50e-01	3.44e-21*	-

Kruskal-Wallis *p*-value < 2.2e-16.
*depicts statistical significancy (adjusted *p-value* < 0.05).
Statistical testing was performed using the non-parametric Kruskal Wallis test, followed by post-hoc Dunn's multiple comparison test, and adjusted *p-values* are presented. *p-value* adjustment was performed using the Benjamini-Hochberg method. H0 states no difference between tissues. Relates to Figure 1C.

Supplementary Table 2. Enrichment of protein coding and long non-coding RNAs (lncRNAs) in each tissue's top dynamic (Trendy) genes.

Tissue	Proportion of PC genes within Trendy genes	Proportion of PC genes within reference genome	PC enrichment *p-value*	Proportion of lncRNAs within Trendy genes	Proportion of lncRNAs within reference genome	lncRNA under-enrichment *p-value*
Brain	0.86		8.277036e-124	0.07		1.736139e-15
Heart	0.83		3.558923e-245	0.07		5.160152e-34
Liver	0.9	0.43	0.000000e+00	0.05	0.18	1.643966e-89
Muscle	0.9		1.141175e-199	0.06		1.568121e-24
Pancreas	0.88		1.450038e-61	0.07		8.077910e-08

PC – protein coding.
lncRNAs – long non-coding RNAs.
Statistical testing was performed using Fisher's Exact Test; H0 implies the proportion of each biotype being equal to the proportion of that biotype in the reference genome's annotation. Relates to Figure 1E.
