# Supplementary material for: Integration of segmented regression analysis with weighted gene correlation network analysis identifies genes whose expression is remodeled throughout physiological aging in mouse tissues
Source: Aging (Albany NY). 2021 Jul 29;13(14):18150–90. doi: 10.18632/aging.203379 (PMC8351669; doi:10.18632/aging.203379)
Supplement: Supplementary Table 3 [file aging-13-203379-s004.docx]

**Supplementary Table 3. Global characterization of WGCNA modules.** Tissue-specific modules of co-expressed genes and number of genes in each module. In all networks, the *grey* module contains unassigned genes. Relates to Figure 2.

| **Tissue** | **Module** | **No. of Genes** | **Total no. of Genes** |
| --- | --- | --- | --- |
| Brain | Black | 411 | 34164 |
|  | Blue | 1818 |  |
|  | Brown | 1006 |  |
|  | Cyan | 120 |  |
|  | Darkgreen | 69 |  |
|  | Darkgrey | 59 |  |
|  | Darkred | 73 |  |
|  | Darkturquoise | 64 |  |
|  | Green | 778 |  |
|  | Greenyellow | 283 |  |
|  | Grey | 17870 |  |
|  | Grey60 | 97 |  |
|  | Lightcyan | 108 |  |
|  | Lightgreen | 97 |  |
|  | Lightyellow | 85 |  |
|  | Magenta | 369 |  |
|  | Midnightblue | 108 |  |
|  | Pink | 372 |  |
|  | Purple | 368 |  |
|  | Red | 613 |  |
|  | Royalblue | 82 |  |
|  | Salmon | 129 |  |
|  | Tan | 220 |  |
|  | Turquoise | 8126 |  |
|  | Yellow | 839 |  |
| Heart | Black | 245 | 28073 |
|  | Blue | 1209 |  |
|  | Brown | 904 |  |
|  | Cyan | 99 |  |
|  | Green | 415 |  |
|  | Greenyellow | 135 |  |
|  | Grey | 14055 |  |
|  | Grey60 | 64 |  |
|  | Lightcyan | 71 |  |
|  | Lightgreen | 63 |  |
|  | Lightyellow | 61 |  |
|  | Magenta | 154 |  |
|  | Midnightblue | 90 |  |
|  | Pink | 181 |  |
|  | Purple | 143 |  |
|  | Red | 257 |  |
|  | Salmon | 107 |  |
|  | Tan | 125 |  |
|  | Turquoise | 9039 |  |
|  | Yellow | 656 |  |
| Liver | Black | 482 | 20157 |
|  | Blue | 954 |  |
|  | Brown | 791 |  |
|  | Cyan | 223 |  |
|  | Darkgreen | 122 |  |
|  | Darkgrey | 116 |  |
|  | Darkmagenta | 66 |  |
|  | Darkolivegreen | 70 |  |
|  | Darkorange | 97 |  |
|  | Darkred | 123 |  |
|  | Darkturquoise | 118 |  |
|  | Green | 595 |  |
|  | Greenyellow | 293 |  |
|  | Grey | 6603 |  |
|  | Grey60 | 182 |  |
|  | Lightcyan | 192 |  |
|  | Lightgreen | 178 |  |
|  | Lightyellow | 158 |  |
|  | Magenta | 416 |  |
|  | Midnightblue | 217 |  |
|  | Orange | 110 |  |
|  | Paleturquoise | 71 |  |
|  | Pink | 449 |  |
|  | Purple | 346 |  |
|  | Red | 536 |  |
|  | Royalblue | 146 |  |
|  | Saddlebrown | 87 |  |
|  | Salmon | 232 |  |
|  | Sienna3 | 64 |  |
|  | Skyblue | 87 |  |
|  | Steelblue | 82 |  |
|  | Tan | 267 |  |
|  | Turquoise | 4772 |  |
|  | Violet | 70 |  |
|  | White | 93 |  |
|  | Yellow | 687 |  |
|  | Yellowgreen | 62 |  |
| Muscle | Black | 241 | 18977 |
|  | Blue | 949 |  |
|  | Brown | 806 |  |
|  | Cyan | 88 |  |
|  | Darkred | 59 |  |
|  | Green | 518 |  |
|  | Greenyellow | 140 |  |
|  | Grey | 9336 |  |
|  | Grey60 | 81 |  |
|  | Lightcyan | 84 |  |
|  | Lightgreen | 73 |  |
|  | Lightyellow | 64 |  |
|  | Magenta | 203 |  |
|  | Midnightblue | 88 |  |
|  | Pink | 214 |  |
|  | Purple | 174 |  |
|  | Red | 484 |  |
|  | Royalblue | 62 |  |
|  | Salmon | 92 |  |
|  | Tan | 135 |  |
|  | Turquosie | 4338 |  |
|  | Yellow | 748 |  |
| Pancreas | Black | 141 | 18411 |
|  | Blue | 1596 |  |
|  | Brown | 981 |  |
|  | Green | 496 |  |
|  | Grey | 9162 |  |
|  | Magenta | 94 |  |
|  | Pink | 129 |  |
|  | Purple | 55 |  |
|  | Red | 475 |  |
|  | Turquoise | 4686 |  |
|  | Yellow | 596 |  |
| *Grey* modules comprise unassigned genes | | | |
