# Supplementary material for: Integration of segmented regression analysis with weighted gene correlation network analysis identifies genes whose expression is remodeled throughout physiological aging in mouse tissues
Source: Aging (Albany NY). 2021 Jul 29;13(14):18150–90. doi: 10.18632/aging.203379 (PMC8351669; doi:10.18632/aging.203379)
Supplement: Supplementary Table 4 [file aging-13-203379-s005.docx]

**Supplementary Table 4. Hub genes present in modules significantly associated with age and sex, and respective gene significance and module membership values.** Hub genes were considered based on module membership (MM) and gene significance (GS) higher than 0.8 and 0.2, respectively. Relates to Figure 3B.

| **Tissue** | **Module** | **Significant association** | **Hub gene** | **Module Membership** | **Gene Significance** |
| --- | --- | --- | --- | --- | --- |
| Brain | Tan | Age | *B2m* | 0.86 | 0.72 |
| Brain | Tan | Age | *C1qa* | 0.87 | 0.70 |
| Brain | Tan | Age | *C1qb* | 0.84 | 0.64 |
| Brain | Tan | Age | *C1qc* | 0.85 | 0.64 |
| Brain | Tan | Age | *C3* | 0.81 | 0.82 |
| Brain | Tan | Age | *C4b* | 0.94 | 0.95 |
| Brain | Tan | Age | *Csf1* | 0.84 | 0.83 |
| Brain | Tan | Age | *Ctsd* | 0.86 | 0.65 |
| Brain | Tan | Age | *Ctsh* | 0.82 | 0.68 |
| Brain | Tan | Age | *Ctss* | 0.91 | 0.86 |
| Brain | Tan | Age | *Ctsz* | 0.87 | 0.70 |
| Brain | Tan | Age | *Cx3cr1* | 0.81 | 0.63 |
| Brain | Tan | Age | *Gbp3* | 0.80 | 0.74 |
| Brain | Tan | Age | *Gfap* | 0.92 | 0.84 |
| Brain | Tan | Age | *H2-D1* | 0.93 | 0.81 |
| Brain | Tan | Age | *H2-K1* | 0.96 | 0.88 |
| Brain | Tan | Age | *H2-T23* | 0.80 | 0.67 |
| Brain | Tan | Age | *Hexb* | 0.86 | 0.69 |
| Brain | Tan | Age | *Ifi27* | 0.87 | 0.77 |
| Brain | Tan | Age | *Ifit3* | 0.88 | 0.79 |
| Brain | Tan | Age | *Il33* | 0.81 | 0.70 |
| Brain | Tan | Age | *Irf7* | 0.81 | 0.73 |
| Brain | Tan | Age | *Itgb2* | 0.83 | 0.81 |
| Brain | Tan | Age | *Lag3* | 0.80 | 0.77 |
| Brain | Tan | Age | *Laptm5* | 0.89 | 0.75 |
| Brain | Tan | Age | *Lgals3* | 0.82 | 0.81 |
| Brain | Tan | Age | *Lgals3bp* | 0.93 | 0.89 |
| Brain | Tan | Age | *Lyz2* | 0.91 | 0.82 |
| Brain | Tan | Age | *Neat1* | 0.89 | 0.84 |
| Brain | Tan | Age | *Psmb8* | 0.84 | 0.77 |
| Brain | Tan | Age | *Serpina3n* | 0.83 | 0.75 |
| Brain | Tan | Age | *Slc11a1* | 0.83 | 0.76 |
| Brain | Tan | Age | *Tap2* | 0.83 | 0.69 |
| Brain | Tan | Age | *Tapbp* | 0.82 | 0.62 |
| Brain | Grey60 | Sex | *Gm18796* | 0.87 | 0.78 |
| Brain | Grey60 | Sex | *Gm18797* | 0.86 | 0.72 |
| Brain | Grey60 | Sex | *Gm20788* | 0.88 | 0.81 |
| Brain | Grey60 | Sex | *Gm20830* | 0.87 | 0.78 |
| Brain | Grey60 | Sex | *Gm21064* | 0.85 | 0.80 |
| Brain | Grey60 | Sex | *Gm21292* | 0.82 | 0.76 |
| Brain | Grey60 | Sex | *Gm21719* | 0.90 | 0.78 |
| Brain | Grey60 | Sex | *Gm21721* | 0.83 | 0.78 |
| Brain | Grey60 | Sex | *Gm21854* | 0.84 | 0.75 |
| Brain | Grey60 | Sex | *Gm21865* | 0.81 | 0.75 |
| Brain | Grey60 | Sex | *Gm21874* | 0.85 | 0.76 |
| Brain | Grey60 | Sex | *Gm28278* | 0.82 | 0.78 |
| Brain | Grey60 | Sex | *Gm28348* | 0.83 | 0.72 |
| Brain | Grey60 | Sex | *Gm28356* | 0.91 | 0.83 |
| Brain | Grey60 | Sex | *Gm28444* | 0.84 | 0.75 |
| Brain | Grey60 | Sex | *Gm28445* | 0.86 | 0.77 |
| Brain | Grey60 | Sex | *Gm28507* | 0.84 | 0.81 |
| Brain | Grey60 | Sex | *Gm28510* | 0.86 | 0.82 |
| Brain | Grey60 | Sex | *Gm28587* | 0.84 | 0.73 |
| Brain | Grey60 | Sex | *Gm28597* | 0.88 | 0.80 |
| Brain | Grey60 | Sex | *Gm28649* | 0.85 | 0.72 |
| Brain | Grey60 | Sex | *Gm28674* | 0.89 | 0.76 |
| Brain | Grey60 | Sex | *Gm28919* | 0.84 | 0.76 |
| Brain | Grey60 | Sex | *Gm29049* | 0.83 | 0.73 |
| Brain | Grey60 | Sex | *Gm29274* | 0.83 | 0.72 |
| Brain | Grey60 | Sex | *Gm37222* | 0.88 | 0.78 |
| Brain | Grey60 | Sex | *Gm37236* | 0.87 | 0.76 |
| Brain | Grey60 | Sex | *Gm8446* | 0.87 | 0.80 |
| Brain | Grey60 | Sex | *Kdm5d* | 0.82 | 0.90 |
| Brain | Grey60 | Sex | *Tspy-ps* | 0.81 | 0.72 |
| Brain | Grey60 | Sex | *Uba1y* | 0.89 | 0.84 |
| Brain | Grey60 | Sex | *Uba1y-ps2* | 0.91 | 0.83 |
| Brain | Grey60 | Sex | *Usp9y* | 0.92 | 0.86 |
| Brain | Grey60 | Sex | *Uty* | 0.80 | 0.88 |
| Brain | Grey60 | Sex | *Vmn2r-ps139* | 0.83 | 0.71 |
| Brain | Grey60 | Sex | *Zfy1* | 0.85 | 0.79 |
| Brain | Grey60 | Sex | *Zfy2* | 0.89 | 0.84 |
| Heart | Tan | Age | *Acsm5* | 0.91 | 0.66 |
| Heart | Tan | Age | *Amy1* | 0.85 | 0.71 |
| Heart | Tan | Age | *Cd209f* | 0.85 | 0.69 |
| Heart | Tan | Age | *Cds1* | 0.86 | 0.69 |
| Heart | Tan | Age | *Ighg2c* | 0.94 | 0.77 |
| Heart | Tan | Age | *Kcnk1* | 0.91 | 0.76 |
| Heart | Tan | Age | *Pcdhb20* | 0.81 | 0.69 |
| Heart | Tan | Age | *Prkcq* | 0.83 | 0.82 |
| Heart | Tan | Age | *Scn4b* | 0.88 | 0.61 |
| Heart | Tan | Age | *Skap2* | 0.85 | 0.76 |
| Heart | Tan | Age | *Vgll2* | 0.82 | 0.82 |
| Heart | Blue | Age | *Acaa1a* | 0.81 | -0.51 |
| Heart | Blue | Age | *Acaa2* | 0.81 | -0.41 |
| Heart | Blue | Age | *Actr1a* | 0.82 | -0.43 |
| Heart | Blue | Age | *Adipor1* | 0.88 | -0.59 |
| Heart | Blue | Age | *Adk* | 0.83 | -0.55 |
| Heart | Blue | Age | *Adsl* | 0.81 | -0.39 |
| Heart | Blue | Age | *Arf1* | 0.87 | -0.47 |
| Heart | Blue | Age | *Atp5b* | 0.88 | -0.49 |
| Heart | Blue | Age | *Atpaf1* | 0.82 | -0.34 |
| Heart | Blue | Age | *Auh* | 0.82 | -0.71 |
| Heart | Blue | Age | *Bcat2* | 0.80 | -0.33 |
| Heart | Blue | Age | *Bsg* | 0.84 | -0.56 |
| Heart | Blue | Age | *C030006K11Rik* | 0.80 | -0.33 |
| Heart | Blue | Age | *Capzb* | 0.83 | -0.44 |
| Heart | Blue | Age | *Casq2* | 0.82 | -0.47 |
| Heart | Blue | Age | *Cct5* | 0.82 | -0.51 |
| Heart | Blue | Age | *Cd81* | 0.81 | -0.42 |
| Heart | Blue | Age | *Cdc34* | 0.82 | -0.36 |
| Heart | Blue | Age | *Cdc37* | 0.86 | -0.36 |
| Heart | Blue | Age | *Chchd3* | 0.84 | -0.61 |
| Heart | Blue | Age | *Ckm* | 0.93 | -0.53 |
| Heart | Blue | Age | *Ckmt2* | 0.86 | -0.55 |
| Heart | Blue | Age | *Cops7a* | 0.88 | -0.48 |
| Heart | Blue | Age | *Cops8* | 0.86 | -0.57 |
| Heart | Blue | Age | *Coq2* | 0.86 | -0.60 |
| Heart | Blue | Age | *Coq5* | 0.86 | -0.50 |
| Heart | Blue | Age | *Coq6* | 0.86 | -0.53 |
| Heart | Blue | Age | *Coq9* | 0.92 | -0.62 |
| Heart | Blue | Age | *Cox10* | 0.84 | -0.51 |
| Heart | Blue | Age | *Ctbp1* | 0.89 | -0.50 |
| Heart | Blue | Age | *Cyc1* | 0.94 | -0.51 |
| Heart | Blue | Age | *Dele1* | 0.88 | -0.55 |
| Heart | Blue | Age | *Des* | 0.84 | -0.49 |
| Heart | Blue | Age | *Dnajb2* | 0.83 | -0.52 |
| Heart | Blue | Age | *Dnpep* | 0.81 | -0.42 |
| Heart | Blue | Age | *Echs1* | 0.83 | -0.28 |
| Heart | Blue | Age | *Eci2* | 0.85 | -0.58 |
| Heart | Blue | Age | *Ecsit* | 0.84 | -0.55 |
| Heart | Blue | Age | *Egln2* | 0.81 | -0.36 |
| Heart | Blue | Age | *Eif5a* | 0.83 | -0.27 |
| Heart | Blue | Age | *Eno3* | 0.91 | -0.71 |
| Heart | Blue | Age | *Etfb* | 0.89 | -0.56 |
| Heart | Blue | Age | *Fars2* | 0.82 | -0.47 |
| Heart | Blue | Age | *Fastk* | 0.90 | -0.55 |
| Heart | Blue | Age | *Fbxw5* | 0.83 | -0.28 |
| Heart | Blue | Age | *Fh1* | 0.83 | -0.56 |
| Heart | Blue | Age | *Fkbp4* | 0.81 | -0.71 |
| Heart | Blue | Age | *Gnpat* | 0.81 | -0.71 |
| Heart | Blue | Age | *Gpi1* | 0.85 | -0.36 |
| Heart | Blue | Age | *Hadh* | 0.84 | -0.43 |
| Heart | Blue | Age | *Hmgcl* | 0.82 | -0.45 |
| Heart | Blue | Age | *Idh3g* | 0.83 | -0.42 |
| Heart | Blue | Age | *Immt* | 0.80 | -0.48 |
| Heart | Blue | Age | *Isca1* | 0.84 | -0.65 |
| Heart | Blue | Age | *Klhdc2* | 0.81 | -0.41 |
| Heart | Blue | Age | *Ldha* | 0.82 | -0.34 |
| Heart | Blue | Age | *Ldhb* | 0.89 | -0.43 |
| Heart | Blue | Age | *Maf1* | 0.82 | -0.45 |
| Heart | Blue | Age | *Map1lc3b* | 0.82 | -0.57 |
| Heart | Blue | Age | *Mccc2* | 0.80 | -0.51 |
| Heart | Blue | Age | *Mdh1* | 0.89 | -0.55 |
| Heart | Blue | Age | *Mdh2* | 0.93 | -0.49 |
| Heart | Blue | Age | *Mrpl37* | 0.84 | -0.53 |
| Heart | Blue | Age | *Mrpl38* | 0.83 | -0.55 |
| Heart | Blue | Age | *Mrpl4* | 0.89 | -0.44 |
| Heart | Blue | Age | *Mrpl45* | 0.82 | -0.50 |
| Heart | Blue | Age | *Mtfr1l* | 0.84 | -0.25 |
| Heart | Blue | Age | *Myzap* | 0.82 | -0.44 |
| Heart | Blue | Age | *Napa* | 0.81 | -0.25 |
| Heart | Blue | Age | *Ndufa10* | 0.88 | -0.42 |
| Heart | Blue | Age | *Ndufa9* | 0.85 | -0.40 |
| Heart | Blue | Age | *Ndufs2* | 0.91 | -0.46 |
| Heart | Blue | Age | *Ndufv1* | 0.84 | -0.42 |
| Heart | Blue | Age | *Nfs1* | 0.83 | -0.53 |
| Heart | Blue | Age | *Obscn* | 0.86 | -0.41 |
| Heart | Blue | Age | *Pdhb* | 0.89 | -0.60 |
| Heart | Blue | Age | *Pdrg1* | 0.80 | -0.38 |
| Heart | Blue | Age | *Pgm2* | 0.84 | -0.43 |
| Heart | Blue | Age | *Phb* | 0.87 | -0.45 |
| Heart | Blue | Age | *Phyh* | 0.85 | -0.55 |
| Heart | Blue | Age | *Pkm* | 0.86 | -0.38 |
| Heart | Blue | Age | *Pmpcb* | 0.86 | -0.42 |
| Heart | Blue | Age | *Poldip2* | 0.81 | -0.40 |
| Heart | Blue | Age | *Popdc2* | 0.82 | -0.21 |
| Heart | Blue | Age | *Ppp1ca* | 0.83 | -0.52 |
| Heart | Blue | Age | *Ppp2r5d* | 0.82 | -0.38 |
| Heart | Blue | Age | *Ppp5c* | 0.83 | -0.52 |
| Heart | Blue | Age | *Prdx3* | 0.87 | -0.57 |
| Heart | Blue | Age | *Prkaca* | 0.88 | -0.39 |
| Heart | Blue | Age | *Psma1* | 0.81 | -0.43 |
| Heart | Blue | Age | *Psmc4* | 0.80 | -0.38 |
| Heart | Blue | Age | *Psmc5* | 0.80 | -0.31 |
| Heart | Blue | Age | *Psmd2* | 0.84 | -0.53 |
| Heart | Blue | Age | *Psmd3* | 0.83 | -0.36 |
| Heart | Blue | Age | *Psmd7* | 0.82 | -0.42 |
| Heart | Blue | Age | *Ptcd2* | 0.82 | -0.47 |
| Heart | Blue | Age | *Ptges2* | 0.87 | -0.53 |
| Heart | Blue | Age | *Pygm* | 0.84 | -0.49 |
| Heart | Blue | Age | *Rnf187* | 0.90 | -0.53 |
| Heart | Blue | Age | *Rpl3l* | 0.85 | -0.61 |
| Heart | Blue | Age | *Rrp1* | 0.81 | -0.33 |
| Heart | Blue | Age | *Rxrg* | 0.83 | -0.51 |
| Heart | Blue | Age | *Samm50* | 0.90 | -0.52 |
| Heart | Blue | Age | *Sdhd* | 0.84 | -0.51 |
| Heart | Blue | Age | *Serf2* | 0.89 | -0.42 |
| Heart | Blue | Age | *Sgca* | 0.84 | -0.44 |
| Heart | Blue | Age | *Slc25a11* | 0.88 | -0.43 |
| Heart | Blue | Age | *Slc25a12* | 0.80 | -0.51 |
| Heart | Blue | Age | *Slc25a39* | 0.84 | -0.41 |
| Heart | Blue | Age | *Slc25a5* | 0.82 | -0.47 |
| Heart | Blue | Age | *Slc2a4* | 0.83 | -0.52 |
| Heart | Blue | Age | *Smpd1* | 0.85 | -0.42 |
| Heart | Blue | Age | *Snta1* | 0.82 | -0.46 |
| Heart | Blue | Age | *Sod2* | 0.81 | -0.42 |
| Heart | Blue | Age | *Stoml2* | 0.80 | -0.40 |
| Heart | Blue | Age | *Suclg1* | 0.86 | -0.51 |
| Heart | Blue | Age | *Tcp1* | 0.81 | -0.55 |
| Heart | Blue | Age | *Tmem70* | 0.81 | -0.63 |
| Heart | Blue | Age | *Tprgl* | 0.83 | -0.58 |
| Heart | Blue | Age | *Tufm* | 0.86 | -0.46 |
| Heart | Blue | Age | *Txn2* | 0.84 | -0.53 |
| Heart | Blue | Age | *Ube2b* | 0.85 | -0.61 |
| Heart | Blue | Age | *Ube2g2* | 0.86 | -0.48 |
| Heart | Blue | Age | *Ubl7* | 0.86 | -0.49 |
| Heart | Blue | Age | *Uqcrc1* | 0.85 | -0.55 |
| Heart | Blue | Age | *Uqcrc2* | 0.90 | -0.53 |
| Heart | Blue | Age | *Wdr18* | 0.81 | -0.30 |
| Heart | Blue | Age | *Yipf3* | 0.84 | -0.47 |
| Liver | Tan | Sex | *Babam1* | 0.83 | 0.52 |
| Liver | Tan | Sex | *Cope* | 0.84 | 0.43 |
| Liver | Tan | Sex | *Gps1* | 0.85 | 0.65 |
| Liver | Tan | Sex | *Grhpr* | 0.82 | 0.67 |
| Liver | Tan | Sex | *Mbl1* | 0.81 | 0.23 |
| Liver | Tan | Sex | *Mgst1* | 0.86 | 0.67 |
| Liver | Tan | Sex | *Mup1* | 0.85 | 0.70 |
| Liver | Tan | Sex | *Nme1* | 0.84 | 0.46 |
| Liver | Tan | Sex | *Psmc4* | 0.86 | 0.62 |
| Liver | Tan | Sex | *Psmd13* | 0.84 | 0.51 |
| Liver | Tan | Sex | *Psmd4* | 0.88 | 0.56 |
| Liver | Tan | Sex | *Psmd6* | 0.82 | 0.43 |
| Liver | Tan | Sex | *Rarres1* | 0.85 | 0.45 |
| Liver | Tan | Sex | *Rexo2* | 0.85 | 0.61 |
| Liver | Salmon | Age | *Ccl5* | 0.85 | 0.64 |
| Liver | Salmon | Age | *H2-Aa* | 0.83 | 0.54 |
| Liver | Salmon | Age | *H2-Eb1* | 0.82 | 0.60 |
| Liver | Salmon | Age | *Ntrk2* | 0.82 | 0.70 |
| Liver | Salmon | Age | *Slamf7* | 0.82 | 0.55 |
| Liver | Red | Sex | *Abcd1* | 0.81 | -0.61 |
| Liver | Red | Sex | *Acad9* | 0.85 | -0.69 |
| Liver | Red | Sex | *Acss3* | 0.84 | -0.81 |
| Liver | Red | Sex | *Agmo* | 0.80 | -0.64 |
| Liver | Red | Sex | *Akr1c20* | 0.83 | -0.72 |
| Liver | Red | Sex | *Akr1d1* | 0.84 | -0.80 |
| Liver | Red | Sex | *Aldh9a1* | 0.85 | -0.70 |
| Liver | Red | Sex | *Apol7a* | 0.85 | -0.60 |
| Liver | Red | Sex | *B630019A10Rik* | 0.85 | -0.64 |
| Liver | Red | Sex | *Bphl* | 0.84 | -0.62 |
| Liver | Red | Sex | *Car5a* | 0.88 | -0.53 |
| Liver | Red | Sex | *Ces1g* | 0.90 | -0.65 |
| Liver | Red | Sex | *Dhrs7* | 0.81 | -0.63 |
| Liver | Red | Sex | *Dpys* | 0.82 | -0.76 |
| Liver | Red | Sex | *Echs1* | 0.84 | -0.43 |
| Liver | Red | Sex | *Erg28* | 0.82 | -0.70 |
| Liver | Red | Sex | *Gm4756* | 0.81 | -0.47 |
| Liver | Red | Sex | *Gstt3* | 0.81 | -0.71 |
| Liver | Red | Sex | *Hadh* | 0.90 | -0.66 |
| Liver | Red | Sex | *Macrod1* | 0.85 | -0.49 |
| Liver | Red | Sex | *Mpc1* | 0.86 | -0.71 |
| Liver | Red | Sex | *Mpc1-ps* | 0.82 | -0.67 |
| Liver | Red | Sex | *Mup-ps16* | 0.83 | -0.54 |
| Liver | Red | Sex | *Ndrg2* | 0.82 | -0.62 |
| Liver | Red | Sex | *Pecr* | 0.81 | -0.60 |
| Liver | Red | Sex | *Plpp3* | 0.81 | -0.55 |
| Liver | Red | Sex | *Plscr2* | 0.81 | -0.67 |
| Liver | Red | Sex | *Pon1* | 0.84 | -0.79 |
| Liver | Red | Sex | *Serpinb1a* | 0.82 | -0.70 |
| Liver | Red | Sex | *Shmt1* | 0.85 | -0.75 |
| Liver | Red | Sex | *Slc47a1* | 0.83 | -0.73 |
| Liver | Red | Sex | *Tox* | 0.82 | -0.75 |
| Liver | Red | Sex | *Vnn3* | 0.83 | -0.70 |
| Liver | Darkturquoise | Age | *Cd19* | 0.80 | 0.54 |
| Liver | Darkturquoise | Age | *Cd79a* | 0.83 | 0.54 |
| Liver | Darkturquoise | Age | *Cd79b* | 0.82 | 0.49 |
| Liver | Darkturquoise | Age | *Ighg2b* | 0.89 | 0.52 |
| Liver | Darkturquoise | Age | *Ighg2c* | 0.81 | 0.53 |
| Liver | Darkturquoise | Age | *Ighm* | 0.85 | 0.64 |
| Liver | Darkturquoise | Age | *Ighv1-53* | 0.81 | 0.53 |
| Liver | Darkturquoise | Age | *Igkc* | 0.92 | 0.56 |
| Liver | Darkturquoise | Age | *Igkv3-2* | 0.85 | 0.45 |
| Liver | Darkturquoise | Age | *Igkv3-5* | 0.82 | 0.28 |
| Liver | Darkturquoise | Age | *Iglc1* | 0.81 | 0.40 |
| Liver | Darkturquoise | Age | *Iglc2* | 0.82 | 0.51 |
| Liver | Darkturquoise | Age | *Iglv1* | 0.80 | 0.39 |
| Liver | Darkturquoise | Age | *Jchain* | 0.93 | 0.57 |
| Liver | Darkturquoise | Age | *Mzb1* | 0.87 | 0.44 |
| Liver | Darkolivegreen | Sex | *Acss2* | 0.85 | -0.33 |
| Liver | Darkolivegreen | Sex | *Dhcr7* | 0.92 | -0.39 |
| Liver | Darkolivegreen | Sex | *Fdft1* | 0.92 | -0.55 |
| Liver | Darkolivegreen | Sex | *Hmgcs1* | 0.92 | -0.52 |
| Liver | Darkolivegreen | Sex | *Mmab* | 0.87 | -0.42 |
| Liver | Darkolivegreen | Sex | *Msmo1* | 0.87 | -0.53 |
| Liver | Darkolivegreen | Sex | *Mvd* | 0.88 | -0.52 |
| Liver | Darkolivegreen | Sex | *Mvk* | 0.81 | -0.46 |
| Liver | Darkolivegreen | Sex | *Pmvk* | 0.81 | -0.26 |
| Liver | Darkolivegreen | Sex | *Rdh11* | 0.90 | -0.47 |
| Liver | Darkolivegreen | Sex | *Spns2* | 0.80 | -0.52 |
| Liver | Darkgrey | Sex | *9130409I23Rik* | 0.80 | 0.64 |
| Liver | Darkgrey | Sex | *Arsa* | 0.91 | 0.87 |
| Liver | Darkgrey | Sex | *Chpt1* | 0.84 | 0.86 |
| Liver | Darkgrey | Sex | *Cidec* | 0.89 | 0.72 |
| Liver | Darkgrey | Sex | *Clstn3* | 0.91 | 0.83 |
| Liver | Darkgrey | Sex | *Cox19* | 0.84 | 0.75 |
| Liver | Darkgrey | Sex | *Cyp2u1* | 0.80 | 0.82 |
| Liver | Darkgrey | Sex | *Dpy19l3* | 0.82 | 0.77 |
| Liver | Darkgrey | Sex | *Fancl* | 0.83 | 0.71 |
| Liver | Darkgrey | Sex | *Fitm1* | 0.90 | 0.77 |
| Liver | Darkgrey | Sex | *Gpc1* | 0.87 | 0.68 |
| Liver | Darkgrey | Sex | *Gprc5b* | 0.84 | 0.62 |
| Liver | Darkgrey | Sex | *Nat8* | 0.87 | 0.86 |
| Liver | Darkgrey | Sex | *Ntrk1* | 0.82 | 0.63 |
| Liver | Darkgrey | Sex | *Olig1* | 0.89 | 0.75 |
| Liver | Darkgrey | Sex | *Osbpl3* | 0.86 | 0.57 |
| Liver | Darkgrey | Sex | *Pard3b* | 0.84 | 0.82 |
| Liver | Darkgrey | Sex | *Rassf3* | 0.85 | 0.79 |
| Liver | Darkgrey | Sex | *Snhg11* | 0.89 | 0.79 |
| Liver | Darkgrey | Sex | *Unc119* | 0.84 | 0.80 |
| Liver | Darkgrey | Sex | *Uox* | 0.81 | 0.70 |
| Liver | Darkgrey | Sex | *Zfp979* | 0.89 | 0.71 |
| Liver | Darkgrey | Sex | *Zfp982* | 0.85 | 0.74 |
| Liver | Darkgrey | Sex | *Zfp992* | 0.88 | 0.77 |
| Liver | Cyan | Sex | *Arcn1* | 0.81 | 0.62 |
| Liver | Cyan | Sex | *Copg1* | 0.84 | 0.64 |
| Liver | Cyan | Sex | *Creld2* | 0.84 | 0.34 |
| Liver | Cyan | Sex | *Hspa5* | 0.80 | 0.36 |
| Liver | Cyan | Sex | *Iars* | 0.82 | 0.51 |
| Liver | Cyan | Sex | *Manf* | 0.81 | 0.40 |
| Liver | Cyan | Sex | *Sdf2l1* | 0.81 | 0.46 |
| Liver | Cyan | Sex | *Sec22b* | 0.84 | 0.69 |
| Liver | Cyan | Sex | *Sec24d* | 0.82 | 0.55 |
| Liver | Cyan | Sex | *Sec61a1* | 0.81 | 0.67 |
| Liver | Cyan | Sex | *Serp1* | 0.83 | 0.73 |
| Liver | Cyan | Sex | *Slc33a1* | 0.80 | 0.57 |
| Liver | Cyan | Sex | *Ssr1* | 0.82 | 0.49 |
| Liver | Brown | Sex | *A1bg* | 0.84 | -0.88 |
| Liver | Brown | Sex | *Acot3* | 0.80 | -0.79 |
| Liver | Brown | Sex | *Aldh3b3* | 0.86 | -0.87 |
| Liver | Brown | Sex | *Arrdc4* | 0.80 | -0.81 |
| Liver | Brown | Sex | *Atp6v0d2* | 0.87 | -0.83 |
| Liver | Brown | Sex | *Chic1* | 0.82 | -0.86 |
| Liver | Brown | Sex | *Cux2* | 0.84 | -0.89 |
| Liver | Brown | Sex | *Cyp17a1* | 0.82 | -0.83 |
| Liver | Brown | Sex | *Cyp2a22* | 0.84 | -0.84 |
| Liver | Brown | Sex | *Cyp2a4* | 0.86 | -0.86 |
| Liver | Brown | Sex | *Cyp2b10* | 0.83 | -0.86 |
| Liver | Brown | Sex | *Cyp2b9* | 0.83 | -0.83 |
| Liver | Brown | Sex | *Cyp2c38* | 0.81 | -0.83 |
| Liver | Brown | Sex | *Cyp2c39* | 0.82 | -0.84 |
| Liver | Brown | Sex | *Cyp2c68* | 0.83 | -0.88 |
| Liver | Brown | Sex | *Cyp2c69* | 0.80 | -0.86 |
| Liver | Brown | Sex | *Cyp2g1* | 0.85 | -0.81 |
| Liver | Brown | Sex | *Cyp3a41a* | 0.81 | -0.85 |
| Liver | Brown | Sex | *Dqx1* | 0.86 | -0.76 |
| Liver | Brown | Sex | *Echdc3* | 0.86 | -0.78 |
| Liver | Brown | Sex | *Eci3* | 0.86 | -0.88 |
| Liver | Brown | Sex | *Esr1* | 0.80 | -0.78 |
| Liver | Brown | Sex | *Fmo1* | 0.84 | -0.88 |
| Liver | Brown | Sex | *Fmo2* | 0.83 | -0.83 |
| Liver | Brown | Sex | *Fmo3* | 0.84 | -0.87 |
| Liver | Brown | Sex | *Fmo4* | 0.81 | -0.77 |
| Liver | Brown | Sex | *Gm11695* | 0.86 | -0.82 |
| Liver | Brown | Sex | *Gm37273* | 0.81 | -0.84 |
| Liver | Brown | Sex | *Gm42375* | 0.87 | -0.85 |
| Liver | Brown | Sex | *Gm6135* | 0.84 | -0.83 |
| Liver | Brown | Sex | *Gypc* | 0.84 | -0.77 |
| Liver | Brown | Sex | *Hamp2* | 0.83 | -0.86 |
| Liver | Brown | Sex | *Hao2* | 0.89 | -0.87 |
| Liver | Brown | Sex | *Hexb* | 0.89 | -0.81 |
| Liver | Brown | Sex | *Hpd* | 0.83 | -0.82 |
| Liver | Brown | Sex | *Ildr2* | 0.82 | -0.76 |
| Liver | Brown | Sex | *Kat6b-ps2* | 0.81 | -0.84 |
| Liver | Brown | Sex | *Klhl13* | 0.83 | -0.78 |
| Liver | Brown | Sex | *Maob* | 0.85 | -0.83 |
| Liver | Brown | Sex | *Nipal1* | 0.87 | -0.84 |
| Liver | Brown | Sex | *Nt5e* | 0.81 | -0.88 |
| Liver | Brown | Sex | *Papss2* | 0.86 | -0.81 |
| Liver | Brown | Sex | *Prlr* | 0.84 | -0.83 |
| Liver | Brown | Sex | *Rdh16f2* | 0.90 | -0.87 |
| Liver | Brown | Sex | *Rtn4* | 0.83 | -0.82 |
| Liver | Brown | Sex | *Sall1* | 0.82 | -0.85 |
| Liver | Brown | Sex | *Sh2d4a* | 0.83 | -0.81 |
| Liver | Brown | Sex | *Slc16a5* | 0.81 | -0.76 |
| Liver | Brown | Sex | *Slc22a26* | 0.85 | -0.85 |
| Liver | Brown | Sex | *Slc22a27* | 0.85 | -0.83 |
| Liver | Brown | Sex | *Slco1a4* | 0.86 | -0.81 |
| Liver | Brown | Sex | *St3gal6* | 0.80 | -0.81 |
| Liver | Brown | Sex | *Sult1a1* | 0.83 | -0.80 |
| Liver | Brown | Sex | *Sult1d1* | 0.82 | -0.79 |
| Liver | Brown | Sex | *Sult2a-ps1* | 0.81 | -0.82 |
| Liver | Brown | Sex | *Sult2a-ps2* | 0.83 | -0.84 |
| Liver | Brown | Sex | *Sult2a1* | 0.85 | -0.89 |
| Liver | Brown | Sex | *Sult2a2* | 0.84 | -0.88 |
| Liver | Brown | Sex | *Sult2a7* | 0.83 | -0.81 |
| Liver | Brown | Sex | *Sult3a1* | 0.84 | -0.89 |
| Liver | Brown | Sex | *Tcn2* | 0.83 | -0.80 |
| Liver | Brown | Sex | *Tm6sf2* | 0.86 | -0.82 |
| Liver | Brown | Sex | *Tmem167-ps2* | 0.81 | -0.80 |
| Liver | Brown | Sex | *Tmem98* | 0.86 | -0.82 |
| Liver | Brown | Sex | *Uba7* | 0.83 | -0.75 |
| Liver | Brown | Sex | *Vldlr* | 0.80 | -0.77 |
| Liver | Brown | Sex | *Xist* | 0.83 | -0.88 |
| Liver | Brown | Sex | *Zbed4-ps2* | 0.81 | -0.85 |
| Liver | Blue | Sex | *1110038B12Rik* | 0.35 | 0.31 |
| Liver | Blue | Sex | *1810013L24Rik* | 0.55 | 0.61 |
| Liver | Blue | Sex | *5730455P16Rik* | 0.55 | 0.55 |
| Liver | Blue | Sex | *9130401M01Rik* | 0.38 | 0.38 |
| Liver | Blue | Sex | *AI182371* | 0.40 | 0.19 |
| Liver | Blue | Sex | *AI463229* | 0.80 | 0.74 |
| Liver | Blue | Sex | *Abcb10* | 0.78 | 0.78 |
| Liver | Blue | Sex | *Abcg2* | 0.88 | 0.83 |
| Liver | Blue | Sex | *Acox1* | 0.75 | 0.67 |
| Liver | Blue | Sex | *Actr1b* | 0.84 | 0.72 |
| Liver | Blue | Sex | *Ankrd27* | 0.49 | 0.42 |
| Liver | Blue | Sex | *Ankrd52* | 0.45 | 0.39 |
| Liver | Blue | Sex | *Arfgap3* | 0.51 | 0.61 |
| Liver | Blue | Sex | *Arl2bp* | 0.31 | 0.22 |
| Liver | Blue | Sex | *Arsg* | 0.50 | 0.47 |
| Liver | Blue | Sex | *Asap3* | 0.66 | 0.60 |
| Liver | Blue | Sex | *Asb6* | 0.54 | 0.49 |
| Liver | Blue | Sex | *Bcap31* | 0.41 | 0.19 |
| Liver | Blue | Sex | *Bpnt1* | 0.66 | 0.48 |
| Liver | Blue | Sex | *C6* | 0.91 | 0.87 |
| Liver | Blue | Sex | *C730027H18Rik* | 0.73 | 0.77 |
| Liver | Blue | Sex | *Cadm4* | 0.54 | 0.56 |
| Liver | Blue | Sex | *Caml* | 0.36 | 0.36 |
| Liver | Blue | Sex | *Capn7* | 0.57 | 0.40 |
| Liver | Blue | Sex | *Capza2* | 0.64 | 0.56 |
| Liver | Blue | Sex | *Cbfa2t2* | 0.42 | 0.29 |
| Liver | Blue | Sex | *Cdc34* | 0.37 | 0.43 |
| Liver | Blue | Sex | *Cdh15* | 0.39 | 0.44 |
| Liver | Blue | Sex | *Cdhr5* | 0.64 | 0.64 |
| Liver | Blue | Sex | *Cdipt* | 0.73 | 0.55 |
| Liver | Blue | Sex | *Ces2a* | 0.68 | 0.67 |
| Liver | Blue | Sex | *Ces2b* | 0.36 | 0.37 |
| Liver | Blue | Sex | *Chmp1b* | 0.66 | 0.57 |
| Liver | Blue | Sex | *Chrd* | 0.36 | 0.30 |
| Liver | Blue | Sex | *Cmtm6* | 0.82 | 0.67 |
| Liver | Blue | Sex | *Cnot7* | 0.47 | 0.40 |
| Liver | Blue | Sex | *Csad* | 0.69 | 0.69 |
| Liver | Blue | Sex | *Csnk1d* | 0.70 | 0.53 |
| Liver | Blue | Sex | *Ctr9* | 0.60 | 0.47 |
| Liver | Blue | Sex | *Cul2* | 0.79 | 0.59 |
| Liver | Blue | Sex | *Cxadr* | 0.64 | 0.71 |
| Liver | Blue | Sex | *Cyp4a12a* | 0.85 | 0.87 |
| Liver | Blue | Sex | *Dcakd* | 0.62 | 0.46 |
| Liver | Blue | Sex | *Dnase2b* | 0.74 | 0.83 |
| Liver | Blue | Sex | *Dnd1* | 0.37 | 0.28 |
| Liver | Blue | Sex | *Dus1l* | 0.77 | 0.59 |
| Liver | Blue | Sex | *Dusp8* | 0.60 | 0.59 |
| Liver | Blue | Sex | *Dync1h1* | 0.55 | 0.50 |
| Liver | Blue | Sex | *Dynll2* | 0.40 | 0.45 |
| Liver | Blue | Sex | *Eed* | 0.50 | 0.50 |
| Liver | Blue | Sex | *Eif5* | 0.76 | 0.59 |
| Liver | Blue | Sex | *Elovl2* | 0.53 | 0.42 |
| Liver | Blue | Sex | *Elovl3* | 0.84 | 0.83 |
| Liver | Blue | Sex | *Emc6* | 0.60 | 0.53 |
| Liver | Blue | Sex | *Ephx1* | 0.75 | 0.72 |
| Liver | Blue | Sex | *Ephx2* | 0.80 | 0.72 |
| Liver | Blue | Sex | *Eri1* | 0.54 | 0.52 |
| Liver | Blue | Sex | *Etfbkmt* | 0.88 | 0.83 |
| Liver | Blue | Sex | *Fcor* | 0.26 | 0.35 |
| Liver | Blue | Sex | *Fech* | 0.65 | 0.61 |
| Liver | Blue | Sex | *Ftl2-ps* | 0.41 | 0.32 |
| Liver | Blue | Sex | *Fyttd1* | 0.60 | 0.41 |
| Liver | Blue | Sex | *Galnt1* | 0.41 | 0.40 |
| Liver | Blue | Sex | *Garem1* | 0.43 | 0.35 |
| Liver | Blue | Sex | *Gas2l1* | 0.76 | 0.72 |
| Liver | Blue | Sex | *Gm11963* | 0.38 | 0.36 |
| Liver | Blue | Sex | *Gm15883* | 0.54 | 0.49 |
| Liver | Blue | Sex | *Gm31036* | 0.49 | 0.38 |
| Liver | Blue | Sex | *Gm34654* | 0.52 | 0.64 |
| Liver | Blue | Sex | *Gm40787* | 0.74 | 0.85 |
| Liver | Blue | Sex | *Gm42688* | 0.39 | 0.42 |
| Liver | Blue | Sex | *Gm44507* | 0.46 | 0.50 |
| Liver | Blue | Sex | *Gm45727* | 0.36 | 0.36 |
| Liver | Blue | Sex | *Gm49338* | 0.52 | 0.43 |
| Liver | Blue | Sex | *Gpat4* | 0.79 | 0.75 |
| Liver | Blue | Sex | *Gpr39* | 0.44 | 0.42 |
| Liver | Blue | Sex | *Gprin3* | 0.39 | 0.38 |
| Liver | Blue | Sex | *Gpsm2* | 0.60 | 0.53 |
| Liver | Blue | Sex | *Gspt1* | 0.69 | 0.53 |
| Liver | Blue | Sex | *Gsr* | 0.83 | 0.77 |
| Liver | Blue | Sex | *Gt(ROSA)26Sor* | 0.55 | 0.43 |
| Liver | Blue | Sex | *Gtpbp4-ps1* | 0.77 | 0.84 |
| Liver | Blue | Sex | *Hras* | 0.38 | 0.31 |
| Liver | Blue | Sex | *Hsd17b12* | 0.71 | 0.67 |
| Liver | Blue | Sex | *Igsf5* | 0.75 | 0.65 |
| Liver | Blue | Sex | *Ikbke* | 0.56 | 0.59 |
| Liver | Blue | Sex | *Inhbc* | 0.70 | 0.56 |
| Liver | Blue | Sex | *Inpp4a* | 0.53 | 0.43 |
| Liver | Blue | Sex | *Ints9* | 0.34 | 0.19 |
| Liver | Blue | Sex | *Kdm4b* | 0.55 | 0.50 |
| Liver | Blue | Sex | *Klk1b4* | 0.56 | 0.68 |
| Liver | Blue | Sex | *Lcp1* | 0.50 | 0.47 |
| Liver | Blue | Sex | *Ldah* | 0.82 | 0.78 |
| Liver | Blue | Sex | *Lmo4* | 0.46 | 0.46 |
| Liver | Blue | Sex | *Lrrc3* | 0.44 | 0.27 |
| Liver | Blue | Sex | *Lrrc42* | 0.58 | 0.46 |
| Liver | Blue | Sex | *Lurap1l* | 0.71 | 0.56 |
| Liver | Blue | Sex | *Map1lc3b* | 0.54 | 0.32 |
| Liver | Blue | Sex | *Mbnl2* | 0.41 | 0.44 |
| Liver | Blue | Sex | *Mcm2* | 0.36 | 0.41 |
| Liver | Blue | Sex | *Mcph1* | 0.40 | 0.48 |
| Liver | Blue | Sex | *Mctp2* | 0.71 | 0.75 |
| Liver | Blue | Sex | *Mecr* | 0.49 | 0.32 |
| Liver | Blue | Sex | *Med26* | 0.38 | 0.32 |
| Liver | Blue | Sex | *Mfsd11* | 0.38 | 0.35 |
| Liver | Blue | Sex | *Mgrn1* | 0.70 | 0.67 |
| Liver | Blue | Sex | *Msrb3* | 0.41 | 0.41 |
| Liver | Blue | Sex | *Mta2* | 0.65 | 0.60 |
| Liver | Blue | Sex | *Mup6* | 0.41 | 0.48 |
| Liver | Blue | Sex | *Mup9* | 0.65 | 0.54 |
| Liver | Blue | Sex | *Myo1c* | 0.42 | 0.47 |
| Liver | Blue | Sex | *Myo6* | 0.72 | 0.66 |
| Liver | Blue | Sex | *Mzt1* | 0.36 | 0.42 |
| Liver | Blue | Sex | *Nfyc* | 0.36 | 0.23 |
| Liver | Blue | Sex | *Npepps* | 0.57 | 0.49 |
| Liver | Blue | Sex | *Ogdh* | 0.76 | 0.66 |
| Liver | Blue | Sex | *Ola1* | 0.44 | 0.43 |
| Liver | Blue | Sex | *Pafah1b3* | 0.51 | 0.64 |
| Liver | Blue | Sex | *Paip2* | 0.51 | 0.35 |
| Liver | Blue | Sex | *Pak1ip1* | 0.64 | 0.60 |
| Liver | Blue | Sex | *Paqr7* | 0.57 | 0.64 |
| Liver | Blue | Sex | *Pdilt* | 0.58 | 0.53 |
| Liver | Blue | Sex | *Pgs1* | 0.56 | 0.53 |
| Liver | Blue | Sex | *Phf20l1* | 0.69 | 0.70 |
| Liver | Blue | Sex | *Phlda2* | 0.42 | 0.41 |
| Liver | Blue | Sex | *Phyh* | 0.84 | 0.82 |
| Liver | Blue | Sex | *Pip5k1a* | 0.38 | 0.36 |
| Liver | Blue | Sex | *Pisd* | 0.61 | 0.43 |
| Liver | Blue | Sex | *Plekha1* | 0.68 | 0.70 |
| Liver | Blue | Sex | *Plk2* | 0.62 | 0.67 |
| Liver | Blue | Sex | *Ppp2ca* | 0.49 | 0.40 |
| Liver | Blue | Sex | *Ppp2r5c* | 0.59 | 0.38 |
| Liver | Blue | Sex | *Psmd10* | 0.45 | 0.36 |
| Liver | Blue | Sex | *Psmd14* | 0.63 | 0.56 |
| Liver | Blue | Sex | *Psme3* | 0.76 | 0.60 |
| Liver | Blue | Sex | *Ptma* | 0.51 | 0.56 |
| Liver | Blue | Sex | *Pum2* | 0.31 | 0.30 |
| Liver | Blue | Sex | *Rap2a* | 0.53 | 0.50 |
| Liver | Blue | Sex | *Rest* | 0.47 | 0.36 |
| Liver | Blue | Sex | *Rpf1* | 0.37 | 0.39 |
| Liver | Blue | Sex | *Rpl26-ps6* | 0.39 | 0.34 |
| Liver | Blue | Sex | *Sae1* | 0.42 | 0.38 |
| Liver | Blue | Sex | *Sall2* | 0.30 | 0.32 |
| Liver | Blue | Sex | *Samd1* | 0.49 | 0.44 |
| Liver | Blue | Sex | *Sars* | 0.74 | 0.62 |
| Liver | Blue | Sex | *Scp2* | 0.91 | 0.87 |
| Liver | Blue | Sex | *Selenbp2* | 0.77 | 0.67 |
| Liver | Blue | Sex | *Sephs1* | 0.74 | 0.59 |
| Liver | Blue | Sex | *Serinc1* | 0.66 | 0.44 |
| Liver | Blue | Sex | *Serpina11* | 0.67 | 0.53 |
| Liver | Blue | Sex | *Serpina4-ps1* | 0.77 | 0.71 |
| Liver | Blue | Sex | *Serpine2* | 0.73 | 0.77 |
| Liver | Blue | Sex | *Sfr1* | 0.54 | 0.46 |
| Liver | Blue | Sex | *Sgpp1* | 0.66 | 0.72 |
| Liver | Blue | Sex | *Slc35b3* | 0.43 | 0.25 |
| Liver | Blue | Sex | *Slc35e3* | 0.80 | 0.80 |
| Liver | Blue | Sex | *Smpd1* | 0.75 | 0.52 |
| Liver | Blue | Sex | *Snhg3* | 0.34 | 0.33 |
| Liver | Blue | Sex | *Socs4* | 0.51 | 0.39 |
| Liver | Blue | Sex | *Sort1* | 0.59 | 0.56 |
| Liver | Blue | Sex | *Spaca6* | 0.46 | 0.47 |
| Liver | Blue | Sex | *Stat6* | 0.65 | 0.47 |
| Liver | Blue | Sex | *Susd4* | 0.82 | 0.83 |
| Liver | Blue | Sex | *Tesk2* | 0.62 | 0.44 |
| Liver | Blue | Sex | *Tex30* | 0.32 | 0.25 |
| Liver | Blue | Sex | *Thoc6* | 0.48 | 0.39 |
| Liver | Blue | Sex | *Thtpa* | 0.61 | 0.59 |
| Liver | Blue | Sex | *Thumpd3* | 0.39 | 0.30 |
| Liver | Blue | Sex | *Tm9sf1* | 0.57 | 0.41 |
| Liver | Blue | Sex | *Tmem125* | 0.60 | 0.55 |
| Liver | Blue | Sex | *Tmigd1* | 0.71 | 0.79 |
| Liver | Blue | Sex | *Topors* | 0.60 | 0.45 |
| Liver | Blue | Sex | *Tpmt* | 0.77 | 0.79 |
| Liver | Blue | Sex | *Tpp1* | 0.50 | 0.33 |
| Liver | Blue | Sex | *Traf2* | 0.41 | 0.32 |
| Liver | Blue | Sex | *Tram1* | 0.81 | 0.74 |
| Liver | Blue | Sex | *Tsg101* | 0.78 | 0.59 |
| Liver | Blue | Sex | *Ttc33* | 0.30 | 0.19 |
| Liver | Blue | Sex | *Uap1l1* | 0.57 | 0.59 |
| Liver | Blue | Sex | *Ubac2* | 0.54 | 0.37 |
| Liver | Blue | Sex | *Ufm1* | 0.36 | 0.40 |
| Liver | Blue | Sex | *Ugdh* | 0.81 | 0.80 |
| Liver | Blue | Sex | *Ugt2b38* | 0.88 | 0.75 |
| Liver | Blue | Sex | *Ugt2b5* | 0.85 | 0.70 |
| Liver | Blue | Sex | *Uri1* | 0.70 | 0.66 |
| Liver | Blue | Sex | *Vkorc1l1* | 0.43 | 0.49 |
| Liver | Blue | Sex | *Vrk3* | 0.56 | 0.45 |
| Liver | Blue | Sex | *Yipf3* | 0.31 | 0.13 |
| Liver | Blue | Sex | *Ythdf1* | 0.50 | 0.44 |
| Liver | Blue | Sex | *Zbtb42* | 0.71 | 0.60 |
| Liver | Blue | Sex | *Zc3h12a* | 0.33 | 0.29 |
| Liver | Blue | Sex | *Zc3h14* | 0.43 | 0.19 |
| Liver | Blue | Sex | *Zdhhc16* | 0.50 | 0.40 |
| Liver | Blue | Sex | *Zfp125* | 0.68 | 0.75 |
| Liver | Blue | Sex | *Zfp706* | 0.81 | 0.65 |
| Liver | Blue | Sex | *Zfpm1* | 0.60 | 0.64 |
| Liver | Blue | Sex | *Zfyve1* | 0.36 | 0.22 |
| Liver | Blue | Sex | *Zkscan3* | 0.62 | 0.56 |
| Liver | Blue | Sex | *Zranb2* | 0.48 | 0.31 |
| Liver | Blue | Sex | *Zyg11a* | 0.65 | 0.54 |
| Muscle | Magenta | Age | *Cpe* | 0.80 | 0.69 |
| Muscle | Magenta | Age | *Eif3e* | 0.81 | 0.59 |
| Muscle | Magenta | Age | *Itgb5* | 0.80 | 0.63 |
| Muscle | Magenta | Age | *Kcmf1* | 0.86 | 0.44 |
| Muscle | Magenta | Age | *Mib1* | 0.81 | 0.71 |
| Muscle | Magenta | Age | *Plekhb1* | 0.81 | 0.81 |
| Muscle | Magenta | Age | *Rab2a* | 0.88 | 0.72 |
| Muscle | Magenta | Age | *Rasd2* | 0.85 | 0.82 |
| Muscle | Magenta | Age | *Setd3* | 0.82 | 0.52 |
| Muscle | Brown | Age | *Adamts2* | 0.90 | -0.71 |
| Muscle | Brown | Age | *Angptl1* | 0.83 | -0.73 |
| Muscle | Brown | Age | *Antxr2* | 0.80 | -0.53 |
| Muscle | Brown | Age | *Anxa2* | 0.83 | -0.67 |
| Muscle | Brown | Age | *Axl* | 0.87 | -0.66 |
| Muscle | Brown | Age | *C3* | 0.81 | -0.59 |
| Muscle | Brown | Age | *Cd34* | 0.88 | -0.65 |
| Muscle | Brown | Age | *Clec3b* | 0.82 | -0.59 |
| Muscle | Brown | Age | *Col1a1* | 0.86 | -0.85 |
| Muscle | Brown | Age | *Col1a2* | 0.85 | -0.84 |
| Muscle | Brown | Age | *Col3a1* | 0.81 | -0.79 |
| Muscle | Brown | Age | *Col5a1* | 0.81 | -0.77 |
| Muscle | Brown | Age | *Col5a2* | 0.81 | -0.79 |
| Muscle | Brown | Age | *Col6a1* | 0.92 | -0.75 |
| Muscle | Brown | Age | *Col6a2* | 0.89 | -0.73 |
| Muscle | Brown | Age | *Col6a3* | 0.89 | -0.71 |
| Muscle | Brown | Age | *Ddr2* | 0.81 | -0.56 |
| Muscle | Brown | Age | *Dok2* | 0.81 | -0.57 |
| Muscle | Brown | Age | *Dstn* | 0.81 | -0.77 |
| Muscle | Brown | Age | *Fbn1* | 0.80 | -0.71 |
| Muscle | Brown | Age | *Fn1* | 0.86 | -0.83 |
| Muscle | Brown | Age | *Fndc1* | 0.93 | -0.73 |
| Muscle | Brown | Age | *Fstl1* | 0.82 | -0.76 |
| Muscle | Brown | Age | *Igfbp6* | 0.86 | -0.68 |
| Muscle | Brown | Age | *Islr* | 0.80 | -0.54 |
| Muscle | Brown | Age | *Itgbl1* | 0.87 | -0.71 |
| Muscle | Brown | Age | *Lpar1* | 0.83 | -0.66 |
| Muscle | Brown | Age | *Lrp1* | 0.89 | -0.63 |
| Muscle | Brown | Age | *Metrnl* | 0.81 | -0.56 |
| Muscle | Brown | Age | *Mfap5* | 0.84 | -0.73 |
| Muscle | Brown | Age | *Mmp2* | 0.83 | -0.69 |
| Muscle | Brown | Age | *Mrc1* | 0.87 | -0.58 |
| Muscle | Brown | Age | *Mrc2* | 0.81 | -0.67 |
| Muscle | Brown | Age | *Ndn* | 0.84 | -0.65 |
| Muscle | Brown | Age | *Nid1* | 0.83 | -0.73 |
| Muscle | Brown | Age | *Olfml2b* | 0.81 | -0.67 |
| Muscle | Brown | Age | *Olfml3* | 0.87 | -0.67 |
| Muscle | Brown | Age | *P3h3* | 0.81 | -0.56 |
| Muscle | Brown | Age | *Pcolce* | 0.92 | -0.78 |
| Muscle | Brown | Age | *Pcolce2* | 0.81 | -0.59 |
| Muscle | Brown | Age | *Pi16* | 0.86 | -0.75 |
| Muscle | Brown | Age | *Pltp* | 0.81 | -0.56 |
| Muscle | Brown | Age | *Plxdc2* | 0.81 | -0.62 |
| Muscle | Brown | Age | *Rcn1* | 0.81 | -0.58 |
| Muscle | Brown | Age | *Rcn3* | 0.85 | -0.67 |
| Muscle | Brown | Age | *Rnase4* | 0.83 | -0.57 |
| Muscle | Brown | Age | *Serpinf1* | 0.91 | -0.75 |
| Muscle | Brown | Age | *Serping1* | 0.83 | -0.57 |
| Muscle | Brown | Age | *Sod3* | 0.84 | -0.70 |
| Muscle | Brown | Age | *Sparc* | 0.85 | -0.77 |
| Muscle | Brown | Age | *Ssc5d* | 0.83 | -0.70 |
| Muscle | Brown | Age | *Tgfbi* | 0.84 | -0.61 |
| Muscle | Brown | Age | *Timp2* | 0.84 | -0.61 |
| Muscle | Red | Sex | *1700001O22Rik* | 0.85 | 0.86 |
| Muscle | Red | Sex | *Amd1* | 0.86 | 0.88 |
| Muscle | Red | Sex | *Amd2* | 0.81 | 0.83 |
| Muscle | Red | Sex | *Anxa7* | 0.83 | 0.78 |
| Muscle | Red | Sex | *C7* | 0.81 | 0.84 |
| Muscle | Red | Sex | *Cbr2* | 0.85 | 0.87 |
| Muscle | Red | Sex | *Cdk19* | 0.80 | 0.82 |
| Muscle | Red | Sex | *Ddx3y* | 0.83 | 0.86 |
| Muscle | Red | Sex | *Eif2s3y* | 0.81 | 0.85 |
| Muscle | Red | Sex | *Eif4ebp1* | 0.84 | 0.80 |
| Muscle | Red | Sex | *Fam131a* | 0.84 | 0.84 |
| Muscle | Red | Sex | *Gm10032* | 0.82 | 0.76 |
| Muscle | Red | Sex | *Gm12240* | 0.84 | 0.80 |
| Muscle | Red | Sex | *Gm8734* | 0.84 | 0.82 |
| Muscle | Red | Sex | *Grina* | 0.80 | 0.70 |
| Muscle | Red | Sex | *Hipk2* | 0.87 | 0.82 |
| Muscle | Red | Sex | *Htra4* | 0.82 | 0.81 |
| Muscle | Red | Sex | *Irx3os* | 0.82 | 0.85 |
| Muscle | Red | Sex | *Kdm5d* | 0.85 | 0.90 |
| Muscle | Red | Sex | *Ldlr* | 0.82 | 0.83 |
| Muscle | Red | Sex | *Mgst1* | 0.84 | 0.82 |
| Muscle | Red | Sex | *Musk* | 0.83 | 0.76 |
| Muscle | Red | Sex | *Nek6* | 0.82 | 0.73 |
| Muscle | Red | Sex | *Ppp1r14b* | 0.86 | 0.85 |
| Muscle | Red | Sex | *Psmd8* | 0.88 | 0.86 |
| Muscle | Red | Sex | *Samd10* | 0.81 | 0.71 |
| Muscle | Red | Sex | *Sbk2* | 0.80 | 0.79 |
| Muscle | Red | Sex | *Serpinb6a* | 0.87 | 0.83 |
| Muscle | Red | Sex | *Slc15a5* | 0.84 | 0.81 |
| Muscle | Red | Sex | *Slc2a3* | 0.84 | 0.79 |
| Muscle | Red | Sex | *Slc30a2* | 0.87 | 0.88 |
| Muscle | Red | Sex | *Spns2* | 0.88 | 0.83 |
| Muscle | Red | Sex | *Stab2* | 0.89 | 0.81 |
| Muscle | Red | Sex | *Sub1* | 0.83 | 0.81 |
| Muscle | Red | Sex | *Tfcp2l1* | 0.86 | 0.82 |
| Muscle | Red | Sex | *Tmem37* | 0.80 | 0.74 |
| Muscle | Red | Sex | *Ubl7* | 0.84 | 0.74 |
| Muscle | Red | Sex | *Uty* | 0.82 | 0.86 |
| Muscle | Red | Sex | *Vldlr* | 0.92 | 0.86 |
| Muscle | Red | Sex | *Wsb2* | 0.85 | 0.73 |
| Muscle | Purple | Sex | *Acox1* | 0.87 | -0.71 |
| Muscle | Purple | Sex | *Aldh2* | 0.82 | -0.83 |
| Muscle | Purple | Sex | *Ces1d* | 0.80 | -0.72 |
| Muscle | Purple | Sex | *Egf* | 0.80 | -0.71 |
| Muscle | Purple | Sex | *Gcdh* | 0.86 | -0.56 |
| Muscle | Purple | Sex | *Gstm2* | 0.85 | -0.71 |
| Muscle | Purple | Sex | *Mybpc1* | 0.80 | -0.63 |
| Muscle | Purple | Sex | *Ppp1r1a* | 0.87 | -0.62 |
| Muscle | Purple | Sex | *Selenbp1* | 0.84 | -0.72 |
| Muscle | Purple | Sex | *Suclg2* | 0.85 | -0.63 |
| Muscle | Greenyellow | Sex | *Cyfip2* | 0.81 | -0.86 |
| Muscle | Greenyellow | Sex | *Ddx3x* | 0.84 | -0.86 |
| Muscle | Greenyellow | Sex | *Eif2s3x* | 0.83 | -0.85 |
| Muscle | Greenyellow | Sex | *Gm15337* | 0.84 | -0.87 |
| Muscle | Greenyellow | Sex | *Gm47708* | 0.88 | -0.86 |
| Muscle | Greenyellow | Sex | *Homer2* | 0.84 | -0.75 |
| Muscle | Greenyellow | Sex | *Lamb2* | 0.82 | -0.82 |
| Muscle | Greenyellow | Sex | *Mybph* | 0.80 | -0.80 |
| Muscle | Greenyellow | Sex | *Neu2* | 0.82 | -0.82 |
| Muscle | Greenyellow | Sex | *Padi2* | 0.84 | -0.80 |
| Muscle | Greenyellow | Sex | *Xist* | 0.81 | -0.86 |
| Muscle | Blue | Sex | *0610012G03Rik* | 0.81 | 0.47 |
| Muscle | Blue | Sex | *Acot13* | 0.85 | 0.42 |
| Muscle | Blue | Sex | *Atp5c1* | 0.82 | 0.42 |
| Muscle | Blue | Sex | *Atp5d* | 0.80 | 0.43 |
| Muscle | Blue | Sex | *Atp5e* | 0.94 | 0.44 |
| Muscle | Blue | Sex | *Atp5g1* | 0.82 | 0.46 |
| Muscle | Blue | Sex | *Atp5g2* | 0.80 | 0.42 |
| Muscle | Blue | Sex | *Atp5g3* | 0.86 | 0.42 |
| Muscle | Blue | Sex | *Atp5h* | 0.86 | 0.38 |
| Muscle | Blue | Sex | *Atp5j* | 0.88 | 0.35 |
| Muscle | Blue | Sex | *Atp5j2* | 0.93 | 0.48 |
| Muscle | Blue | Sex | *Atp5l* | 0.90 | 0.60 |
| Muscle | Blue | Sex | *Atp5mpl* | 0.87 | 0.54 |
| Muscle | Blue | Sex | *Atp5o* | 0.89 | 0.37 |
| Muscle | Blue | Sex | *Chchd1* | 0.83 | 0.27 |
| Muscle | Blue | Sex | *Chchd10* | 0.89 | 0.69 |
| Muscle | Blue | Sex | *Cisd1* | 0.82 | 0.27 |
| Muscle | Blue | Sex | *Cox14* | 0.82 | 0.58 |
| Muscle | Blue | Sex | *Cox4i1* | 0.90 | 0.46 |
| Muscle | Blue | Sex | *Cox5a* | 0.84 | 0.41 |
| Muscle | Blue | Sex | *Cox5b* | 0.86 | 0.42 |
| Muscle | Blue | Sex | *Cox6a2* | 0.88 | 0.30 |
| Muscle | Blue | Sex | *Cox6b1* | 0.91 | 0.44 |
| Muscle | Blue | Sex | *Cox6c* | 0.93 | 0.48 |
| Muscle | Blue | Sex | *Cox7a1* | 0.85 | 0.53 |
| Muscle | Blue | Sex | *Cox7a2* | 0.91 | 0.53 |
| Muscle | Blue | Sex | *Cox7c* | 0.89 | 0.51 |
| Muscle | Blue | Sex | *Cox8a* | 0.87 | 0.49 |
| Muscle | Blue | Sex | *Cox8b* | 0.92 | 0.57 |
| Muscle | Blue | Sex | *D8Ertd738e* | 0.82 | 0.28 |
| Muscle | Blue | Sex | *Elob* | 0.90 | 0.37 |
| Muscle | Blue | Sex | *Fau* | 0.81 | 0.50 |
| Muscle | Blue | Sex | *Fmc1* | 0.84 | 0.48 |
| Muscle | Blue | Sex | *Fxyd1* | 0.83 | 0.24 |
| Muscle | Blue | Sex | *Gng5* | 0.91 | 0.39 |
| Muscle | Blue | Sex | *Higd1a* | 0.87 | 0.44 |
| Muscle | Blue | Sex | *Hint2* | 0.81 | 0.29 |
| Muscle | Blue | Sex | *Krtcap2* | 0.86 | 0.38 |
| Muscle | Blue | Sex | *Lamtor2* | 0.81 | 0.37 |
| Muscle | Blue | Sex | *Lars2* | 0.82 | 0.43 |
| Muscle | Blue | Sex | *Mpc2* | 0.86 | 0.50 |
| Muscle | Blue | Sex | *Mrpl14* | 0.87 | 0.50 |
| Muscle | Blue | Sex | *Mrpl23* | 0.80 | 0.39 |
| Muscle | Blue | Sex | *Mrpl33* | 0.81 | 0.37 |
| Muscle | Blue | Sex | *Mrpl51* | 0.81 | 0.58 |
| Muscle | Blue | Sex | *Mrps18a* | 0.87 | 0.52 |
| Muscle | Blue | Sex | *Mrps21* | 0.87 | 0.30 |
| Muscle | Blue | Sex | *Mrps24* | 0.81 | 0.40 |
| Muscle | Blue | Sex | *Mylpf* | 0.80 | 0.48 |
| Muscle | Blue | Sex | *Ndufa11* | 0.90 | 0.36 |
| Muscle | Blue | Sex | *Ndufa12* | 0.89 | 0.53 |
| Muscle | Blue | Sex | *Ndufa13* | 0.88 | 0.36 |
| Muscle | Blue | Sex | *Ndufa2* | 0.93 | 0.46 |
| Muscle | Blue | Sex | *Ndufa4* | 0.82 | 0.59 |
| Muscle | Blue | Sex | *Ndufa5* | 0.88 | 0.45 |
| Muscle | Blue | Sex | *Ndufa6* | 0.93 | 0.47 |
| Muscle | Blue | Sex | *Ndufa7* | 0.92 | 0.50 |
| Muscle | Blue | Sex | *Ndufa8* | 0.83 | 0.52 |
| Muscle | Blue | Sex | *Ndufb10* | 0.88 | 0.44 |
| Muscle | Blue | Sex | *Ndufb11* | 0.90 | 0.34 |
| Muscle | Blue | Sex | *Ndufb2* | 0.86 | 0.49 |
| Muscle | Blue | Sex | *Ndufb4* | 0.88 | 0.43 |
| Muscle | Blue | Sex | *Ndufb5* | 0.85 | 0.27 |
| Muscle | Blue | Sex | *Ndufb6* | 0.90 | 0.45 |
| Muscle | Blue | Sex | *Ndufb7* | 0.89 | 0.38 |
| Muscle | Blue | Sex | *Ndufb8* | 0.91 | 0.49 |
| Muscle | Blue | Sex | *Ndufb9* | 0.93 | 0.38 |
| Muscle | Blue | Sex | *Ndufc1* | 0.89 | 0.44 |
| Muscle | Blue | Sex | *Ndufc2* | 0.84 | 0.46 |
| Muscle | Blue | Sex | *Ndufs5* | 0.86 | 0.39 |
| Muscle | Blue | Sex | *Ndufs6* | 0.94 | 0.43 |
| Muscle | Blue | Sex | *Ndufs7* | 0.89 | 0.46 |
| Muscle | Blue | Sex | *Ndufv3* | 0.90 | 0.47 |
| Muscle | Blue | Sex | *Nedd8* | 0.80 | 0.40 |
| Muscle | Blue | Sex | *Nenf* | 0.80 | 0.40 |
| Muscle | Blue | Sex | *Nop10* | 0.81 | 0.35 |
| Muscle | Blue | Sex | *Pam16* | 0.81 | 0.46 |
| Muscle | Blue | Sex | *Pdcd5* | 0.84 | 0.42 |
| Muscle | Blue | Sex | *Pfdn5* | 0.84 | 0.50 |
| Muscle | Blue | Sex | *Polr2l* | 0.82 | 0.44 |
| Muscle | Blue | Sex | *Psmb1* | 0.82 | 0.51 |
| Muscle | Blue | Sex | *Psmb3* | 0.81 | 0.55 |
| Muscle | Blue | Sex | *Psmb4* | 0.84 | 0.44 |
| Muscle | Blue | Sex | *Psmb5* | 0.82 | 0.40 |
| Muscle | Blue | Sex | *Pvalb* | 0.80 | 0.58 |
| Muscle | Blue | Sex | *Romo1* | 0.91 | 0.45 |
| Muscle | Blue | Sex | *Rpl10a* | 0.81 | 0.46 |
| Muscle | Blue | Sex | *Rpl11* | 0.86 | 0.45 |
| Muscle | Blue | Sex | *Rpl12* | 0.82 | 0.47 |
| Muscle | Blue | Sex | *Rpl13* | 0.80 | 0.30 |
| Muscle | Blue | Sex | *Rpl14* | 0.82 | 0.28 |
| Muscle | Blue | Sex | *Rpl18* | 0.82 | 0.28 |
| Muscle | Blue | Sex | *Rpl18a* | 0.90 | 0.42 |
| Muscle | Blue | Sex | *Rpl19* | 0.83 | 0.37 |
| Muscle | Blue | Sex | *Rpl21* | 0.91 | 0.49 |
| Muscle | Blue | Sex | *Rpl23* | 0.89 | 0.47 |
| Muscle | Blue | Sex | *Rpl26* | 0.86 | 0.45 |
| Muscle | Blue | Sex | *Rpl27* | 0.83 | 0.30 |
| Muscle | Blue | Sex | *Rpl28* | 0.88 | 0.46 |
| Muscle | Blue | Sex | *Rpl30* | 0.81 | 0.49 |
| Muscle | Blue | Sex | *Rpl31* | 0.89 | 0.46 |
| Muscle | Blue | Sex | *Rpl32* | 0.87 | 0.45 |
| Muscle | Blue | Sex | *Rpl34* | 0.84 | 0.53 |
| Muscle | Blue | Sex | *Rpl35a* | 0.80 | 0.53 |
| Muscle | Blue | Sex | *Rpl36* | 0.93 | 0.45 |
| Muscle | Blue | Sex | *Rpl36a* | 0.86 | 0.35 |
| Muscle | Blue | Sex | *Rpl37* | 0.87 | 0.48 |
| Muscle | Blue | Sex | *Rpl37a* | 0.89 | 0.48 |
| Muscle | Blue | Sex | *Rpl38* | 0.80 | 0.45 |
| Muscle | Blue | Sex | *Rpl41* | 0.90 | 0.45 |
| Muscle | Blue | Sex | *Rpl7a* | 0.83 | 0.42 |
| Muscle | Blue | Sex | *Rpl8* | 0.85 | 0.30 |
| Muscle | Blue | Sex | *Rplp1* | 0.89 | 0.48 |
| Muscle | Blue | Sex | *Rplp2* | 0.89 | 0.45 |
| Muscle | Blue | Sex | *Rps11* | 0.89 | 0.55 |
| Muscle | Blue | Sex | *Rps13* | 0.86 | 0.44 |
| Muscle | Blue | Sex | *Rps14* | 0.90 | 0.60 |
| Muscle | Blue | Sex | *Rps15* | 0.86 | 0.43 |
| Muscle | Blue | Sex | *Rps16* | 0.87 | 0.42 |
| Muscle | Blue | Sex | *Rps17* | 0.84 | 0.35 |
| Muscle | Blue | Sex | *Rps18* | 0.90 | 0.43 |
| Muscle | Blue | Sex | *Rps19* | 0.84 | 0.41 |
| Muscle | Blue | Sex | *Rps20* | 0.82 | 0.42 |
| Muscle | Blue | Sex | *Rps21* | 0.81 | 0.39 |
| Muscle | Blue | Sex | *Rps23* | 0.88 | 0.48 |
| Muscle | Blue | Sex | *Rps24* | 0.88 | 0.42 |
| Muscle | Blue | Sex | *Rps25* | 0.84 | 0.47 |
| Muscle | Blue | Sex | *Rps26* | 0.91 | 0.44 |
| Muscle | Blue | Sex | *Rps27* | 0.86 | 0.58 |
| Muscle | Blue | Sex | *Rps27a* | 0.87 | 0.45 |
| Muscle | Blue | Sex | *Rps27l* | 0.82 | 0.52 |
| Muscle | Blue | Sex | *Rps28* | 0.82 | 0.44 |
| Muscle | Blue | Sex | *Rps3* | 0.80 | 0.28 |
| Muscle | Blue | Sex | *Rps5* | 0.84 | 0.48 |
| Muscle | Blue | Sex | *Rps8* | 0.90 | 0.41 |
| Muscle | Blue | Sex | *Rps9* | 0.86 | 0.54 |
| Muscle | Blue | Sex | *S100a1* | 0.82 | 0.65 |
| Muscle | Blue | Sex | *Sem1* | 0.88 | 0.49 |
| Muscle | Blue | Sex | *Smim26* | 0.81 | 0.35 |
| Muscle | Blue | Sex | *Timm13* | 0.87 | 0.35 |
| Muscle | Blue | Sex | *Timm8b* | 0.89 | 0.38 |
| Muscle | Blue | Sex | *Tmem147* | 0.85 | 0.50 |
| Muscle | Blue | Sex | *Tmem256* | 0.84 | 0.41 |
| Muscle | Blue | Sex | *Tnni2* | 0.88 | 0.64 |
| Muscle | Blue | Sex | *Tomm7* | 0.89 | 0.39 |
| Muscle | Blue | Sex | *Tpt1* | 0.88 | 0.50 |
| Muscle | Blue | Sex | *Uqcc2* | 0.90 | 0.46 |
| Muscle | Blue | Sex | *Uqcr10* | 0.81 | 0.44 |
| Muscle | Blue | Sex | *Uqcr11* | 0.85 | 0.44 |
| Muscle | Blue | Sex | *Uqcrb* | 0.82 | 0.40 |
| Muscle | Blue | Sex | *Uqcrh* | 0.86 | 0.39 |
| Muscle | Blue | Sex | *Uqcrq* | 0.91 | 0.34 |
| Muscle | Blue | Sex | *mt-Nd2* | 0.80 | 0.53 |
