# Supplementary material for: Integration of segmented regression analysis with weighted gene correlation network analysis identifies genes whose expression is remodeled throughout physiological aging in mouse tissues
Source: Aging (Albany NY). 2021 Jul 29;13(14):18150–90. doi: 10.18632/aging.203379 (PMC8351669; doi:10.18632/aging.203379)
Supplement: Supplementary Table 5 [file aging-13-203379-s006.docx]

**Supplementary Table 5. Gene overlap between the top dynamic genes (Trendy) and the genes present in the significant modules.** Hub genes are highlighted in bold. Relates to Figure 4 and Supplemental Figure S4.

| **Tissue** | **Module** | **Significant association** | **Gene overlap** |
| --- | --- | --- | --- |
| Brain | Tan | Age | *Adamtsl4, Aga, Alpl, Anxa3, Anxa4, Apod, Arhgap45, Arpin, Arsg, Aspg, Ass1, Avp, B230209E15Rik,* ***B2m****, Bcl3, Bin2, Bst2,* ***C1qa****,* ***C1qb****,* ***C1qc****,* ***C3****,* ***C4b****, Capg, Cd37, Cd52, Cd68, Cd82, Chil1, Clec7a, Comp,* ***Csf1****, Csf3r, Cst7,* ***Ctsd****,* ***Ctsh****,* ***Ctss****,* ***Ctsz****,* ***Cx3cr1****, Cxcl16, Cyba, Cyth4, Dhx58, Emp3, Eva1c, F11r, Fcer1g, Fcgr1, Frmd4b, Galnt6,* ***Gbp3****,* ***Gfap****, Gm11681, Gm13905, Gm40466, Gpnmb, Gpr37, Gstm2, Gstm6, Gstt3,* ***H2-D1****, H2-Eb1,* ***H2-K1****, H2-Q5, H2-Q6, H2-Q7,* ***H2-T23****, Hapln2,* ***Hexb****, Hfe, Hvcn1, Icam1, Ier3,* ***Ifi27****, Ifi27l2a, Ifi35, Ifit1,* ***Ifit3****, Ifit3b, Ifitm3, Ighm, Igkc, Igtp, Il10ra, Il21r,* ***Il33****, Inpp5d, Irf5,* ***Irf7****, Irf8, Irf9, Irgm1, Irgm2,* ***Itgb2****, Itgb4, Itih5, Klc3,* ***Lag3****,* ***Laptm5****, Lcp1,* ***Lgals3****,* ***Lgals3bp****, Lgals9, Lpcat2, Lsp1, Ly86,* ***Lyz2****, Med18, Mms19, Msn, Myo1d, Myo1f, Ncf1,* ***Neat1****, Nfe2l2, Ntn5, Oasl2, Parp12, Parp3, Parvg, Pcdhb14, Pcdhb5, Pcdhb8, Pcdhb9, Pcdhga2, Pcdhga4, Pcdhga6, Pcdhga7, Pcdhga8, Pdlim4, Pisd-ps1, Plcg2, Pld4, Plek,* ***Psmb8****, Psmb9, Ptgis, Ptpn6, Rab20, Rac2, Rarg, Rasal3, Rftn1, Rnf207, Rtp4, S1pr3, Scamp2, Selplg,* ***Serpina3n****, Serpinb1a, Shc1,* ***Slc11a1****, Slc5a5, Smoc1, Sorbs3, Srebf1, Stat1, Stat6, Tap1,* ***Tap2****,* ***Tapbp****, Tcirg1, Thbs1, Thbs2, Tjp2, Tln1, Tmc6, Tmem176a, Tmem63a, Tnfrsf1a, Tnfrsf1b, Tor3a, Trappc6a, Trem2, Trim59, Tspo, Txnrd3, Tyrobp, Uba7, Unc93b1, Xaf1, Zc3hav1* |
| Heart | Tan | Age | *2310002L09Rik, 2310016D03Rik, Abat, Acsl6,* ***Acsm5****, Agtr1a, Aldob, Amot,* ***Amy1****, Ankrd1, Ankrd45, Arc, Camk2b, Casp12, Ccdc122, Ccl11, Ccr2, Cd209b,* ***Cd209f****, Cd209g, Cdh22,* ***Cds1****, Cnmd, Cpne7, Cpxm2, Cxcl14, Cyp2b10, Dpep1, Dpysl4, Edn3, Efemp1, Esm1, Gm2619, Gm42517, Gm44608, Gm7592, H2-M3, Hpse, Hrh2, Hsd11b1,* ***Ighg2c****, Il17re,* ***Kcnc1****, Kcnj14, Kcnk1, Lrrc3, Mef2c, Mettl11b, Myh7, Ninl, Nqo1, Ociad2, Parp3, Pcdhac2, Pcdhb14,* ***Pcdhb20****, Pcdhb22, Pced1b, Pde11a, Pla2g2d,* ***Prkcq****, Prkcz, Prlr, Prom2, Prune2, Ptprn, Rbm3, Ric3,* ***Scn4b****, Sh3gl2, Sh3rf2, Shtn1,* ***Skap2****, Slc22a3, Slc26a10, Slc6a17, Snai3, Snhg11, Tdrp, Tmem150c, Tmem196, Tmprss13, Tspan17, Ttc39b, Vegfd,* ***Vgll2****, Vit, Zfp536* |
|  | Blue | Age | *2210016F16Rik, 2310061I04Rik, A530016L24Rik, Aars2, Abcb10, Abhd11,* ***Acaa1a****,* ***Acaa2****, Acad9, Acadl, Acads, Acot1, Actr3b, Adh5, Adhfe1,* ***Adipor1****,* ***Adk****, Afg1l, Afg3l2, Agpat3, Aifm1, Aig1, Ak1, Ak3, Akr7a5, Amz2, Ap3d1, Apba3, Apip, Apool,* ***Arf1****, Asb8, Atp1a2, Atp1b1, Atp6v1b2, Atpaf2,* ***Auh****, Bag5, Bag6, Bckdhb, Bckdk, Bfar, Blmh, Bphl,* ***Bsg****, Btbd2, Bves, Capn2,* ***Capzb****, Car14, Carnmt1, Cars2,* ***Casq2****, Ccdc97,* ***Cct5****, Cct6a, Cct7,* ***Chchd3****, Chp1, Chst4,* ***Ckm****,* ***Ckmt2****, Copg1, Cops3,* ***Cops7a****,* ***Cops8****,* ***Coq2****, Coq4,* ***Coq5****,* ***Coq6****,* ***Coq9****,* ***Cox10****, Cpox, Cpsf1, Crispld2,* ***Ctbp1****,* ***Cyc1****, Dap3, Dctn1, Dda1, Ddost,* ***Dele1****, Derl1,* ***Des****, Dlat, Dlst, Dmac2, Dnaja2, Dnaja3,* ***Dnajb2****, Dnajc28,* ***Dnpep****, Dus3l, Dyrk2, Ears2,* ***Eci2****,* ***Ecsit****, Eef1akmt1, Ei24, Eif2b4, Eif4e, Emc2, Emc3,* ***Eno3****,* ***Etfb****, Exoc7, Faf1, Fahd1, Fam234a,* ***Fars2****,* ***Fastk****, Fblim1, Fbxl6,* ***Fbxw5****,* ***Fh1****,* ***Fkbp4****, Fkbp8, Flad1, Flywch1, Fndc5, Fnta, Foxred1, Fuca2, Gcdh, Gdpd1, Gfra4, Ggcx, Gid8, Ginm1, Gins4, Gmpr,* ***Gnpat****, Got1, Gpaa1, Gpc1, Gpr157, Gps2, Gpsm1, Gys1, H6pd,* ***Hadh****, Haus1, Hdhd2, Hhatl, Hibch, Hikeshi,* ***Hmgcl****, Hspa4l, Hspd1, Htra1,* ***Idh3g****, Ifngr2, Immp1l,* ***Immt****, Ipo13,* ***Isca1****, Jup, Kcnip2, Klhdc3, Klhl22, Kmt5a, Lap3, Laptm4b,* ***Ldhb****, Letm1, Lmcd1, Lmf1, Lrp10, Lrrc10, Lrtm1, Malsu1,* ***Map1lc3b****, Map2k3, Map7d1, Mapre2, Mat2b, Mavs, Mcat,* ***Mccc2****, Mcfd2, Mcur1,* ***Mdh1****,* ***Mdh2****, Med16, Med25, Mesd, Mgrn1, Mipep, Mkrn2, Mlycd, Mmaa, Mpv17, Mrfap1, Mrpl35,* ***Mrpl37****,* ***Mrpl38****, Mrpl39,* ***Mrpl4****, Mrpl44,* ***Mrpl45****, Mrps25, Mrps27, Mrps30, Mrps9, Mtch2, Mterf2, Mtif2, Myoz2,* ***Myzap****, Ncln,* ***Ndufa10****,* ***Ndufa9****,* ***Ndufs2****,* ***Nfs1****, Nkiras2, Noa1, Npepl1, Nudc, Nudt9, Oat,* ***Obscn****, Osgep, Osgepl1, Ostc, Oxct1, Paqr4, Pbxip1, Pcca, Pcyt2, Pde6d,* ***Pdhb****, Pdhx, Pdss2, Perp, Pex10, Pex19, Pex6, Pgap2, Pgk1,* ***Pgm2****,* ***Phb****,* ***Phyh****, Pla2g12a, Plin3, Plin5, Plod1, Pnkd,* ***Poldip2****, Polr2c, Popdc3, Ppif,* ***Ppp1ca****,* ***Ppp5c****,* ***Prdx3****, Preb,* ***Prkaca****, Psmc2,* ***Psmc4****, Psmd11, Psmd12, Psmd13,* ***Psmd2****, Ptdss2,* ***Ptges2****, Pttg1ip,* ***Pygm****, Rab11b, Rab22a, Rab2a, Rdh14, Rfng, Rhbdl3, Rilpl1, Rit1, Rmc1,* ***Rnf128****, Rnf187, Rnpepl1, Rom1,* ***Rpl3l****, Rpn2, Rps6ka2, Rtcb, Rtn4ip1, Rxra, Rxrb,* ***Rxrg****,* ***Samm50****, Scamp3, Scarb1, Sccpdh, Scyl1,* ***Sdhd****, Sf3b2,* ***Sgca****, Sgta, Sh3bp5, Sh3glb1, Sirt5,* ***Slc25a11****,* ***Slc25a12****, Slc25a13, Slc25a3, Slc25a34, Slc25a38,* ***Slc25a39****,* ***Slc25a5****,* ***Slc2a4****, Slc48a1,* ***Smpd1****, Smyd2,* ***Snta1****, Ssna1, St3gal4, Strap, Sucla2,* ***Suclg1****, Surf4, Syngr2, Sys1, Tango2, Tarbp2, Tars2, Tarsl2,* ***Tcp1****, Timm44, Tmed2, Tmem126b, Tmem150a, Tmem230, Tmem59,* ***Tmem70****, Tmod1, Tpra1,* ***Tprgl****, Trim63, Trpc4ap, Tsfm, Tspan7, Tuba4a,* ***Tufm****, Twf2,* ***Txn2****, Txndc15, Uba5, Ube2a,* ***Ube2b****,* ***Ube2g2****, Ube2h, Ube2j2, Ube2n, Ubl4a,* ***Ubl7****, Ublcp1, Uckl1, Uqcc1,* ***Uqcrc1****,* ***Uqcrc2****, Usp13, Vac14, Vdac2, Vdac3, Vezf1, Vldlr, Vps33a, Vps4a, Vps52, Wipi1, Xpnpep1, Ybx1,* ***Yipf3****, Ywhae, Zfp706, Zfp839* |
| Liver | Tan | Sex | *5033430I15Rik, 9430037G07Rik, A530020G20Rik, Abhd8, Ankrd46, Ap3s2, Apoo, Arf1, Arl16, Atg4b,* ***Babam1****, Bik, Bud31, Car14, Ccdc124, Cct2, Cct5, Cct7, Ces2c, Ces4a, Cgrrf1, Cib1, Clns1a, Clpp, Cmas, Cnih1, Cntrl, Copb2,* ***Cope****, Cops6, Cox18, Cyc1, Cyp2d10, Cyp2d40, Cyp8b1, Ddost, Ddx28, Derl3, Dhps, Dus2, Eef1g, Eif5a, Emc4, Erp29, Fam131c, Fam222b, Glyat, Gm16551, Gm31717,* ***Gps1****,* ***Grhpr****, Hacd2, Harbi1, Hax1, Higd1a, Ints11, Jtb, Lad1, Letm1, Lman2, Lmf1, Lsm1, Lsm6, Mapre3,* ***Mbl1****, Mettl26,* ***Mgst1****, Mrpl28, Mrpl37, Mrps22, Mrps7, Mtch2, Mthfd2, Mtif2,* ***Mup1****, Mup12, Mup13, Mup15, Mup16, Mup17, Mup19, Mup2, Mup-ps13, Nans, Ndufa10, Nfs1, Nhp2, Nlrp12,* ***Nme1****, Nop16, Nsmf, Nubp2, Nudt18, Pfkfb1, Pfn2, Plcxd2, Pomgnt1, Ppib, Pqbp1, Prkcsh, Psma5, Psmc3,* ***Psmc4****,* ***Psmd13****,* ***Psmd6****, Psph, Ptcd2, Ran, Rangap1,* ***Rarres1****, Reep5,* ***Rexo2****, Rint1, Rnf25, Rogdi, Rpl4, Rpusd3, Rtn4ip1, Saysd1, Sec11a, Sec13, Serinc2, Sgsm3, Slc22a7, Snap47, Spryd3, Stoml2, Tars2, Tbcb, Tcp1, Timm23, Tmem11, Tmem70, Tmem79, Tmx2, Tpgs1, Trmu, Tsen54, Unc45a, Upf3a, Urod, Vps45, Wdr83, Xpnpep1, Zdhhc24, Zfp428* |
|  | Salmon | Age | *9930111J21Rik2, A4galt, Acap1, Acod1, Acss1, AI662270, Akr1b10, Angptl8, Apobec3, Ar, Arpc1a, Arrdc1, Art2b, Bcl2a1d, Bgn, Card11, Ccl2, Ccl4,* ***Ccl5****, Ccr7, Cd160, Cd3e, Cd3g, Cd52, Cd6, Cd63, Cd74, Cd83, Cd8a, Cds1, Cep83os, Ciita, Ckap4, Cnn2, Col16a1, Col6a2, Cpm, Cx3cl1, Cx3cr1, Cxcr3, D630033O11Rik, Dchs1, Dennd2a, Dennd4b, Dgkz, E130307A14Rik, Efemp1, Epb41l2, Esrrb, Fign, Fmnl3, Fut4, Gdf3, Gimap3, Gm13375, Gm15523, Gm2619, Gm26873, Gm33699, Gm40457, Gm49410, Gm5122, Gm7592, Gnb2, Gpnmb, Gpr137b-ps, Gpr18, Gsg1l,* ***H2-Aa****, H2-Ab1, H2-DMa, H2-DMb1,* ***H2-Eb1****, H2-M2, H2-M3, Havcr2, Hic1, Hk2, Hvcn1, Ids, Ifi27, Ift57, Igfbp3, Igkv5-39, Ikzf1, Il16, Il21r, Impg2, Itgax, Itgb7, Jdp2, Kcng2, Klc1, Lat2, Lbh, Lncbate1, Lpxn, Ltb, Ltbp1, Ltbp4, Mamdc2, Mfng, Mlph, Mmp12, Mmp2, Mrpl48, Ms4a7, Myo9a, Naip5, Nod2, Nt5c2,* ***Ntrk2****, Oxct1, Papln, Pdgfb, Pdk3, Pear1, Pianp, Pld2, Pola1, Prex1, Ptpn7, Rasgrp1, Rassf2, Rel, Rims3, Rps18-ps3, S100a11, S100a4, Scarf2, Sema3f, Sema4d, Sh2d2a, Shisa3,* ***Slamf7****, Slc4a9, Smim24, Snn, Sp110, Srgap2, Stard9, Syk, Tamm41, Tbc1d9, Thy1, Tm4sf1, Tmem132e, Tmem45b, Tnfaip3, Tnfaip8, Tnfrsf26, Tns3, Unc13d, Unc5b, Vim, Wdfy4, Wipf1* |
|  | Darkturquoise | Age | *A530040E14Rik, AU020206, Btla,* ***Cd19****, Cd2, Cd22, Cd3d,* ***Cd79a****,* ***Cd79b****, Cklf, Cxcr5, Dock11, Fcrla, Gpr137b, H2-DMb2, H2-Ob, Igha,* ***Ighm****, Ighv1-55, Ighv1-78,* ***Igkc****,* ***Igkv3-5****, Igkv6-23,* ***Iglc2****, Iglc3, Irf4,* ***Jchain****, Lax1, Map4k1, Mgat4a, Mrc2,* ***Mzb1****, Napsa, Pax5, Prdm1, Rhoh, Rragd, Runx3, Siglecg, Slc25a24, Slpi, Tnfrsf13b* |
|  | Darkgrey | Sex | ***9130409I23Rik****, Akr1c19, Apba2,* ***Arsa****, Btc, C430042M11Rik, Cidea,* ***Cidec****,* ***Clstn3****,* ***Cox19****, Cplane1, Cyp2c55,* ***Cyp2u1****,* ***Dpy19l3****, Dsg1c, Epb41l1,* ***Fancl****, Fbxo40,* ***Fitm1****, Gal3st1, Gas2, Gm15411, Gm31522, Gm3776, Gm48120, Gm5524, Gnai1,* ***Gpc1****,* ***Gprc5b****, Gsta1, Gsta2, Gstm1, Gstm3, Hnmt, Iars2, Kcp, Lrrc15, Map3k20,* ***Nat8****,* ***Ntrk1****, Nudt19, Ogfrl1, Olfm3,* ***Olig1****,* ***Osbpl3****,* ***Pard3b****, Pdgfa, Pir, Pla2g6, Plekhb1, Pnldc1, Prpf19, Prss8,* ***Rassf3****, Rpp40, Samd4, Scd1, Serpina7, Slc35d1,* ***Snhg11****, Snrpn, Socs7, Swap70, Tbc1d4, Tiam2, Tle3, Tmem51, Trhde, Tspan17,* ***Unc119****,* ***Uox****, Upp1, Whamm, Zfp54, Zfp951, Znf41-ps* |
|  | Cyan | Sex | *9530034E10Rik, Abhd5, Adal, Adpgk, Ahcyl2, Ahsa1, Ahsa2, Aktip, Alg8, Ankrd23,* ***Arcn1****, Arf4, Arl1, Armcx3, Atg13, Baat, Bcl2l1, Bop1, C9orf72, Cacybp, Calr, Calu, Ccar1, Ccdc125, Ccdc25, Cct3, Cct6a, Cdk2ap2, Cenpt, Clic4, Clptm1l, Copb1,* ***Copg1****, Copz1, Creld1,* ***Creld2****, Crem, Cstf1, Ddx1, Dhx29, Dnajb1, Dnajb11, Dnajc3, Dtwd1, Eif3b, Eif4e, Elavl1, Elovl1, Eprs, Erp44, Fam222a, Gart, Gdap2, Gm19409, Gm43305, Gosr2, Hars, Hip1r, Hnrnph2, Hsp90aa1, Hsp90ab1, Hsp90b1,* ***Hspa5****, Hsph1, Hyou1,* ***Iars****, Insc, Jpt2, Kctd3, Lbhd1, Lrrc59, Lysmd3, Map7d1, Morf4l2, Naa25, Nat10, Ncbp1, Nelfa, Nudcd1, Nudt5, Nup62, P4ha2, Papss1, Pdia3, Pdia6, Piga, Pno1, Ppid, Ppme1, Prkar2a, Rbbp5, Ric8b, Riok3, Rpn1, Scfd1, Scfd2,* ***Sdf2l1****, Sec16a,* ***Sec22b****,* ***Sec24d****,* ***Sec61a1****, Selenos,* ***Serp1****,* ***Slc33a1****, Slc35b1, Slc35c1, Spcs2, Srp72, Srrt,* ***Ssr1****, Ssr3, Stip1, Stx5a, Surf4, Tardbp, Tirap, Tmed7, Tmed9, Tmem167, Tmem214, Tmem263, Tmem33, Tnpo3, Ttpal, Tubgcp4, Uba5, Ube2g2, Ube2j1, Ubfd1, Ubqln1, Unc119b, Usp14, Wars, Yipf5, Ywhaz, Zbtb7c, Zfc3h1, Zw10* |
|  | Blue | Sex | ***1110038B12Rik****, 1700123O20Rik, 2610301B20Rik, 4930402H24Rik, 6030458C11Rik, Aars,* ***Abcg2****, Acadsb,* ***Acox1****, Acox2,* ***Actr1b****, Adgrf1, Adh6-ps1, Adora1, Adrb3,* ***AI182371****,* ***AI463229****, Alas2, Alg3, Amfr,* ***Ankrd52****, Aox3, Ap2m1, Ap3s1, Apex2, Apoa1, Apob, Arhgap42,* ***Arl2bp****, Arl6ip1,* ***Arsg****, Asb13, Asns, Atg16l2, Atp13a1, B3galt1, B4galt7, BC021767,* ***Bcap31****, Becn1, Bet1,* ***Bpnt1****, Brcc3, Btg3, C1ql4, C1ra, C2, C330002G04Rik, C4bp,* ***C6****,* ***C730027H18Rik****, C8a, C8b, C9, Cabyr, Capn8, Ccdc58, Ccl25, Ccnf, Cct8, Cdc123,* ***Cdipt****,* ***Ces2b****, Ces3a, Cfhr2, Chmp7,* ***Chrd****, Cldnd1, Clk3, Clpb, Cltb, Clu, Cmah, Cmpk1,* ***Cmtm6****, Cnot9, Coa5, Cobl, Comt, Cpne8, Cpsf1, Cript,* ***Csad****,* ***Csnk1d****, Cspg5,* ***Ctr9****,* ***Cul2****, Cux1, Cycs, Cyp21a2-ps, Cyp2c67, Cyp2d11, Cyp2d41-ps, Cyp2d9, Cyp2f2,* ***Cyp4a12a****, Cyp4a12b, Cyp7b1, Cyth2, Dcaf12,* ***Dcakd****, Ddx41, Ddx49, Deptor, Desi1, Dis3l, Dna2, Dnajc21,* ***Dnd1****, Dnm2, Dpp7, Drg1, Dtnb,* ***Dus1l****, Echdc2, Edem2, Efr3a, Egfr, Eif2d, Eif2s3y, Eif3l, Eif4ebp1, Eif4g3,* ***Eif5****,* ***Elovl2****,* ***Elovl3****, Enpp2, Enpp3,* ***Ephx2****, Ergic2,* ***Etfbkmt****, F12, F2r, F830016B08Rik, Fahd1, Fbxo22, Fbxo4, Fermt2, Fgd6, Fh1, Fkbp11, Flad1, Frrs1, Gabarapl2, Galns,* ***Garem1****, Gars, Gdf9, Glmp, Gm12366, Gm15638,* ***Gm15883****, Gm17228, Gm26967, Gm29475,* ***Gm31036****, Gm38266, Gm38416,* ***Gm40787****, Gm45724, Gm47465, Gm48199, Gm48855,* ***Gm49338****, Gm49405, Gnai3, Gorasp2,* ***Gpsm2****, Grina,* ***Gspt1****,* ***Gsr****, Gstp1, Gstp2, Gstp-ps,* ***Gt(ROSA)26Sor****, Gucd1, H13, H6pd, Habp4, Hao1, Herc4, Hmbs, Hprt,* ***Hsd17b12****, Hsd17b13, Hsd3b5, Hspa9, Hspb1, Hypk,* ***Igsf5****, Ihh, Ikbkg, Il1rap, Impa1,* ***Inhbc****,* ***Ints9****, Ipp, Itpa, Kat2a, Kat8, Kctd15, Keg1, Kif21a, Klhl2, Kmo, Kyat1, Lactb2, Lama3, Lamp1, Lancl1, Lap3, Ldah,* ***Ldha****, Ldhd,* ***Lrrc3****, Lrrfip2,* ***Lurap1l****, Maged1, Magee1, Manba,* ***Map1lc3b****, Map2k1, Map7, Marveld3, Masp2, Mbd3, Mcur1, Mdh2,* ***Mecr****, Memo1, Mfsd8, Mier2, Mindy3, Mpi, Mpp6, Mrpl38, Mtfr1, Mto1, Mug1, Mup10, Mup11, Mup14, Mup20, Mup21, Mup3, Mup7,* ***Mup9****, Mup-ps20, Mup-ps7, Myl9,* ***Myo1c****,* ***Myo6****, Nars, Nat8f5, Ncoa4, Nde1, Nek2, Nek6, Nhsl1, Nipa2, Nomo1, Nras, Nsun2, Nsun4, Nudc, Nudcd2, Nudt7, Obp2a,* ***Ola1****, Omd, Osbpl9, P2rx4,* ***Paip2****, Pak4, Pdhx,* ***Pdilt****, Pdlim7, Pgam1, Pgm3,* ***Phyh****, Pim2,* ***Pip5k1a****,* ***Pisd****, Pitpnb, Plaa, Pms1, Pnpo, Podn, Polrmt, Pon2, Ppm1d, Ppp1r15a, Ppp1r2, Ppp4c, Prep, Prpf6, Psmc2, Psmd1, Psmd12,* ***Psmd14****, Psmd2, Psmd5, Psmd7,* ***Psme3****, Ptpn11, Pycrl, Rab11a, Rab14, Rab6a, Raf1, Ranbp9, Rbbp4, Rbm7, Rc3h1,* ***Rest****, Rmc1, Rnf6, Rnmt, Rpe,* ***Rpl26-ps6****, Rpn2, Rrbp1,* ***Sars****, Scamp3, Scamp5, Scara5,* ***Scp2****, Sec23a, Sec61a2, Sec63, Sel1l3,* ***Selenbp2****, Selenof,* ***Sephs1****,* ***Serinc1****,* ***Serpina11****, Serpina1c, Serpina1e, Serpina3k,* ***Serpina4-ps1****,* ***Serpine2****, Slc11a2, Slc16a13, Slc22a28, Slc22a30, Slc25a17, Slc30a7, Slc30a9,* ***Slc35b3****,* ***Slc35e3****, Slc38a10, Slco1a1,* ***Smpd1****,* ***Socs4****,* ***Sort1****, Spc25, Spns1, Spop, Srpk1, Srpr, Sstr2,* ***Stat6****, Stk38, Strbp, Sumf1, Synj2bp, Tank, Tax1bp1, Tbk1, Tbl3, Tedc2, Terf2ip,* ***Tesk2****, Tfdp1, Tfe3, Them4,* ***Thoc6****, Timm9, Tm9sf3, Tmc7,* ***Tmem125****, Tmem150a, Tmem186, Tmem243, Top3a,* ***Topors****, Tpst1, Tpst2,* ***Tram1****, Trip4,* ***Tsg101****,* ***Ttc33****, Ttc39c, Tubg1, Tuft1, Txnl4a,* ***Ubac2****, Ube2a, Ubp1, Ubtd1, Ubxn2a, Uchl5, Ufd1, Ufsp2, Ugt2b1, Ugt2b35,* ***Ugt2b38****,* ***Ugt2b5****, Usp10, Usp3, Usp9x, Vapa, Vcp, Vmp1, Vps25, Vtn, Washc2, Wdr53, Wipi2, Xiap, Yars, Yipf2,* ***Yipf3****, Ywhaq, Zbtb20,* ***Zbtb42****,* ***Zc3h14****, Zdhhc12, Zdhhc6, Zfp112, Zfp445, Zfp592,* ***Zfp706****,* ***Zfyve1****, Zmpste24, Zmynd19,* ***Zranb2****,* ***Zyg11a*** |
| Muscle | Magenta | Age | *Aldh1a1, Amy1, Arih2, Arpp19, Asb15, Atg4a, Bmi1, Cacng1, Camk2b, Ccdc122, Ccdc85a, Chd1l, Cnbp,* ***Cpe****, Ddx50, Des, Eif2b3, Eif3a,* ***Eif3e****, Elf3, Etf1, Fam241b, Fech, Flot1, Gm14261,* ***Itgb5****,* ***Kcmf1****, Larp1, Lonp2, Lrrfip2, Map4, Map7,* ***Mib1****, mt-Nd1, mt-Nd5, Mvp, Nfkbib, Nploc4, Nqo1, Ociad2, Pacsin3, Pdzrn3,* ***Plekhb1****, Pou4f1, Prkcq, Psmd2, Psmd4,* ***Rab2a****, Ramp1,* ***Rasd2****, Rbbp7, Riok3, Rorc, Rpl22,* ***Setd3****, Smyd2, Sorbs2, Ssu72, Ston2, Synm, Syt9, Thap12, Tmem19, Tmod4, Tsnax, Uaca, Ube2r2, Vcp, Zrsr1, Zxdc* |
|  | Brown | Age | *1810055G02Rik, Abcd1, Abi3bp, Ace, Actn1, Adamts10, AI506816,* ***Angptl1****,* ***Anxa2****, Aplp2, Boc, Cald1, Ccnd3, Cd248,* ***Cd34****, Cdr2l, Chodl, Cilp2, Ckb, Clu, Col15a1, Col2a1,* ***Col3a1****, Col4a1, Col4a2,* ***Col5a1****,* ***Col5a2****, Col5a3,* ***Col6a1****,* ***Col6a2****,* ***Col6a3****, Creb3l1, Crtap, Ctsk, Dbn1, Dcn,* ***Dok2****, Dpt,* ***Dstn****, Eef1a1, Emp1, Entpd1, Epb41l2, Exoc7, Fbln2, Fgl2, Fibin, Fkbp10, Fmod,* ***Fn1****,* ***Fndc1****, Fndc3b,* ***Fstl1****, Fzd1, Ganab, Gclm, Ggta1, Gm11451, Golim4, Grem2, Gsn, Hacd1, Hpcal1, Htra3, Igf1,* ***Igfbp6****, Itga11,* ***Itgbl1****, Itm2c, Kctd12, Kera, Lamb1, Lamc1, Lox, Loxl1, Lrrc15, Ltbp1, Ltbr, Lyz1, Maged1, Map2k3, Map4k4, Mbtps1, Mfap4, Mlf1, Mob1a, Mrgprg, Mthfr,* ***Ndn****,* ***Nid1****, Nsmf, Ntn1,* ***Olfml3****, Pabpc1, Palld,* ***Pcolce****,* ***Pcolce2****, Pdgfra, Pdgfrl, Pdia4, Pgghg, Phyhd1,* ***Pi16****, Piezo1, Pip4p2, Plod1, Plod3,* ***Plxdc2****, Podn, Prelp, Prrg3, Pttg1ip, Ret, Rftn1, Rmdn1, Rnd3,* ***Serpinf1****,* ***Serping1****, Serpinh1, Sf3b3,* ***Sod3****,* ***Sparc****, Sptbn1, Srpx,* ***Ssc5d****, St6galnac4, Stt3a, Sulf1, Tceal8, Telo2, Tent5a,* ***Tgfbi****, Thbs2, Thbs3, Thbs4,* ***Timp2****, Tmem178b, Tmem30b, Tnc, Tnmd, Tram1, Trp53inp2, Tspan5, Tubb4a, Wfdc1* |
|  | Blue | Sex | *A830052D11Rik, Arpc5l,* ***Atp5g3****,* ***Atp5l****, Atp6v1f, Avpi1, Card19, Ccdc85c, Cltb,* ***Cox5a****, Crim1, Cst6, CT010467.1, Dtnbp1, Dvl1,* ***Elob****, Eloc, F830016B08Rik, Fam53a, Gm7607, Grpel1, Kcne1l,* ***Mrpl14****, Mrps12,* ***Mrps18a****,* ***mt-Nd2****,* ***Ndufa8****,* ***Ndufb2****, Neurl2, Nxn,* ***Pam16****,* ***Pfdn5****,* ***Psmb1****, Psmb2,* ***Psmb3****, Psmg4,* ***Rpl13****,* ***Rpl27****,* ***Rpl36****,* ***Rpl41****,* ***Rps14****,* ***Rps16****, Rtraf, Sctr, Selenok,* ***Sem1****, Smim4, Tbcb,* ***Tnni2****, Tsg101, Ubc, Ube2e1, Vps28, Zdhhc18, Zdhhc4, Zfp358, Zranb1, Zswim7* |
