# Supplementary material for: Integration of segmented regression analysis with weighted gene correlation network analysis identifies genes whose expression is remodeled throughout physiological aging in mouse tissues
Source: Aging (Albany NY). 2021 Jul 29;13(14):18150–90. doi: 10.18632/aging.203379 (PMC8351669; doi:10.18632/aging.203379)
Supplement: Supplementary Table 6 [file aging-13-203379-s007.docx]

**Supplementary Table 6. Gene Ontology (GO) over-representation and network analysis.** GO terms with FDR adjusted *p-values* less than 0.05 were considered for analysis. Summary network *p-values* and adjusted p-values were automatically calculated by the software and consist of the mean value of the individual GO terms' *p-values* and adjusted *p-values*. GeneRatio refers to number of genes of the input list that are annotated to the GO term. BgRatio refers to the total number of genes annotated to that specific GO term within the tissue-specific universe of expressed genes. Relates to Table 1.

| **Tissue** | **Module** | **Significant Association** | **GO ID** | **Description** | **GeneRatio** | **BgRatio** | **FDR** | **Genes** | **Summary network description** | **Summary network FDR** |
| --- | --- | --- | --- | --- | --- | --- | --- | --- | --- | --- |
| Brain | Tan | Age | GO:0002443 | leukocyte mediated immunity | 15/34 | 421/19660 | 7.68E-14 | *C4b, H2-K1, C3, H2-D1, Itgb2, Irf7, Ctsh, Lag3, B2m, Slc11a1, C1qa, Tap2, C1qc, C1qb, H2-T23* | mediated immunoglobulin regulation response | 8.06E-03 |
| Brain | Tan | Age | GO:0002449 | lymphocyte mediated immunity | 14/34 | 339/19660 | 7.68E-14 | *C4b, H2-K1, C3, H2-D1, Irf7, Ctsh, Lag3, B2m, Slc11a1, C1qa, Tap2, C1qc, C1qb, H2-T23* | mediated immunoglobulin regulation response | 8.06E-03 |
| Brain | Tan | Age | GO:0002460 | adaptive immune response based on somatic recombination of immune receptors built from immunoglobulin superfamily domains | 14/34 | 353/19660 | 9.00E-14 | *C4b, H2-K1, C3, H2-D1, Irf7, Ctsh, B2m, Slc11a1, C1qa, Tap2, Il33, C1qc, C1qb, H2-T23* | mediated immunoglobulin regulation response | 8.06E-03 |
| Brain | Tan | Age | GO:0002250 | adaptive immune response | 15/34 | 476/19660 | 1.35E-13 | *C4b, H2-K1, C3, H2-D1, Irf7, Ctsh, Lag3, B2m, Slc11a1, C1qa, Tap2, Il33, C1qc, C1qb, H2-T23* | mediated immunoglobulin regulation response | 8.06E-03 |
| Brain | Tan | Age | GO:0019882 | antigen processing and presentation | 9/34 | 97/19660 | 1.08E-11 | *H2-K1, Ctss, H2-D1, Psmb8, B2m, Slc11a1, Tap2, H2-T23, Tapbp* | mediated immunoglobulin regulation response | 8.06E-03 |
| Brain | Tan | Age | GO:0048002 | antigen processing and presentation of peptide antigen | 8/34 | 60/19660 | 1.26E-11 | *H2-K1, Ctss, H2-D1, B2m, Slc11a1, Tap2, H2-T23, Tapbp* | mediated immunoglobulin regulation response | 8.06E-03 |
| Brain | Tan | Age | GO:0002483 | antigen processing and presentation of endogenous peptide antigen | 6/34 | 33/19660 | 1.97E-09 | *H2-K1, H2-D1, B2m, Tap2, H2-T23, Tapbp* | mediated immunoglobulin regulation response | 8.06E-03 |
| Brain | Tan | Age | GO:0019885 | antigen processing and presentation of endogenous peptide antigen via MHC class I | 6/34 | 33/19660 | 1.97E-09 | *H2-K1, H2-D1, B2m, Tap2, H2-T23, Tapbp* | mediated immunoglobulin regulation response | 8.06E-03 |
| Brain | Tan | Age | GO:0002819 | regulation of adaptive immune response | 9/34 | 183/19660 | 1.97E-09 | *H2-K1, C3, H2-D1, Irf7, B2m, Slc11a1, Tap2, Il33, H2-T23* | mediated immunoglobulin regulation response | 8.06E-03 |
| Brain | Tan | Age | GO:0019883 | antigen processing and presentation of endogenous antigen | 6/34 | 36/19660 | 3.02E-09 | *H2-K1, H2-D1, B2m, Tap2, H2-T23, Tapbp* | mediated immunoglobulin regulation response | 8.06E-03 |
| Brain | Tan | Age | GO:0002474 | antigen processing and presentation of peptide antigen via MHC class I | 6/34 | 43/19660 | 8.52E-09 | *H2-K1, H2-D1, B2m, Tap2, H2-T23, Tapbp* | mediated immunoglobulin regulation response | 8.06E-03 |
| Brain | Tan | Age | GO:0002699 | positive regulation of immune effector process | 9/34 | 233/19660 | 1.26E-08 | *H2-K1, C3, H2-D1, Itgb2, Lag3, B2m, Tap2, Il33, H2-T23* | mediated immunoglobulin regulation response | 8.06E-03 |
| Brain | Tan | Age | GO:0001906 | cell killing | 8/34 | 153/19660 | 1.26E-08 | *H2-K1, C3, H2-D1, Ctsh, Lag3, B2m, Tap2, H2-T23* | mediated immunoglobulin regulation response | 8.06E-03 |
| Brain | Tan | Age | GO:0002705 | positive regulation of leukocyte mediated immunity | 8/34 | 155/19660 | 1.30E-08 | *H2-K1, C3, H2-D1, Itgb2, Lag3, B2m, Tap2, H2-T23* | mediated immunoglobulin regulation response | 8.06E-03 |
| Brain | Tan | Age | GO:0002822 | regulation of adaptive immune response based on somatic recombination of immune receptors built from immunoglobulin superfamily domains | 8/34 | 167/19660 | 2.20E-08 | *H2-K1, C3, H2-D1, B2m, Slc11a1, Tap2, Il33, H2-T23* | mediated immunoglobulin regulation response | 8.06E-03 |
| Brain | Tan | Age | GO:0002478 | antigen processing and presentation of exogenous peptide antigen | 5/34 | 25/19660 | 3.33E-08 | *H2-K1, B2m, Tap2, H2-T23, Tapbp* | mediated immunoglobulin regulation response | 8.06E-03 |
| Brain | Tan | Age | GO:0002697 | regulation of immune effector process | 10/34 | 382/19660 | 3.33E-08 | *H2-K1, C3, H2-D1, Lgals3, Itgb2, Lag3, B2m, Tap2, Il33, H2-T23* | mediated immunoglobulin regulation response | 8.06E-03 |
| Brain | Tan | Age | GO:0001913 | T cell mediated cytotoxicity | 6/34 | 63/19660 | 5.48E-08 | *H2-K1, H2-D1, Ctsh, B2m, Tap2, H2-T23* | mediated immunoglobulin regulation response | 8.06E-03 |
| Brain | Tan | Age | GO:0002428 | antigen processing and presentation of peptide antigen via MHC class Ib | 5/34 | 28/19660 | 5.48E-08 | *H2-K1, H2-D1, B2m, Tap2, H2-T23* | mediated immunoglobulin regulation response | 8.06E-03 |
| Brain | Tan | Age | GO:0002824 | positive regulation of adaptive immune response based on somatic recombination of immune receptors built from immunoglobulin superfamily domains | 7/34 | 121/19660 | 6.07E-08 | *H2-K1, C3, H2-D1, B2m, Slc11a1, Tap2, H2-T23* | mediated immunoglobulin regulation response | 8.06E-03 |
| Brain | Tan | Age | GO:0002456 | T cell mediated immunity | 7/34 | 122/19660 | 6.07E-08 | *H2-K1, H2-D1, Ctsh, B2m, Slc11a1, Tap2, H2-T23* | mediated immunoglobulin regulation response | 8.06E-03 |
| Brain | Tan | Age | GO:0002708 | positive regulation of lymphocyte mediated immunity | 7/34 | 122/19660 | 6.07E-08 | *H2-K1, C3, H2-D1, Lag3, B2m, Tap2, H2-T23* | mediated immunoglobulin regulation response | 8.06E-03 |
| Brain | Tan | Age | GO:0001909 | leukocyte mediated cytotoxicity | 7/34 | 123/19660 | 6.15E-08 | *H2-K1, H2-D1, Ctsh, Lag3, B2m, Tap2, H2-T23* | mediated immunoglobulin regulation response | 8.06E-03 |
| Brain | Tan | Age | GO:0019884 | antigen processing and presentation of exogenous antigen | 5/34 | 31/19660 | 7.47E-08 | *H2-K1, B2m, Tap2, H2-T23, Tapbp* | mediated immunoglobulin regulation response | 8.06E-03 |
| Brain | Tan | Age | GO:0002821 | positive regulation of adaptive immune response | 7/34 | 128/19660 | 7.49E-08 | *H2-K1, C3, H2-D1, B2m, Slc11a1, Tap2, H2-T23* | mediated immunoglobulin regulation response | 8.06E-03 |
| Brain | Tan | Age | GO:0002475 | antigen processing and presentation via MHC class Ib | 5/34 | 32/19660 | 8.16E-08 | *H2-K1, H2-D1, B2m, Tap2, H2-T23* | mediated immunoglobulin regulation response | 8.06E-03 |
| Brain | Tan | Age | GO:0001912 | positive regulation of leukocyte mediated cytotoxicity | 6/34 | 73/19660 | 9.34E-08 | *H2-K1, H2-D1, Lag3, B2m, Tap2, H2-T23* | mediated immunoglobulin regulation response | 8.06E-03 |
| Brain | Tan | Age | GO:0002703 | regulation of leukocyte mediated immunity | 8/34 | 227/19660 | 1.34E-07 | *H2-K1, C3, H2-D1, Itgb2, Lag3, B2m, Tap2, H2-T23* | mediated immunoglobulin regulation response | 8.06E-03 |
| Brain | Tan | Age | GO:0150146 | cell junction disassembly | 4/34 | 13/19660 | 1.74E-07 | *C3, C1qa, C1qc, C1qb* | mediated immunoglobulin regulation response | 8.06E-03 |
| Brain | Tan | Age | GO:0031343 | positive regulation of cell killing | 6/34 | 85/19660 | 2.13E-07 | *H2-K1, H2-D1, Lag3, B2m, Tap2, H2-T23* | mediated immunoglobulin regulation response | 8.06E-03 |
| Brain | Tan | Age | GO:0001916 | positive regulation of T cell mediated cytotoxicity | 5/34 | 42/19660 | 2.86E-07 | *H2-K1, H2-D1, B2m, Tap2, H2-T23* | mediated immunoglobulin regulation response | 8.06E-03 |
| Brain | Tan | Age | GO:0001910 | regulation of leukocyte mediated cytotoxicity | 6/34 | 96/19660 | 4.17E-07 | *H2-K1, H2-D1, Lag3, B2m, Tap2, H2-T23* | mediated immunoglobulin regulation response | 8.06E-03 |
| Brain | Tan | Age | GO:0002706 | regulation of lymphocyte mediated immunity | 7/34 | 171/19660 | 4.27E-07 | *H2-K1, C3, H2-D1, Lag3, B2m, Tap2, H2-T23* | mediated immunoglobulin regulation response | 8.06E-03 |
| Brain | Tan | Age | GO:0001914 | regulation of T cell mediated cytotoxicity | 5/34 | 47/19660 | 4.67E-07 | *H2-K1, H2-D1, B2m, Tap2, H2-T23* | mediated immunoglobulin regulation response | 8.06E-03 |
| Brain | Tan | Age | GO:0016064 | immunoglobulin mediated immune response | 7/34 | 189/19660 | 8.03E-07 | *C4b, C3, Irf7, C1qa, C1qc, C1qb, H2-T23* | mediated immunoglobulin regulation response | 8.06E-03 |
| Brain | Tan | Age | GO:0019724 | B cell mediated immunity | 7/34 | 193/19660 | 9.01E-07 | *C4b, C3, Irf7, C1qa, C1qc, C1qb, H2-T23* | mediated immunoglobulin regulation response | 8.06E-03 |
| Brain | Tan | Age | GO:0031341 | regulation of cell killing | 6/34 | 115/19660 | 1.07E-06 | *H2-K1, H2-D1, Lag3, B2m, Tap2, H2-T23* | mediated immunoglobulin regulation response | 8.06E-03 |
| Brain | Tan | Age | GO:0002711 | positive regulation of T cell mediated immunity | 5/34 | 64/19660 | 2.03E-06 | *H2-K1, H2-D1, B2m, Tap2, H2-T23* | mediated immunoglobulin regulation response | 8.06E-03 |
| Brain | Tan | Age | GO:0002476 | antigen processing and presentation of endogenous peptide antigen via MHC class Ib | 4/34 | 27/19660 | 3.05E-06 | *H2-K1, H2-D1, Tap2, H2-T23* | mediated immunoglobulin regulation response | 8.06E-03 |
| Brain | Tan | Age | GO:0002484 | antigen processing and presentation of endogenous peptide antigen via MHC class I via ER pathway | 4/34 | 27/19660 | 3.05E-06 | *H2-K1, H2-D1, Tap2, H2-T23* | mediated immunoglobulin regulation response | 8.06E-03 |
| Brain | Tan | Age | GO:0002709 | regulation of T cell mediated immunity | 5/34 | 87/19660 | 8.88E-06 | *H2-K1, H2-D1, B2m, Tap2, H2-T23* | mediated immunoglobulin regulation response | 8.06E-03 |
| Brain | Tan | Age | GO:0006956 | complement activation | 5/34 | 126/19660 | 5.46E-05 | *C4b, C3, C1qa, C1qc, C1qb* | mediated immunoglobulin regulation response | 8.06E-03 |
| Brain | Tan | Age | GO:0006959 | humoral immune response | 6/34 | 231/19660 | 5.61E-05 | *C4b, C3, C1qa, C1qc, C1qb, H2-T23* | mediated immunoglobulin regulation response | 8.06E-03 |
| Brain | Tan | Age | GO:0032103 | positive regulation of response to external stimulus | 7/34 | 373/19660 | 6.30E-05 | *Ctss, C3, Csf1, Irf7, Lag3, Il33, H2-T23* | mediated immunoglobulin regulation response | 8.06E-03 |
| Brain | Tan | Age | GO:0031349 | positive regulation of defense response | 6/34 | 248/19660 | 8.08E-05 | *Ctss, C3, Irf7, Lag3, Il33, H2-T23* | mediated immunoglobulin regulation response | 8.06E-03 |
| Brain | Tan | Age | GO:0010977 | negative regulation of neuron projection development | 5/34 | 172/19660 | 2.28E-04 | *Gfap, H2-K1, H2-D1, B2m, Ctsz* | mediated immunoglobulin regulation response | 8.06E-03 |
| Brain | Tan | Age | GO:0070661 | leukocyte proliferation | 6/34 | 303/19660 | 2.39E-04 | *Csf1, Lgals3, Itgb2, Slc11a1, Il33, H2-T23* | leukocyte granulocyte migration chemotaxis | 1.48E-02 |
| Brain | Tan | Age | GO:0002486 | antigen processing and presentation of endogenous peptide antigen via MHC class I via ER pathway, TAP-independent | 3/34 | 26/19660 | 2.39E-04 | *H2-K1, H2-D1, H2-T23* | mediated immunoglobulin regulation response | 8.06E-03 |
| Brain | Tan | Age | GO:0042116 | macrophage activation | 4/34 | 83/19660 | 2.43E-04 | *Slc11a1, C1qa, Il33, Cx3cr1* | neuron death activation microglial | 6.59E-03 |
| Brain | Tan | Age | GO:0045766 | positive regulation of angiogenesis | 5/34 | 178/19660 | 2.48E-04 | *C3, Lgals3, Itgb2, Ctsh, Cx3cr1* | leukocyte granulocyte migration chemotaxis | 1.48E-02 |
| Brain | Tan | Age | GO:1904018 | positive regulation of vasculature development | 5/34 | 197/19660 | 3.96E-04 | *C3, Lgals3, Itgb2, Ctsh, Cx3cr1* | leukocyte granulocyte migration chemotaxis | 1.48E-02 |
| Brain | Tan | Age | GO:0002274 | myeloid leukocyte activation | 5/34 | 200/19660 | 4.18E-04 | *Itgb2, Slc11a1, C1qa, Il33, Cx3cr1* | leukocyte granulocyte migration chemotaxis | 1.48E-02 |
| Brain | Tan | Age | GO:0031345 | negative regulation of cell projection organization | 5/34 | 202/19660 | 4.30E-04 | *Gfap, H2-K1, H2-D1, B2m, Ctsz* | mediated immunoglobulin regulation response | 8.06E-03 |
| Brain | Tan | Age | GO:0002253 | activation of immune response | 6/34 | 344/19660 | 4.33E-04 | *C4b, C3, Lgals3, C1qa, C1qc, C1qb* | mediated immunoglobulin regulation response | 8.06E-03 |
| Brain | Tan | Age | GO:0006958 | complement activation, classical pathway | 4/34 | 105/19660 | 5.49E-04 | *C3, C1qa, C1qc, C1qb* | mediated immunoglobulin regulation response | 8.06E-03 |
| Brain | Tan | Age | GO:0002455 | humoral immune response mediated by circulating immunoglobulin | 4/34 | 117/19660 | 7.82E-04 | *C3, C1qa, C1qc, C1qb* | mediated immunoglobulin regulation response | 8.06E-03 |
| Brain | Tan | Age | GO:0001774 | microglial cell activation | 3/34 | 41/19660 | 7.82E-04 | *C1qa, Il33, Cx3cr1* | neuron death activation microglial | 6.59E-03 |
| Brain | Tan | Age | GO:0002269 | leukocyte activation involved in inflammatory response | 3/34 | 41/19660 | 7.82E-04 | *C1qa, Il33, Cx3cr1* | neuron death activation microglial | 6.59E-03 |
| Brain | Tan | Age | GO:0048246 | macrophage chemotaxis | 3/34 | 41/19660 | 7.82E-04 | *Csf1, Lgals3, Cx3cr1* | leukocyte granulocyte migration chemotaxis | 1.48E-02 |
| Brain | Tan | Age | GO:0002888 | positive regulation of myeloid leukocyte mediated immunity | 3/34 | 42/19660 | 8.18E-04 | *C3, Itgb2, H2-T23* | mediated immunoglobulin regulation response | 8.06E-03 |
| Brain | Tan | Age | GO:0002366 | leukocyte activation involved in immune response | 5/34 | 238/19660 | 8.18E-04 | *Lgals3, Itgb2, Slc11a1, Il33, Cx3cr1* | leukocyte granulocyte migration chemotaxis | 1.48E-02 |
| Brain | Tan | Age | GO:0002263 | cell activation involved in immune response | 5/34 | 242/19660 | 8.71E-04 | *Lgals3, Itgb2, Slc11a1, Il33, Cx3cr1* | leukocyte granulocyte migration chemotaxis | 1.48E-02 |
| Brain | Tan | Age | GO:0050729 | positive regulation of inflammatory response | 4/34 | 129/19660 | 1.06E-03 | *Ctss, C3, Il33, H2-T23* | mediated immunoglobulin regulation response | 8.06E-03 |
| Brain | Tan | Age | GO:0045665 | negative regulation of neuron differentiation | 5/34 | 254/19660 | 1.06E-03 | *Gfap, H2-K1, H2-D1, B2m, Ctsz* | mediated immunoglobulin regulation response | 8.06E-03 |
| Brain | Tan | Age | GO:0042269 | regulation of natural killer cell mediated cytotoxicity | 3/34 | 47/19660 | 1.07E-03 | *Lag3, Tap2, H2-T23* | mediated immunoglobulin regulation response | 8.06E-03 |
| Brain | Tan | Age | GO:0002698 | negative regulation of immune effector process | 4/34 | 131/19660 | 1.08E-03 | *Lgals3, Tap2, Il33, H2-T23* | mediated immunoglobulin regulation response | 8.06E-03 |
| Brain | Tan | Age | GO:0001819 | positive regulation of cytokine production | 6/34 | 423/19660 | 1.08E-03 | *C3, Irf7, B2m, Slc11a1, Il33, H2-T23* | mediated immunoglobulin regulation response | 8.06E-03 |
| Brain | Tan | Age | GO:0002715 | regulation of natural killer cell mediated immunity | 3/34 | 48/19660 | 1.08E-03 | *Lag3, Tap2, H2-T23* | mediated immunoglobulin regulation response | 8.06E-03 |
| Brain | Tan | Age | GO:0061900 | glial cell activation | 3/34 | 48/19660 | 1.08E-03 | *C1qa, Il33, Cx3cr1* | neuron death activation microglial | 6.59E-03 |
| Brain | Tan | Age | GO:0002683 | negative regulation of immune system process | 6/34 | 426/19660 | 1.10E-03 | *Lgals3, Lag3, Tap2, Il33, C1qc, H2-T23* | mediated immunoglobulin regulation response | 8.06E-03 |
| Brain | Tan | Age | GO:0014009 | glial cell proliferation | 3/34 | 51/19660 | 1.25E-03 | *Gfap, Csf1, Il33* | astrocyte glial proliferation gliogenesis | 1.33E-02 |
| Brain | Tan | Age | GO:1905517 | macrophage migration | 3/34 | 54/19660 | 1.47E-03 | *Csf1, Lgals3, Cx3cr1* | leukocyte granulocyte migration chemotaxis | 1.48E-02 |
| Brain | Tan | Age | GO:0042110 | T cell activation | 6/34 | 456/19660 | 1.53E-03 | *Lgals3, Itgb2, Lag3, B2m, Slc11a1, H2-T23* | mediated immunoglobulin regulation response | 8.06E-03 |
| Brain | Tan | Age | GO:0042742 | defense response to bacterium | 5/34 | 283/19660 | 1.53E-03 | *H2-K1, Lyz2, Gbp3, Slc11a1, H2-T23* | response gram-positive bacterium defense | 1.73E-02 |
| Brain | Tan | Age | GO:0032943 | mononuclear cell proliferation | 5/34 | 285/19660 | 1.56E-03 | *Csf1, Lgals3, Itgb2, Slc11a1, H2-T23* | leukocyte granulocyte migration chemotaxis | 1.48E-02 |
| Brain | Tan | Age | GO:0002579 | positive regulation of antigen processing and presentation | 2/34 | 10/19660 | 1.61E-03 | *Slc11a1, Tap2* | mediated immunoglobulin regulation response | 8.06E-03 |
| Brain | Tan | Age | GO:0042267 | natural killer cell mediated cytotoxicity | 3/34 | 57/19660 | 1.61E-03 | *Lag3, Tap2, H2-T23* | mediated immunoglobulin regulation response | 8.06E-03 |
| Brain | Tan | Age | GO:0002228 | natural killer cell mediated immunity | 3/34 | 59/19660 | 1.74E-03 | *Lag3, Tap2, H2-T23* | mediated immunoglobulin regulation response | 8.06E-03 |
| Brain | Tan | Age | GO:0150076 | neuroinflammatory response | 3/34 | 59/19660 | 1.74E-03 | *C1qa, Il33, Cx3cr1* | neuron death activation microglial | 6.59E-03 |
| Brain | Tan | Age | GO:0050777 | negative regulation of immune response | 4/34 | 156/19660 | 1.76E-03 | *Lgals3, Tap2, Il33, H2-T23* | mediated immunoglobulin regulation response | 8.06E-03 |
| Brain | Tan | Age | GO:0045765 | regulation of angiogenesis | 5/34 | 298/19660 | 1.78E-03 | *C3, Lgals3, Itgb2, Ctsh, Cx3cr1* | leukocyte granulocyte migration chemotaxis | 1.48E-02 |
| Brain | Tan | Age | GO:0002885 | positive regulation of hypersensitivity | 2/34 | 11/19660 | 1.85E-03 | *C3, H2-T23* | mediated immunoglobulin regulation response | 8.06E-03 |
| Brain | Tan | Age | GO:0002886 | regulation of myeloid leukocyte mediated immunity | 3/34 | 62/19660 | 1.92E-03 | *C3, Itgb2, H2-T23* | mediated immunoglobulin regulation response | 8.06E-03 |
| Brain | Tan | Age | GO:0007159 | leukocyte cell-cell adhesion | 5/34 | 308/19660 | 2.00E-03 | *Lgals3, Itgb2, Lag3, Cx3cr1, H2-T23* | leukocyte granulocyte migration chemotaxis | 1.48E-02 |
| Brain | Tan | Age | GO:0051024 | positive regulation of immunoglobulin secretion | 2/34 | 12/19660 | 2.14E-03 | *Il33, H2-T23* | mediated immunoglobulin regulation response | 8.06E-03 |
| Brain | Tan | Age | GO:0042063 | gliogenesis | 5/34 | 315/19660 | 2.15E-03 | *Gfap, Csf1, C1qa, Hexb, Il33* | astrocyte glial proliferation gliogenesis | 1.33E-02 |
| Brain | Tan | Age | GO:0050727 | regulation of inflammatory response | 5/34 | 316/19660 | 2.15E-03 | *Ctss, C3, Il33, Cx3cr1, H2-T23* | mediated immunoglobulin regulation response | 8.06E-03 |
| Brain | Tan | Age | GO:0050900 | leukocyte migration | 5/34 | 316/19660 | 2.15E-03 | *Csf1, Lgals3, Itgb2, Il33, Cx3cr1* | leukocyte granulocyte migration chemotaxis | 1.48E-02 |
| Brain | Tan | Age | GO:0045088 | regulation of innate immune response | 4/34 | 170/19660 | 2.20E-03 | *Irf7, Lag3, Tap2, H2-T23* | mediated immunoglobulin regulation response | 8.06E-03 |
| Brain | Tan | Age | GO:0002866 | positive regulation of acute inflammatory response to antigenic stimulus | 2/34 | 13/19660 | 2.38E-03 | *C3, H2-T23* | mediated immunoglobulin regulation response | 8.06E-03 |
| Brain | Tan | Age | GO:0050768 | negative regulation of neurogenesis | 5/34 | 330/19660 | 2.54E-03 | *Gfap, H2-K1, H2-D1, B2m, Ctsz* | mediated immunoglobulin regulation response | 8.06E-03 |
| Brain | Tan | Age | GO:1901342 | regulation of vasculature development | 5/34 | 331/19660 | 2.55E-03 | *C3, Lgals3, Itgb2, Ctsh, Cx3cr1* | leukocyte granulocyte migration chemotaxis | 1.48E-02 |
| Brain | Tan | Age | GO:0002883 | regulation of hypersensitivity | 2/34 | 14/19660 | 2.66E-03 | *C3, H2-T23* | mediated immunoglobulin regulation response | 8.06E-03 |
| Brain | Tan | Age | GO:0032736 | positive regulation of interleukin-13 production | 2/34 | 14/19660 | 2.66E-03 | *Il33, H2-T23* | mediated immunoglobulin regulation response | 8.06E-03 |
| Brain | Tan | Age | GO:0002281 | macrophage activation involved in immune response | 2/34 | 15/19660 | 2.99E-03 | *Il33, Cx3cr1* | leukocyte granulocyte migration chemotaxis | 1.48E-02 |
| Brain | Tan | Age | GO:0002524 | hypersensitivity | 2/34 | 15/19660 | 2.99E-03 | *C3, H2-T23* | mediated immunoglobulin regulation response | 8.06E-03 |
| Brain | Tan | Age | GO:0022411 | cellular component disassembly | 5/34 | 347/19660 | 2.99E-03 | *Ctss, C3, C1qa, C1qc, C1qb* | mediated immunoglobulin regulation response | 8.06E-03 |
| Brain | Tan | Age | GO:0097529 | myeloid leukocyte migration | 4/34 | 189/19660 | 2.99E-03 | *Csf1, Lgals3, Itgb2, Cx3cr1* | leukocyte granulocyte migration chemotaxis | 1.48E-02 |
| Brain | Tan | Age | GO:0051961 | negative regulation of nervous system development | 5/34 | 353/19660 | 3.18E-03 | *Gfap, H2-K1, H2-D1, B2m, Ctsz* | mediated immunoglobulin regulation response | 8.06E-03 |
| Brain | Tan | Age | GO:0030595 | leukocyte chemotaxis | 4/34 | 193/19660 | 3.18E-03 | *Csf1, Lgals3, Itgb2, Cx3cr1* | leukocyte granulocyte migration chemotaxis | 1.48E-02 |
| Brain | Tan | Age | GO:0002863 | positive regulation of inflammatory response to antigenic stimulus | 2/34 | 16/19660 | 3.22E-03 | *C3, H2-T23* | mediated immunoglobulin regulation response | 8.06E-03 |
| Brain | Tan | Age | GO:0016322 | neuron remodeling | 2/34 | 16/19660 | 3.22E-03 | *C3, C1qa* | mediated immunoglobulin regulation response | 8.06E-03 |
| Brain | Tan | Age | GO:0042098 | T cell proliferation | 4/34 | 198/19660 | 3.35E-03 | *Lgals3, Itgb2, Slc11a1, H2-T23* | leukocyte granulocyte migration chemotaxis | 1.48E-02 |
| Brain | Tan | Age | GO:0002275 | myeloid cell activation involved in immune response | 3/34 | 81/19660 | 3.35E-03 | *Itgb2, Il33, Cx3cr1* | leukocyte granulocyte migration chemotaxis | 1.48E-02 |
| Brain | Tan | Age | GO:0050830 | defense response to Gram-positive bacterium | 3/34 | 81/19660 | 3.35E-03 | *Lyz2, Gbp3, H2-T23* | response gram-positive bacterium defense | 1.73E-02 |
| Brain | Tan | Age | GO:0010721 | negative regulation of cell development | 5/34 | 378/19660 | 4.07E-03 | *Gfap, H2-K1, H2-D1, B2m, Ctsz* | mediated immunoglobulin regulation response | 8.06E-03 |
| Brain | Tan | Age | GO:0002577 | regulation of antigen processing and presentation | 2/34 | 19/19660 | 4.21E-03 | *Slc11a1, Tap2* | mediated immunoglobulin regulation response | 8.06E-03 |
| Brain | Tan | Age | GO:0002864 | regulation of acute inflammatory response to antigenic stimulus | 2/34 | 19/19660 | 4.21E-03 | *C3, H2-T23* | mediated immunoglobulin regulation response | 8.06E-03 |
| Brain | Tan | Age | GO:0032656 | regulation of interleukin-13 production | 2/34 | 19/19660 | 4.21E-03 | *Il33, H2-T23* | mediated immunoglobulin regulation response | 8.06E-03 |
| Brain | Tan | Age | GO:0045953 | negative regulation of natural killer cell mediated cytotoxicity | 2/34 | 19/19660 | 4.21E-03 | *Tap2, H2-T23* | mediated immunoglobulin regulation response | 8.06E-03 |
| Brain | Tan | Age | GO:0051023 | regulation of immunoglobulin secretion | 2/34 | 19/19660 | 4.21E-03 | *Il33, H2-T23* | mediated immunoglobulin regulation response | 8.06E-03 |
| Brain | Tan | Age | GO:0002444 | myeloid leukocyte mediated immunity | 3/34 | 92/19660 | 4.55E-03 | *C3, Itgb2, H2-T23* | mediated immunoglobulin regulation response | 8.06E-03 |
| Brain | Tan | Age | GO:0002716 | negative regulation of natural killer cell mediated immunity | 2/34 | 20/19660 | 4.55E-03 | *Tap2, H2-T23* | mediated immunoglobulin regulation response | 8.06E-03 |
| Brain | Tan | Age | GO:0045649 | regulation of macrophage differentiation | 2/34 | 20/19660 | 4.55E-03 | *Csf1, C1qc* | differentiation cytokine-mediated signaling hemopoiesis | 2.32E-02 |
| Brain | Tan | Age | GO:0002702 | positive regulation of production of molecular mediator of immune response | 3/34 | 93/19660 | 4.59E-03 | *B2m, Il33, H2-T23* | mediated immunoglobulin regulation response | 8.06E-03 |
| Brain | Tan | Age | GO:0002367 | cytokine production involved in immune response | 3/34 | 98/19660 | 5.31E-03 | *B2m, Slc11a1, H2-T23* | mediated immunoglobulin regulation response | 8.06E-03 |
| Brain | Tan | Age | GO:0032616 | interleukin-13 production | 2/34 | 22/19660 | 5.37E-03 | *Il33, H2-T23* | mediated immunoglobulin regulation response | 8.06E-03 |
| Brain | Tan | Age | GO:0002526 | acute inflammatory response | 3/34 | 100/19660 | 5.53E-03 | *C3, Serpina3n, H2-T23* | mediated immunoglobulin regulation response | 8.06E-03 |
| Brain | Tan | Age | GO:0001911 | negative regulation of leukocyte mediated cytotoxicity | 2/34 | 23/19660 | 5.78E-03 | *Tap2, H2-T23* | mediated immunoglobulin regulation response | 8.06E-03 |
| Brain | Tan | Age | GO:0032753 | positive regulation of interleukin-4 production | 2/34 | 24/19660 | 6.22E-03 | *Il33, H2-T23* | mediated immunoglobulin regulation response | 8.06E-03 |
| Brain | Tan | Age | GO:0045089 | positive regulation of innate immune response | 3/34 | 105/19660 | 6.22E-03 | *Irf7, Lag3, H2-T23* | mediated immunoglobulin regulation response | 8.06E-03 |
| Brain | Tan | Age | GO:0048305 | immunoglobulin secretion | 2/34 | 25/19660 | 6.67E-03 | *Il33, H2-T23* | mediated immunoglobulin regulation response | 8.06E-03 |
| Brain | Tan | Age | GO:0071621 | granulocyte chemotaxis | 3/34 | 109/19660 | 6.82E-03 | *Csf1, Lgals3, Itgb2* | leukocyte granulocyte migration chemotaxis | 1.48E-02 |
| Brain | Tan | Age | GO:0001818 | negative regulation of cytokine production | 4/34 | 251/19660 | 6.88E-03 | *Lag3, Slc11a1, Il33, Cx3cr1* | leukocyte granulocyte migration chemotaxis | 1.48E-02 |
| Brain | Tan | Age | GO:0031342 | negative regulation of cell killing | 2/34 | 26/19660 | 7.05E-03 | *Tap2, H2-T23* | mediated immunoglobulin regulation response | 8.06E-03 |
| Brain | Tan | Age | GO:0050808 | synapse organization | 5/34 | 446/19660 | 7.22E-03 | *C3, C1qa, C1qc, Cx3cr1, C1qb* | mediated immunoglobulin regulation response | 8.06E-03 |
| Brain | Tan | Age | GO:0042089 | cytokine biosynthetic process | 3/34 | 113/19660 | 7.33E-03 | *Irf7, Lag3, Cx3cr1* | leukocyte granulocyte migration chemotaxis | 1.48E-02 |
| Brain | Tan | Age | GO:0002438 | acute inflammatory response to antigenic stimulus | 2/34 | 27/19660 | 7.42E-03 | *C3, H2-T23* | mediated immunoglobulin regulation response | 8.06E-03 |
| Brain | Tan | Age | GO:0042107 | cytokine metabolic process | 3/34 | 115/19660 | 7.59E-03 | *Irf7, Lag3, Cx3cr1* | leukocyte granulocyte migration chemotaxis | 1.48E-02 |
| Brain | Tan | Age | GO:0002825 | regulation of T-helper 1 type immune response | 2/34 | 28/19660 | 7.86E-03 | *Slc11a1, Il33* | mediated immunoglobulin regulation response | 8.06E-03 |
| Brain | Tan | Age | GO:0002831 | regulation of response to biotic stimulus | 4/34 | 265/19660 | 7.95E-03 | *Irf7, Lag3, Tap2, H2-T23* | mediated immunoglobulin regulation response | 8.06E-03 |
| Brain | Tan | Age | GO:0002237 | response to molecule of bacterial origin | 4/34 | 266/19660 | 7.95E-03 | *B2m, Slc11a1, Tap2, Cx3cr1* | mediated immunoglobulin regulation response | 8.06E-03 |
| Brain | Tan | Age | GO:0002440 | production of molecular mediator of immune response | 4/34 | 266/19660 | 7.95E-03 | *B2m, Slc11a1, Il33, H2-T23* | mediated immunoglobulin regulation response | 8.06E-03 |
| Brain | Tan | Age | GO:0032673 | regulation of interleukin-4 production | 2/34 | 29/19660 | 8.17E-03 | *Il33, H2-T23* | mediated immunoglobulin regulation response | 8.06E-03 |
| Brain | Tan | Age | GO:0060326 | cell chemotaxis | 4/34 | 269/19660 | 8.17E-03 | *Csf1, Lgals3, Itgb2, Cx3cr1* | leukocyte granulocyte migration chemotaxis | 1.48E-02 |
| Brain | Tan | Age | GO:0097242 | amyloid-beta clearance | 2/34 | 30/19660 | 8.63E-03 | *C3, Itgb2* | leukocyte granulocyte migration chemotaxis | 1.48E-02 |
| Brain | Tan | Age | GO:1903037 | regulation of leukocyte cell-cell adhesion | 4/34 | 277/19660 | 8.89E-03 | *Lgals3, Itgb2, Lag3, H2-T23* | leukocyte granulocyte migration chemotaxis | 1.48E-02 |
| Brain | Tan | Age | GO:0002675 | positive regulation of acute inflammatory response | 2/34 | 31/19660 | 8.89E-03 | *C3, H2-T23* | mediated immunoglobulin regulation response | 8.06E-03 |
| Brain | Tan | Age | GO:0002717 | positive regulation of natural killer cell mediated immunity | 2/34 | 31/19660 | 8.89E-03 | *Lag3, H2-T23* | mediated immunoglobulin regulation response | 8.06E-03 |
| Brain | Tan | Age | GO:0002861 | regulation of inflammatory response to antigenic stimulus | 2/34 | 31/19660 | 8.89E-03 | *C3, H2-T23* | mediated immunoglobulin regulation response | 8.06E-03 |
| Brain | Tan | Age | GO:0045954 | positive regulation of natural killer cell mediated cytotoxicity | 2/34 | 31/19660 | 8.89E-03 | *Lag3, H2-T23* | mediated immunoglobulin regulation response | 8.06E-03 |
| Brain | Tan | Age | GO:0046651 | lymphocyte proliferation | 4/34 | 283/19660 | 9.36E-03 | *Lgals3, Itgb2, Slc11a1, H2-T23* | leukocyte granulocyte migration chemotaxis | 1.48E-02 |
| Brain | Tan | Age | GO:0042832 | defense response to protozoan | 2/34 | 33/19660 | 9.83E-03 | *Gbp3, Slc11a1* | response gram-positive bacterium defense | 1.73E-02 |
| Brain | Tan | Age | GO:0060441 | epithelial tube branching involved in lung morphogenesis | 2/34 | 33/19660 | 9.83E-03 | *Ctsh, Ctsz* | morphogenesis epithelial tube structure | 2.86E-02 |
| Brain | Tan | Age | GO:0097530 | granulocyte migration | 3/34 | 131/19660 | 9.83E-03 | *Csf1, Lgals3, Itgb2* | leukocyte granulocyte migration chemotaxis | 1.48E-02 |
| Brain | Tan | Age | GO:0001525 | angiogenesis | 5/34 | 495/19660 | 9.90E-03 | *C3, Lgals3, Itgb2, Ctsh, Cx3cr1* | leukocyte granulocyte migration chemotaxis | 1.48E-02 |
| Brain | Tan | Age | GO:0002931 | response to ischemia | 2/34 | 34/19660 | 1.03E-02 | *Csf1, Cx3cr1* | leukocyte granulocyte migration chemotaxis | 1.48E-02 |
| Brain | Tan | Age | GO:0002700 | regulation of production of molecular mediator of immune response | 3/34 | 135/19660 | 1.05E-02 | *B2m, Il33, H2-T23* | mediated immunoglobulin regulation response | 8.06E-03 |
| Brain | Tan | Age | GO:0001562 | response to protozoan | 2/34 | 36/19660 | 1.10E-02 | *Gbp3, Slc11a1* | response gram-positive bacterium defense | 1.73E-02 |
| Brain | Tan | Age | GO:0014002 | astrocyte development | 2/34 | 36/19660 | 1.10E-02 | *Gfap, C1qa* | astrocyte glial proliferation gliogenesis | 1.33E-02 |
| Brain | Tan | Age | GO:0030225 | macrophage differentiation | 2/34 | 36/19660 | 1.10E-02 | *Csf1, C1qc* | differentiation cytokine-mediated signaling hemopoiesis | 2.32E-02 |
| Brain | Tan | Age | GO:0032633 | interleukin-4 production | 2/34 | 36/19660 | 1.10E-02 | *Il33, H2-T23* | mediated immunoglobulin regulation response | 8.06E-03 |
| Brain | Tan | Age | GO:0061756 | leukocyte adhesion to vascular endothelial cell | 2/34 | 36/19660 | 1.10E-02 | *Itgb2, Cx3cr1* | leukocyte granulocyte migration chemotaxis | 1.48E-02 |
| Brain | Tan | Age | GO:0002833 | positive regulation of response to biotic stimulus | 3/34 | 141/19660 | 1.14E-02 | *Irf7, Lag3, H2-T23* | mediated immunoglobulin regulation response | 8.06E-03 |
| Brain | Tan | Age | GO:0002714 | positive regulation of B cell mediated immunity | 2/34 | 39/19660 | 1.27E-02 | *C3, H2-T23* | mediated immunoglobulin regulation response | 8.06E-03 |
| Brain | Tan | Age | GO:0002891 | positive regulation of immunoglobulin mediated immune response | 2/34 | 39/19660 | 1.27E-02 | *C3, H2-T23* | mediated immunoglobulin regulation response | 8.06E-03 |
| Brain | Tan | Age | GO:0002369 | T cell cytokine production | 2/34 | 40/19660 | 1.33E-02 | *B2m, Slc11a1* | mediated immunoglobulin regulation response | 8.06E-03 |
| Brain | Tan | Age | GO:0002639 | positive regulation of immunoglobulin production | 2/34 | 42/19660 | 1.44E-02 | *Il33, H2-T23* | mediated immunoglobulin regulation response | 8.06E-03 |
| Brain | Tan | Age | GO:0035458 | cellular response to interferon-beta | 2/34 | 42/19660 | 1.44E-02 | *Ifit3, Gbp3* | response gram-positive bacterium defense | 1.73E-02 |
| Brain | Tan | Age | GO:0001961 | positive regulation of cytokine-mediated signaling pathway | 2/34 | 45/19660 | 1.64E-02 | *Csf1, Irf7* | differentiation cytokine-mediated signaling hemopoiesis | 2.32E-02 |
| Brain | Tan | Age | GO:0002707 | negative regulation of lymphocyte mediated immunity | 2/34 | 46/19660 | 1.69E-02 | *Tap2, H2-T23* | mediated immunoglobulin regulation response | 8.06E-03 |
| Brain | Tan | Age | GO:0042088 | T-helper 1 type immune response | 2/34 | 46/19660 | 1.69E-02 | *Slc11a1, Il33* | mediated immunoglobulin regulation response | 8.06E-03 |
| Brain | Tan | Age | GO:0002673 | regulation of acute inflammatory response | 2/34 | 47/19660 | 1.76E-02 | *C3, H2-T23* | mediated immunoglobulin regulation response | 8.06E-03 |
| Brain | Tan | Age | GO:0006826 | iron ion transport | 2/34 | 48/19660 | 1.81E-02 | *B2m, Slc11a1* | mediated immunoglobulin regulation response | 8.06E-03 |
| Brain | Tan | Age | GO:0071675 | regulation of mononuclear cell migration | 2/34 | 48/19660 | 1.81E-02 | *Csf1, Lgals3* | leukocyte granulocyte migration chemotaxis | 1.48E-02 |
| Brain | Tan | Age | GO:1901214 | regulation of neuron death | 4/34 | 355/19660 | 1.82E-02 | *Csf1, Ctsz, C1qa, Cx3cr1* | neuron death activation microglial | 6.59E-03 |
| Brain | Tan | Age | GO:0048754 | branching morphogenesis of an epithelial tube | 3/34 | 176/19660 | 1.97E-02 | *Csf1, Ctsh, Ctsz* | morphogenesis epithelial tube structure | 2.86E-02 |
| Brain | Tan | Age | GO:0060760 | positive regulation of response to cytokine stimulus | 2/34 | 51/19660 | 2.00E-02 | *Csf1, Irf7* | differentiation cytokine-mediated signaling hemopoiesis | 2.32E-02 |
| Brain | Tan | Age | GO:0002712 | regulation of B cell mediated immunity | 2/34 | 52/19660 | 2.03E-02 | *C3, H2-T23* | mediated immunoglobulin regulation response | 8.06E-03 |
| Brain | Tan | Age | GO:0002720 | positive regulation of cytokine production involved in immune response | 2/34 | 52/19660 | 2.03E-02 | *B2m, H2-T23* | mediated immunoglobulin regulation response | 8.06E-03 |
| Brain | Tan | Age | GO:0002889 | regulation of immunoglobulin mediated immune response | 2/34 | 52/19660 | 2.03E-02 | *C3, H2-T23* | mediated immunoglobulin regulation response | 8.06E-03 |
| Brain | Tan | Age | GO:0035456 | response to interferon-beta | 2/34 | 52/19660 | 2.03E-02 | *Ifit3, Gbp3* | response gram-positive bacterium defense | 1.73E-02 |
| Brain | Tan | Age | GO:1903706 | regulation of hemopoiesis | 4/34 | 373/19660 | 2.08E-02 | *Csf1, Lag3, B2m, C1qc* | differentiation cytokine-mediated signaling hemopoiesis | 2.32E-02 |
| Brain | Tan | Age | GO:0002437 | inflammatory response to antigenic stimulus | 2/34 | 53/19660 | 2.08E-02 | *C3, H2-T23* | mediated immunoglobulin regulation response | 8.06E-03 |
| Brain | Tan | Age | GO:0048260 | positive regulation of receptor-mediated endocytosis | 2/34 | 55/19660 | 2.23E-02 | *C3, B2m* | mediated immunoglobulin regulation response | 8.06E-03 |
| Brain | Tan | Age | GO:0052547 | regulation of peptidase activity | 4/34 | 382/19660 | 2.23E-02 | *Serpina3n, Ctsh, Psmb8, Ctsd* | regulation peptidase activity | 2.23E-02 |
| Brain | Tan | Age | GO:0022407 | regulation of cell-cell adhesion | 4/34 | 383/19660 | 2.24E-02 | *Lgals3, Itgb2, Lag3, H2-T23* | leukocyte granulocyte migration chemotaxis | 1.48E-02 |
| Brain | Tan | Age | GO:0002704 | negative regulation of leukocyte mediated immunity | 2/34 | 56/19660 | 2.26E-02 | *Tap2, H2-T23* | mediated immunoglobulin regulation response | 8.06E-03 |
| Brain | Tan | Age | GO:0042551 | neuron maturation | 2/34 | 56/19660 | 2.26E-02 | *C3, C1qa* | mediated immunoglobulin regulation response | 8.06E-03 |
| Brain | Tan | Age | GO:0045637 | regulation of myeloid cell differentiation | 3/34 | 190/19660 | 2.27E-02 | *Csf1, B2m, C1qc* | differentiation cytokine-mediated signaling hemopoiesis | 2.32E-02 |
| Brain | Tan | Age | GO:0031638 | zymogen activation | 2/34 | 57/19660 | 2.31E-02 | *Ctsh, Ctsz* | morphogenesis epithelial tube structure | 2.86E-02 |
| Brain | Tan | Age | GO:0070997 | neuron death | 4/34 | 390/19660 | 2.33E-02 | *Csf1, Ctsz, C1qa, Cx3cr1* | neuron death activation microglial | 6.59E-03 |
| Brain | Tan | Age | GO:0008347 | glial cell migration | 2/34 | 58/19660 | 2.35E-02 | *Csf1, Hexb* | differentiation cytokine-mediated signaling hemopoiesis | 2.32E-02 |
| Brain | Tan | Age | GO:0002685 | regulation of leukocyte migration | 3/34 | 194/19660 | 2.35E-02 | *Csf1, Lgals3, Il33* | leukocyte granulocyte migration chemotaxis | 1.48E-02 |
| Brain | Tan | Age | GO:0045824 | negative regulation of innate immune response | 2/34 | 59/19660 | 2.42E-02 | *Tap2, H2-T23* | mediated immunoglobulin regulation response | 8.06E-03 |
| Brain | Tan | Age | GO:0045123 | cellular extravasation | 2/34 | 61/19660 | 2.54E-02 | *Itgb2, Cx3cr1* | leukocyte granulocyte migration chemotaxis | 1.48E-02 |
| Brain | Tan | Age | GO:0060425 | lung morphogenesis | 2/34 | 61/19660 | 2.54E-02 | *Ctsh, Ctsz* | morphogenesis epithelial tube structure | 2.86E-02 |
| Brain | Tan | Age | GO:0048839 | inner ear development | 3/34 | 201/19660 | 2.54E-02 | *H2-K1, C1qb, H2-T23* | mediated immunoglobulin regulation response | 8.06E-03 |
| Brain | Tan | Age | GO:0002637 | regulation of immunoglobulin production | 2/34 | 62/19660 | 2.61E-02 | *Il33, H2-T23* | mediated immunoglobulin regulation response | 8.06E-03 |
| Brain | Tan | Age | GO:0045428 | regulation of nitric oxide biosynthetic process | 2/34 | 63/19660 | 2.68E-02 | *Itgb2, Cx3cr1* | leukocyte granulocyte migration chemotaxis | 1.48E-02 |
| Brain | Tan | Age | GO:0030100 | regulation of endocytosis | 3/34 | 210/19660 | 2.79E-02 | *C3, Lgals3, B2m* | mediated immunoglobulin regulation response | 8.06E-03 |
| Brain | Tan | Age | GO:0050829 | defense response to Gram-negative bacterium | 2/34 | 65/19660 | 2.79E-02 | *Lyz2, Slc11a1* | response gram-positive bacterium defense | 1.73E-02 |
| Brain | Tan | Age | GO:0016485 | protein processing | 3/34 | 211/19660 | 2.79E-02 | *Ctss, Ctsh, Ctsz* | morphogenesis epithelial tube structure | 2.86E-02 |
| Brain | Tan | Age | GO:0031348 | negative regulation of defense response | 3/34 | 211/19660 | 2.79E-02 | *Tap2, Cx3cr1, H2-T23* | mediated immunoglobulin regulation response | 8.06E-03 |
| Brain | Tan | Age | GO:0051607 | defense response to virus | 3/34 | 211/19660 | 2.79E-02 | *Irf7, Ifit3, Il33* | response gram-positive bacterium defense | 1.73E-02 |
| Brain | Tan | Age | GO:0061138 | morphogenesis of a branching epithelium | 3/34 | 211/19660 | 2.79E-02 | *Csf1, Ctsh, Ctsz* | morphogenesis epithelial tube structure | 2.86E-02 |
| Brain | Tan | Age | GO:0032623 | interleukin-2 production | 2/34 | 66/19660 | 2.83E-02 | *Lag3, Slc11a1* | mediated immunoglobulin regulation response | 8.06E-03 |
| Brain | Tan | Age | GO:0019915 | lipid storage | 2/34 | 67/19660 | 2.88E-02 | *C3, Hexb* | mediated immunoglobulin regulation response | 8.06E-03 |
| Brain | Tan | Age | GO:2000179 | positive regulation of neural precursor cell proliferation | 2/34 | 67/19660 | 2.88E-02 | *Ctsz, Cx3cr1* | morphogenesis epithelial tube structure | 2.86E-02 |
| Brain | Tan | Age | GO:0006898 | receptor-mediated endocytosis | 3/34 | 218/19660 | 2.98E-02 | *C3, Itgb2, B2m* | mediated immunoglobulin regulation response | 8.06E-03 |
| Brain | Tan | Age | GO:0032944 | regulation of mononuclear cell proliferation | 3/34 | 218/19660 | 2.98E-02 | *Csf1, Lgals3, H2-T23* | leukocyte granulocyte migration chemotaxis | 1.48E-02 |
| Brain | Tan | Age | GO:0097191 | extrinsic apoptotic signaling pathway | 3/34 | 221/19660 | 3.08E-02 | *Lgals3, Il33, Cx3cr1* | leukocyte granulocyte migration chemotaxis | 1.48E-02 |
| Brain | Tan | Age | GO:0006809 | nitric oxide biosynthetic process | 2/34 | 71/19660 | 3.16E-02 | *Itgb2, Cx3cr1* | leukocyte granulocyte migration chemotaxis | 1.48E-02 |
| Brain | Tan | Age | GO:0001894 | tissue homeostasis | 3/34 | 225/19660 | 3.20E-02 | *Ctss, Csf1, Ctsh* | morphogenesis epithelial tube structure | 2.86E-02 |
| Brain | Tan | Age | GO:0071674 | mononuclear cell migration | 2/34 | 72/19660 | 3.21E-02 | *Csf1, Lgals3* | leukocyte granulocyte migration chemotaxis | 1.48E-02 |
| Brain | Tan | Age | GO:0070663 | regulation of leukocyte proliferation | 3/34 | 227/19660 | 3.25E-02 | *Csf1, Lgals3, H2-T23* | leukocyte granulocyte migration chemotaxis | 1.48E-02 |
| Brain | Tan | Age | GO:0050766 | positive regulation of phagocytosis | 2/34 | 73/19660 | 3.27E-02 | *C3, Slc11a1* | mediated immunoglobulin regulation response | 8.06E-03 |
| Brain | Tan | Age | GO:0043583 | ear development | 3/34 | 229/19660 | 3.29E-02 | *H2-K1, C1qb, H2-T23* | mediated immunoglobulin regulation response | 8.06E-03 |
| Brain | Tan | Age | GO:0001763 | morphogenesis of a branching structure | 3/34 | 230/19660 | 3.32E-02 | *Csf1, Ctsh, Ctsz* | morphogenesis epithelial tube structure | 2.86E-02 |
| Brain | Tan | Age | GO:0046209 | nitric oxide metabolic process | 2/34 | 75/19660 | 3.38E-02 | *Itgb2, Cx3cr1* | leukocyte granulocyte migration chemotaxis | 1.48E-02 |
| Brain | Tan | Age | GO:0055072 | iron ion homeostasis | 2/34 | 75/19660 | 3.38E-02 | *B2m, Slc11a1* | mediated immunoglobulin regulation response | 8.06E-03 |
| Brain | Tan | Age | GO:0043393 | regulation of protein binding | 3/34 | 233/19660 | 3.39E-02 | *Lgals3, B2m, Ctsz* | morphogenesis epithelial tube structure | 2.86E-02 |
| Brain | Tan | Age | GO:1903532 | positive regulation of secretion by cell | 4/34 | 455/19660 | 3.41E-02 | *Lgals3, Itgb2, Il33, H2-T23* | leukocyte granulocyte migration chemotaxis | 1.48E-02 |
| Brain | Tan | Age | GO:0009611 | response to wounding | 4/34 | 458/19660 | 3.47E-02 | *Gfap, C3, Neat1, Slc11a1* | mediated immunoglobulin regulation response | 8.06E-03 |
| Brain | Tan | Age | GO:2001057 | reactive nitrogen species metabolic process | 2/34 | 77/19660 | 3.49E-02 | *Itgb2, Cx3cr1* | leukocyte granulocyte migration chemotaxis | 1.48E-02 |
| Brain | Tan | Age | GO:0048871 | multicellular organismal homeostasis | 4/34 | 465/19660 | 3.62E-02 | *Ctss, Csf1, Ctsh, Slc11a1* | morphogenesis epithelial tube structure | 2.86E-02 |
| Brain | Tan | Age | GO:0002718 | regulation of cytokine production involved in immune response | 2/34 | 80/19660 | 3.72E-02 | *B2m, H2-T23* | mediated immunoglobulin regulation response | 8.06E-03 |
| Brain | Tan | Age | GO:0030101 | natural killer cell activation | 2/34 | 81/19660 | 3.79E-02 | *Itgb2, H2-T23* | mediated immunoglobulin regulation response | 8.06E-03 |
| Brain | Tan | Age | GO:0048708 | astrocyte differentiation | 2/34 | 82/19660 | 3.87E-02 | *Gfap, C1qa* | astrocyte glial proliferation gliogenesis | 1.33E-02 |
| Brain | Tan | Age | GO:0009615 | response to virus | 3/34 | 249/19660 | 3.92E-02 | *Irf7, Ifit3, Il33* | response gram-positive bacterium defense | 1.73E-02 |
| Brain | Tan | Age | GO:0014015 | positive regulation of gliogenesis | 2/34 | 84/19660 | 4.01E-02 | *Gfap, Csf1* | differentiation cytokine-mediated signaling hemopoiesis | 2.32E-02 |
| Brain | Tan | Age | GO:0071695 | anatomical structure maturation | 3/34 | 254/19660 | 4.10E-02 | *C3, C1qa, Cx3cr1* | mediated immunoglobulin regulation response | 8.06E-03 |
| Brain | Tan | Age | GO:0030593 | neutrophil chemotaxis | 2/34 | 87/19660 | 4.26E-02 | *Lgals3, Itgb2* | leukocyte granulocyte migration chemotaxis | 1.48E-02 |
| Brain | Tan | Age | GO:0002832 | negative regulation of response to biotic stimulus | 2/34 | 89/19660 | 4.43E-02 | *Tap2, H2-T23* | mediated immunoglobulin regulation response | 8.06E-03 |
| Brain | Tan | Age | GO:0030198 | extracellular matrix organization | 3/34 | 263/19660 | 4.44E-02 | *Gfap, Ctss, Lgals3* | extracellular structure organization matrix | 4.45E-02 |
| Brain | Tan | Age | GO:0043062 | extracellular structure organization | 3/34 | 264/19660 | 4.47E-02 | *Gfap, Ctss, Lgals3* | extracellular structure organization matrix | 4.45E-02 |
| Brain | Tan | Age | GO:0051604 | protein maturation | 3/34 | 265/19660 | 4.49E-02 | *Ctss, Ctsh, Ctsz* | morphogenesis epithelial tube structure | 2.86E-02 |
| Brain | Tan | Age | GO:0006909 | phagocytosis | 3/34 | 269/19660 | 4.66E-02 | *C3, Itgb2, Slc11a1* | mediated immunoglobulin regulation response | 8.06E-03 |
| Brain | Tan | Age | GO:0002286 | T cell activation involved in immune response | 2/34 | 93/19660 | 4.69E-02 | *Lgals3, Slc11a1* | leukocyte granulocyte migration chemotaxis | 1.48E-02 |
| Brain | Tan | Age | GO:2001237 | negative regulation of extrinsic apoptotic signaling pathway | 2/34 | 93/19660 | 4.69E-02 | *Lgals3, Cx3cr1* | leukocyte granulocyte migration chemotaxis | 1.48E-02 |
| Brain | Tan | Age | GO:0000041 | transition metal ion transport | 2/34 | 95/19660 | 4.84E-02 | *B2m, Slc11a1* | mediated immunoglobulin regulation response | 8.06E-03 |
| Brain | Tan | Age | GO:1903426 | regulation of reactive oxygen species biosynthetic process | 2/34 | 95/19660 | 4.84E-02 | *Itgb2, Cx3cr1* | leukocyte granulocyte migration chemotaxis | 1.48E-02 |
| Brain | Tan | Age | GO:1902105 | regulation of leukocyte differentiation | 3/34 | 278/19660 | 4.98E-02 | *Csf1, Lag3, C1qc* | differentiation cytokine-mediated signaling hemopoiesis | 2.32E-02 |
| Brain | Grey60 | Sex | NA | NA | NA | NA | NA | NA | NA | NA |
| Heart | Tan | Age | NA | NA | NA | NA | NA | NA | NA | NA |
| Heart | Blue | Age | GO:0015980 | energy derivation by oxidation of organic compounds | 18/92 | 237/18396 | 1.71E-13 | *Coq9, Slc25a12, Pdhb, Uqcrc1, Cox10, Cyc1, Suclg1, Ndufa10, Fh1, Ppp1ca, Mdh1, Idh3g, Pgm2, Ndufs2, Mdh2, Prkaca, Pygm, Sdhd* | respiratory electron transport ubiquinol | 7.50E-03 |
| Heart | Blue | Age | GO:0045333 | cellular respiration | 15/92 | 150/18396 | 3.99E-13 | *Coq9, Slc25a12, Pdhb, Uqcrc1, Cox10, Cyc1, Suclg1, Ndufa10, Fh1, Mdh1, Idh3g, Ndufs2, Mdh2, Prkaca, Sdhd* | respiratory electron transport ubiquinol | 7.50E-03 |
| Heart | Blue | Age | GO:0006091 | generation of precursor metabolites and energy | 20/92 | 359/18396 | 3.99E-13 | *Eno3, Coq9, Slc25a12, Pdhb, Uqcrc1, Cox10, Cyc1, Suclg1, Ndufa10, Fh1, Ppp1ca, Mdh1, Idh3g, Pgm2, Ndufs2, Mdh2, Prkaca, Pygm, Hmgcl, Sdhd* | respiratory electron transport ubiquinol | 7.50E-03 |
| Heart | Blue | Age | GO:0006099 | tricarboxylic acid cycle | 7/92 | 29/18396 | 3.01E-08 | *Pdhb, Suclg1, Fh1, Mdh1, Idh3g, Mdh2, Sdhd* | respiratory electron transport ubiquinol | 7.50E-03 |
| Heart | Blue | Age | GO:0051186 | cofactor metabolic process | 15/92 | 358/18396 | 7.44E-08 | *Coq9, Isca1, Coq5, Pdhb, Prdx3, Coq2, Nfs1, Cox10, Mccc2, Coq6, Slc25a39, Suclg1, Acaa2, Ndufa9, Hmgcl* | biosynthetic oxidoreduction coenzyme process | 8.62E-04 |
| Heart | Blue | Age | GO:0009060 | aerobic respiration | 8/92 | 67/18396 | 3.33E-07 | *Pdhb, Cox10, Suclg1, Fh1, Mdh1, Idh3g, Mdh2, Sdhd* | respiratory electron transport ubiquinol | 7.50E-03 |
| Heart | Blue | Age | GO:0007005 | mitochondrion organization | 15/92 | 436/18396 | 7.90E-07 | *Tmem70, Chchd3, Prdx3, Map1lc3b, Cox10, Uqcrc2, Samm50, Ndufa10, Acaa2, Poldip2, Ndufa9, Immt, Hmgcl, Phb, Slc25a5* | mitochondrion organization membrane mitochondrial | 4.96E-03 |
| Heart | Blue | Age | GO:0006732 | coenzyme metabolic process | 11/92 | 201/18396 | 8.50E-07 | *Coq9, Coq5, Pdhb, Coq2, Nfs1, Mccc2, Coq6, Suclg1, Acaa2, Ndufa9, Hmgcl* | biosynthetic oxidoreduction coenzyme process | 8.62E-04 |
| Heart | Blue | Age | GO:0051188 | cofactor biosynthetic process | 10/92 | 171/18396 | 2.04E-06 | *Coq9, Isca1, Coq5, Pdhb, Coq2, Nfs1, Cox10, Coq6, Slc25a39, Ndufa9* | biosynthetic oxidoreduction coenzyme process | 8.62E-04 |
| Heart | Blue | Age | GO:0006744 | ubiquinone biosynthetic process | 5/92 | 17/18396 | 2.04E-06 | *Coq9, Coq5, Coq2, Coq6, Ndufa9* | biosynthetic oxidoreduction coenzyme process | 8.62E-04 |
| Heart | Blue | Age | GO:1901663 | quinone biosynthetic process | 5/92 | 17/18396 | 2.04E-06 | *Coq9, Coq5, Coq2, Coq6, Ndufa9* | biosynthetic oxidoreduction coenzyme process | 8.62E-04 |
| Heart | Blue | Age | GO:0006743 | ubiquinone metabolic process | 5/92 | 18/18396 | 2.55E-06 | *Coq9, Coq5, Coq2, Coq6, Ndufa9* | biosynthetic oxidoreduction coenzyme process | 8.62E-04 |
| Heart | Blue | Age | GO:0019395 | fatty acid oxidation | 8/92 | 95/18396 | 2.55E-06 | *Auh, Eci2, Adipor1, Phyh, Etfb, Hadh, Acaa2, Acaa1a* | acid catabolic lipid fatty | 4.19E-04 |
| Heart | Blue | Age | GO:0034440 | lipid oxidation | 8/92 | 97/18396 | 2.80E-06 | *Auh, Eci2, Adipor1, Phyh, Etfb, Hadh, Acaa2, Acaa1a* | acid catabolic lipid fatty | 4.19E-04 |
| Heart | Blue | Age | GO:0022904 | respiratory electron transport chain | 7/92 | 73/18396 | 7.00E-06 | *Coq9, Slc25a12, Uqcrc1, Cyc1, Ndufa10, Ndufs2, Sdhd* | respiratory electron transport ubiquinol | 7.50E-03 |
| Heart | Blue | Age | GO:0022900 | electron transport chain | 7/92 | 77/18396 | 9.53E-06 | *Coq9, Slc25a12, Uqcrc1, Cyc1, Ndufa10, Ndufs2, Sdhd* | respiratory electron transport ubiquinol | 7.50E-03 |
| Heart | Blue | Age | GO:0042775 | mitochondrial ATP synthesis coupled electron transport | 6/92 | 52/18396 | 1.79E-05 | *Coq9, Uqcrc1, Cyc1, Ndufa10, Ndufs2, Sdhd* | respiratory electron transport ubiquinol | 7.50E-03 |
| Heart | Blue | Age | GO:0042773 | ATP synthesis coupled electron transport | 6/92 | 53/18396 | 1.90E-05 | *Coq9, Uqcrc1, Cyc1, Ndufa10, Ndufs2, Sdhd* | respiratory electron transport ubiquinol | 7.50E-03 |
| Heart | Blue | Age | GO:0009062 | fatty acid catabolic process | 7/92 | 93/18396 | 2.92E-05 | *Auh, Eci2, Phyh, Etfb, Hadh, Acaa2, Acaa1a* | acid catabolic lipid fatty | 4.19E-04 |
| Heart | Blue | Age | GO:1901661 | quinone metabolic process | 5/92 | 31/18396 | 2.92E-05 | *Coq9, Coq5, Coq2, Coq6, Ndufa9* | biosynthetic oxidoreduction coenzyme process | 8.62E-04 |
| Heart | Blue | Age | GO:0009108 | coenzyme biosynthetic process | 7/92 | 102/18396 | 5.04E-05 | *Coq9, Coq5, Pdhb, Coq2, Nfs1, Coq6, Ndufa9* | biosynthetic oxidoreduction coenzyme process | 8.62E-04 |
| Heart | Blue | Age | GO:0006635 | fatty acid beta-oxidation | 6/92 | 67/18396 | 6.25E-05 | *Auh, Eci2, Etfb, Hadh, Acaa2, Acaa1a* | acid catabolic lipid fatty | 4.19E-04 |
| Heart | Blue | Age | GO:0016054 | organic acid catabolic process | 9/92 | 211/18396 | 6.25E-05 | *Auh, Eci2, Phyh, Mccc2, Etfb, Hadh, Acaa2, Acaa1a, Hmgcl* | acid catabolic lipid fatty | 4.19E-04 |
| Heart | Blue | Age | GO:0046395 | carboxylic acid catabolic process | 9/92 | 211/18396 | 6.25E-05 | *Auh, Eci2, Phyh, Mccc2, Etfb, Hadh, Acaa2, Acaa1a, Hmgcl* | acid catabolic lipid fatty | 4.19E-04 |
| Heart | Blue | Age | GO:0072329 | monocarboxylic acid catabolic process | 7/92 | 112/18396 | 7.98E-05 | *Auh, Eci2, Phyh, Etfb, Hadh, Acaa2, Acaa1a* | acid catabolic lipid fatty | 4.19E-04 |
| Heart | Blue | Age | GO:0006733 | oxidoreduction coenzyme metabolic process | 5/92 | 42/18396 | 1.04E-04 | *Coq9, Coq5, Coq2, Coq6, Ndufa9* | biosynthetic oxidoreduction coenzyme process | 8.62E-04 |
| Heart | Blue | Age | GO:0042181 | ketone biosynthetic process | 5/92 | 42/18396 | 1.04E-04 | *Coq9, Coq5, Coq2, Coq6, Ndufa9* | biosynthetic oxidoreduction coenzyme process | 8.62E-04 |
| Heart | Blue | Age | GO:0044282 | small molecule catabolic process | 10/92 | 302/18396 | 1.31E-04 | *Auh, Eci2, Phyh, Mccc2, Etfb, Hadh, Acaa2, Pgm2, Acaa1a, Hmgcl* | acid catabolic lipid fatty | 4.19E-04 |
| Heart | Blue | Age | GO:0043648 | dicarboxylic acid metabolic process | 6/92 | 86/18396 | 2.12E-04 | *Phyh, Suclg1, Fh1, Mdh1, Idh3g, Mdh2* | respiratory electron transport ubiquinol | 7.50E-03 |
| Heart | Blue | Age | GO:0006119 | oxidative phosphorylation | 6/92 | 87/18396 | 2.17E-04 | *Coq9, Uqcrc1, Cyc1, Ndufa10, Ndufs2, Sdhd* | respiratory electron transport ubiquinol | 7.50E-03 |
| Heart | Blue | Age | GO:0044242 | cellular lipid catabolic process | 8/92 | 190/18396 | 2.17E-04 | *Auh, Eci2, Phyh, Etfb, Hadh, Acaa2, Acaa1a, Smpd1* | acid catabolic lipid fatty | 4.19E-04 |
| Heart | Blue | Age | GO:0030258 | lipid modification | 8/92 | 209/18396 | 4.23E-04 | *Auh, Eci2, Adipor1, Phyh, Etfb, Hadh, Acaa2, Acaa1a* | acid catabolic lipid fatty | 4.19E-04 |
| Heart | Blue | Age | GO:0006631 | fatty acid metabolic process | 10/92 | 361/18396 | 5.32E-04 | *Gnpat, Auh, Eci2, Ptges2, Adipor1, Phyh, Etfb, Hadh, Acaa2, Acaa1a* | acid catabolic lipid fatty | 4.19E-04 |
| Heart | Blue | Age | GO:0009083 | branched-chain amino acid catabolic process | 3/92 | 15/18396 | 2.05E-03 | *Auh, Mccc2, Hmgcl* | branched-chain amino process metabolic | 1.57E-02 |
| Heart | Blue | Age | GO:0042407 | cristae formation | 3/92 | 15/18396 | 2.05E-03 | *Chchd3, Samm50, Immt* | mitochondrion organization membrane mitochondrial | 4.96E-03 |
| Heart | Blue | Age | GO:0009081 | branched-chain amino acid metabolic process | 3/92 | 16/18396 | 2.44E-03 | *Auh, Mccc2, Hmgcl* | branched-chain amino process metabolic | 1.57E-02 |
| Heart | Blue | Age | GO:0016042 | lipid catabolic process | 8/92 | 286/18396 | 3.42E-03 | *Auh, Eci2, Phyh, Etfb, Hadh, Acaa2, Acaa1a, Smpd1* | acid catabolic lipid fatty | 4.19E-04 |
| Heart | Blue | Age | GO:0033865 | nucleoside bisphosphate metabolic process | 5/92 | 95/18396 | 3.87E-03 | *Pdhb, Mccc2, Suclg1, Acaa2, Hmgcl* | branched-chain amino process metabolic | 1.57E-02 |
| Heart | Blue | Age | GO:0033875 | ribonucleoside bisphosphate metabolic process | 5/92 | 95/18396 | 3.87E-03 | *Pdhb, Mccc2, Suclg1, Acaa2, Hmgcl* | branched-chain amino process metabolic | 1.57E-02 |
| Heart | Blue | Age | GO:0034032 | purine nucleoside bisphosphate metabolic process | 5/92 | 95/18396 | 3.87E-03 | *Pdhb, Mccc2, Suclg1, Acaa2, Hmgcl* | branched-chain amino process metabolic | 1.57E-02 |
| Heart | Blue | Age | GO:0007006 | mitochondrial membrane organization | 5/92 | 96/18396 | 3.97E-03 | *Chchd3, Samm50, Acaa2, Immt, Slc25a5* | mitochondrion organization membrane mitochondrial | 4.96E-03 |
| Heart | Blue | Age | GO:0046034 | ATP metabolic process | 7/92 | 223/18396 | 4.14E-03 | *Eno3, Coq9, Uqcrc1, Cyc1, Ndufa10, Ndufs2, Sdhd* | respiratory electron transport ubiquinol | 7.50E-03 |
| Heart | Blue | Age | GO:0006163 | purine nucleotide metabolic process | 8/92 | 314/18396 | 5.59E-03 | *Eno3, Pdhb, Mccc2, Cyc1, Suclg1, Adk, Acaa2, Hmgcl* | branched-chain amino process metabolic | 1.57E-02 |
| Heart | Blue | Age | GO:0006839 | mitochondrial transport | 6/92 | 177/18396 | 8.09E-03 | *Slc25a12, Uqcrc2, Cyc1, Samm50, Acaa2, Slc25a5* | mitochondrion organization membrane mitochondrial | 4.96E-03 |
| Heart | Blue | Age | GO:0042180 | cellular ketone metabolic process | 6/92 | 182/18396 | 9.19E-03 | *Coq9, Coq5, Coq2, Coq6, Fh1, Ndufa9* | biosynthetic oxidoreduction coenzyme process | 8.62E-04 |
| Heart | Blue | Age | GO:0007007 | inner mitochondrial membrane organization | 3/92 | 28/18396 | 1.07E-02 | *Chchd3, Samm50, Immt* | mitochondrion organization membrane mitochondrial | 4.96E-03 |
| Heart | Blue | Age | GO:0072521 | purine-containing compound metabolic process | 8/92 | 351/18396 | 1.08E-02 | *Eno3, Pdhb, Mccc2, Cyc1, Suclg1, Adk, Acaa2, Hmgcl* | branched-chain amino process metabolic | 1.57E-02 |
| Heart | Blue | Age | GO:0006734 | NADH metabolic process | 3/92 | 29/18396 | 1.14E-02 | *Mdh1, Idh3g, Mdh2* | respiratory electron transport ubiquinol | 7.50E-03 |
| Heart | Blue | Age | GO:0006637 | acyl-CoA metabolic process | 4/92 | 78/18396 | 1.71E-02 | *Pdhb, Suclg1, Acaa2, Hmgcl* | branched-chain amino process metabolic | 1.57E-02 |
| Heart | Blue | Age | GO:0035383 | thioester metabolic process | 4/92 | 78/18396 | 1.71E-02 | *Pdhb, Suclg1, Acaa2, Hmgcl* | branched-chain amino process metabolic | 1.57E-02 |
| Heart | Blue | Age | GO:0009150 | purine ribonucleotide metabolic process | 7/92 | 295/18396 | 1.85E-02 | *Eno3, Pdhb, Mccc2, Cyc1, Suclg1, Acaa2, Hmgcl* | branched-chain amino process metabolic | 1.57E-02 |
| Heart | Blue | Age | GO:0009117 | nucleotide metabolic process | 8/92 | 388/18396 | 1.89E-02 | *Eno3, Pdhb, Mccc2, Cyc1, Suclg1, Adk, Acaa2, Hmgcl* | branched-chain amino process metabolic | 1.57E-02 |
| Heart | Blue | Age | GO:0006753 | nucleoside phosphate metabolic process | 8/92 | 397/18396 | 2.13E-02 | *Eno3, Pdhb, Mccc2, Cyc1, Suclg1, Adk, Acaa2, Hmgcl* | branched-chain amino process metabolic | 1.57E-02 |
| Heart | Blue | Age | GO:0009259 | ribonucleotide metabolic process | 7/92 | 305/18396 | 2.13E-02 | *Eno3, Pdhb, Mccc2, Cyc1, Suclg1, Acaa2, Hmgcl* | branched-chain amino process metabolic | 1.57E-02 |
| Heart | Blue | Age | GO:0090407 | organophosphate biosynthetic process | 8/92 | 401/18396 | 2.22E-02 | *Gnpat, Ckmt2, Pdhb, Ckm, Nfs1, Cyc1, Suclg1, Adk* | branched-chain amino process metabolic | 1.57E-02 |
| Heart | Blue | Age | GO:0031057 | negative regulation of histone modification | 3/92 | 39/18396 | 2.35E-02 | *Ube2b, Fh1, Ctbp1* | negative regulation organelle organization | 3.63E-02 |
| Heart | Blue | Age | GO:0019693 | ribose phosphate metabolic process | 7/92 | 316/18396 | 2.41E-02 | *Eno3, Pdhb, Mccc2, Cyc1, Suclg1, Acaa2, Hmgcl* | branched-chain amino process metabolic | 1.57E-02 |
| Heart | Blue | Age | GO:0006662 | glycerol ether metabolic process | 2/92 | 10/18396 | 2.41E-02 | *Gnpat, Txn2* | glycerol ether metabolic process | 3.01E-02 |
| Heart | Blue | Age | GO:0010288 | response to lead ion | 2/92 | 10/18396 | 2.41E-02 | *Ppp1ca, Ppp5c* | corticosteroid receptor signaling pathway | 3.42E-02 |
| Heart | Blue | Age | GO:0070142 | synaptic vesicle budding | 2/92 | 10/18396 | 2.41E-02 | *Slc2a4, Arf1* | synaptic vesicle budding | 2.41E-02 |
| Heart | Blue | Age | GO:1904851 | positive regulation of establishment of protein localization to telomere | 2/92 | 10/18396 | 2.41E-02 | *Tcp1, Cct5* | regulation establishment protein localization | 3.48E-02 |
| Heart | Blue | Age | GO:0006107 | oxaloacetate metabolic process | 2/92 | 11/18396 | 2.76E-02 | *Mdh1, Mdh2* | respiratory electron transport ubiquinol | 7.50E-03 |
| Heart | Blue | Age | GO:0006122 | mitochondrial electron transport, ubiquinol to cytochrome c | 2/92 | 11/18396 | 2.76E-02 | *Uqcrc1, Cyc1* | respiratory electron transport ubiquinol | 7.50E-03 |
| Heart | Blue | Age | GO:0070203 | regulation of establishment of protein localization to telomere | 2/92 | 11/18396 | 2.76E-02 | *Tcp1, Cct5* | regulation establishment protein localization | 3.48E-02 |
| Heart | Blue | Age | GO:2000322 | regulation of glucocorticoid receptor signaling pathway | 2/92 | 11/18396 | 2.76E-02 | *Ppp5c, Phb* | corticosteroid receptor signaling pathway | 3.42E-02 |
| Heart | Blue | Age | GO:1905268 | negative regulation of chromatin organization | 3/92 | 46/18396 | 3.20E-02 | *Ube2b, Fh1, Ctbp1* | negative regulation organelle organization | 3.63E-02 |
| Heart | Blue | Age | GO:0070202 | regulation of establishment of protein localization to chromosome | 2/92 | 12/18396 | 3.20E-02 | *Tcp1, Cct5* | regulation establishment protein localization | 3.48E-02 |
| Heart | Blue | Age | GO:0006790 | sulfur compound metabolic process | 6/92 | 254/18396 | 3.45E-02 | *Isca1, Pdhb, Nfs1, Suclg1, Acaa2, Hmgcl* | branched-chain amino process metabolic | 1.57E-02 |
| Heart | Blue | Age | GO:0018904 | ether metabolic process | 2/92 | 13/18396 | 3.61E-02 | *Gnpat, Txn2* | glycerol ether metabolic process | 3.01E-02 |
| Heart | Blue | Age | GO:1904816 | positive regulation of protein localization to chromosome, telomeric region | 2/92 | 13/18396 | 3.61E-02 | *Tcp1, Cct5* | regulation establishment protein localization | 3.48E-02 |
| Heart | Blue | Age | GO:0055086 | nucleobase-containing small molecule metabolic process | 8/92 | 456/18396 | 3.87E-02 | *Eno3, Pdhb, Mccc2, Cyc1, Suclg1, Adk, Acaa2, Hmgcl* | branched-chain amino process metabolic | 1.57E-02 |
| Heart | Blue | Age | GO:0042921 | glucocorticoid receptor signaling pathway | 2/92 | 14/18396 | 4.03E-02 | *Ppp5c, Phb* | corticosteroid receptor signaling pathway | 3.42E-02 |
| Heart | Blue | Age | GO:1904814 | regulation of protein localization to chromosome, telomeric region | 2/92 | 14/18396 | 4.03E-02 | *Tcp1, Cct5* | regulation establishment protein localization | 3.48E-02 |
| Heart | Blue | Age | GO:0031115 | negative regulation of microtubule polymerization | 2/92 | 15/18396 | 4.46E-02 | *Fkbp4, Capzb* | negative regulation organelle organization | 3.63E-02 |
| Heart | Blue | Age | GO:0031958 | corticosteroid receptor signaling pathway | 2/92 | 15/18396 | 4.46E-02 | *Ppp5c, Phb* | corticosteroid receptor signaling pathway | 3.42E-02 |
| Heart | Blue | Age | GO:0070936 | protein K48-linked ubiquitination | 3/92 | 54/18396 | 4.46E-02 | *Ube2b, Rnf187, Ube2g2* | protein k48-linked ubiquitination | 4.46E-02 |
| Heart | Blue | Age | GO:0010639 | negative regulation of organelle organization | 7/92 | 370/18396 | 4.50E-02 | *Fkbp4, Ube2b, Fh1, Capzb, Acaa2, Ctbp1, Slc25a5* | negative regulation organelle organization | 3.63E-02 |
| Heart | Blue | Age | GO:0006103 | 2-oxoglutarate metabolic process | 2/92 | 16/18396 | 4.88E-02 | *Phyh, Idh3g* | respiratory electron transport ubiquinol | 7.50E-03 |
| Heart | Blue | Age | GO:0070200 | establishment of protein localization to telomere | 2/92 | 16/18396 | 4.88E-02 | *Tcp1, Cct5* | regulation establishment protein localization | 3.48E-02 |
| Liver | Salmon | Age | GO:0034341 | response to interferon-gamma | 3/5 | 117/15600 | 1.35E-03 | *Ccl5, H2-Eb1, H2-Aa* | regulation assembly cell positive | 3.32E-02 |
| Liver | Salmon | Age | GO:0019886 | antigen processing and presentation of exogenous peptide antigen via MHC class II | 2/5 | 14/15600 | 1.35E-03 | *H2-Eb1, H2-Aa* | regulation assembly cell positive | 3.32E-02 |
| Liver | Salmon | Age | GO:0002495 | antigen processing and presentation of peptide antigen via MHC class II | 2/5 | 17/15600 | 1.35E-03 | *H2-Eb1, H2-Aa* | regulation assembly cell positive | 3.32E-02 |
| Liver | Salmon | Age | GO:0002504 | antigen processing and presentation of peptide or polysaccharide antigen via MHC class II | 2/5 | 18/15600 | 1.35E-03 | *H2-Eb1, H2-Aa* | regulation assembly cell positive | 3.32E-02 |
| Liver | Salmon | Age | GO:0002478 | antigen processing and presentation of exogenous peptide antigen | 2/5 | 25/15600 | 2.11E-03 | *H2-Eb1, H2-Aa* | regulation assembly cell positive | 3.32E-02 |
| Liver | Salmon | Age | GO:0019884 | antigen processing and presentation of exogenous antigen | 2/5 | 31/15600 | 2.72E-03 | *H2-Eb1, H2-Aa* | regulation assembly cell positive | 3.32E-02 |
| Liver | Salmon | Age | GO:0048002 | antigen processing and presentation of peptide antigen | 2/5 | 51/15600 | 6.38E-03 | *H2-Eb1, H2-Aa* | regulation assembly cell positive | 3.32E-02 |
| Liver | Salmon | Age | GO:0014068 | positive regulation of phosphatidylinositol 3-kinase signaling | 2/5 | 61/15600 | 7.01E-03 | *Ntrk2, Ccl5* | regulation assembly cell positive | 3.32E-02 |
| Liver | Salmon | Age | GO:0002250 | adaptive immune response | 3/5 | 393/15600 | 7.01E-03 | *Slamf7, H2-Eb1, H2-Aa* | regulation assembly cell positive | 3.32E-02 |
| Liver | Salmon | Age | GO:0051249 | regulation of lymphocyte activation | 3/5 | 402/15600 | 7.01E-03 | *Ccl5, Slamf7, H2-Aa* | regulation assembly cell positive | 3.32E-02 |
| Liver | Salmon | Age | GO:0002694 | regulation of leukocyte activation | 3/5 | 483/15600 | 1.01E-02 | *Ccl5, Slamf7, H2-Aa* | regulation assembly cell positive | 3.32E-02 |
| Liver | Salmon | Age | GO:0014066 | regulation of phosphatidylinositol 3-kinase signaling | 2/5 | 84/15600 | 1.01E-02 | *Ntrk2, Ccl5* | regulation assembly cell positive | 3.32E-02 |
| Liver | Salmon | Age | GO:0019882 | antigen processing and presentation | 2/5 | 88/15600 | 1.03E-02 | *H2-Eb1, H2-Aa* | regulation assembly cell positive | 3.32E-02 |
| Liver | Salmon | Age | GO:0014065 | phosphatidylinositol 3-kinase signaling | 2/5 | 114/15600 | 1.60E-02 | *Ntrk2, Ccl5* | regulation assembly cell positive | 3.32E-02 |
| Liver | Salmon | Age | GO:0048015 | phosphatidylinositol-mediated signaling | 2/5 | 141/15600 | 1.98E-02 | *Ntrk2, Ccl5* | regulation assembly cell positive | 3.32E-02 |
| Liver | Salmon | Age | GO:1902107 | positive regulation of leukocyte differentiation | 2/5 | 142/15600 | 1.98E-02 | *Ccl5, H2-Aa* | regulation assembly cell positive | 3.32E-02 |
| Liver | Salmon | Age | GO:0042129 | regulation of T cell proliferation | 2/5 | 143/15600 | 1.98E-02 | *Ccl5, H2-Aa* | regulation assembly cell positive | 3.32E-02 |
| Liver | Salmon | Age | GO:0048017 | inositol lipid-mediated signaling | 2/5 | 144/15600 | 1.98E-02 | *Ntrk2, Ccl5* | regulation assembly cell positive | 3.32E-02 |
| Liver | Salmon | Age | GO:0043491 | protein kinase B signaling | 2/5 | 161/15600 | 2.31E-02 | *Ntrk2, Ccl5* | regulation assembly cell positive | 3.32E-02 |
| Liver | Salmon | Age | GO:0050870 | positive regulation of T cell activation | 2/5 | 164/15600 | 2.31E-02 | *Ccl5, H2-Aa* | regulation assembly cell positive | 3.32E-02 |
| Liver | Salmon | Age | GO:1903708 | positive regulation of hemopoiesis | 2/5 | 175/15600 | 2.39E-02 | *Ccl5, H2-Aa* | regulation assembly cell positive | 3.32E-02 |
| Liver | Salmon | Age | GO:0042098 | T cell proliferation | 2/5 | 176/15600 | 2.39E-02 | *Ccl5, H2-Aa* | regulation assembly cell positive | 3.32E-02 |
| Liver | Salmon | Age | GO:1903039 | positive regulation of leukocyte cell-cell adhesion | 2/5 | 179/15600 | 2.39E-02 | *Ccl5, H2-Aa* | regulation assembly cell positive | 3.32E-02 |
| Liver | Salmon | Age | GO:0050670 | regulation of lymphocyte proliferation | 2/5 | 191/15600 | 2.50E-02 | *Ccl5, H2-Aa* | regulation assembly cell positive | 3.32E-02 |
| Liver | Salmon | Age | GO:0032944 | regulation of mononuclear cell proliferation | 2/5 | 193/15600 | 2.50E-02 | *Ccl5, H2-Aa* | regulation assembly cell positive | 3.32E-02 |
| Liver | Salmon | Age | GO:1901215 | negative regulation of neuron death | 2/5 | 195/15600 | 2.50E-02 | *Ntrk2, Ccl5* | regulation assembly cell positive | 3.32E-02 |
| Liver | Salmon | Age | GO:0070663 | regulation of leukocyte proliferation | 2/5 | 200/15600 | 2.53E-02 | *Ccl5, H2-Aa* | regulation assembly cell positive | 3.32E-02 |
| Liver | Salmon | Age | GO:0022409 | positive regulation of cell-cell adhesion | 2/5 | 214/15600 | 2.79E-02 | *Ccl5, H2-Aa* | regulation assembly cell positive | 3.32E-02 |
| Liver | Salmon | Age | GO:0046651 | lymphocyte proliferation | 2/5 | 245/15600 | 2.98E-02 | *Ccl5, H2-Aa* | regulation assembly cell positive | 3.32E-02 |
| Liver | Salmon | Age | GO:0032943 | mononuclear cell proliferation | 2/5 | 247/15600 | 2.98E-02 | *Ccl5, H2-Aa* | regulation assembly cell positive | 3.32E-02 |
| Liver | Salmon | Age | GO:1902105 | regulation of leukocyte differentiation | 2/5 | 248/15600 | 2.98E-02 | *Ccl5, H2-Aa* | regulation assembly cell positive | 3.32E-02 |
| Liver | Salmon | Age | GO:1903037 | regulation of leukocyte cell-cell adhesion | 2/5 | 253/15600 | 2.98E-02 | *Ccl5, H2-Aa* | regulation assembly cell positive | 3.32E-02 |
| Liver | Salmon | Age | GO:0050863 | regulation of T cell activation | 2/5 | 261/15600 | 2.98E-02 | *Ccl5, H2-Aa* | regulation assembly cell positive | 3.32E-02 |
| Liver | Salmon | Age | GO:0070661 | leukocyte proliferation | 2/5 | 262/15600 | 2.98E-02 | *Ccl5, H2-Aa* | regulation assembly cell positive | 3.32E-02 |
| Liver | Salmon | Age | GO:0051251 | positive regulation of lymphocyte activation | 2/5 | 269/15600 | 2.98E-02 | *Ccl5, H2-Aa* | regulation assembly cell positive | 3.32E-02 |
| Liver | Salmon | Age | GO:0043087 | regulation of GTPase activity | 2/5 | 270/15600 | 2.98E-02 | *Ntrk2, Ccl5* | regulation assembly cell positive | 3.32E-02 |
| Liver | Salmon | Age | GO:0007159 | leukocyte cell-cell adhesion | 2/5 | 282/15600 | 2.98E-02 | *Ccl5, H2-Aa* | regulation assembly cell positive | 3.32E-02 |
| Liver | Salmon | Age | GO:0002551 | mast cell chemotaxis | 1/5 | 10/15600 | 2.98E-02 | *Ccl5* | regulation assembly cell positive | 3.32E-02 |
| Liver | Salmon | Age | GO:0019227 | neuronal action potential propagation | 1/5 | 10/15600 | 2.98E-02 | *Ntrk2* | regulation assembly cell positive | 3.32E-02 |
| Liver | Salmon | Age | GO:0031652 | positive regulation of heat generation | 1/5 | 10/15600 | 2.98E-02 | *Ccl5* | regulation assembly cell positive | 3.32E-02 |
| Liver | Salmon | Age | GO:0098870 | action potential propagation | 1/5 | 10/15600 | 2.98E-02 | *Ntrk2* | regulation assembly cell positive | 3.32E-02 |
| Liver | Salmon | Age | GO:0001660 | fever generation | 1/5 | 11/15600 | 2.98E-02 | *Ccl5* | regulation assembly cell positive | 3.32E-02 |
| Liver | Salmon | Age | GO:0043922 | negative regulation by host of viral transcription | 1/5 | 11/15600 | 2.98E-02 | *Ccl5* | regulation assembly cell positive | 3.32E-02 |
| Liver | Salmon | Age | GO:0072683 | T cell extravasation | 1/5 | 11/15600 | 2.98E-02 | *Ccl5* | regulation assembly cell positive | 3.32E-02 |
| Liver | Salmon | Age | GO:0097531 | mast cell migration | 1/5 | 11/15600 | 2.98E-02 | *Ccl5* | regulation assembly cell positive | 3.32E-02 |
| Liver | Salmon | Age | GO:2000322 | regulation of glucocorticoid receptor signaling pathway | 1/5 | 11/15600 | 2.98E-02 | *Ntrk2* | regulation assembly cell positive | 3.32E-02 |
| Liver | Salmon | Age | GO:2000343 | positive regulation of chemokine (C-X-C motif) ligand 2 production | 1/5 | 11/15600 | 2.98E-02 | *Ccl5* | regulation assembly cell positive | 3.32E-02 |
| Liver | Salmon | Age | GO:0002696 | positive regulation of leukocyte activation | 2/5 | 309/15600 | 2.98E-02 | *Ccl5, H2-Aa* | regulation assembly cell positive | 3.32E-02 |
| Liver | Salmon | Age | GO:1901214 | regulation of neuron death | 2/5 | 310/15600 | 2.98E-02 | *Ntrk2, Ccl5* | regulation assembly cell positive | 3.32E-02 |
| Liver | Salmon | Age | GO:0031268 | pseudopodium organization | 1/5 | 12/15600 | 2.98E-02 | *Ccl5* | regulation assembly cell positive | 3.32E-02 |
| Liver | Salmon | Age | GO:0031269 | pseudopodium assembly | 1/5 | 12/15600 | 2.98E-02 | *Ccl5* | regulation assembly cell positive | 3.32E-02 |
| Liver | Salmon | Age | GO:0031650 | regulation of heat generation | 1/5 | 12/15600 | 2.98E-02 | *Ccl5* | regulation assembly cell positive | 3.32E-02 |
| Liver | Salmon | Age | GO:0033632 | regulation of cell-cell adhesion mediated by integrin | 1/5 | 12/15600 | 2.98E-02 | *Ccl5* | regulation assembly cell positive | 3.32E-02 |
| Liver | Salmon | Age | GO:0060221 | retinal rod cell differentiation | 1/5 | 12/15600 | 2.98E-02 | *Ntrk2* | regulation assembly cell positive | 3.32E-02 |
| Liver | Salmon | Age | GO:0050867 | positive regulation of cell activation | 2/5 | 321/15600 | 2.98E-02 | *Ccl5, H2-Aa* | regulation assembly cell positive | 3.32E-02 |
| Liver | Salmon | Age | GO:0010820 | positive regulation of T cell chemotaxis | 1/5 | 13/15600 | 2.98E-02 | *Ccl5* | regulation assembly cell positive | 3.32E-02 |
| Liver | Salmon | Age | GO:0099550 | trans-synaptic signaling, modulating synaptic transmission | 1/5 | 13/15600 | 2.98E-02 | *Ntrk2* | regulation assembly cell positive | 3.32E-02 |
| Liver | Salmon | Age | GO:1903706 | regulation of hemopoiesis | 2/5 | 332/15600 | 2.98E-02 | *Ccl5, H2-Aa* | regulation assembly cell positive | 3.32E-02 |
| Liver | Salmon | Age | GO:0010819 | regulation of T cell chemotaxis | 1/5 | 14/15600 | 2.98E-02 | *Ccl5* | regulation assembly cell positive | 3.32E-02 |
| Liver | Salmon | Age | GO:0042921 | glucocorticoid receptor signaling pathway | 1/5 | 14/15600 | 2.98E-02 | *Ntrk2* | regulation assembly cell positive | 3.32E-02 |
| Liver | Salmon | Age | GO:0048245 | eosinophil chemotaxis | 1/5 | 14/15600 | 2.98E-02 | *Ccl5* | regulation assembly cell positive | 3.32E-02 |
| Liver | Salmon | Age | GO:0070234 | positive regulation of T cell apoptotic process | 1/5 | 14/15600 | 2.98E-02 | *Ccl5* | regulation assembly cell positive | 3.32E-02 |
| Liver | Salmon | Age | GO:0072567 | chemokine (C-X-C motif) ligand 2 production | 1/5 | 14/15600 | 2.98E-02 | *Ccl5* | regulation assembly cell positive | 3.32E-02 |
| Liver | Salmon | Age | GO:2000341 | regulation of chemokine (C-X-C motif) ligand 2 production | 1/5 | 14/15600 | 2.98E-02 | *Ccl5* | regulation assembly cell positive | 3.32E-02 |
| Liver | Salmon | Age | GO:0070997 | neuron death | 2/5 | 343/15600 | 2.98E-02 | *Ntrk2, Ccl5* | regulation assembly cell positive | 3.32E-02 |
| Liver | Salmon | Age | GO:0031958 | corticosteroid receptor signaling pathway | 1/5 | 15/15600 | 2.98E-02 | *Ntrk2* | regulation assembly cell positive | 3.32E-02 |
| Liver | Salmon | Age | GO:0090026 | positive regulation of monocyte chemotaxis | 1/5 | 15/15600 | 2.98E-02 | *Ccl5* | regulation assembly cell positive | 3.32E-02 |
| Liver | Salmon | Age | GO:2000811 | negative regulation of anoikis | 1/5 | 15/15600 | 2.98E-02 | *Ntrk2* | regulation assembly cell positive | 3.32E-02 |
| Liver | Salmon | Age | GO:0022407 | regulation of cell-cell adhesion | 2/5 | 350/15600 | 2.98E-02 | *Ccl5, H2-Aa* | regulation assembly cell positive | 3.32E-02 |
| Liver | Salmon | Age | GO:0033631 | cell-cell adhesion mediated by integrin | 1/5 | 16/15600 | 3.00E-02 | *Ccl5* | regulation assembly cell positive | 3.32E-02 |
| Liver | Salmon | Age | GO:0140131 | positive regulation of lymphocyte chemotaxis | 1/5 | 16/15600 | 3.00E-02 | *Ccl5* | regulation assembly cell positive | 3.32E-02 |
| Liver | Salmon | Age | GO:0045785 | positive regulation of cell adhesion | 2/5 | 370/15600 | 3.00E-02 | *Ccl5, H2-Aa* | regulation assembly cell positive | 3.32E-02 |
| Liver | Salmon | Age | GO:0033033 | negative regulation of myeloid cell apoptotic process | 1/5 | 17/15600 | 3.00E-02 | *Ccl5* | regulation assembly cell positive | 3.32E-02 |
| Liver | Salmon | Age | GO:0033145 | positive regulation of intracellular steroid hormone receptor signaling pathway | 1/5 | 17/15600 | 3.00E-02 | *Ntrk2* | regulation assembly cell positive | 3.32E-02 |
| Liver | Salmon | Age | GO:0035584 | calcium-mediated signaling using intracellular calcium source | 1/5 | 17/15600 | 3.00E-02 | *Ntrk2* | regulation assembly cell positive | 3.32E-02 |
| Liver | Salmon | Age | GO:0072677 | eosinophil migration | 1/5 | 17/15600 | 3.00E-02 | *Ccl5* | regulation assembly cell positive | 3.32E-02 |
| Liver | Salmon | Age | GO:0071407 | cellular response to organic cyclic compound | 2/5 | 377/15600 | 3.00E-02 | *Ntrk2, Ccl5* | regulation assembly cell positive | 3.32E-02 |
| Liver | Salmon | Age | GO:0010759 | positive regulation of macrophage chemotaxis | 1/5 | 18/15600 | 3.00E-02 | *Ccl5* | regulation assembly cell positive | 3.32E-02 |
| Liver | Salmon | Age | GO:0010818 | T cell chemotaxis | 1/5 | 18/15600 | 3.00E-02 | *Ccl5* | regulation assembly cell positive | 3.32E-02 |
| Liver | Salmon | Age | GO:0031649 | heat generation | 1/5 | 18/15600 | 3.00E-02 | *Ccl5* | regulation assembly cell positive | 3.32E-02 |
| Liver | Salmon | Age | GO:0071396 | cellular response to lipid | 2/5 | 388/15600 | 3.00E-02 | *Ntrk2, Ccl5* | regulation assembly cell positive | 3.32E-02 |
| Liver | Salmon | Age | GO:0045666 | positive regulation of neuron differentiation | 2/5 | 394/15600 | 3.00E-02 | *Ntrk2, Ccl5* | regulation assembly cell positive | 3.32E-02 |
| Liver | Salmon | Age | GO:0033630 | positive regulation of cell adhesion mediated by integrin | 1/5 | 19/15600 | 3.00E-02 | *Ccl5* | regulation assembly cell positive | 3.32E-02 |
| Liver | Salmon | Age | GO:0070230 | positive regulation of lymphocyte apoptotic process | 1/5 | 19/15600 | 3.00E-02 | *Ccl5* | regulation assembly cell positive | 3.32E-02 |
| Liver | Salmon | Age | GO:0071677 | positive regulation of mononuclear cell migration | 1/5 | 19/15600 | 3.00E-02 | *Ccl5* | regulation assembly cell positive | 3.32E-02 |
| Liver | Salmon | Age | GO:1901623 | regulation of lymphocyte chemotaxis | 1/5 | 19/15600 | 3.00E-02 | *Ccl5* | regulation assembly cell positive | 3.32E-02 |
| Liver | Salmon | Age | GO:2000209 | regulation of anoikis | 1/5 | 19/15600 | 3.00E-02 | *Ntrk2* | regulation assembly cell positive | 3.32E-02 |
| Liver | Salmon | Age | GO:0090025 | regulation of monocyte chemotaxis | 1/5 | 20/15600 | 3.11E-02 | *Ccl5* | regulation assembly cell positive | 3.32E-02 |
| Liver | Salmon | Age | GO:0042110 | T cell activation | 2/5 | 407/15600 | 3.11E-02 | *Ccl5, H2-Aa* | regulation assembly cell positive | 3.32E-02 |
| Liver | Salmon | Age | GO:0006925 | inflammatory cell apoptotic process | 1/5 | 21/15600 | 3.20E-02 | *Ccl5* | regulation assembly cell positive | 3.32E-02 |
| Liver | Salmon | Age | GO:0032897 | negative regulation of viral transcription | 1/5 | 22/15600 | 3.26E-02 | *Ccl5* | regulation assembly cell positive | 3.32E-02 |
| Liver | Salmon | Age | GO:0070233 | negative regulation of T cell apoptotic process | 1/5 | 22/15600 | 3.26E-02 | *Ccl5* | regulation assembly cell positive | 3.32E-02 |
| Liver | Salmon | Age | GO:0043410 | positive regulation of MAPK cascade | 2/5 | 427/15600 | 3.26E-02 | *Ntrk2, Ccl5* | regulation assembly cell positive | 3.32E-02 |
| Liver | Salmon | Age | GO:0060219 | camera-type eye photoreceptor cell differentiation | 1/5 | 23/15600 | 3.32E-02 | *Ntrk2* | regulation assembly cell positive | 3.32E-02 |
| Liver | Salmon | Age | GO:1905523 | positive regulation of macrophage migration | 1/5 | 23/15600 | 3.32E-02 | *Ccl5* | regulation assembly cell positive | 3.32E-02 |
| Liver | Salmon | Age | GO:0002230 | positive regulation of defense response to virus by host | 1/5 | 24/15600 | 3.43E-02 | *Ccl5* | regulation assembly cell positive | 3.32E-02 |
| Liver | Salmon | Age | GO:0022011 | myelination in peripheral nervous system | 1/5 | 25/15600 | 3.43E-02 | *Ntrk2* | regulation assembly cell positive | 3.32E-02 |
| Liver | Salmon | Age | GO:0032292 | peripheral nervous system axon ensheathment | 1/5 | 25/15600 | 3.43E-02 | *Ntrk2* | regulation assembly cell positive | 3.32E-02 |
| Liver | Salmon | Age | GO:0042119 | neutrophil activation | 1/5 | 25/15600 | 3.43E-02 | *Ccl5* | regulation assembly cell positive | 3.32E-02 |
| Liver | Salmon | Age | GO:0051968 | positive regulation of synaptic transmission, glutamatergic | 1/5 | 25/15600 | 3.43E-02 | *Ntrk2* | regulation assembly cell positive | 3.32E-02 |
| Liver | Salmon | Age | GO:0002675 | positive regulation of acute inflammatory response | 1/5 | 26/15600 | 3.43E-02 | *Ccl5* | regulation assembly cell positive | 3.32E-02 |
| Liver | Salmon | Age | GO:0014047 | glutamate secretion | 1/5 | 26/15600 | 3.43E-02 | *Ntrk2* | regulation assembly cell positive | 3.32E-02 |
| Liver | Salmon | Age | GO:0042462 | eye photoreceptor cell development | 1/5 | 26/15600 | 3.43E-02 | *Ntrk2* | regulation assembly cell positive | 3.32E-02 |
| Liver | Salmon | Age | GO:0045672 | positive regulation of osteoclast differentiation | 1/5 | 26/15600 | 3.43E-02 | *Ccl5* | regulation assembly cell positive | 3.32E-02 |
| Liver | Salmon | Age | GO:0002521 | leukocyte differentiation | 2/5 | 467/15600 | 3.44E-02 | *Ccl5, H2-Aa* | regulation assembly cell positive | 3.32E-02 |
| Liver | Salmon | Age | GO:0010758 | regulation of macrophage chemotaxis | 1/5 | 27/15600 | 3.49E-02 | *Ccl5* | regulation assembly cell positive | 3.32E-02 |
| Liver | Salmon | Age | GO:0014044 | Schwann cell development | 1/5 | 28/15600 | 3.55E-02 | *Ntrk2* | regulation assembly cell positive | 3.32E-02 |
| Liver | Salmon | Age | GO:0034110 | regulation of homotypic cell-cell adhesion | 1/5 | 28/15600 | 3.55E-02 | *Ccl5* | regulation assembly cell positive | 3.32E-02 |
| Liver | Salmon | Age | GO:0033032 | regulation of myeloid cell apoptotic process | 1/5 | 29/15600 | 3.59E-02 | *Ccl5* | regulation assembly cell positive | 3.32E-02 |
| Liver | Salmon | Age | GO:0043276 | anoikis | 1/5 | 29/15600 | 3.59E-02 | *Ntrk2* | regulation assembly cell positive | 3.32E-02 |
| Liver | Salmon | Age | GO:0050769 | positive regulation of neurogenesis | 2/5 | 491/15600 | 3.59E-02 | *Ntrk2, Ccl5* | regulation assembly cell positive | 3.32E-02 |
| Liver | Salmon | Age | GO:0033028 | myeloid cell apoptotic process | 1/5 | 30/15600 | 3.64E-02 | *Ccl5* | regulation assembly cell positive | 3.32E-02 |
| Liver | Salmon | Age | GO:2000108 | positive regulation of leukocyte apoptotic process | 1/5 | 30/15600 | 3.64E-02 | *Ccl5* | regulation assembly cell positive | 3.32E-02 |
| Liver | Salmon | Age | GO:0036230 | granulocyte activation | 1/5 | 31/15600 | 3.69E-02 | *Ccl5* | regulation assembly cell positive | 3.32E-02 |
| Liver | Salmon | Age | GO:2000406 | positive regulation of T cell migration | 1/5 | 31/15600 | 3.69E-02 | *Ccl5* | regulation assembly cell positive | 3.32E-02 |
| Liver | Salmon | Age | GO:0007616 | long-term memory | 1/5 | 32/15600 | 3.75E-02 | *Ntrk2* | regulation assembly cell positive | 3.32E-02 |
| Liver | Salmon | Age | GO:0070229 | negative regulation of lymphocyte apoptotic process | 1/5 | 32/15600 | 3.75E-02 | *Ccl5* | regulation assembly cell positive | 3.32E-02 |
| Liver | Salmon | Age | GO:1990089 | response to nerve growth factor | 1/5 | 33/15600 | 3.78E-02 | *Ntrk2* | regulation assembly cell positive | 3.32E-02 |
| Liver | Salmon | Age | GO:1990090 | cellular response to nerve growth factor stimulus | 1/5 | 33/15600 | 3.78E-02 | *Ntrk2* | regulation assembly cell positive | 3.32E-02 |
| Liver | Salmon | Age | GO:0014037 | Schwann cell differentiation | 1/5 | 34/15600 | 3.78E-02 | *Ntrk2* | regulation assembly cell positive | 3.32E-02 |
| Liver | Salmon | Age | GO:0042531 | positive regulation of tyrosine phosphorylation of STAT protein | 1/5 | 34/15600 | 3.78E-02 | *Ccl5* | regulation assembly cell positive | 3.32E-02 |
| Liver | Salmon | Age | GO:0050691 | regulation of defense response to virus by host | 1/5 | 34/15600 | 3.78E-02 | *Ccl5* | regulation assembly cell positive | 3.32E-02 |
| Liver | Salmon | Age | GO:2000403 | positive regulation of lymphocyte migration | 1/5 | 34/15600 | 3.78E-02 | *Ccl5* | regulation assembly cell positive | 3.32E-02 |
| Liver | Salmon | Age | GO:0006953 | acute-phase response | 1/5 | 35/15600 | 3.83E-02 | *Ccl5* | regulation assembly cell positive | 3.32E-02 |
| Liver | Salmon | Age | GO:0032814 | regulation of natural killer cell activation | 1/5 | 35/15600 | 3.83E-02 | *Slamf7* | regulation assembly cell positive | 3.32E-02 |
| Liver | Salmon | Age | GO:0010518 | positive regulation of phospholipase activity | 1/5 | 36/15600 | 3.88E-02 | *Ccl5* | regulation assembly cell positive | 3.32E-02 |
| Liver | Salmon | Age | GO:0048246 | macrophage chemotaxis | 1/5 | 36/15600 | 3.88E-02 | *Ccl5* | regulation assembly cell positive | 3.32E-02 |
| Liver | Salmon | Age | GO:1905521 | regulation of macrophage migration | 1/5 | 37/15600 | 3.95E-02 | *Ccl5* | regulation assembly cell positive | 3.32E-02 |
| Liver | Salmon | Age | GO:0001754 | eye photoreceptor cell differentiation | 1/5 | 38/15600 | 3.95E-02 | *Ntrk2* | regulation assembly cell positive | 3.32E-02 |
| Liver | Salmon | Age | GO:0002548 | monocyte chemotaxis | 1/5 | 38/15600 | 3.95E-02 | *Ccl5* | regulation assembly cell positive | 3.32E-02 |
| Liver | Salmon | Age | GO:0033628 | regulation of cell adhesion mediated by integrin | 1/5 | 38/15600 | 3.95E-02 | *Ccl5* | regulation assembly cell positive | 3.32E-02 |
| Liver | Salmon | Age | GO:0002673 | regulation of acute inflammatory response | 1/5 | 39/15600 | 3.95E-02 | *Ccl5* | regulation assembly cell positive | 3.32E-02 |
| Liver | Salmon | Age | GO:0046782 | regulation of viral transcription | 1/5 | 39/15600 | 3.95E-02 | *Ccl5* | regulation assembly cell positive | 3.32E-02 |
| Liver | Salmon | Age | GO:0048247 | lymphocyte chemotaxis | 1/5 | 39/15600 | 3.95E-02 | *Ccl5* | regulation assembly cell positive | 3.32E-02 |
| Liver | Salmon | Age | GO:0070232 | regulation of T cell apoptotic process | 1/5 | 39/15600 | 3.95E-02 | *Ccl5* | regulation assembly cell positive | 3.32E-02 |
| Liver | Salmon | Age | GO:0042461 | photoreceptor cell development | 1/5 | 40/15600 | 3.99E-02 | *Ntrk2* | regulation assembly cell positive | 3.32E-02 |
| Liver | Salmon | Age | GO:0071675 | regulation of mononuclear cell migration | 1/5 | 40/15600 | 3.99E-02 | *Ccl5* | regulation assembly cell positive | 3.32E-02 |
| Liver | Salmon | Age | GO:2000404 | regulation of T cell migration | 1/5 | 41/15600 | 4.06E-02 | *Ccl5* | regulation assembly cell positive | 3.32E-02 |
| Liver | Salmon | Age | GO:0003407 | neural retina development | 1/5 | 42/15600 | 4.10E-02 | *Ntrk2* | regulation assembly cell positive | 3.32E-02 |
| Liver | Salmon | Age | GO:0019083 | viral transcription | 1/5 | 42/15600 | 4.10E-02 | *Ccl5* | regulation assembly cell positive | 3.32E-02 |
| Liver | Salmon | Age | GO:0071622 | regulation of granulocyte chemotaxis | 1/5 | 43/15600 | 4.17E-02 | *Ccl5* | regulation assembly cell positive | 3.32E-02 |
| Liver | Salmon | Age | GO:0070098 | chemokine-mediated signaling pathway | 1/5 | 44/15600 | 4.24E-02 | *Ccl5* | regulation assembly cell positive | 3.32E-02 |
| Liver | Salmon | Age | GO:0045744 | negative regulation of G protein-coupled receptor signaling pathway | 1/5 | 46/15600 | 4.34E-02 | *Ccl5* | regulation assembly cell positive | 3.32E-02 |
| Liver | Salmon | Age | GO:0010517 | regulation of phospholipase activity | 1/5 | 47/15600 | 4.34E-02 | *Ccl5* | regulation assembly cell positive | 3.32E-02 |
| Liver | Salmon | Age | GO:0060042 | retina morphogenesis in camera-type eye | 1/5 | 47/15600 | 4.34E-02 | *Ntrk2* | regulation assembly cell positive | 3.32E-02 |
| Liver | Salmon | Age | GO:0007528 | neuromuscular junction development | 1/5 | 48/15600 | 4.34E-02 | *Ntrk2* | regulation assembly cell positive | 3.32E-02 |
| Liver | Salmon | Age | GO:0031663 | lipopolysaccharide-mediated signaling pathway | 1/5 | 48/15600 | 4.34E-02 | *Ccl5* | regulation assembly cell positive | 3.32E-02 |
| Liver | Salmon | Age | GO:0042509 | regulation of tyrosine phosphorylation of STAT protein | 1/5 | 48/15600 | 4.34E-02 | *Ccl5* | regulation assembly cell positive | 3.32E-02 |
| Liver | Salmon | Age | GO:0045071 | negative regulation of viral genome replication | 1/5 | 48/15600 | 4.34E-02 | *Ccl5* | regulation assembly cell positive | 3.32E-02 |
| Liver | Salmon | Age | GO:0060193 | positive regulation of lipase activity | 1/5 | 48/15600 | 4.34E-02 | *Ccl5* | regulation assembly cell positive | 3.32E-02 |
| Liver | Salmon | Age | GO:1905517 | macrophage migration | 1/5 | 48/15600 | 4.34E-02 | *Ccl5* | regulation assembly cell positive | 3.32E-02 |
| Liver | Salmon | Age | GO:0015800 | acidic amino acid transport | 1/5 | 50/15600 | 4.47E-02 | *Ntrk2* | regulation assembly cell positive | 3.32E-02 |
| Liver | Salmon | Age | GO:2000107 | negative regulation of leukocyte apoptotic process | 1/5 | 50/15600 | 4.47E-02 | *Ccl5* | regulation assembly cell positive | 3.32E-02 |
| Liver | Salmon | Age | GO:0002763 | positive regulation of myeloid leukocyte differentiation | 1/5 | 51/15600 | 4.50E-02 | *Ccl5* | regulation assembly cell positive | 3.32E-02 |
| Liver | Salmon | Age | GO:0072678 | T cell migration | 1/5 | 51/15600 | 4.50E-02 | *Ccl5* | regulation assembly cell positive | 3.32E-02 |
| Liver | Salmon | Age | GO:0007260 | tyrosine phosphorylation of STAT protein | 1/5 | 52/15600 | 4.50E-02 | *Ccl5* | regulation assembly cell positive | 3.32E-02 |
| Liver | Salmon | Age | GO:2000401 | regulation of lymphocyte migration | 1/5 | 52/15600 | 4.50E-02 | *Ccl5* | regulation assembly cell positive | 3.32E-02 |
| Liver | Salmon | Age | GO:0032722 | positive regulation of chemokine production | 1/5 | 53/15600 | 4.50E-02 | *Ccl5* | regulation assembly cell positive | 3.32E-02 |
| Liver | Salmon | Age | GO:0007422 | peripheral nervous system development | 1/5 | 54/15600 | 4.50E-02 | *Ntrk2* | regulation assembly cell positive | 3.32E-02 |
| Liver | Salmon | Age | GO:0046530 | photoreceptor cell differentiation | 1/5 | 54/15600 | 4.50E-02 | *Ntrk2* | regulation assembly cell positive | 3.32E-02 |
| Liver | Salmon | Age | GO:0051851 | modulation by host of symbiont process | 1/5 | 54/15600 | 4.50E-02 | *Ccl5* | regulation assembly cell positive | 3.32E-02 |
| Liver | Salmon | Age | GO:0070231 | T cell apoptotic process | 1/5 | 54/15600 | 4.50E-02 | *Ccl5* | regulation assembly cell positive | 3.32E-02 |
| Liver | Salmon | Age | GO:1990868 | response to chemokine | 1/5 | 54/15600 | 4.50E-02 | *Ccl5* | regulation assembly cell positive | 3.32E-02 |
| Liver | Salmon | Age | GO:1990869 | cellular response to chemokine | 1/5 | 54/15600 | 4.50E-02 | *Ccl5* | regulation assembly cell positive | 3.32E-02 |
| Liver | Salmon | Age | GO:0019080 | viral gene expression | 1/5 | 55/15600 | 4.52E-02 | *Ccl5* | regulation assembly cell positive | 3.32E-02 |
| Liver | Salmon | Age | GO:0051966 | regulation of synaptic transmission, glutamatergic | 1/5 | 55/15600 | 4.52E-02 | *Ntrk2* | regulation assembly cell positive | 3.32E-02 |
| Liver | Salmon | Age | GO:0014911 | positive regulation of smooth muscle cell migration | 1/5 | 57/15600 | 4.58E-02 | *Ccl5* | regulation assembly cell positive | 3.32E-02 |
| Liver | Salmon | Age | GO:0019226 | transmission of nerve impulse | 1/5 | 57/15600 | 4.58E-02 | *Ntrk2* | regulation assembly cell positive | 3.32E-02 |
| Liver | Salmon | Age | GO:0045123 | cellular extravasation | 1/5 | 57/15600 | 4.58E-02 | *Ccl5* | regulation assembly cell positive | 3.32E-02 |
| Liver | Salmon | Age | GO:0070228 | regulation of lymphocyte apoptotic process | 1/5 | 57/15600 | 4.58E-02 | *Ccl5* | regulation assembly cell positive | 3.32E-02 |
| Liver | Salmon | Age | GO:0042130 | negative regulation of T cell proliferation | 1/5 | 60/15600 | 4.76E-02 | *H2-Aa* | regulation assembly cell positive | 3.32E-02 |
| Liver | Salmon | Age | GO:0045670 | regulation of osteoclast differentiation | 1/5 | 60/15600 | 4.76E-02 | *Ccl5* | regulation assembly cell positive | 3.32E-02 |
| Liver | Salmon | Age | GO:0033627 | cell adhesion mediated by integrin | 1/5 | 61/15600 | 4.76E-02 | *Ccl5* | regulation assembly cell positive | 3.32E-02 |
| Liver | Salmon | Age | GO:0051965 | positive regulation of synapse assembly | 1/5 | 61/15600 | 4.76E-02 | *Ntrk2* | regulation assembly cell positive | 3.32E-02 |
| Liver | Salmon | Age | GO:0071674 | mononuclear cell migration | 1/5 | 61/15600 | 4.76E-02 | *Ccl5* | regulation assembly cell positive | 3.32E-02 |
| Liver | Salmon | Age | GO:0034109 | homotypic cell-cell adhesion | 1/5 | 62/15600 | 4.80E-02 | *Ccl5* | regulation assembly cell positive | 3.32E-02 |
| Liver | Salmon | Age | GO:0006835 | dicarboxylic acid transport | 1/5 | 63/15600 | 4.80E-02 | *Ntrk2* | regulation assembly cell positive | 3.32E-02 |
| Liver | Salmon | Age | GO:0071230 | cellular response to amino acid stimulus | 1/5 | 63/15600 | 4.80E-02 | *Ntrk2* | regulation assembly cell positive | 3.32E-02 |
| Liver | Salmon | Age | GO:0071347 | cellular response to interleukin-1 | 1/5 | 63/15600 | 4.80E-02 | *Ccl5* | regulation assembly cell positive | 3.32E-02 |
| Liver | Salmon | Age | GO:0046427 | positive regulation of receptor signaling pathway via JAK-STAT | 1/5 | 64/15600 | 4.85E-02 | *Ccl5* | regulation assembly cell positive | 3.32E-02 |
| Liver | Salmon | Age | GO:0007631 | feeding behavior | 1/5 | 65/15600 | 4.90E-02 | *Ntrk2* | regulation assembly cell positive | 3.32E-02 |
| Liver | Salmon | Age | GO:0030101 | natural killer cell activation | 1/5 | 66/15600 | 4.94E-02 | *Slamf7* | regulation assembly cell positive | 3.32E-02 |
| Liver | Salmon | Age | GO:0050688 | regulation of defense response to virus | 1/5 | 67/15600 | 4.96E-02 | *Ccl5* | regulation assembly cell positive | 3.32E-02 |
| Liver | Salmon | Age | GO:0060191 | regulation of lipase activity | 1/5 | 67/15600 | 4.96E-02 | *Ccl5* | regulation assembly cell positive | 3.32E-02 |
| Liver | Darkturquoise | Age | GO:0050853 | B cell receptor signaling pathway | 6/9 | 88/15600 | 3.64E-10 | *Ighm, Igkc, Cd19, Cd79a, Iglc2, Cd79b* | response immune regulation cell | 7.07E-03 |
| Liver | Darkturquoise | Age | GO:0042113 | B cell activation | 7/9 | 257/15600 | 8.61E-10 | *Ighm, Igkc, Cd19, Cd79a, Mzb1, Iglc2, Cd79b* | response immune regulation cell | 7.07E-03 |
| Liver | Darkturquoise | Age | GO:0002250 | adaptive immune response | 7/9 | 393/15600 | 1.06E-08 | *Ighm, Jchain, Igkc, Cd19, Cd79a, Iglc2, Cd79b* | response immune regulation cell | 7.07E-03 |
| Liver | Darkturquoise | Age | GO:0050851 | antigen receptor-mediated signaling pathway | 6/9 | 192/15600 | 1.06E-08 | *Ighm, Igkc, Cd19, Cd79a, Iglc2, Cd79b* | response immune regulation cell | 7.07E-03 |
| Liver | Darkturquoise | Age | GO:0002429 | immune response-activating cell surface receptor signaling pathway | 6/9 | 217/15600 | 1.50E-08 | *Ighm, Igkc, Cd19, Cd79a, Iglc2, Cd79b* | response immune regulation cell | 7.07E-03 |
| Liver | Darkturquoise | Age | GO:0002757 | immune response-activating signal transduction | 6/9 | 218/15600 | 1.50E-08 | *Ighm, Igkc, Cd19, Cd79a, Iglc2, Cd79b* | response immune regulation cell | 7.07E-03 |
| Liver | Darkturquoise | Age | GO:0002768 | immune response-regulating cell surface receptor signaling pathway | 6/9 | 225/15600 | 1.50E-08 | *Ighm, Igkc, Cd19, Cd79a, Iglc2, Cd79b* | response immune regulation cell | 7.07E-03 |
| Liver | Darkturquoise | Age | GO:0002764 | immune response-regulating signaling pathway | 6/9 | 228/15600 | 1.50E-08 | *Ighm, Igkc, Cd19, Cd79a, Iglc2, Cd79b* | response immune regulation cell | 7.07E-03 |
| Liver | Darkturquoise | Age | GO:0030183 | B cell differentiation | 5/9 | 114/15600 | 4.23E-08 | *Ighm, Igkc, Cd19, Cd79a, Cd79b* | response immune regulation cell | 7.07E-03 |
| Liver | Darkturquoise | Age | GO:0002253 | activation of immune response | 6/9 | 293/15600 | 5.41E-08 | *Ighm, Igkc, Cd19, Cd79a, Iglc2, Cd79b* | response immune regulation cell | 7.07E-03 |
| Liver | Darkturquoise | Age | GO:0050864 | regulation of B cell activation | 5/9 | 154/15600 | 1.58E-07 | *Ighm, Igkc, Cd19, Mzb1, Iglc2* | response immune regulation cell | 7.07E-03 |
| Liver | Darkturquoise | Age | GO:0050778 | positive regulation of immune response | 6/9 | 474/15600 | 7.60E-07 | *Ighm, Igkc, Cd19, Cd79a, Iglc2, Cd79b* | response immune regulation cell | 7.07E-03 |
| Liver | Darkturquoise | Age | GO:0042100 | B cell proliferation | 4/9 | 75/15600 | 7.60E-07 | *Ighm, Cd19, Cd79a, Mzb1* | response immune regulation cell | 7.07E-03 |
| Liver | Darkturquoise | Age | GO:0030098 | lymphocyte differentiation | 5/9 | 324/15600 | 5.10E-06 | *Ighm, Igkc, Cd19, Cd79a, Cd79b* | response immune regulation cell | 7.07E-03 |
| Liver | Darkturquoise | Age | GO:0016064 | immunoglobulin mediated immune response | 4/9 | 144/15600 | 9.14E-06 | *Ighm, Igkc, Cd19, Iglc2* | response immune regulation cell | 7.07E-03 |
| Liver | Darkturquoise | Age | GO:0019724 | B cell mediated immunity | 4/9 | 147/15600 | 9.31E-06 | *Ighm, Igkc, Cd19, Iglc2* | response immune regulation cell | 7.07E-03 |
| Liver | Darkturquoise | Age | GO:0051249 | regulation of lymphocyte activation | 5/9 | 402/15600 | 1.22E-05 | *Ighm, Igkc, Cd19, Mzb1, Iglc2* | response immune regulation cell | 7.07E-03 |
| Liver | Darkturquoise | Age | GO:0006959 | humoral immune response | 4/9 | 164/15600 | 1.28E-05 | *Ighm, Jchain, Igkc, Iglc2* | response immune regulation cell | 7.07E-03 |
| Liver | Darkturquoise | Age | GO:0002521 | leukocyte differentiation | 5/9 | 467/15600 | 2.29E-05 | *Ighm, Igkc, Cd19, Cd79a, Cd79b* | response immune regulation cell | 7.07E-03 |
| Liver | Darkturquoise | Age | GO:0042742 | defense response to bacterium | 4/9 | 198/15600 | 2.44E-05 | *Ighm, Jchain, Igkc, Iglc2* | response immune regulation cell | 7.07E-03 |
| Liver | Darkturquoise | Age | GO:0002694 | regulation of leukocyte activation | 5/9 | 483/15600 | 2.44E-05 | *Ighm, Igkc, Cd19, Mzb1, Iglc2* | response immune regulation cell | 7.07E-03 |
| Liver | Darkturquoise | Age | GO:0006910 | phagocytosis, recognition | 3/9 | 56/15600 | 2.67E-05 | *Ighm, Igkc, Iglc2* | response immune regulation cell | 7.07E-03 |
| Liver | Darkturquoise | Age | GO:0046651 | lymphocyte proliferation | 4/9 | 245/15600 | 4.71E-05 | *Ighm, Cd19, Cd79a, Mzb1* | response immune regulation cell | 7.07E-03 |
| Liver | Darkturquoise | Age | GO:0006958 | complement activation, classical pathway | 3/9 | 70/15600 | 4.71E-05 | *Ighm, Igkc, Iglc2* | response immune regulation cell | 7.07E-03 |
| Liver | Darkturquoise | Age | GO:0032943 | mononuclear cell proliferation | 4/9 | 247/15600 | 4.71E-05 | *Ighm, Cd19, Cd79a, Mzb1* | response immune regulation cell | 7.07E-03 |
| Liver | Darkturquoise | Age | GO:0070661 | leukocyte proliferation | 4/9 | 262/15600 | 5.71E-05 | *Ighm, Cd19, Cd79a, Mzb1* | response immune regulation cell | 7.07E-03 |
| Liver | Darkturquoise | Age | GO:0002449 | lymphocyte mediated immunity | 4/9 | 271/15600 | 6.29E-05 | *Ighm, Igkc, Cd19, Iglc2* | response immune regulation cell | 7.07E-03 |
| Liver | Darkturquoise | Age | GO:0002455 | humoral immune response mediated by circulating immunoglobulin | 3/9 | 82/15600 | 6.65E-05 | *Ighm, Igkc, Iglc2* | response immune regulation cell | 7.07E-03 |
| Liver | Darkturquoise | Age | GO:0002460 | adaptive immune response based on somatic recombination of immune receptors built from immunoglobulin superfamily domains | 4/9 | 290/15600 | 7.65E-05 | *Ighm, Igkc, Cd19, Iglc2* | response immune regulation cell | 7.07E-03 |
| Liver | Darkturquoise | Age | GO:0006911 | phagocytosis, engulfment | 3/9 | 91/15600 | 8.22E-05 | *Ighm, Igkc, Iglc2* | response immune regulation cell | 7.07E-03 |
| Liver | Darkturquoise | Age | GO:0006956 | complement activation | 3/9 | 91/15600 | 8.22E-05 | *Ighm, Igkc, Iglc2* | response immune regulation cell | 7.07E-03 |
| Liver | Darkturquoise | Age | GO:0099024 | plasma membrane invagination | 3/9 | 99/15600 | 1.03E-04 | *Ighm, Igkc, Iglc2* | response immune regulation cell | 7.07E-03 |
| Liver | Darkturquoise | Age | GO:0010324 | membrane invagination | 3/9 | 106/15600 | 1.22E-04 | *Ighm, Igkc, Iglc2* | response immune regulation cell | 7.07E-03 |
| Liver | Darkturquoise | Age | GO:0002443 | leukocyte mediated immunity | 4/9 | 343/15600 | 1.26E-04 | *Ighm, Igkc, Cd19, Iglc2* | response immune regulation cell | 7.07E-03 |
| Liver | Darkturquoise | Age | GO:0050871 | positive regulation of B cell activation | 3/9 | 111/15600 | 1.32E-04 | *Ighm, Igkc, Iglc2* | response immune regulation cell | 7.07E-03 |
| Liver | Darkturquoise | Age | GO:0002377 | immunoglobulin production | 3/9 | 134/15600 | 2.26E-04 | *Igkv3-5, Ighm, Mzb1* | response immune regulation cell | 7.07E-03 |
| Liver | Darkturquoise | Age | GO:0008037 | cell recognition | 3/9 | 143/15600 | 2.66E-04 | *Ighm, Igkc, Iglc2* | response immune regulation cell | 7.07E-03 |
| Liver | Darkturquoise | Age | GO:0002440 | production of molecular mediator of immune response | 3/9 | 214/15600 | 8.58E-04 | *Igkv3-5, Ighm, Mzb1* | response immune regulation cell | 7.07E-03 |
| Liver | Darkturquoise | Age | GO:0006909 | phagocytosis | 3/9 | 229/15600 | 1.02E-03 | *Ighm, Igkc, Iglc2* | response immune regulation cell | 7.07E-03 |
| Liver | Darkturquoise | Age | GO:0051251 | positive regulation of lymphocyte activation | 3/9 | 269/15600 | 1.60E-03 | *Ighm, Igkc, Iglc2* | response immune regulation cell | 7.07E-03 |
| Liver | Darkturquoise | Age | GO:0002637 | regulation of immunoglobulin production | 2/9 | 55/15600 | 1.71E-03 | *Ighm, Mzb1* | response immune regulation cell | 7.07E-03 |
| Liver | Darkturquoise | Age | GO:0030888 | regulation of B cell proliferation | 2/9 | 59/15600 | 1.92E-03 | *Ighm, Mzb1* | response immune regulation cell | 7.07E-03 |
| Liver | Darkturquoise | Age | GO:0002696 | positive regulation of leukocyte activation | 3/9 | 309/15600 | 2.23E-03 | *Ighm, Igkc, Iglc2* | response immune regulation cell | 7.07E-03 |
| Liver | Darkturquoise | Age | GO:0050867 | positive regulation of cell activation | 3/9 | 321/15600 | 2.43E-03 | *Ighm, Igkc, Iglc2* | response immune regulation cell | 7.07E-03 |
| Liver | Darkturquoise | Age | GO:0002700 | regulation of production of molecular mediator of immune response | 2/9 | 122/15600 | 7.58E-03 | *Ighm, Mzb1* | response immune regulation cell | 7.07E-03 |
| Liver | Darkturquoise | Age | GO:0006897 | endocytosis | 3/9 | 484/15600 | 7.64E-03 | *Ighm, Igkc, Iglc2* | response immune regulation cell | 7.07E-03 |
| Liver | Darkturquoise | Age | GO:0050670 | regulation of lymphocyte proliferation | 2/9 | 191/15600 | 1.75E-02 | *Ighm, Mzb1* | response immune regulation cell | 7.07E-03 |
| Liver | Darkturquoise | Age | GO:0032944 | regulation of mononuclear cell proliferation | 2/9 | 193/15600 | 1.75E-02 | *Ighm, Mzb1* | response immune regulation cell | 7.07E-03 |
| Liver | Darkturquoise | Age | GO:0070663 | regulation of leukocyte proliferation | 2/9 | 200/15600 | 1.83E-02 | *Ighm, Mzb1* | response immune regulation cell | 7.07E-03 |
| Liver | Darkturquoise | Age | GO:0060263 | regulation of respiratory burst | 1/9 | 13/15600 | 2.42E-02 | *Jchain* | respiratory burst renal filtration | 3.34E-02 |
| Liver | Darkturquoise | Age | GO:0050855 | regulation of B cell receptor signaling pathway | 1/9 | 17/15600 | 3.10E-02 | *Cd19* | response immune regulation cell | 7.07E-03 |
| Liver | Darkturquoise | Age | GO:0019731 | antibacterial humoral response | 1/9 | 18/15600 | 3.22E-02 | *Jchain* | respiratory burst renal filtration | 3.34E-02 |
| Liver | Darkturquoise | Age | GO:0003094 | glomerular filtration | 1/9 | 19/15600 | 3.34E-02 | *Jchain* | respiratory burst renal filtration | 3.34E-02 |
| Liver | Darkturquoise | Age | GO:0097205 | renal filtration | 1/9 | 20/15600 | 3.44E-02 | *Jchain* | respiratory burst renal filtration | 3.34E-02 |
| Liver | Darkturquoise | Age | GO:0032461 | positive regulation of protein oligomerization | 1/9 | 21/15600 | 3.55E-02 | *Jchain* | respiratory burst renal filtration | 3.34E-02 |
| Liver | Darkturquoise | Age | GO:0033622 | integrin activation | 1/9 | 23/15600 | 3.82E-02 | *Mzb1* | response immune regulation cell | 7.07E-03 |
| Liver | Darkturquoise | Age | GO:0002335 | mature B cell differentiation | 1/9 | 25/15600 | 4.07E-02 | *Cd19* | response immune regulation cell | 7.07E-03 |
| Liver | Darkturquoise | Age | GO:0051260 | protein homooligomerization | 2/9 | 330/15600 | 4.07E-02 | *Cd79a, Cd79b* | response immune regulation cell | 7.07E-03 |
| Liver | Darkturquoise | Age | GO:0045730 | respiratory burst | 1/9 | 26/15600 | 4.09E-02 | *Jchain* | respiratory burst renal filtration | 3.34E-02 |
| Liver | Darkturquoise | Age | GO:0002697 | regulation of immune effector process | 2/9 | 340/15600 | 4.11E-02 | *Ighm, Mzb1* | response immune regulation cell | 7.07E-03 |
| Liver | Darkturquoise | Age | GO:0043552 | positive regulation of phosphatidylinositol 3-kinase activity | 1/9 | 27/15600 | 4.11E-02 | *Cd19* | response immune regulation cell | 7.07E-03 |
| Liver | Darkturquoise | Age | GO:0051281 | positive regulation of release of sequestered calcium ion into cytosol | 1/9 | 30/15600 | 4.42E-02 | *Cd19* | response immune regulation cell | 7.07E-03 |
| Liver | Darkturquoise | Age | GO:0090218 | positive regulation of lipid kinase activity | 1/9 | 30/15600 | 4.42E-02 | *Cd19* | response immune regulation cell | 7.07E-03 |
| Liver | Tan | Sex | NA | NA | NA | NA | NA | *NA* | NA | NA |
| Liver | Red | Sex | GO:0051186 | cofactor metabolic process | 4/14 | 347/15600 | 4.81E-02 | *Gstt3, Mpc1, Vnn3, Abcd1* | process metabolic acid fatty | 4.81E-02 |
| Liver | Red | Sex | GO:0006732 | coenzyme metabolic process | 3/14 | 196/15600 | 4.81E-02 | *Mpc1, Vnn3, Abcd1* | process metabolic acid fatty | 4.81E-02 |
| Liver | Red | Sex | GO:0030258 | lipid modification | 3/14 | 197/15600 | 4.81E-02 | *Abcd1, Plpp3, Echs1* | process metabolic acid fatty | 4.81E-02 |
| Liver | Red | Sex | GO:0006790 | sulfur compound metabolic process | 3/14 | 251/15600 | 4.81E-02 | *Gstt3, Mpc1, Abcd1* | process metabolic acid fatty | 4.81E-02 |
| Liver | Red | Sex | GO:0006635 | fatty acid beta-oxidation | 2/14 | 64/15600 | 4.81E-02 | *Abcd1, Echs1* | process metabolic acid fatty | 4.81E-02 |
| Liver | Red | Sex | GO:0016042 | lipid catabolic process | 3/14 | 267/15600 | 4.81E-02 | *Ces1g, Abcd1, Echs1* | process metabolic acid fatty | 4.81E-02 |
| Liver | Red | Sex | GO:0006637 | acyl-CoA metabolic process | 2/14 | 77/15600 | 4.81E-02 | *Mpc1, Abcd1* | process metabolic acid fatty | 4.81E-02 |
| Liver | Red | Sex | GO:0035383 | thioester metabolic process | 2/14 | 77/15600 | 4.81E-02 | *Mpc1, Abcd1* | process metabolic acid fatty | 4.81E-02 |
| Liver | Red | Sex | GO:1903825 | organic acid transmembrane transport | 2/14 | 84/15600 | 4.81E-02 | *Mpc1, Abcd1* | process metabolic acid fatty | 4.81E-02 |
| Liver | Red | Sex | GO:1905039 | carboxylic acid transmembrane transport | 2/14 | 84/15600 | 4.81E-02 | *Mpc1, Abcd1* | process metabolic acid fatty | 4.81E-02 |
| Liver | Red | Sex | GO:0001676 | long-chain fatty acid metabolic process | 2/14 | 85/15600 | 4.81E-02 | *Acad9, Abcd1* | process metabolic acid fatty | 4.81E-02 |
| Liver | Red | Sex | GO:0009062 | fatty acid catabolic process | 2/14 | 90/15600 | 4.81E-02 | *Abcd1, Echs1* | process metabolic acid fatty | 4.81E-02 |
| Liver | Red | Sex | GO:0019395 | fatty acid oxidation | 2/14 | 91/15600 | 4.81E-02 | *Abcd1, Echs1* | process metabolic acid fatty | 4.81E-02 |
| Liver | Red | Sex | GO:0007009 | plasma membrane organization | 2/14 | 92/15600 | 4.81E-02 | *Plscr2, Abcd1* | process metabolic acid fatty | 4.81E-02 |
| Liver | Red | Sex | GO:0034440 | lipid oxidation | 2/14 | 92/15600 | 4.81E-02 | *Abcd1, Echs1* | process metabolic acid fatty | 4.81E-02 |
| Liver | Red | Sex | GO:0072521 | purine-containing compound metabolic process | 3/14 | 336/15600 | 4.81E-02 | *Mpc1, Abcd1, Macrod1* | process metabolic acid fatty | 4.81E-02 |
| Liver | Red | Sex | GO:0033865 | nucleoside bisphosphate metabolic process | 2/14 | 93/15600 | 4.81E-02 | *Mpc1, Abcd1* | process metabolic acid fatty | 4.81E-02 |
| Liver | Red | Sex | GO:0033875 | ribonucleoside bisphosphate metabolic process | 2/14 | 93/15600 | 4.81E-02 | *Mpc1, Abcd1* | process metabolic acid fatty | 4.81E-02 |
| Liver | Red | Sex | GO:0034032 | purine nucleoside bisphosphate metabolic process | 2/14 | 93/15600 | 4.81E-02 | *Mpc1, Abcd1* | process metabolic acid fatty | 4.81E-02 |
| Liver | Red | Sex | GO:0006631 | fatty acid metabolic process | 3/14 | 338/15600 | 4.81E-02 | *Acad9, Abcd1, Echs1* | process metabolic acid fatty | 4.81E-02 |
| Liver | Darkolivegreen | Sex | GO:0006720 | isoprenoid metabolic process | 3/4 | 72/15600 | 3.38E-05 | *Fdft1, Rdh11, Pmvk* | detection visible cellular stimulus | 2.13E-02 |
| Liver | Darkolivegreen | Sex | GO:0008299 | isoprenoid biosynthetic process | 2/4 | 27/15600 | 5.59E-04 | *Fdft1, Pmvk* | process nucleobase-containing metabolic biosynthetic | 1.30E-02 |
| Liver | Darkolivegreen | Sex | GO:0006084 | acetyl-CoA metabolic process | 2/4 | 32/15600 | 5.59E-04 | *Pmvk, Acss2* | process nucleobase-containing metabolic biosynthetic | 1.30E-02 |
| Liver | Darkolivegreen | Sex | GO:0006066 | alcohol metabolic process | 3/4 | 289/15600 | 5.59E-04 | *Fdft1, Rdh11, Pmvk* | detection visible cellular stimulus | 2.13E-02 |
| Liver | Darkolivegreen | Sex | GO:0006695 | cholesterol biosynthetic process | 2/4 | 44/15600 | 7.12E-04 | *Fdft1, Pmvk* | process nucleobase-containing metabolic biosynthetic | 1.30E-02 |
| Liver | Darkolivegreen | Sex | GO:1902653 | secondary alcohol biosynthetic process | 2/4 | 45/15600 | 7.12E-04 | *Fdft1, Pmvk* | process nucleobase-containing metabolic biosynthetic | 1.30E-02 |
| Liver | Darkolivegreen | Sex | GO:0016126 | sterol biosynthetic process | 2/4 | 48/15600 | 7.12E-04 | *Fdft1, Pmvk* | process nucleobase-containing metabolic biosynthetic | 1.30E-02 |
| Liver | Darkolivegreen | Sex | GO:0006721 | terpenoid metabolic process | 2/4 | 55/15600 | 7.76E-04 | *Fdft1, Rdh11* | detection visible cellular stimulus | 2.13E-02 |
| Liver | Darkolivegreen | Sex | GO:1901615 | organic hydroxy compound metabolic process | 3/4 | 423/15600 | 7.76E-04 | *Fdft1, Rdh11, Pmvk* | detection visible cellular stimulus | 2.13E-02 |
| Liver | Darkolivegreen | Sex | GO:0008610 | lipid biosynthetic process | 3/4 | 482/15600 | 1.03E-03 | *Fdft1, Pmvk, Acss2* | process nucleobase-containing metabolic biosynthetic | 1.30E-02 |
| Liver | Darkolivegreen | Sex | GO:0006637 | acyl-CoA metabolic process | 2/4 | 77/15600 | 1.08E-03 | *Pmvk, Acss2* | process nucleobase-containing metabolic biosynthetic | 1.30E-02 |
| Liver | Darkolivegreen | Sex | GO:0035383 | thioester metabolic process | 2/4 | 77/15600 | 1.08E-03 | *Pmvk, Acss2* | process nucleobase-containing metabolic biosynthetic | 1.30E-02 |
| Liver | Darkolivegreen | Sex | GO:0033865 | nucleoside bisphosphate metabolic process | 2/4 | 93/15600 | 1.26E-03 | *Pmvk, Acss2* | process nucleobase-containing metabolic biosynthetic | 1.30E-02 |
| Liver | Darkolivegreen | Sex | GO:0033875 | ribonucleoside bisphosphate metabolic process | 2/4 | 93/15600 | 1.26E-03 | *Pmvk, Acss2* | process nucleobase-containing metabolic biosynthetic | 1.30E-02 |
| Liver | Darkolivegreen | Sex | GO:0034032 | purine nucleoside bisphosphate metabolic process | 2/4 | 93/15600 | 1.26E-03 | *Pmvk, Acss2* | process nucleobase-containing metabolic biosynthetic | 1.30E-02 |
| Liver | Darkolivegreen | Sex | GO:0008203 | cholesterol metabolic process | 2/4 | 112/15600 | 1.71E-03 | *Fdft1, Pmvk* | process nucleobase-containing metabolic biosynthetic | 1.30E-02 |
| Liver | Darkolivegreen | Sex | GO:0016125 | sterol metabolic process | 2/4 | 120/15600 | 1.78E-03 | *Fdft1, Pmvk* | process nucleobase-containing metabolic biosynthetic | 1.30E-02 |
| Liver | Darkolivegreen | Sex | GO:1902652 | secondary alcohol metabolic process | 2/4 | 122/15600 | 1.78E-03 | *Fdft1, Pmvk* | process nucleobase-containing metabolic biosynthetic | 1.30E-02 |
| Liver | Darkolivegreen | Sex | GO:0046165 | alcohol biosynthetic process | 2/4 | 125/15600 | 1.78E-03 | *Fdft1, Pmvk* | process nucleobase-containing metabolic biosynthetic | 1.30E-02 |
| Liver | Darkolivegreen | Sex | GO:0006694 | steroid biosynthetic process | 2/4 | 128/15600 | 1.78E-03 | *Fdft1, Pmvk* | process nucleobase-containing metabolic biosynthetic | 1.30E-02 |
| Liver | Darkolivegreen | Sex | GO:1901617 | organic hydroxy compound biosynthetic process | 2/4 | 193/15600 | 3.79E-03 | *Fdft1, Pmvk* | process nucleobase-containing metabolic biosynthetic | 1.30E-02 |
| Liver | Darkolivegreen | Sex | GO:0006732 | coenzyme metabolic process | 2/4 | 196/15600 | 3.79E-03 | *Pmvk, Acss2* | process nucleobase-containing metabolic biosynthetic | 1.30E-02 |
| Liver | Darkolivegreen | Sex | GO:0006790 | sulfur compound metabolic process | 2/4 | 251/15600 | 5.93E-03 | *Pmvk, Acss2* | process nucleobase-containing metabolic biosynthetic | 1.30E-02 |
| Liver | Darkolivegreen | Sex | GO:0008202 | steroid metabolic process | 2/4 | 257/15600 | 5.95E-03 | *Fdft1, Pmvk* | process nucleobase-containing metabolic biosynthetic | 1.30E-02 |
| Liver | Darkolivegreen | Sex | GO:0009150 | purine ribonucleotide metabolic process | 2/4 | 280/15600 | 6.77E-03 | *Pmvk, Acss2* | process nucleobase-containing metabolic biosynthetic | 1.30E-02 |
| Liver | Darkolivegreen | Sex | GO:0009259 | ribonucleotide metabolic process | 2/4 | 290/15600 | 6.97E-03 | *Pmvk, Acss2* | process nucleobase-containing metabolic biosynthetic | 1.30E-02 |
| Liver | Darkolivegreen | Sex | GO:0006163 | purine nucleotide metabolic process | 2/4 | 299/15600 | 6.97E-03 | *Pmvk, Acss2* | process nucleobase-containing metabolic biosynthetic | 1.30E-02 |
| Liver | Darkolivegreen | Sex | GO:0019693 | ribose phosphate metabolic process | 2/4 | 301/15600 | 6.97E-03 | *Pmvk, Acss2* | process nucleobase-containing metabolic biosynthetic | 1.30E-02 |
| Liver | Darkolivegreen | Sex | GO:0006644 | phospholipid metabolic process | 2/4 | 315/15600 | 7.37E-03 | *Fdft1, Pmvk* | process nucleobase-containing metabolic biosynthetic | 1.30E-02 |
| Liver | Darkolivegreen | Sex | GO:0072521 | purine-containing compound metabolic process | 2/4 | 336/15600 | 8.09E-03 | *Pmvk, Acss2* | process nucleobase-containing metabolic biosynthetic | 1.30E-02 |
| Liver | Darkolivegreen | Sex | GO:0051186 | cofactor metabolic process | 2/4 | 347/15600 | 8.34E-03 | *Pmvk, Acss2* | process nucleobase-containing metabolic biosynthetic | 1.30E-02 |
| Liver | Darkolivegreen | Sex | GO:0009117 | nucleotide metabolic process | 2/4 | 367/15600 | 8.86E-03 | *Pmvk, Acss2* | process nucleobase-containing metabolic biosynthetic | 1.30E-02 |
| Liver | Darkolivegreen | Sex | GO:0042574 | retinal metabolic process | 1/4 | 13/15600 | 8.86E-03 | *Rdh11* | detection visible cellular stimulus | 2.13E-02 |
| Liver | Darkolivegreen | Sex | GO:0006753 | nucleoside phosphate metabolic process | 2/4 | 375/15600 | 8.86E-03 | *Pmvk, Acss2* | process nucleobase-containing metabolic biosynthetic | 1.30E-02 |
| Liver | Darkolivegreen | Sex | GO:0090407 | organophosphate biosynthetic process | 2/4 | 383/15600 | 8.96E-03 | *Pmvk, Acss2* | process nucleobase-containing metabolic biosynthetic | 1.30E-02 |
| Liver | Darkolivegreen | Sex | GO:0046459 | short-chain fatty acid metabolic process | 1/4 | 14/15600 | 8.96E-03 | *Acss2* | process nucleobase-containing metabolic biosynthetic | 1.30E-02 |
| Liver | Darkolivegreen | Sex | GO:0007602 | phototransduction | 1/4 | 15/15600 | 9.34E-03 | *Rdh11* | detection visible cellular stimulus | 2.13E-02 |
| Liver | Darkolivegreen | Sex | GO:0006085 | acetyl-CoA biosynthetic process | 1/4 | 17/15600 | 1.00E-02 | *Acss2* | process nucleobase-containing metabolic biosynthetic | 1.30E-02 |
| Liver | Darkolivegreen | Sex | GO:0017000 | antibiotic biosynthetic process | 1/4 | 17/15600 | 1.00E-02 | *Acss2* | process nucleobase-containing metabolic biosynthetic | 1.30E-02 |
| Liver | Darkolivegreen | Sex | GO:0055086 | nucleobase-containing small molecule metabolic process | 2/4 | 434/15600 | 1.00E-02 | *Pmvk, Acss2* | process nucleobase-containing metabolic biosynthetic | 1.30E-02 |
| Liver | Darkolivegreen | Sex | GO:0009584 | detection of visible light | 1/4 | 19/15600 | 1.07E-02 | *Rdh11* | detection visible cellular stimulus | 2.13E-02 |
| Liver | Darkolivegreen | Sex | GO:0023058 | adaptation of signaling pathway | 1/4 | 21/15600 | 1.15E-02 | *Rdh11* | detection visible cellular stimulus | 2.13E-02 |
| Liver | Darkolivegreen | Sex | GO:0042572 | retinol metabolic process | 1/4 | 22/15600 | 1.18E-02 | *Rdh11* | detection visible cellular stimulus | 2.13E-02 |
| Liver | Darkolivegreen | Sex | GO:0035384 | thioester biosynthetic process | 1/4 | 24/15600 | 1.23E-02 | *Acss2* | process nucleobase-containing metabolic biosynthetic | 1.30E-02 |
| Liver | Darkolivegreen | Sex | GO:0071616 | acyl-CoA biosynthetic process | 1/4 | 24/15600 | 1.23E-02 | *Acss2* | process nucleobase-containing metabolic biosynthetic | 1.30E-02 |
| Liver | Darkolivegreen | Sex | GO:0009583 | detection of light stimulus | 1/4 | 30/15600 | 1.50E-02 | *Rdh11* | detection visible cellular stimulus | 2.13E-02 |
| Liver | Darkolivegreen | Sex | GO:0033866 | nucleoside bisphosphate biosynthetic process | 1/4 | 34/15600 | 1.56E-02 | *Acss2* | process nucleobase-containing metabolic biosynthetic | 1.30E-02 |
| Liver | Darkolivegreen | Sex | GO:0034030 | ribonucleoside bisphosphate biosynthetic process | 1/4 | 34/15600 | 1.56E-02 | *Acss2* | process nucleobase-containing metabolic biosynthetic | 1.30E-02 |
| Liver | Darkolivegreen | Sex | GO:0034033 | purine nucleoside bisphosphate biosynthetic process | 1/4 | 34/15600 | 1.56E-02 | *Acss2* | process nucleobase-containing metabolic biosynthetic | 1.30E-02 |
| Liver | Darkolivegreen | Sex | GO:1990748 | cellular detoxification | 1/4 | 34/15600 | 1.56E-02 | *Rdh11* | detection visible cellular stimulus | 2.13E-02 |
| Liver | Darkolivegreen | Sex | GO:0001523 | retinoid metabolic process | 1/4 | 48/15600 | 2.12E-02 | *Rdh11* | detection visible cellular stimulus | 2.13E-02 |
| Liver | Darkolivegreen | Sex | GO:0098754 | detoxification | 1/4 | 48/15600 | 2.12E-02 | *Rdh11* | detection visible cellular stimulus | 2.13E-02 |
| Liver | Darkolivegreen | Sex | GO:0016101 | diterpenoid metabolic process | 1/4 | 50/15600 | 2.17E-02 | *Rdh11* | detection visible cellular stimulus | 2.13E-02 |
| Liver | Darkolivegreen | Sex | GO:0006081 | cellular aldehyde metabolic process | 1/4 | 54/15600 | 2.30E-02 | *Rdh11* | detection visible cellular stimulus | 2.13E-02 |
| Liver | Darkolivegreen | Sex | GO:0034308 | primary alcohol metabolic process | 1/4 | 56/15600 | 2.34E-02 | *Rdh11* | detection visible cellular stimulus | 2.13E-02 |
| Liver | Darkolivegreen | Sex | GO:0044272 | sulfur compound biosynthetic process | 1/4 | 76/15600 | 3.11E-02 | *Acss2* | process nucleobase-containing metabolic biosynthetic | 1.30E-02 |
| Liver | Darkolivegreen | Sex | GO:0071482 | cellular response to light stimulus | 1/4 | 83/15600 | 3.33E-02 | *Rdh11* | detection visible cellular stimulus | 2.13E-02 |
| Liver | Darkolivegreen | Sex | GO:0009582 | detection of abiotic stimulus | 1/4 | 85/15600 | 3.34E-02 | *Rdh11* | detection visible cellular stimulus | 2.13E-02 |
| Liver | Darkolivegreen | Sex | GO:0009581 | detection of external stimulus | 1/4 | 86/15600 | 3.34E-02 | *Rdh11* | detection visible cellular stimulus | 2.13E-02 |
| Liver | Darkolivegreen | Sex | GO:0034754 | cellular hormone metabolic process | 1/4 | 88/15600 | 3.34E-02 | *Rdh11* | detection visible cellular stimulus | 2.13E-02 |
| Liver | Darkolivegreen | Sex | GO:0016999 | antibiotic metabolic process | 1/4 | 89/15600 | 3.34E-02 | *Acss2* | process nucleobase-containing metabolic biosynthetic | 1.30E-02 |
| Liver | Darkolivegreen | Sex | GO:0007601 | visual perception | 1/4 | 93/15600 | 3.43E-02 | *Rdh11* | detection visible cellular stimulus | 2.13E-02 |
| Liver | Darkolivegreen | Sex | GO:0050953 | sensory perception of light stimulus | 1/4 | 96/15600 | 3.48E-02 | *Rdh11* | detection visible cellular stimulus | 2.13E-02 |
| Liver | Darkolivegreen | Sex | GO:0009108 | coenzyme biosynthetic process | 1/4 | 101/15600 | 3.61E-02 | *Acss2* | process nucleobase-containing metabolic biosynthetic | 1.30E-02 |
| Liver | Darkolivegreen | Sex | GO:0006633 | fatty acid biosynthetic process | 1/4 | 121/15600 | 4.25E-02 | *Acss2* | process nucleobase-containing metabolic biosynthetic | 1.30E-02 |
| Liver | Darkolivegreen | Sex | GO:0009152 | purine ribonucleotide biosynthetic process | 1/4 | 123/15600 | 4.25E-02 | *Acss2* | process nucleobase-containing metabolic biosynthetic | 1.30E-02 |
| Liver | Darkolivegreen | Sex | GO:0009260 | ribonucleotide biosynthetic process | 1/4 | 132/15600 | 4.49E-02 | *Acss2* | process nucleobase-containing metabolic biosynthetic | 1.30E-02 |
| Liver | Darkolivegreen | Sex | GO:0071478 | cellular response to radiation | 1/4 | 136/15600 | 4.49E-02 | *Rdh11* | detection visible cellular stimulus | 2.13E-02 |
| Liver | Darkolivegreen | Sex | GO:0006164 | purine nucleotide biosynthetic process | 1/4 | 137/15600 | 4.49E-02 | *Acss2* | process nucleobase-containing metabolic biosynthetic | 1.30E-02 |
| Liver | Darkolivegreen | Sex | GO:0046390 | ribose phosphate biosynthetic process | 1/4 | 139/15600 | 4.49E-02 | *Acss2* | process nucleobase-containing metabolic biosynthetic | 1.30E-02 |
| Liver | Darkolivegreen | Sex | GO:0097237 | cellular response to toxic substance | 1/4 | 140/15600 | 4.49E-02 | *Rdh11* | detection visible cellular stimulus | 2.13E-02 |
| Liver | Darkolivegreen | Sex | GO:0072522 | purine-containing compound biosynthetic process | 1/4 | 142/15600 | 4.49E-02 | *Acss2* | process nucleobase-containing metabolic biosynthetic | 1.30E-02 |
| Liver | Darkolivegreen | Sex | GO:0051606 | detection of stimulus | 1/4 | 146/15600 | 4.55E-02 | *Rdh11* | detection visible cellular stimulus | 2.13E-02 |
| Liver | Darkolivegreen | Sex | GO:0008654 | phospholipid biosynthetic process | 1/4 | 151/15600 | 4.64E-02 | *Pmvk* | process nucleobase-containing metabolic biosynthetic | 1.30E-02 |
| Liver | Darkgrey | Sex | NA | NA | NA | NA | NA | *NA* | NA | NA |
| Liver | Cyan | Sex | GO:0006888 | endoplasmic reticulum to Golgi vesicle-mediated transport | 4/11 | 115/15600 | 1.88E-04 | *Sec22b, Copg1, Arcn1, Sec24d* | endoplasmic reticulum golgi vesicle-mediated | 2.80E-03 |
| Liver | Cyan | Sex | GO:0048193 | Golgi vesicle transport | 4/11 | 250/15600 | 1.75E-03 | *Sec22b, Copg1, Arcn1, Sec24d* | endoplasmic reticulum golgi vesicle-mediated | 2.80E-03 |
| Liver | Cyan | Sex | GO:0006620 | posttranslational protein targeting to endoplasmic reticulum membrane | 2/11 | 11/15600 | 1.75E-03 | *Sec61a1, Hspa5* | protein transmembrane response development | 1.41E-02 |
| Liver | Cyan | Sex | GO:0035967 | cellular response to topologically incorrect protein | 3/11 | 95/15600 | 1.85E-03 | *Serp1, Sdf2l1, Hspa5* | protein transmembrane response development | 1.41E-02 |
| Liver | Cyan | Sex | GO:0051645 | Golgi localization | 2/11 | 15/15600 | 2.00E-03 | *Copg1, Arcn1* | endoplasmic reticulum golgi vesicle-mediated | 2.80E-03 |
| Liver | Cyan | Sex | GO:0035966 | response to topologically incorrect protein | 3/11 | 124/15600 | 2.73E-03 | *Serp1, Sdf2l1, Hspa5* | protein transmembrane response development | 1.41E-02 |
| Liver | Cyan | Sex | GO:0021680 | cerebellar Purkinje cell layer development | 2/11 | 24/15600 | 3.75E-03 | *Arcn1, Hspa5* | protein transmembrane response development | 1.41E-02 |
| Liver | Cyan | Sex | GO:0045047 | protein targeting to ER | 2/11 | 28/15600 | 4.48E-03 | *Sec61a1, Hspa5* | protein transmembrane response development | 1.41E-02 |
| Liver | Cyan | Sex | GO:0072599 | establishment of protein localization to endoplasmic reticulum | 2/11 | 32/15600 | 5.22E-03 | *Sec61a1, Hspa5* | protein transmembrane response development | 1.41E-02 |
| Liver | Cyan | Sex | GO:0006890 | retrograde vesicle-mediated transport, Golgi to endoplasmic reticulum | 2/11 | 43/15600 | 7.27E-03 | *Sec22b, Arcn1* | endoplasmic reticulum golgi vesicle-mediated | 2.80E-03 |
| Liver | Cyan | Sex | GO:0034976 | response to endoplasmic reticulum stress | 3/11 | 220/15600 | 7.27E-03 | *Serp1, Sdf2l1, Hspa5* | protein transmembrane response development | 1.41E-02 |
| Liver | Cyan | Sex | GO:0021695 | cerebellar cortex development | 2/11 | 44/15600 | 7.27E-03 | *Arcn1, Hspa5* | protein transmembrane response development | 1.41E-02 |
| Liver | Cyan | Sex | GO:0065002 | intracellular protein transmembrane transport | 2/11 | 46/15600 | 7.27E-03 | *Sec61a1, Hspa5* | protein transmembrane response development | 1.41E-02 |
| Liver | Cyan | Sex | GO:0071806 | protein transmembrane transport | 2/11 | 47/15600 | 7.27E-03 | *Sec61a1, Hspa5* | protein transmembrane response development | 1.41E-02 |
| Liver | Cyan | Sex | GO:0070972 | protein localization to endoplasmic reticulum | 2/11 | 54/15600 | 8.96E-03 | *Sec61a1, Hspa5* | protein transmembrane response development | 1.41E-02 |
| Liver | Cyan | Sex | GO:0030968 | endoplasmic reticulum unfolded protein response | 2/11 | 59/15600 | 1.00E-02 | *Serp1, Hspa5* | protein transmembrane response development | 1.41E-02 |
| Liver | Cyan | Sex | GO:0021549 | cerebellum development | 2/11 | 74/15600 | 1.40E-02 | *Arcn1, Hspa5* | protein transmembrane response development | 1.41E-02 |
| Liver | Cyan | Sex | GO:0034620 | cellular response to unfolded protein | 2/11 | 74/15600 | 1.40E-02 | *Serp1, Hspa5* | protein transmembrane response development | 1.41E-02 |
| Liver | Cyan | Sex | GO:0022037 | metencephalon development | 2/11 | 80/15600 | 1.55E-02 | *Arcn1, Hspa5* | protein transmembrane response development | 1.41E-02 |
| Liver | Cyan | Sex | GO:0006612 | protein targeting to membrane | 2/11 | 92/15600 | 1.85E-02 | *Sec61a1, Hspa5* | protein transmembrane response development | 1.41E-02 |
| Liver | Cyan | Sex | GO:0036503 | ERAD pathway | 2/11 | 92/15600 | 1.85E-02 | *Sdf2l1, Hspa5* | protein transmembrane response development | 1.41E-02 |
| Liver | Cyan | Sex | GO:0006986 | response to unfolded protein | 2/11 | 101/15600 | 2.12E-02 | *Serp1, Hspa5* | protein transmembrane response development | 1.41E-02 |
| Liver | Cyan | Sex | GO:0030902 | hindbrain development | 2/11 | 118/15600 | 2.75E-02 | *Arcn1, Hspa5* | protein transmembrane response development | 1.41E-02 |
| Liver | Cyan | Sex | GO:0006486 | protein glycosylation | 2/11 | 167/15600 | 4.99E-02 | *Serp1, Sdf2l1* | protein transmembrane response development | 1.41E-02 |
| Liver | Cyan | Sex | GO:0043413 | macromolecule glycosylation | 2/11 | 167/15600 | 4.99E-02 | *Serp1, Sdf2l1* | protein transmembrane response development | 1.41E-02 |
| Liver | Brown | Sex | GO:0017144 | drug metabolic process | 10/34 | 458/15600 | 2.05E-05 | *Fmo3, Cyp2c38, Maob, Cyp2c39, Sult1a1, Fmo4, Esr1, Hao2, Cyp2a22, Cyp2g1* | drug process metabolic acid | 1.70E-03 |
| Liver | Brown | Sex | GO:0019373 | epoxygenase P450 pathway | 4/34 | 28/15600 | 1.27E-04 | *Cyp2c38, Cyp2c39, Cyp2a22, Cyp2g1* | drug process metabolic acid | 1.70E-03 |
| Liver | Brown | Sex | GO:0006805 | xenobiotic metabolic process | 5/34 | 102/15600 | 4.22E-04 | *Cyp2c38, Cyp2c39, Sult1a1, Cyp2a22, Cyp2g1* | drug process metabolic acid | 1.70E-03 |
| Liver | Brown | Sex | GO:0042738 | exogenous drug catabolic process | 4/34 | 47/15600 | 4.22E-04 | *Cyp2c38, Cyp2c39, Cyp2a22, Cyp2g1* | drug process metabolic acid | 1.70E-03 |
| Liver | Brown | Sex | GO:0051923 | sulfation | 3/34 | 14/15600 | 4.22E-04 | *Sult1a1, Sult2a1, Sult3a1* | steroid metabolic process sulfation | 8.64E-04 |
| Liver | Brown | Sex | GO:0019369 | arachidonic acid metabolic process | 4/34 | 49/15600 | 4.22E-04 | *Cyp2c38, Cyp2c39, Cyp2a22, Cyp2g1* | drug process metabolic acid | 1.70E-03 |
| Liver | Brown | Sex | GO:0042737 | drug catabolic process | 5/34 | 129/15600 | 8.00E-04 | *Cyp2c38, Cyp2c39, Fmo4, Cyp2a22, Cyp2g1* | drug process metabolic acid | 1.70E-03 |
| Liver | Brown | Sex | GO:0071466 | cellular response to xenobiotic stimulus | 5/34 | 136/15600 | 9.06E-04 | *Cyp2c38, Cyp2c39, Sult1a1, Cyp2a22, Cyp2g1* | drug process metabolic acid | 1.70E-03 |
| Liver | Brown | Sex | GO:0008202 | steroid metabolic process | 6/34 | 257/15600 | 1.30E-03 | *Sult1a1, Sult2a2, Vldlr, Esr1, Sult2a1, Cyp3a41a* | steroid metabolic process sulfation | 8.64E-04 |
| Liver | Brown | Sex | GO:0001676 | long-chain fatty acid metabolic process | 4/34 | 85/15600 | 2.28E-03 | *Cyp2c38, Cyp2c39, Cyp2a22, Cyp2g1* | drug process metabolic acid | 1.70E-03 |
| Liver | Brown | Sex | GO:0033559 | unsaturated fatty acid metabolic process | 4/34 | 87/15600 | 2.28E-03 | *Cyp2c38, Cyp2c39, Cyp2a22, Cyp2g1* | drug process metabolic acid | 1.70E-03 |
| Liver | Brown | Sex | GO:0006690 | icosanoid metabolic process | 4/34 | 92/15600 | 2.61E-03 | *Cyp2c38, Cyp2c39, Cyp2a22, Cyp2g1* | drug process metabolic acid | 1.70E-03 |
| Liver | Brown | Sex | GO:0009410 | response to xenobiotic stimulus | 5/34 | 198/15600 | 3.39E-03 | *Cyp2c38, Cyp2c39, Sult1a1, Cyp2a22, Cyp2g1* | drug process metabolic acid | 1.70E-03 |
| Liver | Brown | Sex | GO:0006631 | fatty acid metabolic process | 6/34 | 338/15600 | 3.88E-03 | *Cyp2c38, Cyp2c39, Hao2, Cyp2a22, Eci3, Cyp2g1* | drug process metabolic acid | 1.70E-03 |
| Liver | Brown | Sex | GO:1901568 | fatty acid derivative metabolic process | 4/34 | 112/15600 | 4.50E-03 | *Cyp2c38, Cyp2c39, Cyp2a22, Cyp2g1* | drug process metabolic acid | 1.70E-03 |
| Liver | Brown | Sex | GO:0060736 | prostate gland growth | 2/34 | 11/15600 | 1.07E-02 | *Prlr, Esr1* | gland lobule development mammary | 2.38E-02 |
| Liver | Brown | Sex | GO:0061180 | mammary gland epithelium development | 3/34 | 64/15600 | 1.44E-02 | *Prlr, Rtn4, Esr1* | gland lobule development mammary | 2.38E-02 |
| Liver | Brown | Sex | GO:0060749 | mammary gland alveolus development | 2/34 | 19/15600 | 2.77E-02 | *Prlr, Esr1* | gland lobule development mammary | 2.38E-02 |
| Liver | Brown | Sex | GO:0061377 | mammary gland lobule development | 2/34 | 19/15600 | 2.77E-02 | *Prlr, Esr1* | gland lobule development mammary | 2.38E-02 |
| Liver | Brown | Sex | GO:1901186 | positive regulation of ERBB signaling pathway | 2/34 | 23/15600 | 3.87E-02 | *Rtn4, Esr1* | gland lobule development mammary | 2.38E-02 |
| Liver | Blue | Sex | GO:0008610 | lipid biosynthetic process | 11/78 | 482/15600 | 3.43E-02 | *Scp2, Elovl3, Hsd17b12, Serinc1, Cdipt, Smpd1, Mecr, Pisd, Elovl2, Rest, Pip5k1a* | lipid biosynthetic process | 3.43E-02 |
| Muscle | Magenta | Age | NA | NA | NA | NA | NA | *NA* | NA | NA |
| Muscle | Brown | Age | GO:0030198 | extracellular matrix organization | 7/30 | 232/15414 | 9.31E-05 | *Col3a1, Col5a2, Col5a1, Fn1, Nid1, Tgfbi, Anxa2* | regulation morphogenesis negative development | 1.12E-02 |
| Muscle | Brown | Age | GO:0043062 | extracellular structure organization | 7/30 | 233/15414 | 9.31E-05 | *Col3a1, Col5a2, Col5a1, Fn1, Nid1, Tgfbi, Anxa2* | regulation morphogenesis negative development | 1.12E-02 |
| Muscle | Brown | Age | GO:0003417 | growth plate cartilage development | 4/30 | 31/15414 | 9.31E-05 | *Col6a2, Col6a1, Col6a3, Anxa2* | regulation morphogenesis negative development | 1.12E-02 |
| Muscle | Brown | Age | GO:0003416 | endochondral bone growth | 4/30 | 37/15414 | 1.45E-04 | *Col6a2, Col6a1, Col6a3, Anxa2* | regulation morphogenesis negative development | 1.12E-02 |
| Muscle | Brown | Age | GO:0098868 | bone growth | 4/30 | 40/15414 | 1.60E-04 | *Col6a2, Col6a1, Col6a3, Anxa2* | regulation morphogenesis negative development | 1.12E-02 |
| Muscle | Brown | Age | GO:0060351 | cartilage development involved in endochondral bone morphogenesis | 4/30 | 45/15414 | 1.65E-04 | *Col6a2, Col6a1, Col6a3, Anxa2* | regulation morphogenesis negative development | 1.12E-02 |
| Muscle | Brown | Age | GO:0003414 | chondrocyte morphogenesis involved in endochondral bone morphogenesis | 3/30 | 13/15414 | 1.65E-04 | *Col6a2, Col6a1, Col6a3* | regulation morphogenesis negative development | 1.12E-02 |
| Muscle | Brown | Age | GO:0003429 | growth plate cartilage chondrocyte morphogenesis | 3/30 | 13/15414 | 1.65E-04 | *Col6a2, Col6a1, Col6a3* | regulation morphogenesis negative development | 1.12E-02 |
| Muscle | Brown | Age | GO:0090171 | chondrocyte morphogenesis | 3/30 | 13/15414 | 1.65E-04 | *Col6a2, Col6a1, Col6a3* | regulation morphogenesis negative development | 1.12E-02 |
| Muscle | Brown | Age | GO:0003422 | growth plate cartilage morphogenesis | 3/30 | 14/15414 | 1.89E-04 | *Col6a2, Col6a1, Col6a3* | regulation morphogenesis negative development | 1.12E-02 |
| Muscle | Brown | Age | GO:0030199 | collagen fibril organization | 4/30 | 52/15414 | 2.12E-04 | *Col3a1, Col5a2, Col5a1, Anxa2* | regulation morphogenesis negative development | 1.12E-02 |
| Muscle | Brown | Age | GO:0003418 | growth plate cartilage chondrocyte differentiation | 3/30 | 18/15414 | 3.50E-04 | *Col6a2, Col6a1, Col6a3* | regulation morphogenesis negative development | 1.12E-02 |
| Muscle | Brown | Age | GO:0003433 | chondrocyte development involved in endochondral bone morphogenesis | 3/30 | 19/15414 | 3.56E-04 | *Col6a2, Col6a1, Col6a3* | regulation morphogenesis negative development | 1.12E-02 |
| Muscle | Brown | Age | GO:0035987 | endodermal cell differentiation | 3/30 | 19/15414 | 3.56E-04 | *Col5a2, Col5a1, Fn1* | regulation morphogenesis negative development | 1.12E-02 |
| Muscle | Brown | Age | GO:0060350 | endochondral bone morphogenesis | 4/30 | 66/15414 | 4.07E-04 | *Col6a2, Col6a1, Col6a3, Anxa2* | regulation morphogenesis negative development | 1.12E-02 |
| Muscle | Brown | Age | GO:0060536 | cartilage morphogenesis | 3/30 | 22/15414 | 4.93E-04 | *Col6a2, Col6a1, Col6a3* | regulation morphogenesis negative development | 1.12E-02 |
| Muscle | Brown | Age | GO:0009611 | response to wounding | 7/30 | 412/15414 | 5.04E-04 | *Col3a1, Col5a1, Fn1, Cd34, Serping1, Anxa2, Lrp1* | regulation morphogenesis negative development | 1.12E-02 |
| Muscle | Brown | Age | GO:0001706 | endoderm formation | 3/30 | 23/15414 | 5.04E-04 | *Col5a2, Col5a1, Fn1* | regulation morphogenesis negative development | 1.12E-02 |
| Muscle | Brown | Age | GO:0003413 | chondrocyte differentiation involved in endochondral bone morphogenesis | 3/30 | 24/15414 | 5.45E-04 | *Col6a2, Col6a1, Col6a3* | regulation morphogenesis negative development | 1.12E-02 |
| Muscle | Brown | Age | GO:0042060 | wound healing | 6/30 | 304/15414 | 8.81E-04 | *Col3a1, Col5a1, Fn1, Cd34, Serping1, Anxa2* | regulation morphogenesis negative development | 1.12E-02 |
| Muscle | Brown | Age | GO:0035265 | organ growth | 5/30 | 186/15414 | 1.02E-03 | *Pi16, Col6a2, Col6a1, Col6a3, Anxa2* | regulation morphogenesis negative development | 1.12E-02 |
| Muscle | Brown | Age | GO:0007160 | cell-matrix adhesion | 5/30 | 191/15414 | 1.10E-03 | *Col3a1, Fn1, Itgbl1, Nid1, Lrp1* | regulation morphogenesis negative development | 1.12E-02 |
| Muscle | Brown | Age | GO:0060348 | bone development | 5/30 | 197/15414 | 1.17E-03 | *Sparc, Col6a2, Col6a1, Col6a3, Anxa2* | regulation morphogenesis negative development | 1.12E-02 |
| Muscle | Brown | Age | GO:0060349 | bone morphogenesis | 4/30 | 97/15414 | 1.17E-03 | *Col6a2, Col6a1, Col6a3, Anxa2* | regulation morphogenesis negative development | 1.12E-02 |
| Muscle | Brown | Age | GO:0002062 | chondrocyte differentiation | 4/30 | 102/15414 | 1.37E-03 | *Col6a2, Col6a1, Col6a3, Anxa2* | regulation morphogenesis negative development | 1.12E-02 |
| Muscle | Brown | Age | GO:0002063 | chondrocyte development | 3/30 | 40/15414 | 1.83E-03 | *Col6a2, Col6a1, Col6a3* | regulation morphogenesis negative development | 1.12E-02 |
| Muscle | Brown | Age | GO:0030195 | negative regulation of blood coagulation | 3/30 | 40/15414 | 1.83E-03 | *Cd34, Serping1, Anxa2* | regulation morphogenesis negative development | 1.12E-02 |
| Muscle | Brown | Age | GO:1900047 | negative regulation of hemostasis | 3/30 | 41/15414 | 1.90E-03 | *Cd34, Serping1, Anxa2* | regulation morphogenesis negative development | 1.12E-02 |
| Muscle | Brown | Age | GO:0050819 | negative regulation of coagulation | 3/30 | 42/15414 | 1.98E-03 | *Cd34, Serping1, Anxa2* | regulation morphogenesis negative development | 1.12E-02 |
| Muscle | Brown | Age | GO:0061448 | connective tissue development | 5/30 | 238/15414 | 2.30E-03 | *Col5a1, Col6a2, Col6a1, Col6a3, Anxa2* | regulation morphogenesis negative development | 1.12E-02 |
| Muscle | Brown | Age | GO:0007492 | endoderm development | 3/30 | 49/15414 | 2.94E-03 | *Col5a2, Col5a1, Fn1* | regulation morphogenesis negative development | 1.12E-02 |
| Muscle | Brown | Age | GO:0008347 | glial cell migration | 3/30 | 53/15414 | 3.60E-03 | *Fn1, Ndn, Lrp1* | regulation morphogenesis negative development | 1.12E-02 |
| Muscle | Brown | Age | GO:0001501 | skeletal system development | 6/30 | 444/15414 | 4.36E-03 | *Col5a2, Sparc, Col6a2, Col6a1, Col6a3, Anxa2* | regulation morphogenesis negative development | 1.12E-02 |
| Muscle | Brown | Age | GO:0050710 | negative regulation of cytokine secretion | 3/30 | 58/15414 | 4.44E-03 | *Fn1, Ssc5d, Cd34* | regulation morphogenesis negative development | 1.12E-02 |
| Muscle | Brown | Age | GO:0071229 | cellular response to acid chemical | 4/30 | 151/15414 | 4.49E-03 | *Col3a1, Col5a2, Col6a1, Anxa2* | regulation morphogenesis negative development | 1.12E-02 |
| Muscle | Brown | Age | GO:0061045 | negative regulation of wound healing | 3/30 | 60/15414 | 4.64E-03 | *Cd34, Serping1, Anxa2* | regulation morphogenesis negative development | 1.12E-02 |
| Muscle | Brown | Age | GO:1903034 | regulation of response to wounding | 4/30 | 155/15414 | 4.69E-03 | *Cd34, Serping1, Anxa2, Lrp1* | regulation morphogenesis negative development | 1.12E-02 |
| Muscle | Brown | Age | GO:0071230 | cellular response to amino acid stimulus | 3/30 | 65/15414 | 5.57E-03 | *Col3a1, Col5a2, Col6a1* | regulation morphogenesis negative development | 1.12E-02 |
| Muscle | Brown | Age | GO:0030193 | regulation of blood coagulation | 3/30 | 66/15414 | 5.68E-03 | *Cd34, Serping1, Anxa2* | regulation morphogenesis negative development | 1.12E-02 |
| Muscle | Brown | Age | GO:1900046 | regulation of hemostasis | 3/30 | 67/15414 | 5.79E-03 | *Cd34, Serping1, Anxa2* | regulation morphogenesis negative development | 1.12E-02 |
| Muscle | Brown | Age | GO:0031589 | cell-substrate adhesion | 5/30 | 316/15414 | 6.00E-03 | *Col3a1, Fn1, Itgbl1, Nid1, Lrp1* | regulation morphogenesis negative development | 1.12E-02 |
| Muscle | Brown | Age | GO:0001704 | formation of primary germ layer | 3/30 | 70/15414 | 6.00E-03 | *Col5a2, Col5a1, Fn1* | regulation morphogenesis negative development | 1.12E-02 |
| Muscle | Brown | Age | GO:0050818 | regulation of coagulation | 3/30 | 70/15414 | 6.00E-03 | *Cd34, Serping1, Anxa2* | regulation morphogenesis negative development | 1.12E-02 |
| Muscle | Brown | Age | GO:0010466 | negative regulation of peptidase activity | 4/30 | 173/15414 | 6.00E-03 | *Serpinf1, Pi16, Serping1, Timp2* | regulation morphogenesis negative development | 1.12E-02 |
| Muscle | Brown | Age | GO:0050707 | regulation of cytokine secretion | 4/30 | 174/15414 | 6.00E-03 | *Fn1, Ssc5d, Cd34, Lrp1* | regulation morphogenesis negative development | 1.12E-02 |
| Muscle | Brown | Age | GO:0051216 | cartilage development | 4/30 | 176/15414 | 6.13E-03 | *Col6a2, Col6a1, Col6a3, Anxa2* | regulation morphogenesis negative development | 1.12E-02 |
| Muscle | Brown | Age | GO:0042730 | fibrinolysis | 2/30 | 15/15414 | 6.36E-03 | *Serping1, Anxa2* | regulation morphogenesis negative development | 1.12E-02 |
| Muscle | Brown | Age | GO:0043200 | response to amino acid | 3/30 | 76/15414 | 6.85E-03 | *Col3a1, Col5a2, Col6a1* | regulation morphogenesis negative development | 1.12E-02 |
| Muscle | Brown | Age | GO:1903035 | negative regulation of response to wounding | 3/30 | 76/15414 | 6.85E-03 | *Cd34, Serping1, Anxa2* | regulation morphogenesis negative development | 1.12E-02 |
| Muscle | Brown | Age | GO:0070208 | protein heterotrimerization | 2/30 | 18/15414 | 8.68E-03 | *Col6a2, Col6a1* | regulation morphogenesis negative development | 1.12E-02 |
| Muscle | Brown | Age | GO:0050663 | cytokine secretion | 4/30 | 202/15414 | 9.31E-03 | *Fn1, Ssc5d, Cd34, Lrp1* | regulation morphogenesis negative development | 1.12E-02 |
| Muscle | Brown | Age | GO:0001101 | response to acid chemical | 4/30 | 216/15414 | 1.17E-02 | *Col3a1, Col5a2, Col6a1, Anxa2* | regulation morphogenesis negative development | 1.12E-02 |
| Muscle | Brown | Age | GO:0048705 | skeletal system morphogenesis | 4/30 | 217/15414 | 1.17E-02 | *Col6a2, Col6a1, Col6a3, Anxa2* | regulation morphogenesis negative development | 1.12E-02 |
| Muscle | Brown | Age | GO:0048588 | developmental cell growth | 4/30 | 228/15414 | 1.38E-02 | *Fn1, Pi16, Ndn, Lrp1* | regulation morphogenesis negative development | 1.12E-02 |
| Muscle | Brown | Age | GO:0018149 | peptide cross-linking | 2/30 | 24/15414 | 1.39E-02 | *Col3a1, Fn1* | regulation morphogenesis negative development | 1.12E-02 |
| Muscle | Brown | Age | GO:0045992 | negative regulation of embryonic development | 2/30 | 24/15414 | 1.39E-02 | *Col5a2, Col5a1* | regulation morphogenesis negative development | 1.12E-02 |
| Muscle | Brown | Age | GO:0032373 | positive regulation of sterol transport | 2/30 | 25/15414 | 1.46E-02 | *Anxa2, Lrp1* | regulation morphogenesis negative development | 1.12E-02 |
| Muscle | Brown | Age | GO:0032376 | positive regulation of cholesterol transport | 2/30 | 25/15414 | 1.46E-02 | *Anxa2, Lrp1* | regulation morphogenesis negative development | 1.12E-02 |
| Muscle | Brown | Age | GO:0010470 | regulation of gastrulation | 2/30 | 30/15414 | 2.06E-02 | *Col5a2, Col5a1* | regulation morphogenesis negative development | 1.12E-02 |
| Muscle | Brown | Age | GO:0045861 | negative regulation of proteolysis | 4/30 | 264/15414 | 2.11E-02 | *Serpinf1, Pi16, Serping1, Timp2* | regulation morphogenesis negative development | 1.12E-02 |
| Muscle | Brown | Age | GO:0001525 | angiogenesis | 5/30 | 451/15414 | 2.11E-02 | *Fn1, Serpinf1, Sparc, Cd34, Anxa2* | regulation morphogenesis negative development | 1.12E-02 |
| Muscle | Brown | Age | GO:0061041 | regulation of wound healing | 3/30 | 122/15414 | 2.14E-02 | *Cd34, Serping1, Anxa2* | regulation morphogenesis negative development | 1.12E-02 |
| Muscle | Brown | Age | GO:0051291 | protein heterooligomerization | 3/30 | 123/15414 | 2.14E-02 | *Col6a2, Col6a1, Anxa2* | regulation morphogenesis negative development | 1.12E-02 |
| Muscle | Brown | Age | GO:0035909 | aorta morphogenesis | 2/30 | 32/15414 | 2.14E-02 | *Col3a1, Lrp1* | regulation morphogenesis negative development | 1.12E-02 |
| Muscle | Brown | Age | GO:0048675 | axon extension | 3/30 | 124/15414 | 2.14E-02 | *Fn1, Ndn, Lrp1* | regulation morphogenesis negative development | 1.12E-02 |
| Muscle | Brown | Age | GO:0050709 | negative regulation of protein secretion | 3/30 | 128/15414 | 2.31E-02 | *Fn1, Ssc5d, Cd34* | regulation morphogenesis negative development | 1.12E-02 |
| Muscle | Brown | Age | GO:0007596 | blood coagulation | 3/30 | 132/15414 | 2.45E-02 | *Cd34, Serping1, Anxa2* | regulation morphogenesis negative development | 1.12E-02 |
| Muscle | Brown | Age | GO:0010951 | negative regulation of endopeptidase activity | 3/30 | 132/15414 | 2.45E-02 | *Serpinf1, Serping1, Timp2* | regulation morphogenesis negative development | 1.12E-02 |
| Muscle | Brown | Age | GO:0007369 | gastrulation | 3/30 | 133/15414 | 2.46E-02 | *Col5a2, Col5a1, Fn1* | regulation morphogenesis negative development | 1.12E-02 |
| Muscle | Brown | Age | GO:0007599 | hemostasis | 3/30 | 134/15414 | 2.46E-02 | *Cd34, Serping1, Anxa2* | regulation morphogenesis negative development | 1.12E-02 |
| Muscle | Brown | Age | GO:0002792 | negative regulation of peptide secretion | 3/30 | 135/15414 | 2.46E-02 | *Fn1, Ssc5d, Cd34* | regulation morphogenesis negative development | 1.12E-02 |
| Muscle | Brown | Age | GO:0050817 | coagulation | 3/30 | 135/15414 | 2.46E-02 | *Cd34, Serping1, Anxa2* | regulation morphogenesis negative development | 1.12E-02 |
| Muscle | Brown | Age | GO:0007169 | transmembrane receptor protein tyrosine kinase signaling pathway | 5/30 | 489/15414 | 2.52E-02 | *Angptl1, Dok2, Igfbp6, Ndn, Lrp1* | regulation morphogenesis negative development | 1.12E-02 |
| Muscle | Brown | Age | GO:0032371 | regulation of sterol transport | 2/30 | 39/15414 | 2.73E-02 | *Anxa2, Lrp1* | regulation morphogenesis negative development | 1.12E-02 |
| Muscle | Brown | Age | GO:0032374 | regulation of cholesterol transport | 2/30 | 39/15414 | 2.73E-02 | *Anxa2, Lrp1* | regulation morphogenesis negative development | 1.12E-02 |
| Muscle | Brown | Age | GO:0051346 | negative regulation of hydrolase activity | 4/30 | 310/15414 | 3.03E-02 | *Serpinf1, Pi16, Serping1, Timp2* | regulation morphogenesis negative development | 1.12E-02 |
| Muscle | Brown | Age | GO:0032102 | negative regulation of response to external stimulus | 4/30 | 315/15414 | 3.16E-02 | *Serpinf1, Cd34, Serping1, Anxa2* | regulation morphogenesis negative development | 1.12E-02 |
| Muscle | Brown | Age | GO:0052547 | regulation of peptidase activity | 4/30 | 321/15414 | 3.34E-02 | *Serpinf1, Pi16, Serping1, Timp2* | regulation morphogenesis negative development | 1.12E-02 |
| Muscle | Brown | Age | GO:0045773 | positive regulation of axon extension | 2/30 | 51/15414 | 4.40E-02 | *Fn1, Lrp1* | regulation morphogenesis negative development | 1.12E-02 |
| Muscle | Brown | Age | GO:1990138 | neuron projection extension | 3/30 | 173/15414 | 4.46E-02 | *Fn1, Ndn, Lrp1* | regulation morphogenesis negative development | 1.12E-02 |
| Muscle | Brown | Age | GO:0051224 | negative regulation of protein transport | 3/30 | 176/15414 | 4.62E-02 | *Fn1, Ssc5d, Cd34* | regulation morphogenesis negative development | 1.12E-02 |
| Muscle | Brown | Age | GO:1904950 | negative regulation of establishment of protein localization | 3/30 | 181/15414 | 4.93E-02 | *Fn1, Ssc5d, Cd34* | regulation morphogenesis negative development | 1.12E-02 |
| Muscle | Red | Sex | GO:0006596 | polyamine biosynthetic process | 2/20 | 11/15414 | 1.14E-02 | *Amd1, Amd2* | metabolic process amine biosynthetic | 1.86E-02 |
| Muscle | Red | Sex | GO:0009309 | amine biosynthetic process | 2/20 | 13/15414 | 1.14E-02 | *Amd1, Amd2* | metabolic process amine biosynthetic | 1.86E-02 |
| Muscle | Red | Sex | GO:0042401 | cellular biogenic amine biosynthetic process | 2/20 | 13/15414 | 1.14E-02 | *Amd1, Amd2* | metabolic process amine biosynthetic | 1.86E-02 |
| Muscle | Red | Sex | GO:0006595 | polyamine metabolic process | 2/20 | 16/15414 | 1.31E-02 | *Amd1, Amd2* | metabolic process amine biosynthetic | 1.86E-02 |
| Muscle | Red | Sex | GO:0006576 | cellular biogenic amine metabolic process | 2/20 | 33/15414 | 4.57E-02 | *Amd1, Amd2* | metabolic process amine biosynthetic | 1.86E-02 |
| Muscle | Purple | Sex | GO:0006790 | sulfur compound metabolic process | 4/9 | 236/15414 | 6.40E-04 | *Gstm2, Ces1d, Suclg2, Gcdh* | process metabolic catabolic acid | 7.26E-03 |
| Muscle | Purple | Sex | GO:0006637 | acyl-CoA metabolic process | 3/9 | 73/15414 | 6.40E-04 | *Ces1d, Suclg2, Gcdh* | process metabolic catabolic acid | 7.26E-03 |
| Muscle | Purple | Sex | GO:0035383 | thioester metabolic process | 3/9 | 73/15414 | 6.40E-04 | *Ces1d, Suclg2, Gcdh* | process metabolic catabolic acid | 7.26E-03 |
| Muscle | Purple | Sex | GO:0009062 | fatty acid catabolic process | 3/9 | 87/15414 | 6.40E-04 | *Ces1d, Acox1, Gcdh* | process metabolic catabolic acid | 7.26E-03 |
| Muscle | Purple | Sex | GO:0033865 | nucleoside bisphosphate metabolic process | 3/9 | 88/15414 | 6.40E-04 | *Ces1d, Suclg2, Gcdh* | process metabolic catabolic acid | 7.26E-03 |
| Muscle | Purple | Sex | GO:0033875 | ribonucleoside bisphosphate metabolic process | 3/9 | 88/15414 | 6.40E-04 | *Ces1d, Suclg2, Gcdh* | process metabolic catabolic acid | 7.26E-03 |
| Muscle | Purple | Sex | GO:0034032 | purine nucleoside bisphosphate metabolic process | 3/9 | 88/15414 | 6.40E-04 | *Ces1d, Suclg2, Gcdh* | process metabolic catabolic acid | 7.26E-03 |
| Muscle | Purple | Sex | GO:0072329 | monocarboxylic acid catabolic process | 3/9 | 102/15414 | 8.16E-04 | *Ces1d, Acox1, Gcdh* | process metabolic catabolic acid | 7.26E-03 |
| Muscle | Purple | Sex | GO:0051186 | cofactor metabolic process | 4/9 | 331/15414 | 8.16E-04 | *Gstm2, Ces1d, Suclg2, Gcdh* | process metabolic catabolic acid | 7.26E-03 |
| Muscle | Purple | Sex | GO:0044242 | cellular lipid catabolic process | 3/9 | 178/15414 | 3.17E-03 | *Ces1d, Acox1, Gcdh* | process metabolic catabolic acid | 7.26E-03 |
| Muscle | Purple | Sex | GO:0006732 | coenzyme metabolic process | 3/9 | 184/15414 | 3.17E-03 | *Ces1d, Suclg2, Gcdh* | process metabolic catabolic acid | 7.26E-03 |
| Muscle | Purple | Sex | GO:0016054 | organic acid catabolic process | 3/9 | 185/15414 | 3.17E-03 | *Ces1d, Acox1, Gcdh* | process metabolic catabolic acid | 7.26E-03 |
| Muscle | Purple | Sex | GO:0046395 | carboxylic acid catabolic process | 3/9 | 185/15414 | 3.17E-03 | *Ces1d, Acox1, Gcdh* | process metabolic catabolic acid | 7.26E-03 |
| Muscle | Purple | Sex | GO:0016042 | lipid catabolic process | 3/9 | 260/15414 | 8.03E-03 | *Ces1d, Acox1, Gcdh* | process metabolic catabolic acid | 7.26E-03 |
| Muscle | Purple | Sex | GO:0044282 | small molecule catabolic process | 3/9 | 275/15414 | 8.64E-03 | *Ces1d, Acox1, Gcdh* | process metabolic catabolic acid | 7.26E-03 |
| Muscle | Purple | Sex | GO:0009150 | purine ribonucleotide metabolic process | 3/9 | 279/15414 | 8.64E-03 | *Ces1d, Suclg2, Gcdh* | process metabolic catabolic acid | 7.26E-03 |
| Muscle | Purple | Sex | GO:0009259 | ribonucleotide metabolic process | 3/9 | 289/15414 | 8.91E-03 | *Ces1d, Suclg2, Gcdh* | process metabolic catabolic acid | 7.26E-03 |
| Muscle | Purple | Sex | GO:0006163 | purine nucleotide metabolic process | 3/9 | 298/15414 | 8.91E-03 | *Ces1d, Suclg2, Gcdh* | process metabolic catabolic acid | 7.26E-03 |
| Muscle | Purple | Sex | GO:0019693 | ribose phosphate metabolic process | 3/9 | 299/15414 | 8.91E-03 | *Ces1d, Suclg2, Gcdh* | process metabolic catabolic acid | 7.26E-03 |
| Muscle | Purple | Sex | GO:0006635 | fatty acid beta-oxidation | 2/9 | 65/15414 | 9.40E-03 | *Acox1, Gcdh* | process metabolic catabolic acid | 7.26E-03 |
| Muscle | Purple | Sex | GO:0006631 | fatty acid metabolic process | 3/9 | 321/15414 | 9.91E-03 | *Ces1d, Acox1, Gcdh* | process metabolic catabolic acid | 7.26E-03 |
| Muscle | Purple | Sex | GO:0072521 | purine-containing compound metabolic process | 3/9 | 334/15414 | 1.06E-02 | *Ces1d, Suclg2, Gcdh* | process metabolic catabolic acid | 7.26E-03 |
| Muscle | Purple | Sex | GO:0006091 | generation of precursor metabolites and energy | 3/9 | 347/15414 | 1.14E-02 | *Ppp1r1a, Acox1, Suclg2* | generation precursor metabolites energy | 1.39E-02 |
| Muscle | Purple | Sex | GO:0009117 | nucleotide metabolic process | 3/9 | 367/15414 | 1.28E-02 | *Ces1d, Suclg2, Gcdh* | process metabolic catabolic acid | 7.26E-03 |
| Muscle | Purple | Sex | GO:0006753 | nucleoside phosphate metabolic process | 3/9 | 374/15414 | 1.30E-02 | *Ces1d, Suclg2, Gcdh* | process metabolic catabolic acid | 7.26E-03 |
| Muscle | Purple | Sex | GO:0019395 | fatty acid oxidation | 2/9 | 92/15414 | 1.44E-02 | *Acox1, Gcdh* | process metabolic catabolic acid | 7.26E-03 |
| Muscle | Purple | Sex | GO:0034440 | lipid oxidation | 2/9 | 94/15414 | 1.45E-02 | *Acox1, Gcdh* | process metabolic catabolic acid | 7.26E-03 |
| Muscle | Purple | Sex | GO:0005976 | polysaccharide metabolic process | 2/9 | 102/15414 | 1.64E-02 | *Ppp1r1a, Egf* | generation precursor metabolites energy | 1.39E-02 |
| Muscle | Purple | Sex | GO:0055086 | nucleobase-containing small molecule metabolic process | 3/9 | 430/15414 | 1.66E-02 | *Ces1d, Suclg2, Gcdh* | process metabolic catabolic acid | 7.26E-03 |
| Muscle | Purple | Sex | GO:1901568 | fatty acid derivative metabolic process | 2/9 | 106/15414 | 1.66E-02 | *Acox1, Gcdh* | process metabolic catabolic acid | 7.26E-03 |
| Muscle | Purple | Sex | GO:0032787 | monocarboxylic acid metabolic process | 3/9 | 483/15414 | 2.19E-02 | *Ces1d, Acox1, Gcdh* | process metabolic catabolic acid | 7.26E-03 |
| Muscle | Greenyellow | Sex | GO:0097202 | activation of cysteine-type endopeptidase activity | 1/1 | 12/15414 | 1.32E-02 | *Cyfip2* | positive regulation endopeptidase activity | 2.47E-02 |
| Muscle | Greenyellow | Sex | GO:0051386 | regulation of neurotrophin TRK receptor signaling pathway | 1/1 | 14/15414 | 1.32E-02 | *Cyfip2* | positive regulation endopeptidase activity | 2.47E-02 |
| Muscle | Greenyellow | Sex | GO:0048011 | neurotrophin TRK receptor signaling pathway | 1/1 | 27/15414 | 1.35E-02 | *Cyfip2* | positive regulation endopeptidase activity | 2.47E-02 |
| Muscle | Greenyellow | Sex | GO:0038179 | neurotrophin signaling pathway | 1/1 | 36/15414 | 1.35E-02 | *Cyfip2* | positive regulation endopeptidase activity | 2.47E-02 |
| Muscle | Greenyellow | Sex | GO:0097484 | dendrite extension | 1/1 | 36/15414 | 1.35E-02 | *Cyfip2* | positive regulation endopeptidase activity | 2.47E-02 |
| Muscle | Greenyellow | Sex | GO:0031638 | zymogen activation | 1/1 | 48/15414 | 1.51E-02 | *Cyfip2* | positive regulation endopeptidase activity | 2.47E-02 |
| Muscle | Greenyellow | Sex | GO:2001056 | positive regulation of cysteine-type endopeptidase activity | 1/1 | 117/15414 | 2.51E-02 | *Cyfip2* | positive regulation endopeptidase activity | 2.47E-02 |
| Muscle | Greenyellow | Sex | GO:0010950 | positive regulation of endopeptidase activity | 1/1 | 133/15414 | 2.51E-02 | *Cyfip2* | positive regulation endopeptidase activity | 2.47E-02 |
| Muscle | Greenyellow | Sex | GO:0010952 | positive regulation of peptidase activity | 1/1 | 142/15414 | 2.51E-02 | *Cyfip2* | positive regulation endopeptidase activity | 2.47E-02 |
| Muscle | Greenyellow | Sex | GO:1990138 | neuron projection extension | 1/1 | 173/15414 | 2.51E-02 | *Cyfip2* | positive regulation endopeptidase activity | 2.47E-02 |
| Muscle | Greenyellow | Sex | GO:0007411 | axon guidance | 1/1 | 190/15414 | 2.51E-02 | *Cyfip2* | positive regulation endopeptidase activity | 2.47E-02 |
| Muscle | Greenyellow | Sex | GO:0097485 | neuron projection guidance | 1/1 | 190/15414 | 2.51E-02 | *Cyfip2* | positive regulation endopeptidase activity | 2.47E-02 |
| Muscle | Greenyellow | Sex | GO:0016485 | protein processing | 1/1 | 192/15414 | 2.51E-02 | *Cyfip2* | positive regulation endopeptidase activity | 2.47E-02 |
| Muscle | Greenyellow | Sex | GO:2000116 | regulation of cysteine-type endopeptidase activity | 1/1 | 197/15414 | 2.51E-02 | *Cyfip2* | positive regulation endopeptidase activity | 2.47E-02 |
| Muscle | Greenyellow | Sex | GO:0048588 | developmental cell growth | 1/1 | 228/15414 | 2.51E-02 | *Cyfip2* | positive regulation endopeptidase activity | 2.47E-02 |
| Muscle | Greenyellow | Sex | GO:0060560 | developmental growth involved in morphogenesis | 1/1 | 235/15414 | 2.51E-02 | *Cyfip2* | positive regulation endopeptidase activity | 2.47E-02 |
| Muscle | Greenyellow | Sex | GO:0090287 | regulation of cellular response to growth factor stimulus | 1/1 | 237/15414 | 2.51E-02 | *Cyfip2* | positive regulation endopeptidase activity | 2.47E-02 |
| Muscle | Greenyellow | Sex | GO:0051604 | protein maturation | 1/1 | 240/15414 | 2.51E-02 | *Cyfip2* | positive regulation endopeptidase activity | 2.47E-02 |
| Muscle | Greenyellow | Sex | GO:0052548 | regulation of endopeptidase activity | 1/1 | 270/15414 | 2.67E-02 | *Cyfip2* | positive regulation endopeptidase activity | 2.47E-02 |
| Muscle | Greenyellow | Sex | GO:0045862 | positive regulation of proteolysis | 1/1 | 296/15414 | 2.78E-02 | *Cyfip2* | positive regulation endopeptidase activity | 2.47E-02 |
| Muscle | Greenyellow | Sex | GO:0052547 | regulation of peptidase activity | 1/1 | 321/15414 | 2.88E-02 | *Cyfip2* | positive regulation endopeptidase activity | 2.47E-02 |
| Muscle | Greenyellow | Sex | GO:0007409 | axonogenesis | 1/1 | 389/15414 | 3.12E-02 | *Cyfip2* | positive regulation endopeptidase activity | 2.47E-02 |
| Muscle | Greenyellow | Sex | GO:0061564 | axon development | 1/1 | 424/15414 | 3.12E-02 | *Cyfip2* | positive regulation endopeptidase activity | 2.47E-02 |
| Muscle | Greenyellow | Sex | GO:0030031 | cell projection assembly | 1/1 | 437/15414 | 3.12E-02 | *Cyfip2* | positive regulation endopeptidase activity | 2.47E-02 |
| Muscle | Greenyellow | Sex | GO:0016049 | cell growth | 1/1 | 442/15414 | 3.12E-02 | *Cyfip2* | positive regulation endopeptidase activity | 2.47E-02 |
| Muscle | Greenyellow | Sex | GO:0051345 | positive regulation of hydrolase activity | 1/1 | 451/15414 | 3.12E-02 | *Cyfip2* | positive regulation endopeptidase activity | 2.47E-02 |
| Muscle | Greenyellow | Sex | GO:0006935 | chemotaxis | 1/1 | 462/15414 | 3.12E-02 | *Cyfip2* | positive regulation endopeptidase activity | 2.47E-02 |
| Muscle | Greenyellow | Sex | GO:0042330 | taxis | 1/1 | 465/15414 | 3.12E-02 | *Cyfip2* | positive regulation endopeptidase activity | 2.47E-02 |
| Muscle | Greenyellow | Sex | GO:0007169 | transmembrane receptor protein tyrosine kinase signaling pathway | 1/1 | 489/15414 | 3.17E-02 | *Cyfip2* | positive regulation endopeptidase activity | 2.47E-02 |
| Muscle | Blue | Sex | GO:0046034 | ATP metabolic process | 4/21 | 216/15414 | 1.57E-02 | *Atp5l, Atp5g3, Cox5a, Ndufa8* | ribonucleoside triphosphate metabolic process | 2.89E-02 |
| Muscle | Blue | Sex | GO:0015985 | energy coupled proton transport, down electrochemical gradient | 2/21 | 18/15414 | 1.57E-02 | *Atp5l, Atp5g3* | ribonucleoside triphosphate metabolic process | 2.89E-02 |
| Muscle | Blue | Sex | GO:0015986 | ATP synthesis coupled proton transport | 2/21 | 18/15414 | 1.57E-02 | *Atp5l, Atp5g3* | ribonucleoside triphosphate metabolic process | 2.89E-02 |
| Muscle | Blue | Sex | GO:0010499 | proteasomal ubiquitin-independent protein catabolic process | 2/21 | 22/15414 | 1.78E-02 | *Psmb1, Psmb3* | proteasomal ubiquitin-independent protein catabolic | 1.78E-02 |
| Muscle | Blue | Sex | GO:0000462 | maturation of SSU-rRNA from tricistronic rRNA transcript (SSU-rRNA, 5.8S rRNA, LSU-rRNA) | 2/21 | 30/15414 | 2.66E-02 | *Rps14, Rps16* | transcript ssu-rrna tricistronic rrna | 3.05E-02 |
| Muscle | Blue | Sex | GO:0030490 | maturation of SSU-rRNA | 2/21 | 41/15414 | 2.86E-02 | *Rps14, Rps16* | transcript ssu-rrna tricistronic rrna | 3.05E-02 |
| Muscle | Blue | Sex | GO:0006754 | ATP biosynthetic process | 2/21 | 44/15414 | 2.86E-02 | *Atp5l, Atp5g3* | ribonucleoside triphosphate metabolic process | 2.89E-02 |
| Muscle | Blue | Sex | GO:0010257 | NADH dehydrogenase complex assembly | 2/21 | 44/15414 | 2.86E-02 | *Ndufa8, Ndufb2* | mitochondrial electron transport chain | 3.24E-02 |
| Muscle | Blue | Sex | GO:0032981 | mitochondrial respiratory chain complex I assembly | 2/21 | 44/15414 | 2.86E-02 | *Ndufa8, Ndufb2* | mitochondrial electron transport chain | 3.24E-02 |
| Muscle | Blue | Sex | GO:0006364 | rRNA processing | 3/21 | 183/15414 | 2.86E-02 | *Rpl27, Rps14, Rps16* | transcript ssu-rrna tricistronic rrna | 3.05E-02 |
| Muscle | Blue | Sex | GO:0016072 | rRNA metabolic process | 3/21 | 189/15414 | 2.86E-02 | *Rpl27, Rps14, Rps16* | transcript ssu-rrna tricistronic rrna | 3.05E-02 |
| Muscle | Blue | Sex | GO:0042775 | mitochondrial ATP synthesis coupled electron transport | 2/21 | 52/15414 | 2.86E-02 | *Cox5a, Ndufa8* | mitochondrial electron transport chain | 3.24E-02 |
| Muscle | Blue | Sex | GO:0009206 | purine ribonucleoside triphosphate biosynthetic process | 2/21 | 53/15414 | 2.86E-02 | *Atp5l, Atp5g3* | ribonucleoside triphosphate metabolic process | 2.89E-02 |
| Muscle | Blue | Sex | GO:0009145 | purine nucleoside triphosphate biosynthetic process | 2/21 | 54/15414 | 2.86E-02 | *Atp5l, Atp5g3* | ribonucleoside triphosphate metabolic process | 2.89E-02 |
| Muscle | Blue | Sex | GO:0042773 | ATP synthesis coupled electron transport | 2/21 | 54/15414 | 2.86E-02 | *Cox5a, Ndufa8* | mitochondrial electron transport chain | 3.24E-02 |
| Muscle | Blue | Sex | GO:0009201 | ribonucleoside triphosphate biosynthetic process | 2/21 | 56/15414 | 2.88E-02 | *Atp5l, Atp5g3* | ribonucleoside triphosphate metabolic process | 2.89E-02 |
| Muscle | Blue | Sex | GO:0042274 | ribosomal small subunit biogenesis | 2/21 | 62/15414 | 3.31E-02 | *Rps14, Rps16* | transcript ssu-rrna tricistronic rrna | 3.05E-02 |
| Muscle | Blue | Sex | GO:0009142 | nucleoside triphosphate biosynthetic process | 2/21 | 65/15414 | 3.35E-02 | *Atp5l, Atp5g3* | ribonucleoside triphosphate metabolic process | 2.89E-02 |
| Muscle | Blue | Sex | GO:0009205 | purine ribonucleoside triphosphate metabolic process | 2/21 | 66/15414 | 3.35E-02 | *Atp5l, Atp5g3* | ribonucleoside triphosphate metabolic process | 2.89E-02 |
| Muscle | Blue | Sex | GO:1902600 | proton transmembrane transport | 2/21 | 68/15414 | 3.38E-02 | *Atp5l, Atp5g3* | ribonucleoside triphosphate metabolic process | 2.89E-02 |
| Muscle | Blue | Sex | GO:0009199 | ribonucleoside triphosphate metabolic process | 2/21 | 70/15414 | 3.40E-02 | *Atp5l, Atp5g3* | ribonucleoside triphosphate metabolic process | 2.89E-02 |
| Muscle | Blue | Sex | GO:0022904 | respiratory electron transport chain | 2/21 | 74/15414 | 3.41E-02 | *Cox5a, Ndufa8* | mitochondrial electron transport chain | 3.24E-02 |
| Muscle | Blue | Sex | GO:0009144 | purine nucleoside triphosphate metabolic process | 2/21 | 75/15414 | 3.41E-02 | *Atp5l, Atp5g3* | ribonucleoside triphosphate metabolic process | 2.89E-02 |
| Muscle | Blue | Sex | GO:0033108 | mitochondrial respiratory chain complex assembly | 2/21 | 75/15414 | 3.41E-02 | *Ndufa8, Ndufb2* | mitochondrial electron transport chain | 3.24E-02 |
| Muscle | Blue | Sex | GO:0022900 | electron transport chain | 2/21 | 78/15414 | 3.53E-02 | *Cox5a, Ndufa8* | mitochondrial electron transport chain | 3.24E-02 |
| Muscle | Blue | Sex | GO:0042254 | ribosome biogenesis | 3/21 | 268/15414 | 3.73E-02 | *Rpl27, Rps14, Rps16* | transcript ssu-rrna tricistronic rrna | 3.05E-02 |
| Muscle | Blue | Sex | GO:0006119 | oxidative phosphorylation | 2/21 | 88/15414 | 4.13E-02 | *Cox5a, Ndufa8* | mitochondrial electron transport chain | 3.24E-02 |
| Muscle | Blue | Sex | GO:0009141 | nucleoside triphosphate metabolic process | 2/21 | 93/15414 | 4.44E-02 | *Atp5l, Atp5g3* | ribonucleoside triphosphate metabolic process | 2.89E-02 |
| *NA* – not applicable/available | | | | | | | | | | |
