# Supplementary material for: Integration of segmented regression analysis with weighted gene correlation network analysis identifies genes whose expression is remodeled throughout physiological aging in mouse tissues
Source: Aging (Albany NY). 2021 Jul 29;13(14):18150–90. doi: 10.18632/aging.203379 (PMC8351669; doi:10.18632/aging.203379)
Supplement: Supplementary Table 7 [file aging-13-203379-s008.pdf]

Supplementary Table 7. Genes in common between tissues.

Tissue overlap	Genes in common
Heart-Liver	*Map1lc3b*, *Phyh*, *Psmc4*, *Smpd1*, *Yipf3*

Genes considered resulted from the overlap between Trendy genes with hub genes in each tissue. In tissues with more than one significant module (i.e., the heart, the liver, and the muscle), the gene lists consist in the combination of each module's intersection. Relates to the left panel of Figure 5.
