# Supplementary material for: Integration of segmented regression analysis with weighted gene correlation network analysis identifies genes whose expression is remodeled throughout physiological aging in mouse tissues
Source: Aging (Albany NY). 2021 Jul 29;13(14):18150–90. doi: 10.18632/aging.203379 (PMC8351669; doi:10.18632/aging.203379)
Supplement: Supplementary Table 8 [file aging-13-203379-s009.docx]

**Supplementary Table 8. Gene Ontology (GO) term overlap between the brain, heart, liver, and muscle.** GO terms with FDR adjusted *p-values* less than 0.05 were considered for analysis. Relates to the right panel of Figure 5 and to Table 2. When gene similarity between two nodes (GO terms) is below 0.7 or in cases where only one GO term is in common between two or more tissues, the summary network is the same as the full network.

| **Brain-Liver** | | | | | | | |
| --- | --- | --- | --- | --- | --- | --- | --- |
| **GO ID** | **Description** | **Genes** | **Summary network description** | **Module - Brain** | **Module - Liver** | **WGCNA expression trend - Brain** | **WGCNA expression trend - Liver** |
| GO:0002250 | adaptive immune response | *C4b, H2-K1, C3, H2-D1, Irf7, Ctsh, Lag3, B2m, Slc11a1, C1qa, Tap2, Il33, C1qc, C1qb, H2-T23, Ighm, Jchain, Igkc, Cd19, Cd79a, Iglc2, Cd79b, Slamf7, H2-Eb1, H2-Aa* | response immune mediated immunoglobulin | Tan | Darkturquoise  -  Salmon | Increase | Increase |
| GO:0002253 | activation of immune response | *C4b, C3, Lgals3, C1qa, C1qc, C1qb, Ighm, Igkc, Cd19, Cd79a, Iglc2, Cd79b* | response immune mediated immunoglobulin | Tan | Darkturquoise | Increase | Increase |
| GO:0002440 | production of molecular mediator of immune response | *B2m, Slc11a1, Il33, H2-T23, Igkv3-5, Ighm, Mzb1* | regulation production cell proliferation | Tan | Darkturquoise | Increase | Increase |
| GO:0002443 | leukocyte mediated immunity | *C4b, H2-K1, C3, H2-D1, Itgb2, Irf7, Ctsh, Lag3, B2m, Slc11a1, C1qa, Tap2, C1qc, C1qb, H2-T23, Ighm, Igkc, Cd19, Iglc2* | response immune mediated immunoglobulin | Tan | Darkturquoise | Increase | Increase |
| GO:0002449 | lymphocyte mediated immunity | *C4b, H2-K1, C3, H2-D1, Irf7, Ctsh, Lag3, B2m, Slc11a1, C1qa, Tap2, C1qc, C1qb, H2-T23, Ighm, Igkc, Cd19, Iglc2* | response immune mediated immunoglobulin | Tan | Darkturquoise | Increase | Increase |
| GO:0002455 | humoral immune response mediated by circulating immunoglobulin | *C3, C1qa, C1qc, C1qb, Ighm, Igkc, Iglc2* | response immune mediated immunoglobulin | Tan | Darkturquoise | Increase | Increase |
| GO:0002460 | adaptive immune response based on somatic recombination of immune receptors built from immunoglobulin superfamily domains | *C4b, H2-K1, C3, H2-D1, Irf7, Ctsh, B2m, Slc11a1, C1qa, Tap2, Il33, C1qc, C1qb, H2-T23, Ighm, Igkc, Cd19, Iglc2* | response immune mediated immunoglobulin | Tan | Darkturquoise | Increase | Increase |
| GO:0002478 | antigen processing and presentation of exogenous peptide antigen | *H2-K1, B2m, Tap2, H2-T23, Tapbp, H2-Eb1, H2-Aa* | processing presentation peptide antigen | Tan | Salmon | Increase | Increase |
| GO:0002637 | regulation of immunoglobulin production | *Il33, H2-T23, Ighm, Mzb1* | regulation production cell proliferation | Tan | Darkturquoise | Increase | Increase |
| GO:0002673 | regulation of acute inflammatory response | *C3, H2-T23, Ccl5* | regulation production cell proliferation | Tan | Salmon | Increase | Increase |
| GO:0002675 | positive regulation of acute inflammatory response | *C3, H2-T23, Ccl5* | regulation production cell proliferation | Tan | Salmon | Increase | Increase |
| GO:0002697 | regulation of immune effector process | *H2-K1, C3, H2-D1, Lgals3, Itgb2, Lag3, B2m, Tap2, Il33, H2-T23, Ighm, Mzb1* | regulation production cell proliferation | Tan | Darkturquoise | Increase | Increase |
| GO:0002700 | regulation of production of molecular mediator of immune response | *B2m, Il33, H2-T23, Ighm, Mzb1* | regulation production cell proliferation | Tan | Darkturquoise | Increase | Increase |
| GO:0006909 | phagocytosis | *C3, Itgb2, Slc11a1, Ighm, Igkc, Iglc2* | response immune mediated immunoglobulin | Tan | Darkturquoise | Increase | Increase |
| GO:0006956 | complement activation | *C4b, C3, C1qa, C1qc, C1qb, Ighm, Igkc, Iglc2* | response immune mediated immunoglobulin | Tan | Darkturquoise | Increase | Increase |
| GO:0006958 | complement activation, classical pathway | *C3, C1qa, C1qc, C1qb, Ighm, Igkc, Iglc2* | response immune mediated immunoglobulin | Tan | Darkturquoise | Increase | Increase |
| GO:0006959 | humoral immune response | *C4b, C3, C1qa, C1qc, C1qb, H2-T23, Ighm, Jchain, Igkc, Iglc2* | response immune mediated immunoglobulin | Tan | Darkturquoise | Increase | Increase |
| GO:0007159 | leukocyte cell-cell adhesion | *Lgals3, Itgb2, Lag3, Cx3cr1, H2-T23, Ccl5, H2-Aa* | regulation production cell proliferation | Tan | Salmon | Increase | Increase |
| GO:0016064 | immunoglobulin mediated immune response | *C4b, C3, Irf7, C1qa, C1qc, C1qb, H2-T23, Ighm, Igkc, Cd19, Iglc2* | response immune mediated immunoglobulin | Tan | Darkturquoise | Increase | Increase |
| GO:0019724 | B cell mediated immunity | *C4b, C3, Irf7, C1qa, C1qc, C1qb, H2-T23, Ighm, Igkc, Cd19, Iglc2* | response immune mediated immunoglobulin | Tan | Darkturquoise | Increase | Increase |
| GO:0019882 | antigen processing and presentation | *H2-K1, Ctss, H2-D1, Psmb8, B2m, Slc11a1, Tap2, H2-T23, Tapbp, H2-Eb1, H2-Aa* | processing presentation peptide antigen | Tan | Salmon | Increase | Increase |
| GO:0019884 | antigen processing and presentation of exogenous antigen | *H2-K1, B2m, Tap2, H2-T23, Tapbp, H2-Eb1, H2-Aa* | processing presentation peptide antigen | Tan | Salmon | Increase | Increase |
| GO:0022407 | regulation of cell-cell adhesion | *Lgals3, Itgb2, Lag3, H2-T23, Ccl5, H2-Aa* | regulation production cell proliferation | Tan | Salmon | Increase | Increase |
| GO:0030101 | natural killer cell activation | *Itgb2, H2-T23, Slamf7* | regulation production cell proliferation | Tan | Salmon | Increase | Increase |
| GO:0032943 | mononuclear cell proliferation | *Csf1, Lgals3, Itgb2, Slc11a1, H2-T23, Ighm, Cd19, Cd79a, Mzb1, Ccl5, H2-Aa* | regulation production cell proliferation | Tan | Darkturquoise  -  Salmon | Increase | Increase |
| GO:0032944 | regulation of mononuclear cell proliferation | *Csf1, Lgals3, H2-T23, Ighm, Mzb1, Ccl5, H2-Aa* | regulation production cell proliferation | Tan | Darkturquoise  -  Salmon | Increase | Increase |
| GO:0042098 | T cell proliferation | *Lgals3, Itgb2, Slc11a1, H2-T23, Ccl5, H2-Aa* | regulation production cell proliferation | Tan | Salmon | Increase | Increase |
| GO:0042110 | T cell activation | *Lgals3, Itgb2, Lag3, B2m, Slc11a1, H2-T23, Ccl5, H2-Aa* | regulation production cell proliferation | Tan | Salmon | Increase | Increase |
| GO:0042742 | defense response to bacterium | *H2-K1, Lyz2, Gbp3, Slc11a1, H2-T23, Ighm, Jchain, Igkc, Iglc2* | response immune mediated immunoglobulin | Tan | Darkturquoise | Increase | Increase |
| GO:0045123 | cellular extravasation | *Itgb2, Cx3cr1, Ccl5* | regulation production cell proliferation | Tan | Salmon | Increase | Increase |
| GO:0046651 | lymphocyte proliferation | *Lgals3, Itgb2, Slc11a1, H2-T23, Ighm, Cd19, Cd79a, Mzb1, Ccl5, H2-Aa* | regulation production cell proliferation | Tan | Darkturquoise  -  Salmon | Increase | Increase |
| GO:0048002 | antigen processing and presentation of peptide antigen | *H2-K1, Ctss, H2-D1, B2m, Slc11a1, Tap2, H2-T23, Tapbp, H2-Eb1, H2-Aa* | processing presentation peptide antigen | Tan | Salmon | Increase | Increase |
| GO:0048246 | macrophage chemotaxis | *Csf1, Lgals3, Cx3cr1, Ccl5* | regulation production cell proliferation | Tan | Salmon | Increase | Increase |
| GO:0070661 | leukocyte proliferation | *Csf1, Lgals3, Itgb2, Slc11a1, Il33, H2-T23, Ighm, Cd19, Cd79a, Mzb1, Ccl5, H2-Aa* | regulation production cell proliferation | Tan | Darkturquoise  -  Salmon | Increase | Increase |
| GO:0070663 | regulation of leukocyte proliferation | *Csf1, Lgals3, H2-T23, Ighm, Mzb1, Ccl5, H2-Aa* | regulation production cell proliferation | Tan | Darkturquoise  -  Salmon | Increase | Increase |
| GO:0070997 | neuron death | *Csf1, Ctsz, C1qa, Cx3cr1, Ntrk2, Ccl5* | regulation production cell proliferation | Tan | Salmon | Increase | Increase |
| GO:0071674 | mononuclear cell migration | *Csf1, Lgals3, Ccl5* | regulation production cell proliferation | Tan | Salmon | Increase | Increase |
| GO:0071675 | regulation of mononuclear cell migration | *Csf1, Lgals3, Ccl5* | regulation production cell proliferation | Tan | Salmon | Increase | Increase |
| GO:1901214 | regulation of neuron death | *Csf1, Ctsz, C1qa, Cx3cr1, Ntrk2, Ccl5* | regulation production cell proliferation | Tan | Salmon | Increase | Increase |
| GO:1902105 | regulation of leukocyte differentiation | *Csf1, Lag3, C1qc, Ccl5, H2-Aa* | regulation production cell proliferation | Tan | Salmon | Increase | Increase |
| GO:1903037 | regulation of leukocyte cell-cell adhesion | *Lgals3, Itgb2, Lag3, H2-T23, Ccl5, H2-Aa* | regulation production cell proliferation | Tan | Salmon | Increase | Increase |
| GO:1903706 | regulation of hemopoiesis | *Csf1, Lag3, B2m, C1qc, Ccl5, H2-Aa* | regulation production cell proliferation | Tan | Salmon | Increase | Increase |
| GO:1905517 | macrophage migration | *Csf1, Lgals3, Cx3cr1, Ccl5* | regulation production cell proliferation | Tan | Salmon | Increase | Increase |
| **Brain-Muscle** | | | | | | | |
| **GO ID** | **Description** | **Genes** | **Summary network description** | **Module - Brain** | **Module - Muscle** | **WGCNA expression trend - Brain** | **WGCNA expression trend - Muscle** |
| GO:0001525 | angiogenesis | *C3, Lgals3, Itgb2, Ctsh, Cx3cr1, Fn1, Serpinf1, Sparc, Cd34, Anxa2* | angiogenesis | Tan | Brown | Increase | Decrease |
| GO:0008347 | glial cell migration | *Csf1, Hexb, Fn1, Ndn, Lrp1* | glial cell migration | Tan | Brown | Increase | Decrease |
| GO:0009611 | response to wounding | *Gfap, C3, Neat1, Slc11a1, Col3a1, Col5a1, Fn1, Cd34, Serping1, Anxa2, Lrp1* | extracellular structure organization response | Tan | Brown | Increase | Decrease |
| GO:0030198 | extracellular matrix organization | *Gfap, Ctss, Lgals3, Col3a1, Col5a2, Col5a1, Fn1, Nid1, Tgfbi, Anxa2* | extracellular structure organization response | Tan | Brown | Increase | Decrease |
| GO:0043062 | extracellular structure organization | *Gfap, Ctss, Lgals3, Col3a1, Col5a2, Col5a1, Fn1, Nid1, Tgfbi, Anxa2* | extracellular structure organization response | Tan | Brown | Increase | Decrease |
| GO:0052547 | regulation of peptidase activity | *Serpina3n, Ctsh, Psmb8, Ctsd, Serpinf1, Pi16, Serping1, Timp2* | regulation peptidase activity | Tan | Brown | Increase | Decrease |
| **Heart-Muscle** | | | | | | | |
| **GO ID** | **Description** | **Genes** | **Summary network description** | **Module - Heart** | **Module - Muscle** | **WGCNA expression trend - Heart** | **WGCNA expression trend - Muscle** |
| GO:0006119 | oxidative phosphorylation | Coq9, Uqcrc1, Cyc1, Ndufa10, Ndufs2, Sdhd, Cox5a, Ndufa8 | respiratory electron transport atp | Blue | Blue | Decrease | Increase (males) |
| GO:0022900 | electron transport chain | Coq9, Slc25a12, Uqcrc1, Cyc1, Ndufa10, Ndufs2, Sdhd, Cox5a, Ndufa8 | respiratory electron transport atp | Blue | Blue | Decrease | Increase (males) |
| GO:0022904 | respiratory electron transport chain | Coq9, Slc25a12, Uqcrc1, Cyc1, Ndufa10, Ndufs2, Sdhd,Cox5a, Ndufa8 | respiratory electron transport atp | Blue | Blue | Decrease | Increase (males) |
| GO:0042773 | ATP synthesis coupled electron transport | Coq9, Uqcrc1, Cyc1, Ndufa10, Ndufs2, Sdhd,Cox5a, Ndufa8 | respiratory electron transport atp | Blue | Blue | Decrease | Increase (males) |
| GO:0042775 | mitochondrial ATP synthesis coupled electron transport | Coq9, Uqcrc1, Cyc1, Ndufa10, Ndufs2, Sdhd,Cox5a, Ndufa8 | respiratory electron transport atp | Blue | Blue | Decrease | Increase (males) |
| GO:0046034 | ATP metabolic process | Eno3, Coq9, Uqcrc1, Cyc1, Ndufa10, Ndufs2, Sdhd,Atp5l, Atp5g3, Cox5a, Ndufa8 | respiratory electron transport atp | Blue | Blue | Decrease | Increase (males) |
| **Liver-Heart** | | | | | | | |
| **GO ID** | **Description** | **Genes** | **Summary network description** | **Module - Liver** | **Module - Heart** | **WGCNA expression trend - Liver** | **WGCNA expression trend - Heart** |
| GO:0031958 | corticosteroid receptor signaling pathway | *Ppp5c, Phb, Ntrk2* | receptor signaling pathway glucocorticoid | Salmon | Blue | Increase | Decrease |
| GO:0042921 | glucocorticoid receptor signaling pathway | *Ppp5c, Phb, Ntrk2* | receptor signaling pathway glucocorticoid | Salmon | Blue | Increase | Decrease |
| GO:2000322 | regulation of glucocorticoid receptor signaling pathway | *Ppp5c, Phb, Ntrk2* | receptor signaling pathway glucocorticoid | Salmon | Blue | Increase | Decrease |
| **Liver-Muscle** | | | | | | | |
| **GO ID** | **Description** | **Genes** | **Summary network description** | **Module - Liver** | **Module - Muscle** | **WGCNA expression trend - Liver** | **WGCNA expression trend - Muscle** |
| GO:0071230 | cellular response to amino acid stimulus | *Ntrk2, Col3a1, Col5a2, Col6a1* | cellular response to amino acid stimulus | Salmon | Brown | Increase | Decrease |
