# Supplementary material for: Integration of segmented regression analysis with weighted gene correlation network analysis identifies genes whose expression is remodeled throughout physiological aging in mouse tissues
Source: Aging (Albany NY). 2021 Jul 29;13(14):18150–90. doi: 10.18632/aging.203379 (PMC8351669; doi:10.18632/aging.203379)
Supplement: Supplementary Table 9 [file aging-13-203379-s010.pdf]

Supplementary Table 9. Sample characterization.

Brain

Age (mo.)	Initial no. of samples			No. of samples after removal of low coverage samples			No. of samples after removal of outlier samples		
	Male	Female	Total	Male	Female	Total	Male	Female	Total
3	4	2	6	4	2	6	4	2	6
6	4	2	6	4	2	6	4	2	6
9	4	2	6	4	2	6	4	1	5
12	4	2	6	4	2	6	4	2	6
15	4	2	6	4	2	6	4	1	5
18	4	2	6	4	2	6	4	2	6
21	4	2	6	3	2	5	3	2	5
24	4	0	4	4	0	4	4	0	4
27	4	0	4	3	0	3	3	0	3

Heart

Age (mo.)	Initial no. of samples			No. of samples after removal of low coverage samples			No. of samples after removal of outlier samples		
	Male	Female	Total	Male	Female	Total	Male	Female	Total
3	4	1	5	4	1	5	4	1	5
6	4	2	6	4	1	5	4	1	5
9	4	2	6	4	2	6	4	2	6
12	4	2	6	4	2	6	4	1	5
15	4	2	6	4	2	6	4	2	6
18	3	2	5	3	2	5	3	1	4
21	4	2	6	4	2	6	4	2	6
24	4	0	4	4	0	4	4	0	4
27	4	0	4	4	0	4	4	0	4

Liver

Age (mo.)	Initial no. of samples			No. of samples after removal of low coverage samples			No. of samples after removal of outlier samples		
	Male	Female	Total	Male	Female	Total	Male	Female	Total
3	4	2	6	4	2	6	4	2	6
6	4	2	6	4	2	6	4	2	6
9	4	2	6	3	1	4	3	1	4
12	4	2	6	4	2	6	4	2	6
15	4	2	6	4	2	6	4	2	6
18	4	2	6	3	2	5	3	2	5
21	4	2	6	4	2	6	4	1	5
24	3	0	3	3	0	3	3	0	3
27	4	0	4	4	0	4	3	0	3

Muscle

Age (mo.)	Initial no. of samples			No. of samples after removal of low coverage samples			No. of samples after removal of outlier samples		
	Male	Female	Total	Male	Female	Total	Male	Female	Total
3	4	2	6	4	2	6	4	2	6
6	3	2	5	3	2	5	3	1	4
9	4	2	6	4	2	6	4	2	6
12	4	2	6	4	2	6	3	2	5
15	4	2	6	4	2	6	4	2	6
18	4	2	6	4	2	6	4	2	6
21	4	2	6	3	2	5	3	2	5
24	3	0	3	3	0	3	2	0	2
27	4	0	4	4	0	4	3	0	3

Pancreas

Age (mo.)	Initial no. of samples			No. of samples after removal of low coverage samples			No. of samples after removal of outlier samples		
	Male	Female	Total	Male	Female	Total	Male	Female	Total
3	4	2	6	3	0	3	3	0	3
6	4	2	6	3	1	4	3	1	4

9	4	2	6	2	2	4	2	2	4
12	4	2	6	3	2	5	3	2	5
15	4	2	6	4	1	5	3	1	4
18	4	2	6	2	2	4	2	1	3
21	4	2	6	2	1	3	2	1	3
24	4	0	4	2	0	2	2	0	2
27	4	0	4	4	0	4	3	0	3

mo. – months; *no.* – number.
